# Repair-Mediated Duplication by Capture of Proximal Chromosomal DNA Has Shaped Vertebrate Genome Evolution

**DOI:** 10.1371/journal.pgen.1000469

**Published:** 2009-05-08

**Authors:** John K. Pace, Shurjo K. Sen, Mark A. Batzer, Cédric Feschotte

**Affiliations:** 1 Department of Biology, University of Texas at Arlington, Arlington, Texas, United States of America; 2 Department of Biological Sciences, Louisiana State University, Baton Rouge, Louisiana, United States of America; Stanford University, United States of America

## Abstract

DNA double-strand breaks (DSBs) are a common form of cellular damage that can lead to cell death if not repaired promptly. Experimental systems have shown that DSB repair in eukaryotic cells is often imperfect and may result in the insertion of extra chromosomal DNA or the duplication of existing DNA at the breakpoint. These events are thought to be a source of genomic instability and human diseases, but it is unclear whether they have contributed significantly to genome evolution. Here we developed an innovative computational pipeline that takes advantage of the repetitive structure of genomes to detect repair-mediated duplication events (RDs) that occurred in the germline and created insertions of at least 50 bp of genomic DNA. Using this pipeline we identified over 1,000 probable RDs in the human genome. Of these, 824 were intra-chromosomal, closely linked duplications of up to 619 bp bearing the hallmarks of the synthesis-dependent strand-annealing repair pathway. This mechanism has duplicated hundreds of sequences predicted to be functional in the human genome, including exons, UTRs, intron splice sites and transcription factor binding sites. Dating of the duplication events using comparative genomics and experimental validation revealed that the mechanism has operated continuously but with decreasing intensity throughout primate evolution. The mechanism has produced species-specific duplications in all primate species surveyed and is contributing to genomic variation among humans. Finally, we show that RDs have also occurred, albeit at a lower frequency, in non-primate mammals and other vertebrates, indicating that this mechanism has been an important force shaping vertebrate genome evolution.

## Author Summary

The repair of DNA double-strand breaks (DSBs) is essential for the maintenance of genome integrity. The mechanisms by which DSBs are repaired have been the subject of intense experimental investigations. It has emerged that several imperfect repair pathways exist in eukaryotes that have the potential to result in chromosomal alterations, including genomic duplications. However, it remains unclear to what extent these imperfect repair events have contributed to shaping genomes throughout evolution. Here we introduce an innovative computational approach that takes advantage of the repetitive nature of eukaryotic genomes to identify repair-mediated duplications (RD) that occurred during evolution. We discovered over one thousand RDs in the human genome, with two-thirds resulting from the capture of a chromosomal DNA segment located in close proximity to the presumed site of the DSB, giving rise to local genomic duplications. Comparative genomic analyses reveal that the mechanism has operated continuously, but with decreasing intensity during primate evolution, generating species-specific duplications in all primates surveyed and generating genomic variation among humans. Finally, we show that RDs have also occurred in non-primate mammals and other vertebrates, indicating that this is a previously under-appreciated force shaping vertebrate genomes.

## Introduction

**Figure 1:**
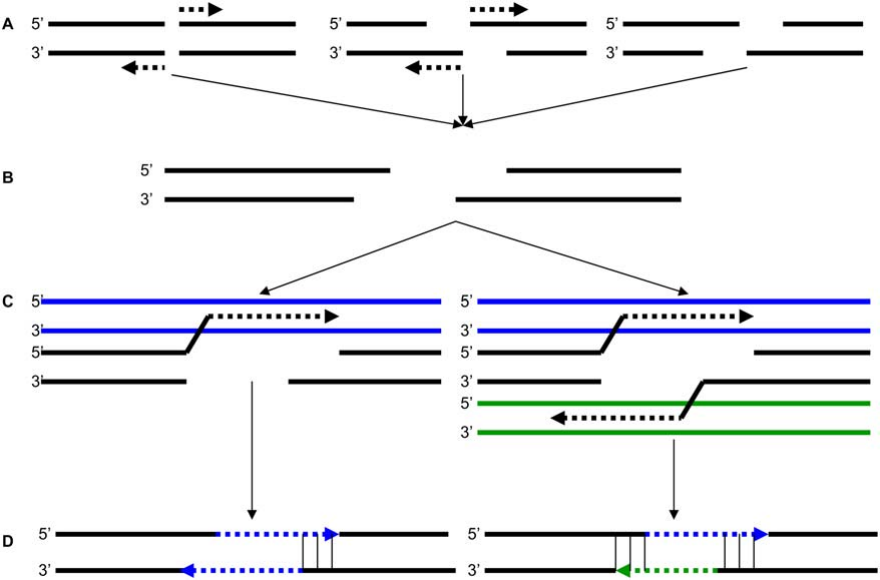
SDSA DSB repair pathway. (A) A DNA molecule suffers a double-strand break. The DSB can result in either blunt ends (left), 5′ overhanging ends (center), or 3′ overhanging ends (left). After the break occurs, exonuclease activity (dotted arrow) creates 3′ overhanging ends at the site of the lesion by removing nucleotides from blunt breaks (left) or breaks with 5′ overhanging ends (center). (B) After the exonuclease activity ceases, the resulting breakpoint has 3′ overhangs at both ends of the lesion. (C) In the SDSA pathway, one 3′ overhanging end can invade another DNA molecule, annealing to a sequence that is complementary, and repair synthesis begins. The other 3′ overhanging end may invade a different DNA molecule and begin repair synthesis as well. (D) After repair synthesis is completed and the new strand has dissociated from the template strand, it anneals with the other end of the initial lesion at a complementary region, creating the duplication of one template sequence (left). If both 3′ ends of the lesion used different template strands for repair synthesis, both strands will anneal at a complementary region, resulting in the duplication of two different template sequences (right).

Environmental agents and normal cellular metabolic processes produce DNA double-strand breaks (DSBs) that can lead to cell death if not repaired [1]. Eukaryotic cells have evolved DSB repair mechanisms that can be classified into two broad categories: homologous recombination (HR) and non-homologous end joining (NHEJ). The canonical HR pathway uses long stretches of homology between the flanking sequences at the site of breakage and the homologous chromosome or sister chromatid to repair DSBs perfectly, leaving no evidence that a break ever occurred. Two other forms of DSB repair, single strand annealing (SSA) and synthesis-dependent strand annealing (SDSA), can also be classified as types of HR. NHEJ repairs the DSB without the use of a repair template and can create deletions or insertions [2] at the site of the lesion (Figure 1) [1],[3].

Previous empirical studies have provided a detailed characterization of the breakpoints produced by imperfect DSB repair mechanisms in eukaryotic cells. In vivo and ex vivo systems designed to track the fate of experimentally induced DSBs in yeast, fly, plant and mammalian cells have shown that imperfect repair is often accompanied by the insertion of extra, “captured” DNA at the breakpoint [4]–[12]. Several studies have found that this captured DNA is a duplication of sequences that have homology with the experimentally induced breakpoints such as (1) a different chromosome [13],[14], (2) extra chromosomal molecules such as plasmids or mitochondrial DNA [7],[10],[11],[15], (3) cDNA copies of retrotransposons [11],[16] or (4) a nearby sequence on the same chromosome [6],[17]. One such mechanism for “capturing,” and thereby duplicating a sequence during DSB repair, is the SDSA pathway.

SDSA occurs when nucleotides on one of the overhanging 3′ end of a DSB anneal with a complementary sequence that serves as a template for synthesis. This template may be either homologous or ectopic. Once synthesis is completed, the invading strand is displaced and anneals to the other resected 3′ end of the DSB. If an ectopic template is used, the process results in a conservative, repair-mediated duplication of the template sequence. If an homologous template is used, no duplication occurs. Additionally, both ends of the break are free to anneal with different templates, initiate repair synthesis, then re-anneal with each other. In this case, the duplication of two different templates can occur at the site of breakage (Figure 1) [1].

Though studies have experimentally observed and characterized repair-mediated duplications (RDs), the genome-wide scope and potential impact of these duplications upon vertebrate evolution has not been investigated. Therefore, we sought to computationally identify duplications in vertebrates whose breakpoints bear the signatures of imperfect repair events and assess their potential impact upon genome evolution. Our analysis focused on primates (human, chimpanzee, orangutan, Rhesus macaque, marmoset), but also chicken, zebrafish and other mammals (mouse, rat, dog, cow). We recovered 824 RDs in the human genome, 15 of which were found to be specific to the human lineage, as they are absent from the chimpanzee, orangutan, Rhesus macaque and marmoset. We confirmed experimentally that one of the human-specific RDs remains polymorphic in the general population. Lineage-specific RDs were found in all genomes for which a closely related ancestor was available as outgroup. Thus, RDs are a previously under-appreciated force shaping vertebrate genomes and generating structural genomic variation among humans.

## Results

### Identification of Potential RDs

**Figure 2:**
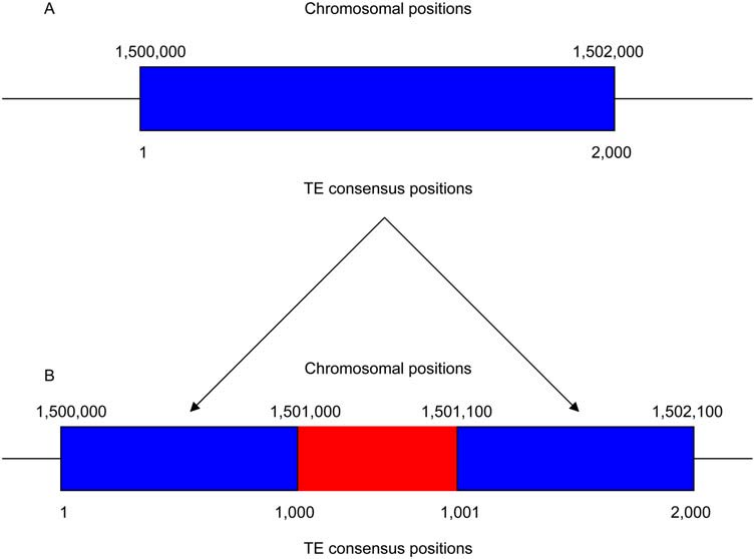
Identification of interrupted TEs. (A) Annotation of an uninterrupted TE (blue rectangle) along a chromosome. The chromosomal location is shown above the rectangle, while the positions of the TE consensus sequence are shown below. (B) Annotation of an interrupted TE. After the insertion of a new sequence (red rectangle), the two TE fragments are separated along the chromosome.

In order to find duplications whose breakpoints bear the previously characterized hallmarks of imperfect repair events, but not those of other known duplication mechanisms (such as retrotransposition), we developed a novel computational approach that capitalizes upon the repetitive nature of eukaryotic genomes. In humans and other primates, about 45% of the genome is composed of interspersed repeats that derive from the activity of a limited number of transposable element (TE) families [18]. Since the derived ancestral consensus sequence for any family of TEs is known, the insertion of a sequence within a TE can be found by locating fragments of annotated TEs that are separated by an intervening sequence that is not alignable with the consensus sequence (Figure 2).

Drawing upon the wealth of TE annotation available, our computational pipeline began with a Perl script that parsed the human RepeatMasker (rmsk) annotation files (hg18 assembly, http://genome.ucsc.edu) to identify TEs that were interrupted by an insertion of at least 50 bp. The first pass returned over 492,000 such insertions, the majority of which were identified as transposon insertions. A small proportion of these insertions could be classified as processed retrogenes or LINE1 (L1)-mediated transduction events and, like the nested transposon insertions, were filtered out (see Materials and Methods). Next, we excluded inserted sequences not identified as any of the above types of duplications, but found at more than one other location in the genome or where a second, parental copy of the insertion could not be confidently identified in the available nuclear genome sequence. In addition, we found 113 insertions that mapped within an annotated segmental duplication (SD) for which we could not confidently identify the parental copy. As such, these insertions were also filtered out from the data set. Finally, we removed any cases where the inserted sequence and putative template sequence were within 50 bp of each other to exclude tandem duplications, which are typically formed by a mechanism other than DSB repair [19]. The remaining dataset included 1,136 interrupted TEs that had suffered the insertion of a sequence found at only one other location in the genome, each of which may represent DNA captured via SDSA at former sites of DSB.

**Figure 3:**
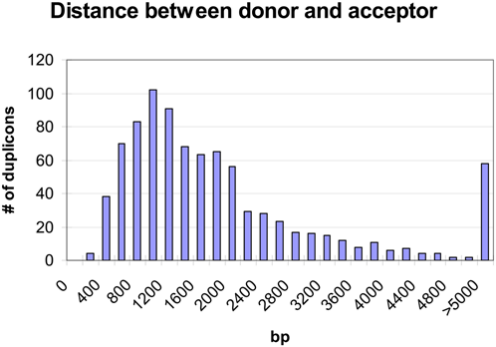
Chromosomal distance between donor and acceptor sequences located on the same chromosome in human.

Strikingly, for 824 of the 1,136 duplicons, the donor sequence was located within 5 kb of the acceptor, with 753 (66%) duplicons separated by less than 3 kb. The remaining 312 duplicons were separated by more than 5 kb (n = 58, max = 122 Mb) or were located on different chromosomes (n = 254). A histogram of the distances between donors and acceptors located on the same chromosome demonstrated a seeming peak in the distance separating the duplicons at approximately 1,200 bp (Figure 3). However, we note that this peak may be an artifact of our method, as we used TEs as markers to identify acceptor sequences. Since the acceptor is located within a TE, the donor and acceptor must be separated by at least the length of the TE fragment the acceptor is located in. However, a long tail at the right side of the histogram was also apparent, as the maximum distance between donor and acceptor was 122 Mb. This clearly indicated that acceptor and donor sequences are more likely to be located within close range of each other than widely separated. For increased readability, distances between donor and acceptor of greater than 5 kb were combined into one bin (>5000, Figure 3). We used this ad-hoc cutoff to split the duplicons into two groups: one where the donor and acceptor were within 5 kb of each other (proximal duplications) and the other where the donor and acceptor were separated by >5 kb or were on different chromosomes (distant duplications). We noticed that the two groups were also distinguishable by the length of the duplicated (acceptor) sequence, which is shorter for proximal duplications (mean = 162 bp, median = 147 bp, max. = 619 bp) than for distant duplications (mean = 302 bp, median = 204 bp, max. = 3,424 bp), a statistically significant difference (Student's t-test, p<0.001).

**Figure 4:**
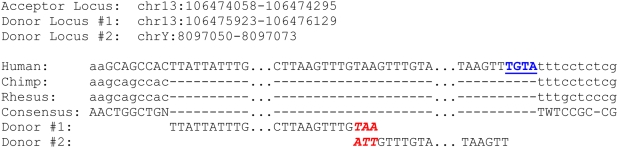
RD where acceptor is a chimera of two different donor sequences. Pre-insertion empty sites for the human-specific RD are shown for chimp and Rhesus macaque. The sequences for donor #1 and donor #2 are shown at the bottom, with a short stretch of base complementarity between the two donors in red, bold, and italics. The filler sequence is in blue, bold, and underlined.

Further examination of the acceptor sequences revealed that in 20 instances, the acceptor was a chimera of 2 different donor sequences (Table S1), as predicted by the SDSA model of DSB repair and observed previously at experimentally induced DSBs [14]. For every chimeric acceptor case, at least one of the donor sequences was located within 5 kb of the acceptor. In five cases, the second donor was within 3.5 kb of the first donor (range = 289–3,432 bp), while in the other 15 cases, the second donor was located on a different chromosome. This type of chimeric duplication may be formed when the 3′ overhanging ends at the site of DSB invade different template strands and reanneal at a stretch of microhomology (Figure 1). One example of such chimeric duplication is shown in Figure 4 together with the “empty” orthologous insertion site in the chimpanzee and macaque genomes, which indicates that this particular duplication is human-specific. Note the short stretch of base complementarity (3 nucleotides) at the presumed site of annealing between the two copied donor sequences, as well as the short filler DNA sequence inserted at one of the duplication breakpoints, another signature of DSB repair (see below).

### Analysis of Duplication Breakpoints

We next focused on precisely defining the breakpoints of the potential RDs in our dataset to determine if the hallmarks of DSB repair were present (Figure S1). For this, we took advantage of the availability of draft genome sequences for four closely related primate species (chimpanzee, orangutan, Rhesus macaque and marmoset) and three non-primate mammals (dog, cow and mouse) available at the UCSC Genome Browser to perform a comparative genomic analysis. We found that 24 of the duplicons were human-specific (present only in human, absent in all other primate species), 67 were hominin-specific (present only in human and chimpanzee), 190 were hominid-specific (present only in human, chimpanzee and orangutan), 289 were catarhinne-specific (present only in human, chimpanzee, orangutan and Rhesus macaque), 513 were primate-specific (present only in primate species) and the remaining 53 were present in all primates and at least one other non-primate mammal (Table S1).

Given the relatively recent divergence of hominids (18 mya), and thus, the short time period for substitutions to accumulate, we focused on precisely defining the breakpoints of RD that occurred in the hominid lineage (Figure S1). We aligned and individually inspected the breakpoints of all human-specific duplications for which orthologous sequences could be unambiguously identified in the other primate species (i.e., 18 out of 24; 14 with the donor <5 kb from the acceptor, 4 where the donor was on a different chromosome) as well as a sample of 50 hominid-specific duplicons (25 randomly selected with the donor <5 kb from the acceptor and 25 randomly selected where the donor was on a different chromosome). We found that 51 of the 68 breakpoints (75%) examined were characterized by the molecular signatures of SDSA events including deletions, “filler” sequences or stretches of microhomology between the flanking sequences of the donor and acceptor (Table 1) [6],[7],[20]. The 17 remaining acceptor loci were characterized by the addition of a polyA or polyT tract of 5 bp or greater at one end of the insertion, indicative of the retrotransposition of processed mRNA by target-primed reverse transcription [21],[22]. Such retrotransposition events are typically accompanied by target site duplications and, indeed, we were able to identify such duplications flanking 12 of the 17 acceptor sites terminating in polyA/T tracts (Table 1). We noticed that none of the 39 acceptor sequences from which the donor was located within 5 kb possessed polyA/T tracts (14 human-specific, 25 hominid-specific), while 17 of the 29 (59%) in the other group did (Table 1). In other words, all proximal duplications (where the donor is <5 kb from the acceptor) bear characteristics consistent with imperfect DSB repair, while a majority of the other, more distant duplications possess characteristics of retrotransposition events. These data indicate that proximal duplications can be confidently classified as RDs, while more distant duplications cannot. Therefore, for the rest of this analysis we focus on the proximal duplication group, yielding a set of 824 probable RDs in the human genome.

**Table 1:** Characteristics of duplication breakpoints.

Breakpoints Characteristics	Intrachromosomal Duplication, Less than 5 kb Apart	Interchromosomal Duplication
Without polyA/T tail		
MH+Del	9	1
MH+Filler	5	1
MH+Del+Filler	10	0
MH+TSD	3	0
MH+TSD+Filler	2	1
MH only	2	0
Filler+Del	5	1
Filler+TSD	0	5
Filler+Del+TSD	1	0
Filler only	2	2
TSD only	0	1
**Total**	**39**	**12**
With polyA/T tail		
MH+Del	0	1
MH+Filler	0	0
MH+Del+Filler	0	0
MH+TSD	0	2
MH+TSD+Filler	0	0
MH only	0	1
Filler+Del	0	0
Filler+TSD	0	2
Filler only	0	0
TSD only	0	8
Del only	0	1
PolyA/T only	0	2
**Total**	**0**	**17**

Breakpoint characteristics and size range in nucleotides: MH = microhomology (2–9 nt), Del = deletion (1–15 nt), Filler = filler sequence (1–68 nt), TSD = target site duplication (4–21 nt), polyA/T = polyA/ or polyT tail of 5 bp or more.

### Rate and Timing of Repair-Mediated Duplication during Primate Evolution

**Figure 5:**
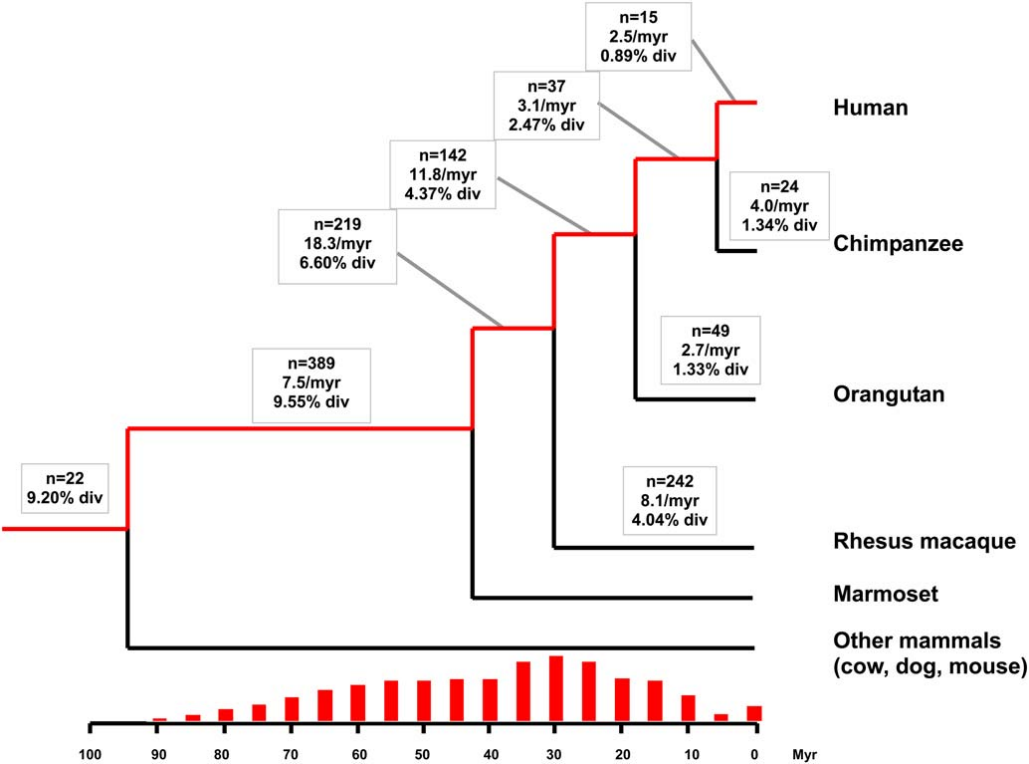
Rate and timing of RD formation. For each branch, the number of RDs created (n), the rate of repair-mediated duplication (per myr), and the average divergence of acceptor and donor pairs (%) during the time period are shown. The timing of RD formation for the human lineage (indicated by the red branches) is shown above the time scale by the red vertical bars. Each individual bar shows the relative proportion of RDs falling within the same, non-overlapping 5-myr bin.

In order to estimate and compare the rate of repair-mediated duplication in different branches of the primate evolutionary tree, we used the same computational pipeline described above to recover chimpanzee-, orangutan- and Rhesus-specific RDs. As for human RDs, we only retained duplicons located within 5 kb of each other as manual inspection of the breakpoints of chimpanzee-specific acceptor sequences for proximal and distant duplicons gave results consistent with those obtained for human (data not shown). From these data, we were able to infer the minimum rate of duplication (i.e., number of repair-mediated duplication events per myr) along different branches of the primate phylogenetic tree over the past ∼40 myr (Figure 5). In the 12 myr separating the divergence of Rhesus macaque from the hominid lineage, the rate was found to be 11.8 RD/myr (142 hominid-specific RDs). However, in the 12 myr between the divergence of orangutan from human and chimpanzee, the rate was only 3.1 RD/myr. The rates in the three hominid species were almost identical (human = 2.5 RD/myr, chimpanzee = 4.0 RD/myr, orangutan = 2.7 RD/myr), while a higher rate was found in the Rhesus macaque lineage (8.1 RD/myr). Thus, there seems to have been a substantial slowdown (about 4 fold) in the rate of repair-mediated duplication events in the hominid lineage as compared to the period predating the divergence of the hominid lineage from Old World monkeys (Figure 5).

A positive correlation was observed between the average sequence divergence between donors and acceptors and the time period at which the duplication event was inferred by the comparative genomic analyses. As expected, the average divergence between donor and acceptor increased as the time since RD formation extended back from the present (Figure 5). The RD divergence values were also in good agreement with the expected sequence divergence between the related species as if the bulk of RDs had evolved at the neutral substitution rate following duplication. For example, the average pairwise divergence between donors and acceptors was 0.89% for human-specific and 1.34% for chimpanzee-specific RDs (Figure 5). Assuming a neutral substitution rate of 2.2×10^−9^ substitutions/yr [23], the expected average divergence for RDs in the human and chimpanzee lineages should be 1.32%. While the average divergence for chimpanzee is almost exactly what is expected, the average divergence is slightly lower than expected for the human-specific RDs. However, the lower than expected divergence in human may be the result of a small sample size (n = 15) and therefore must be viewed with caution.

Interestingly, the average percent divergence of RDs in the orangutan lineage (1.33%) is significantly lower than expected (3.96%) assuming the same neutral substitution rate as in the human and chimpanzee lineages (p<0.05, χ^2^ test). This low percent divergence may be the result of a general slowdown of the neutral substitution rate in the orangutan lineage or of a period of relative quiescence of the mechanism responsible for RD formation early in the orangutan lineage followed by a subsequent increase. Another possible explanation is that a larger fraction of orangutan RDs have evolved under functional constraint following duplication.

### RD Polymorphism and Human Genetic Variation

**Figure 6:**
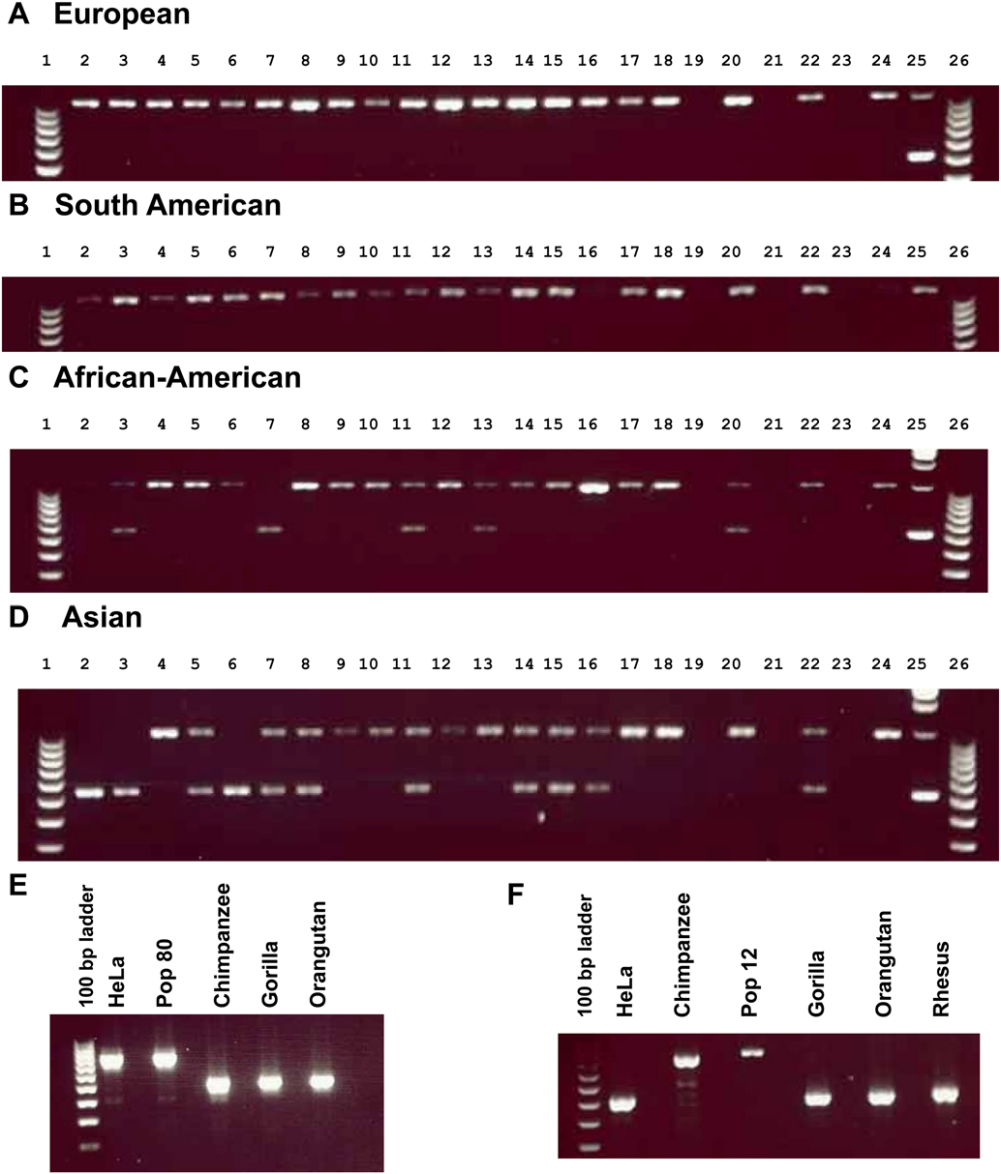
PCR analysis of human-specific and chimpanzee-specific RD. (A–D) PCR genotyping of the human-specific RD at chr15:31,987,740–31,988,005 in the 2006 hg18 assembly. For all 20 European (A) and 20 South American (B) individuals surveyed, the size of the PCR products is consistent with the homozygous presence of the acceptor sequence in all individuals tested. In contrast, among 20 African-American (C) and 20 Asian individuals (D) surveyed, several DNA samples yielded PCR products consistent with the absence of the acceptor sequence, either as homozygous [lanes 4–6 in (C) and 4, 9, and 10 in (D)] or heterozygous [lanes 3, 11, and 13 in (C) and 5, 7, and 8 in (D)]. See Table S2 for full loading orders. (E) Example of PCR validation of human-specific RD (chr10: 96,228,347–96,228,586): PCR products of size consistent with the presence of the acceptor sequence were obtained in human DNA (from HeLa cells or pooled individuals [Pop80]), while shorter PCR products, indicative of the absence of the acceptor sequence, were obtained with chimpanzee, gorilla, and orangutan DNA. (F) Example of PCR validation of chimpanzee-specific RD (chr7:136,854,307–136,854,636): PCR products of size consistent with the presence of the acceptor sequence were obtained with chimpanzee DNA (from single, “chimpanzee,” or pooled individuals [Pop12]), while shorter PCR products, indicative of the absence of the acceptor sequence, were obtained with human (HeLa cells), gorilla, and orangutan DNA.

Identification of 7 human-specific RDs in which the donor and acceptor sequences were identical suggested that these duplications occurred in the very recent past and could still be polymorphic in the human population. To test this hypothesis, we screened 4 of these 7 RDs, along with 6 additional human-specific acceptors in 80 individuals from 4 geographic populations (African-American, Asian, European, and South American) for presence/absence using PCR with primers flanking the insertion. Of these 10 RDs, all but one appeared to be fixed in the human population. In addition, all 10 RDs examined were absent in the chimpanzee, gorilla and orangutan genomes analyzed (see example in Figure 6E), corroborating our computational prediction that they are indeed human-specific. The insertion at chr15:31,987,740–31,988,005 in the hg18 assembly of the human genome, is apparently fixed in all European and South American populations, but remains polymorphic in African-American and Asian populations (Figures 6A–6D). This acceptor sequence was also precisely absent in the Celera human genome assembly, while all other human-specific RDs were present (data not shown).

Next, we screened six chimpanzee-specific acceptors for polymorphism with DNA extracted from 12 unrelated common chimpanzees, but were unable to find any polymorphic RDs. These results may be due to a small chimpanzee sample size (12 individuals) in comparison to the human sample size (80 individuals). Therefore, the possibility cannot be excluded that some chimpanzee-specific RDs are still polymorphic. Each chimpanzee-specific acceptor tested was absent from human, gorilla, orangutan and Rhesus macaque DNA tested (see example in Figure 6F), validating that these RDs are indeed specific to chimpanzees.

### Duplication of Potentially Functional Sequences

We next investigated whether RDs were responsible for the duplication of potentially functional sequences such as exons, predicted transcription factor binding sites and mammalian most conserved sequences (PhastCons, 28-way; Table 2). We found that two complete exons were duplicated, one within the acyl-CoA synthetase bubblegum family member 2 (ACSBG2) gene (exon 7) and the other in a predicted gene of unknown function (C17orf57, exon 20). In the ACSBG2 gene, the duplicated exon inserted within an intron of the same gene, but in the opposite orientation as the donor exon. Transcriptome data indicates that this duplicated exon is transcribed in the opposite orientation relative to the donor exon and appears to form a fusion transcript with an additional non-coding exon located upstream of the ACSBG2 gene boundary (ESTs CD687637, BI912699, BG573431). Although the duplicated exon has preserved an intact open reading frame and high sequence identity (∼94%) to the donor exon, Ka/Ks analysis revealed no evidence of selective constraint acting at the coding level on the duplicated exon since duplication (Ka/Ks not significantly different from 1, p>0.05). No evidence for the transcription of the duplicated exon in the C17orf57 gene could be found, nor any evidence of selective constraint (Ka/Ks not significantly different from 1, p>0.05).

**Table 2:** Potentially functional sequences within acceptor and donor sequences.

Type of Sequence	Nb. in Acceptor	Nb. in Donor
Intron	295	278
3′ UTR	2	11
5′ UTR	0	5
Partial exon/intron	0	4
Full exon	0	2
Intron/Full exon/intron	0	2
Mammalian most conserved sequence (PhastCons)	45	82
Predicted transcription factor binding site	22	21

Of the 824 donor sequences, 22 contained sequences annotated on the UCSC Genome Browser as predicted transcription factor binding sites (TFBS). In 21 instances, the same TFBS was also computationally predicted within the corresponding acceptor. Seven of the duplicated TFBS display 100% nucleotide identity between the donor and acceptor, while the other 14 display an average of 86% identity. These data suggest that RD represents a possible mechanism for locally duplicating TFBS, thereby potentially contributing to evolution of genomic regulation.

Mammalian most conserved (PhastCons) sequences are DNA segments that are significantly more conserved between distant mammalian species than expected under a neutral model of sequence evolution, suggesting that these sequences correspond to functional elements evolving under purifying selection [24],[25]. Of the 824 RD donors, 82 contained at least one full-length PhastCons segment. With the exception of the two aforementioned complete exons and two additional exons partially duplicated, these conserved segments map to non-coding sequences, some of which may possess regulatory functions. In most cases, the duplicated sequences have retained high identity (>90%) with the donor sequence, which suggests that RD is a potent mechanism for the emergence of new functional elements.

### Genomic Distribution of Repair-Mediated Duplications

In principle, RDs may arise anywhere a DSB occurs. Since DSBs can occur on any human chromosome, and assuming that the SDSA pathway can generate RD on any chromosome, we would expect to find RDs on all human chromosomes. Indeed, we were able to identify RDs on all human chromosomes, except the Y chromosome (see Figure S2). To assess whether RDs are equally distributed among chromosomes, we performed Monte Carlo simulations to determine the expected number of RDs per chromosome based upon the percentage of the total genomic DNA accounted for by each chromosome (see Materials and Methods). The distribution of RDs per chromosome did not significantly differ from the expected value (p>0.05 after Bonferroni correction applied).

To ensure that these results were not an artifact of our computational method of finding RDs within TEs, we performed the same simulations as above but calculated the expected number of RDs per chromosome based upon the percentage of TE DNA on each chromosome (see Materials and Methods). Using this method, we discovered a statistically significant deficit of RDs on chromosome X (obs = 29, exp = 53, p<0.0001).

In an attempt to investigate the potential factors underlying this bias, we first looked at a possible inverse correlation between RD and gene densities. This observation might indicate that RDs in gene-rich regions may be deleterious, and thus more likely to be removed from the population, than those in gene-poor regions. To investigate this hypothesis, we first compared gene density within a 2-Mb window centered around each of the 824 RDs identified in the human genome to gene density within a 2-kb window centered around a set of 10,000 randomly sampled sequences from all chromosomes with a length of 162 bp, i.e., the average length of the RD. There was no statistically significant difference in gene density surrounding RDs (mean = 14.3 genes per 2 Mb) and the random set of sequences (mean = 14.8 per 2 Mb; Student's t-test, *p*>0.05). Moreover, these densities were in good agreement with prior estimates of genome-wide gene density [18]. Therefore, RDs do not seem to accumulate in particularly gene-poor or gene-rich regions of the genome.

When we calculated gene densities per chromosome, rather than genome-wide, as expected, we identified chromosome 19 as the most gene-rich chromosome with 56.9 genes per 2 Mb. However, chromosome X, which showed a deficit of RDs, had a gene density of 15.8 genes per 2 Mb, placing it as the 10th most gene dense chromosome, near the genome-wide average [18]. In sum, these analyses revealed no clear relationship between RDs and gene density and therefore the deficiency of RD on chromosome X remains largely unexplained.

### RDs in Other Vertebrate Genomes

With strong evidence that RDs are common in primate genomes, we used our computational pipeline to screen 6 additional sequenced vertebrate genomes for the presence of RDs, using the same methodology that was used for the primate genomes. These genomes included one bird (chicken), one fish (zebrafish) and four other mammalian species (mouse, rat, dog, and cow). Although the amount and density of TEs in each genome differed significantly among these species (from 8% in chicken to 41% in dog), our pipeline proved effective at uncovering RDs in all genomes surveyed (Table 3).

**Table 3:** Repair-mediated duplications in different vertebrate species.

Species	RD Count	RD Density
Chicken	6	0.08
Chimpanzee	834	0.65
Cow	202	0.20
Dog	192	0.22
Human	824	0.62
Mouse	266	0.26
Orangutan	722	0.55
Rat	179	0.18
Rhesus macaque	650	0.52
Zebrafish	80	0.19

RD density was calculated by dividing the number of RDs by the amount of TE DNA (in Mb) analyzed for each species.

**Figure 7:**
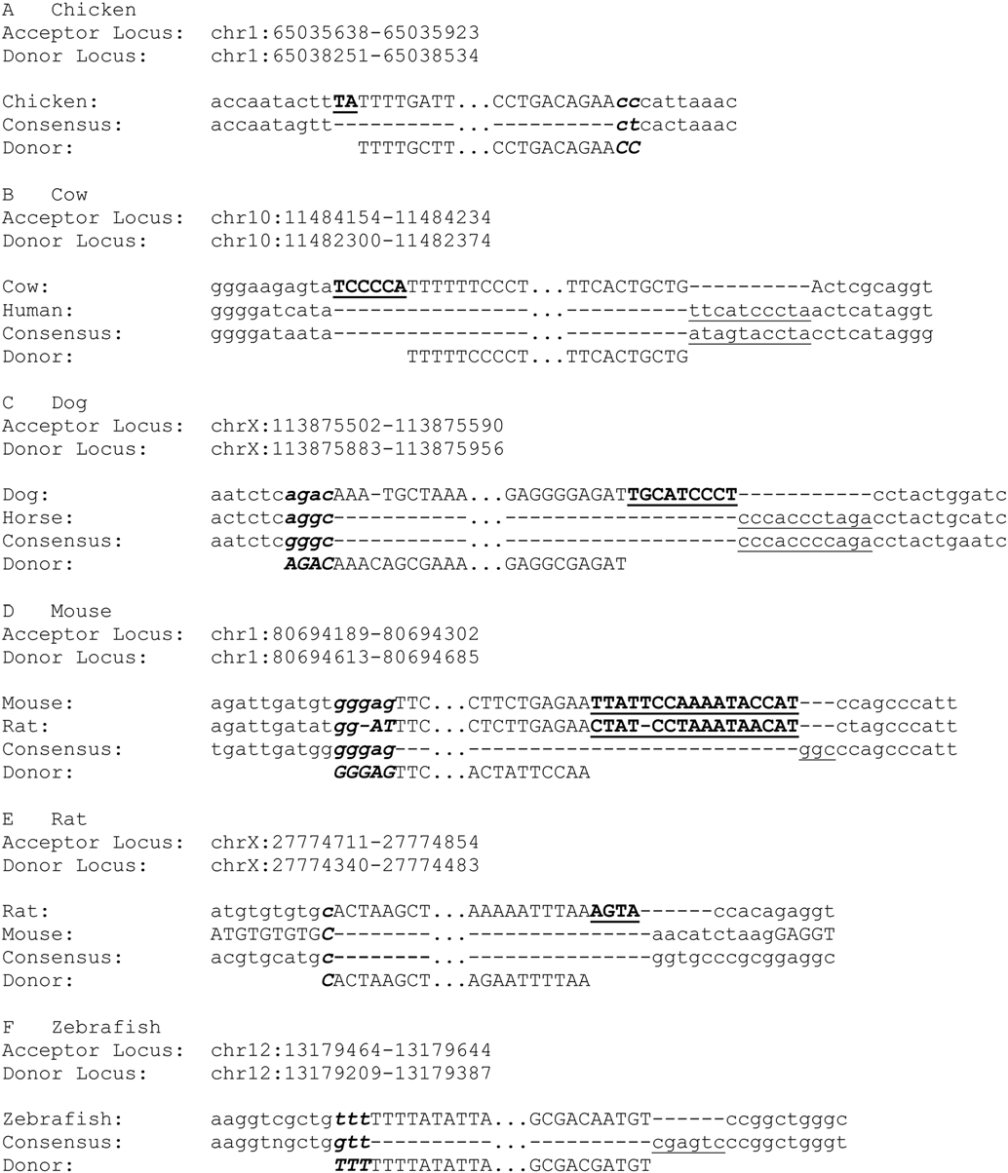
Pre-integration empty sites in non-primate vertebrate species. Microhomologies are in bold and italics, filler is in bold and underlined. Deletions are only underlined.

For those species where there was sufficient genome data from a closely related species, sequence alignments were constructed between the surveyed and related species to precisely examine the breakpoints and validate the RDs (Figure 7). For those species where sequence data from a closely related species was not available, we aligned the TE in which the RD had occurred with the ancestral consensus sequence to identify the pre-integration empty site. Though this method is not as conclusive as cross-species alignment of orthologous loci, it is still effective for predicting the probable breakpoints and identifying the molecular signatures of RD such as deletions, insertions and microhomologies (Figure 7). The analysis of this group of non-primate species shows conclusively that RDs are not only shaping the genomes of primates, but also of other vertebrates.

Comparison of the number of RDs within each surveyed vertebrate species revealed that the number of RDs per megabase of TE sequence (RD density, Table 3) was strikingly higher in all primate species (range = 0.52–0.65) than in other mammals (range = 0.18–0.26) and the difference was even more pronounced between primates and non-mammalian vertebrates (range = .08–0.19). These data suggest that the mechanism of RD and/or the probability of RDs becoming fixed within a population differ substantially between different branches of the vertebrate tree.

## Discussion

### Methodological Advances and Comparison with Other Studies

In this work, we present the first detailed analysis of duplicated DNA segments that bear the hallmarks of repair-mediated capture of other chromosomal sequences across a wide range of vertebrate species. Our investigation focused solely upon RDs that occurred within the portion of the genome derived from TEs. However, we believe that since TEs comprise at least 40% of primate genomes and are found distributed along all chromosomes, our results can be extrapolated to the rest of the genome. Indeed, RDs are, with few exceptions, randomly distributed throughout the human genome (Figure S2) and were found in each of the different TE classes (LINE, SINE, LTR, and DNA) in rough proportion to their relative abundance in the genome (see Table S1).

Computational analysis of low copy number duplications (<10 copies) in large vertebrate genomes has typically focused on segmental duplications (SDs), which are defined as duplicated segments longer than 1 kb with more than 90% identity [26]. It has been shown that SDs account for a sizeable fraction of mammalian genomes (e.g., 5% in human) and represent an abundant source of structural genomic variation within species [27]–[29]. By contrast, the frequency and impact of shorter (<1kb) duplications have not been systematically and thoroughly investigated. One of the challenges of analyzing shorter duplications is the computational time and power involved in identifying and characterizing such duplications, especially in large and complex genomes. Hence, the few studies that have surveyed small-scale duplications have focused on identical or nearly identical duplicons (<100 bp) [30]–[32]. These studies have revealed that most of these short duplications occur in tandem or in close proximity. Interestingly, Thomas et al. [30] discovered an abundant class of short local duplications, called doublets, which share many of the attributes of the RDs identified herein. The authors hypothesized that some of these doublets may have arisen via imperfect DSB repair mechanisms, although this model was not further examined. Our study provides support for this hypothesis and provides additional evidence that a substantial number of short local duplications in the human genome arose via imperfect DSB repair, as predicted initially by Thomas et al. [30].

Our approach distinguishes itself from previous studies by the fact that it provides immediate identification of the template (donor) and duplicated copy (acceptor). Another advantage is that our pipeline significantly reduces both the time and computational resources needed to find such duplications and circumvents the cumbersome and error-prone tasks of parsing large amounts of multi-species alignment data.

One obvious drawback of the method is that it is dependent on the number of TEs and on the annotation of these elements, which vary considerably between genomes and relies on the definition of accurate consensus sequences. However, for genomes such as mammals, which contain large quantities of TEs, and for which high-quality consensus libraries are available, our method provides a powerful alternative to those relying on self- or cross-species alignments. An additional drawback is that the acceptor sequences must be at least 50 bp. One key component of our algorithm is Blast [33], which incorporates the length of the query sequence, in this case, the acceptor, to calculate the statistical significance associated with high-scoring pairs. Therefore, very short query sequences may not produce sufficiently low e-values to accurately identify donor/acceptor pairs.

Our approach is also unique in that it can be tailored to identify duplications that arose by a particular mechanism. Here we focused on duplications that bear the hallmarks of SDSA-mediated DSB repair events characterized experimentally in previous studies. However, one could readily modify our computational pipeline to identify other types of duplications (e.g., retroposition) or imperfect repair events leading to deletions. In fact, our data indicates that the majority of the inter-chromosomal duplications identified during the course of this study are retroposition events that would have escaped detection by methods traditionally employed to identify this type of duplications, i.e., those which use protein-coding sequences as seeds [34].

### Comparison of Repair-Mediated Duplications and Segmental Duplications

Our results suggest that RDs are most likely formed by the SDSA repair pathway. It has been proposed that a mechanism similar to SDSA may be responsible for the formation of segmental duplications in *Drosophila* [35]. Our study provides several lines of evidence indicating that, in mammals and possibly in other vertebrates, SDs and RDs arise by distinct mechanisms.

First, RDs are much shorter than typical SDs. The average size of the acceptor sequences was 162 bp and the largest unequivocal RD we could find was 619 bp. In contrast, SDs are, by definition, larger than 1 kb and often reach dozens of kilobases. While it is conceivable that SDSA could create duplications longer than what our pipeline was able to retrieve, these events would seem to be atypical. Thus, RDs and SDs differ markedly in terms of the size of the duplicated DNA segment.

Second, in 48% of all human SDs, the duplicons are located on different chromosomes [26]. In our analysis, we found that the donor was on a different chromosome than the acceptor in only 254 of the 1,136 potential RDs (22%). In addition, in a sample of 29 inter-chromosomal duplications closely examined, we found that 17 instances (59%) bear the hallmarks of retrotransposition rather than imperfect DSB repair (J.K. Pace and C. Feschotte, unpublished data). Thus, unlike SDs, the overwhelming majority of RDs are intra-chromosomal events.

Third, the genomic distribution of RDs and SDs bear little, if any resemblance. Like RD density, SD density is not uniform among human chromosomes, with some chromosomes showing either an excess (chromosome 19) or deficit (chromosome 8) of duplicons [36]. However, chromosome X, with a deficit in RDs, is neither enriched nor depleted in SDs. While the chromosomal content of SDs can be explained, in part, by a positive correlation between intra-chromosomal SDs and gene densities [36],[37], we found no clear correlation between RDs and local gene densities. The majority of intra-chromosomal SDs form complex clusters aggregated within the interstitial regions of chromosomes (i.e. between pericentromeric and telomeric regions) [26]. We observed no trend for the 824 intra-chromosomal RDs to form clusters or to be located within interstitial regions, but rather to be distributed across the entire length of chromosomes (see Figure S2).

Collectively these data strongly suggest that RDs and SDs represent separate classes of genomic duplications that arise via distinct mechanisms, although both types of duplication must be initiated by DSBs and likely involve repair mechanisms [38]. RDs appear to follow a more uniform genomic distribution than SDs, although some chromosomal biases may be apparent. The most striking characteristic of RDs is their proximal arrangement: the average distance separating RDs is only 1.2 kb and scarcely exceeds 5 kb (Figure 3), while that of intra-chromosomal SDs is 3 Mb [26]. The limited distance between donor and acceptor sequences involved in repair-mediated duplication may reflect the preferred use of ectopic template(s) located on the homologous chromosome (which may explain the deficiency of RDs on the X chromosome) and biophysical constraints during the process of SDSA.

### Genomic Impact of RDs

The 824 RDs identified in this study have duplicated a total of 133 kb of DNA in the human genome. This is a minimal estimate since we could only recover RDs within the portion of the genome occupied by TEs (∼45%). Furthermore, our computational pipeline required the insertion to be present in a single copy elsewhere in the genome, which systematically excluded all instances where part of a TE or another repeat may have been duplicated by the process. Finally, our threshold for retaining high-scoring hits limited us to relatively recent duplication events. Indeed, out of 824 RDs in human, only 53 were found at the orthologous genomic position in a non-primate species (Figure 5). Also excluded from this count are inter-chromosomal events and duplicons located more than 5 kb apart, a fraction of which are likely to represent bonafide RD events (an estimated 41% based on our sampling, i.e., ∼124 events). Thus, the process of RD accounts for hundreds of small-scale duplication events in the human genome.

We found that RD can affect virtually any sequence in the human genome, including exons, untranslated regions (UTRs), predicted transcription factor binding sites (TFBS) and most conserved (PhastCons) elements (Table 2). Each of the functional elements duplicated has the potential to be re-used at the acceptor site. In addition, the duplication of TFBS can lead to changes in chromatin structure at the acceptor site and generate new transcripts by duplication of promoter sequences and splice sites. Thus, like other forms of genomic duplication, RDs have the potential to profoundly alter genome architecture. RDs offer the added originality of creating local duplications, a characteristic that might promote the functionalization of the newly duplicated segment. For example, duplicated exons are likely to be inserted, together with their flanking splice sites, in adjacent intron sequences (see the example of RD within *ACSBG2*), which may facilitate their incorporation into a splice variant producing a new protein isoform.

The local duplication of TFBS may be particularly relevant to regulatory evolution because they are known to occur frequently, and function cooperatively, as closely spaced pairs [39],[40]. RDs may also contribute to the rapid positional turnover observed for TFBS, which is thought to occur in part via local duplication [41]–[43]. Finally, local duplications may promote further genomic rearrangements, such as deletion or inversion of the intervening sequence mediated by ectopic recombination between the duplicated segments. Thus, RDs present a number of characteristics that provide them with a strong potential for genomic restructuring.

### Interspecific Variations in the Amount and Rate of Repair-Mediated Duplication

We found substantial variations (up to eightfold) among different vertebrate species in RD density (see Table 3). In particular, we found two- to threefold higher RD density in primates than in non-primate mammals (mouse, rat, dog, and cow), even though genome size, TE content and TE composition are similar in all these species [44],[45]. The faster rate of DNA substitution and deletion in the rodent lineage could, in principle, account for some of the discrepancy, as it would hinder our ability to detect relatively ancient RDs in these genomes and effectively limit the evolutionary depth of our analysis as compared to the primate lineage. However, this phenomenon cannot account for the difference in the number of RDs observed in the human genome (n = 824) and those in dog (n = 192), in which the dog substitution rate is only 1.18 times higher than that of human [45].

Another explanation could be that these discrepancies reflect intrinsic differences in the fidelity or usage of repair mechanisms in the germline of these mammals. Previous studies have shown that several proteins involved in DSB repair are under positive selection in yeast, potentially due to pressures exerted by retrotransposon activity [46]. Additionally, the repair protein Cernunnos-XLF has been shown to be under positive selection within the human lineage [47]. These observations suggest that DSB repair proteins in different species lineages might be subject to different selective pressures imposed by lineage-specific waves of TE amplification, retroviral invasions or other evolutionary forces. Thus, it is possible that the variation in the number of RDs observed in different mammalian lineages mirrors some intrinsic divergence in their repair machinery.

Still another, not mutually exclusive alternative could be that species variations in RD density reflect different levels of genome instability experienced in each lineage. In other words, one would expect that genomes that have been subject to higher levels of DSBs in the past would display more instances of RD. Indeed, several lines of evidence suggest that primate genomes have been subject to particularly intense genomic instability.

First, the human and chimpanzee genomes have undergone a profusion of lineage-specific segmental duplications in the recent past, with as many as 33% of the human SDs having occurred solely in the human lineage [48]. Furthermore, SDs are more abundant in primate genomes than in the genomes of mouse, rat and dog, especially interspersed (non-tandem) duplications [26],[49],[50], that in turn likely triggered further genomic instability [26].

Secondly, primate genomes have endured large bursts of transposition over relatively short periods of time, especially during the first half of the primate radiation, leading to high copy number TE families specific to the primate lineage [18], [44], [51]–[53]. Such transposition bursts are likely to have been accompanied by a profusion of double-strand breaks caused either directly by the endonuclease activity of transposition enzymes or indirectly by recombination events between dispersed TE copies. Although it is clear that many TEs have been concomitantly active in other mammalian lineages, it appears that carnivores at least [45], and potentially also artiodactyls, have experienced less explosive TE invasions and may have experienced fewer TE-induced DSBs.

The latter scenario could also account for the observed slowdown in the rate of repair-mediated duplication in hominoids compared to their anthropoid ancestors (Old and New World monkeys, see Figure 5). Notably, the periods of highest repair-mediated duplication in primates coincide with the periods of most intense activity of L1, as reflected by the copy numbers of L1, Alu and processed pseudogenes inserted at that time, which all rely on the machinery of the autonomous L1 retrotransposon for amplification [18],[51],[54]. Given that the L1 machinery is also a potent source of DSBs [55], the tremendous activity of L1 during these periods would have caused significant genomic instability, creating hundreds of thousands of opportunities for DSBs to be repaired via the SDSA pathway, thereby creating RDs. In the era between 30 and 42 Mya, when we found the most elevated rate of RD (18.3 per Myr), L1 generated ∼23,000 copies of itself (L1PA7, L1PA8, L1PA8A) [52] and ∼342,000 copies of *AluSx* elements [56] (see http://genome.ucsc.edu for counts). In the subsequent period (30-18 Mya), when the rate of RD decreased to 11.8/Myr, ∼29,000 L1 (L1PA4, L1PA5, L1PA6) [52] elements and ∼139,000 *AluY* [56] elements were added to the primate lineage. In contrast, only ∼17,000 L1 and ∼10,000 *Alu* elements have been added to the human genome in the past 18 myr, where we observe the lowest rate of repair-mediated duplication (∼3/myr). Thus, there is an excellent correlation between the level of activity of L1 and the rate of RD during primate evolution.

A second line of evidence for varying levels of genomic instability during primate evolution lies in the analysis of nuclear mitochondrial insertions, or NUMTs, and of SDs, both of which are likely initiated by DSB [38],[57]. Two independent studies concluded that a significant burst of NUMT integration in the primate lineage occurred between the split of New and Old World monkeys (30–42 Mya), followed by a slowdown of NUMT accumulation [58],[59]. In addition, a significant burst of inter-chromosomal segmental duplication was observed during or shortly after the divergence of Old World monkeys from the hominoid lineage (25–30 mya) [60]. These bursts of NUMT and SD coincide with the highest rate of RD formation in the primate lineage (see Figure 5). While some of these bursts might also be explained by population bottlenecks promoting the fixation of these rearrangements or by differences in generation time between species, these observations collectively suggest that a high level of genomic instability and structural variation occurred in the primate lineage between 18 and 42 Mya.

### Conclusion

Our findings that RDs occur not only in primate genomes, but also in other vertebrate genomes, indicates that this mechanism has been shaping genomes for potentially hundreds of millions of years. Since RDs are most likely created by SDSA, a form of homologous recombination, it is not surprising that RDs have occurred in a wide range of vertebrates. Although there has been a significant decrease in the formation (or fixation) of RDs in the human lineage, the process has nevertheless generated numerous lineage-specific duplications during hominid evolution and produced structural genomic variation among humans. Recent genome-wide analyses have revealed that non-TE insertions ranging in size from a few dozens to a few hundred nucleotides are among the most common structural variants among humans [61],[62]. Based on our data, we can anticipate that some of these structural variants result from imperfect DSB repair processes akin to SDSA.

## Materials and Methods

### Retrieval of Genome Sequences and RepeatMasker rmsk Files

Genome sequences and RepeatMasker rmsk files were downloaded from the UCSC Genome Browser (http://genome.ucsc.edu). The versions used were: human (hg18), chimpanzee (panTro2), Rhesus macaque (rheMac2), mouse (mm8), rat (rn4), dog (canFam2), cow (bosTau2), chicken (galGal3) and zebrafish (danRer4).

### Identification of RDs

Potential RDs were identified by a Perl script that searched the RepeatMasker rmsk files for TEs that had been interrupted by some intervening sequence. A TE was classified as interrupted if the repeat name and orientation of the first segment (TE-A) matched the repeat name and orientation of the second segment (TE-B), TE-A and TE-B were separated by at least 50 bp and neither TE-A nor TE-B was longer than 95% of the length of the consensus sequence. In addition, the ending consensus sequence position of TE-A was within +/− 30 bp of the starting consensus sequence position of TE-B. After the potential RDs were identified, false positives were removed. TEs separated due to nested TE insertions were removed with a Perl script and annotated retrogenes, segmental duplications or L1-mediated 3′ transduction events were manually inspected and removed from the dataset.

For all remaining potential RDs, a Perl script retrieved the acceptor sequence, along with 100 bp flanking each side, and used this sequence as a Blastn query against the entire genome. In order for a Blast hit to be considered, it had to match at least 80% of the length of the query sequence with at least 50% identity. The minimum cutoff score was calculated separately for each acceptor sequence using a sliding e-value that was unique for each query sequence. The Blast output was then parsed. If the acceptor sequence minus the flanking regions was found in more than two HSPs (high-scoring pairs), the RD was removed from the dataset since the donor could not be unequivocally determined. Additionally, if the acceptor sequence with the flanking regions was found more than once, the RD was discarded to avoid including potential segmental duplications or transposition of chimeric TEs. Finally, if the donor was within 50 bp of the acceptor sequence, the RD was also removed from the dataset.

If all of the above criteria were successfully met, the putative donor sequence was used as a Blastn query sequence against the entire genome. This “reciprocal” Blast query then used the same criteria to determine if any sequence matched the donor. In order for the acceptor and donor sequences to be classified as a putative RD, the acceptor sequence had to be the second hit in the Blastn output generated when the donor was the query sequence and the acceptor had to meet all criteria.

### PCR Verification of Computationally Detected RD Loci

To verify that the computationally detected RDs existed in vivo and did not represent genome assembly errors, we designed oligonucleotide primers flanking each locus using the Primer3 web interface (http://frodo.wi.mit.edu/). PCR amplification was performed in 25-ul reactions with 10–50 ng genomic DNA, 200 nM of each oligonucleotide primer, 200 mM dNTPs in 50 mM KCl, 1.5 mM MgCl2, 10 mM Tris-HCl (pH 8.4), and 2.5 units Taq DNA polymerase on an Applied Biosystems GeneAmp PCR System 9700 thermocycler. Amplification cycles were as follows: an initial denaturation step of 94°C for 4 min; followed by 32 cycles of 1 min of denaturation at 94°C, 1 min of annealing at optimal annealing temperature, and 1 min of extension at 72°C; followed by a final extension step at 72°C for 10 min. For loci with large duplications (>2 kb), we used Ex Taq polymerase (TaKaRa) and carried out PCR in 50 ul reactions following the manufacturer's suggested protocol. PCR amplicons were separated on 2% agarose gels, stained with ethidium bromide, and visualized using UV fluorescence.

To identify lineage-specific human and chimpanzee duplication loci, PCR amplification was performed on a panel of genomic DNA from five primate species, including *Homo sapiens* (HeLa; cell line ATCC CCL-2), *Pan troglodytes* (common chimpanzee; cell line AG06939B), *Pan paniscus* (bonobo or pygmy chimpanzee; cell line AG05253B), *Gorilla gorilla* (western lowland gorilla; cell line AG05251), and *Pongo pygmaeus* (orangutan; cell line ATCC CR6301). To evaluate polymorphism rates of human lineage-specific duplications, we amplified loci on a panel of genomic DNA from 80 diverse human individuals (20 from each of four populations: African-American, South American, European, and Asian) that was available from previous studies in the Batzer lab at Louisiana State University (Table S2).

### Calculation of Expected Number of Repair-Mediated Duplications per Chromosome

In order to calculate the expected number of RDs per chromosome based upon the percentage of total genomic DNA accounted for by each chromosome, we used a variation on a previously published Monte Carlo simulation [63]. We used a series of PERL scripts to divide the human genome (version hg18) into 10,000 equal size bins (308,042 bp/bin) and calculate the number of RDs per bin, based upon the number of RDs we had previously discovered. A final PERL script performed the actual Monte Carlo simulation. This script loaded all 10,000 bins, along with the number of RDs in each bin, into an array and the rows were randomized. The first *n* rows of the array, where *n* is the number of bins on a given chromosome based on the chromosomes length, were examined and the total number of RDs in these bins was calculated. For example, since chromosome 1 had 803 bins based upon its length, the first 803 rows of the array were be used to calculate the expected number of RDs on that chromosome. This process was repeated 10,000 times for each chromosome.

A similar process was used to calculate the expected number of RDs per chromosome based upon the percentage of the total amount of transposable element DNA on each chromosome. However, in this simulation, the number of bins used to calculate the expected value per chromosome was determined not by the percentage of genomic DNA occupied by the chromosome, but rather by the total amount of transposable element DNA located on the chromosome. For example, 7.93% of the total human transposable element DNA is located on chromosome 1. Therefore, we used 793 of the 10,000 bins to derive an expected number in each replicate rather than the 803 bins used above.

P-values for each chromosome were calculated using the output of the Monte Carlo simulations. For each chromosome, we calculated the number of replicates where the number of RDs was greater than or equal to the number of RDs we discovered via our computational pipeline. This number was then divided by 10,000 (the total number of replicates per simulation) to derive the p-value.

## Supporting Information

Figure S1 Pre-integration empty sites in primate species. Microhomologies are in bold and italics, filler is in bold and underlined. Deletions are only underlined. Target site duplications are highlighted in green.
(0.18 MB PDF)

Figure S2 Chromosomal distribution of RDs in human. The histogram to the right of each chromosome indicates the number of RDs within the region.
(0.06 MB PDF)

Table S1 All RDs identified in the human genome.
(0.45 MB XLS)

Table S2 Loading order of gels. The numbers for each lane are designators for the 80 human subjects whose DNA forms the Coriell population panels used.
(0.01 MB PDF)
