# Supplementary material for: Repair-Mediated Duplication by Capture of Proximal Chromosomal DNA Has Shaped Vertebrate Genome Evolution
Source: PLoS Genet. 2009 May 8;5(5):e1000469. doi: 10.1371/journal.pgen.1000469 (PMC2671141; doi:10.1371/journal.pgen.1000469)
Supplement: Figure S1 — Pre-integration empty sites in primate species. Microhomologies are in bold and italics, filler is in bold and underlined. Deletions are only underlined. Target site duplications are highlighted in green. (0.18 MB PDF) [file pgen.1000469.s001.pdf]

Human-specific RDs - donor and acceptor within 5 kb of each other

chr6:14395452-14395652

Alignment

Human, chimp, rhesus, MER21C consensus, donor

```
TGGACAATACCAGTGTAAGTAATTTAATCCAAGCCCTCTGCATTCTTGCAACCTTTGTTTTAGCCACAAGGTGATTCTATTTGGTGATT
Tggacaataccagtgtaagtaatttaatccaagctctctgcattcttgcaacctttgttttagccacaaggtgattctatttggtgatt
Tggataataccagtgtaagtaatttaatccaagccctctgcattct-gcaaccttcgtttcagccacaaggtgattccatttggtggct

GCATTCCAACAGcaattctctgacaccaactgggtgtcctacaattcaattcaatacattttgacactaactatccctatttaacatca
gcattccaacagcaattctctgacaccaactgggtgtcctacaattcaattcaatacatttggacactaactacccctatgtaacatca
gcatttcaacagcaattct--gacaccaactgggtgtcctacaattcaattcaatacattttgacattaactaccccgatgtaacatca

Gaacccacaaggtaaagggcaaggtccctaataatactgcccttactcacataccagctacaaacagggtccccaggctaccctcactt
Gaacccacaaggtaaagggcaaggtccctaataatactgcccttactcacataccagctacaaacagggtccccaggc-accctcactt
gaacccacaagttaaagggcaggatccctaacaatactgcccttactcacattctagctacaaagagggtccccaggctaccctcactt

Cagtct-gacttggcaacaaatttaggggttcccacaa-ctccctctggctcaagaattcaatagaatggcttaggaaactcaagtaagca
Cagtct-gacttggcaacaaatttaggggttcccacaa-ctccctctggctcaagaattcaatagaatggcttaggaaactcaagtaagca
Cagtct-gacttggcaacaaatttggggggttcccacaa-ctccctctggcttaagaattcaatagaatggcttatgaaactcaagtaagca

Ctatacttactgttaccattttattataaaggatacagatgaatagcaagagaaagagatacacagggcaaggccttgggggtagaggt
ctatacttactgttaccattttattataaaggatacagatgaatagcaagagaaagagatacacagggcaaggccttgggggtagaggt
ctatgcttactgttacccttttattataaaggatacagatgaatagcaagagaaagagatacacagggcaaggccttgggggtagaggc

ggggtgcagagcttccatgGACTCCTTATGGAAGCCTTCCAAGCCTCTGGTGGACAGGAAGCTGATCTCTCAGGTCCAGTATGTGTCAG
ggg-tgcagagcttccatg----------------------------------------------------------------------
ggggtgcagagcttccatg----------------------------------------------------------------------
ggggtgcagagcttccatg----------------------------------------------------------------------
                                      CAAGCCTCTGGTGGACAGGAAGCTGATCTCTCAGGTCCAGTATGTGTCAG

TCCAAAGGCAGTTGAAGATAGGTAAGATTTGATAAAACAGCACAACAAGACAATGAGAATATATTGTGGACAAACTGTGTGCCTAGGAT
-----------------------------------------------------------------------------------------
-----------------------------------------------------------------------------------------
-----------------------------------------------------------------------------------------
TCCAAAGGCAGTTGAAGATAGGTAAGATTTGATAAAACAGCACAACAAGACAATGAGAATATATTGTGGACAAACTGTGTGCCTAGGAT

CCTAGGGATTAGAAAAGATGTGGAAAACATCCAACAACTTAATtccaggtatgttgccttcccagcacatggatgcgttcat
-------------------------------------------tccaggtatgttgccttcccagcacatggatgtgttcat
-------------------------------------------Tccaggtgtgttgccttcccagcacat-gatgtgttcac
-------------------------------------------Tccaggtatgttgccttccca
CCTAGGGATTAGAAAAGATGTGGAAAACATCCAACAACTTAATTCCA

aacccagGaacttcttggagcctctctgtttcagcgttttattaaggttccattatgcagacatgatttatttaaataccggtcttggt
aacccaggaacttcttggagcttccctgtttcagtgttttattaaggtttcattatgtagacatgatttatttaaataccggtcttggt
aaaccaggaaattctcggagcctccctgtttcagcattttattaaggtttcattatatacacatgattgatgtaaatacgggtcttggt

gactgaacccaacctctctagtccttctaccttccccagaggttcagagtggggctgaaa-ttctaactctttaatcatatggttggttt
gactgaacccaacctct--agtccttctaccttccccagaggttcagagtggggctgaaa-ttctaactcttcaatcatgtggttggttt
gactgaacccaaactct--agtccttctaccttccccagaggttcagagtggggttgaaa-ttctaactcttcagtcatgtggttggttt
```

```
chr13:106474058-106474295
Alignment
Human, chimp, rhesus, LTR9B consensus, donor
CTGCTTGACTATGCCCCATCTCCCTTTCTCCCCTCATCTTCTCATCTAACCTATGGATTATATGCATCACCAAAGCTGGTCTATTGGCT
ctgcttgactatgccccatctccctttctcccctcatcttctcatctaacctatggattatatgcatcaccaaagctggtctattggct

GACCCTGCTTCtgttataggagttattaagaaattattttaggcagataaagaggtaaaggggtccttgggaagtttttgtttatttt
gaccctgcttccattataggagttattaagaaattattttaggcagataaagaggtaaaggggtccttgggaagtttttgtttatttt
           TGTTGTAGGAGTTATTAAGAAATTATTTTAGGCAGATAGAGAGGAAAAGGGGTCCTTGGGAAGTTTTCGTTTCTTTT

aAagcagctccaggaacatttcttgtctaccaggaaagcccca-gctcttgctgggctggaaagctccagaaacatttcttgtctaccagg
aAagcagctccaggaacatttcttgtctaccaggaaagcccca-gctcttgctgggctggaaagctccagaaacatttcttgtctaccagg
                                                              agctccagaaacatttcttgtctaccagg
AAAGCAGCTCCAGAAACATTTCTTGTCTAGCAGAAAAGCCCCGGCTCTT------------------------------------------

Aaagccccagctgttgctgggctggaaagctttgatatgcaaatgcaggccattagaaactgggtccacccaaacatggc---tcccttcat
Aaagccccagctgttgctgggctggaaagctttgatatgcaaatgcaggccattagaaactgggtccacccaaacatggc---tcccttcat
Aaagcccctgctcttgctgggccagaaagttttcatatgcaaatgcaggccattagaaactgggtccacccaaacatggc---tcccctcgt
AGAGCC------------AGGCCGGCAANCTTTGATATGCAAATGCAGGCCATTAGAAACTGGGTCCACCCAA-CATGGCGATTCCCACCGT

Cctcctcttgcccttgcccccacaggtgcctggcaacatggctgcccccacatatccctatgtgtgtagaacatcatggcgccctgcat
ccacctcttgcccttgcccccacaggtgcctggcaacatggctgcccccacatatccctatgtgtgtagaacatcatggcgccctgcat
cctcttcttgcctgtccc--cacatgtgcctggcaacatggctgcccccacatatccccatgtgtgtagaatatcatggcgccctgtat
CGTCTTCTTGCCCTTGCCCC-ACATGTGCCTGGCAACATGGCCGCCCCCACATATCCCCACGTGTGTAGAACATCATGGCGCCCTGCAT

Ttgcatattaaaaggatagggtgggagggccaggtttttccactggctacatgag-tgacatgcctggtcaaaccaatcccctgagccct
Ttgcatattaaaaggatagggtgggagggccaggtttttccactggctacatgaa-tgacatgcctggtcaaaccaatcccctgagccct
ttgcatattaaaaggctagggtgggagggccaggtttttcca-tgggctacatgaatgacgtgcttggtcaaaccaatcccctgagcctt
TTGCATATTAAAAGGCTAGGGTGGGAGGGCCAG-TTTTTTCGCGGGCTACGTGAA-TGACATGCCTGGTCAAACCAATCCCCTGAGCCCT

atgcaaatcagacaccacctcctccagcttcatcgtataaGCAGCCACTTATTATTTGTCAACAACATGCGAAAGGCTCTTTTCTTTAT
atgcaaatcagacaccacctcctccagcttcatcgtataagcagccac-----------------------------------------
atgcaaatcagacaccacctcctcctgcttcctcatataagcagccac-----------------------------------------
ATGCAAATCAGACACCGCCTCCTCCAGCCTCCTCATATAACTGGCTGN-----------------------------------------
                                                TTATTATTTGTCAACAACATGCGAAAGGCTCTTTTCTTTAT

GACCCTGTGGGAGAGTTCAGAGGATCAGAGTATGGATATGTGGCCACAAGCATAAAAATCTAGTTCCCTGGATTGACCTTGAGCAATTA
-----------------------------------------------------------------------------------------
-----------------------------------------------------------------------------------------
-----------------------------------------------------------------------------------------
GACCCTGTGGGAGAGTTCAGAGGATCGGAGTATGGATATGTGGCCACAAGCATAAAAATCTAGTTCCCTGGATTGACCTTGAGCAATTA

GTCTAATATCATTTCTTTACATGACCTTAATGATTATTCAGTTACAAAATAATCTTTCCTTTGCTTAAGTTTGTAAGTTTGTAACTTTG
-----------------------------------------------------------------------------------------
-----------------------------------------------------------------------------------------
-----------------------------------------------------------------------------------------
                                                                         ATTGTTTGTAACTTTG
GTCTAATATCATTTCTTTACATGACCTTAATGATTATTCAGTTACAAAATAATCTTTCCTTTGCTTAAGTTTGTAA--------------

CTTAAGTTTGTAtttcctctcgcact-agggttttctcttt--gttt--gagtcacccctccctctgtctctatacaggggagctgttttcttc
------------tttcctctcgcact-agggttttctcttt--gttt--gagtcacccctccctctttctctatacaggggagctgttttcttc
------------tttgctcccgcact-aggattttctcttt--gttt--gaatccccccctccctctttctata--caggggagctgttttcttc
------------TWTCCGC-CGCACNCGGGGTTTCCTCTCTCGGCTTTGGAGCC-CCCCTCCCTCTGTCTCTGTACAGGGGAGCTTCTTCCTTC
CTTAAGTT

Ttccttg---ctgctctcttgcctgttaaacttgtcgctccgctccttaaaacccctccacatgtgtccatgt----taatccta-tcagtgcaag-
Ttccttg---ctgctctcttgcctgttaaacttgtcgctcc-----ttaaaacccctccacatgtgtccatgt----taatccta-tcagtgcaag-
Ttccttc---ctcctctcctgtccgttaaacttgtcgctcc-----ttaaaaccactccacctgtgtctgtgt-tgttaatccta-tcagtgcaag-
TTTCTTCTCCCTTCTTTCTTGCCTATTAAACT-----CTCCGCTCCTTAAAACCACTCCACGTGTGTCCGTGTCGTTTTATCTAAATCGGCGCGAGG

accaagaaccctggtgttcctccagccatcggagccgtaacaCTTCTACTTTTTCCTTATTTTCTATTAATTACCCTAAAATATGCATGA
accaagaaccctggtgttcctccagccatcggagccgtaacacttctactttttccttattttctattaattaccctaaaatatgcacga
accaagaaccctggtgttcctccaaccatcggagccgtatcacttctacttgtgccttactttctattaattaccctaaaatatgcatga
ACCAAGGACCCTGGTGTTCCTCCASTCATCGGAGCCGTATCA
```

AAGAGAGCAGAGATCGTTAAACATGCCAATTCCTTTCCCTAATCTCAAGTAA
Aagagagcagagatcgttaaacatgccaattcctttccctaatctcaagtaa
aagagagcagagatcattaaacatgccaactcctttccttcatctcaagtaa

Chr18:26242863-26243040
Alignment
Human, Chimp, Rhesus, LTR76 consensus, donor
Tggcctggcactctcaagtaggtctccatgtagctggaagctgctctggcaatcagagttaggccttggtgtcttgttgaatataaaaa
tggcctggcactctcaagtaggtctccatgtagctggaagctgctctggcaatcagagttaggccttggtgtcttgttgaatataaaaa
tggccttgtactctcaagtaggtctccatgtagctggaaactgctctggcaatcagagctaggccttggtgtcttgttgaatgtaaaaa

Atcccacataaccccactacctagaaaggtcactcttggatgattatgataaggtgggaaattaaagaaagaaagaaaaaatttttaaa
atcccacataaccccactacctggaaaggtcactcttggatgattatgatgaggtgggaaattaaagaaagaaagaaaaaaattttttta
tatcccacaaacccc-ctacctggaaaggtcactct--gacgattataatgaggtgggaaattaaagaaagaaagaaaaaaaatttttta

Aaaaa-gacagaaataagctttcctgtattaacctgactttttccagagacagcaacaggcacagcccagacccagggaaagtcttgat
aaaaaagagagaaataagctttcctgtattaggctgactttttccagagacagcaacaggcacagcccagacccagggaaagtcttgat
aaaaaaggt-gaaataagctttcctgtattaggctgacttgtcccagagatagcaacaggcacagcctagacccaggaaaagtcttgat
taagctttcctgtattaggctgacttgtcccagaggcagcaacaggcacagcccagacccaggaaaagtcttgat

aA-actatctctgatatgccctgaagactctcccagcacttcctaaacatagggaggagaaaaactaa-ttttcctctctcttatggtatg
aa-actatctctgatatgccctgaagactctcccagcactccctaaacatagggaggagaaaaactaa-ttttcctttctcttatggtatg
aatactatct--gatttgccctgaagactctcccagcactccactaacacagggtggagaaaaactaa-ttttactttctcttatgacatg
aatattatct--aatgtgctctggagactctcccagcactccctcaacatagggagaagaaaaaacaaattttcctttgttttatggtatg

aGgttagagattcatgttctctctaactagtaacttcatgtattctgttttatctaagcagtggagtgaaggtcataagccatctgagc
aggttagagattcatgttctctctaactagtaacttcatgtattctgttttatctaagcagtggagtgaaggtcataagccatctgagc
agtttagagattcctattctctgtaactagtaacttcaagtattgtattttatgtaagcagtggagtgaaggtcataagccatctgagc
agtttatagattcctgttctctgtaactagtaacttcaagtattctgttttatctaagcagtacagtgaaggtcatgagaagcctgagc

aggcctgaactacagccacctgggggccatagtgaaggtcatgg**CC**TTCAAATTACA--GTTACCTAAGGCTTCCAAGCTTTCATTGAACT
aggcctgagctacagccacctgggggccatagtgaaggtcatgg--
atgcctgagctacagccacctgggcgccatagggaaggtcatgg--
aggcctgaactacagccacctgggcaccatagtgaaggttatgr--
TTCAAATTACACAGTTACCTAAGGCTTCCAAGCTTTCATTGAACT

AATCTCTGTTTTACCTGCTACTATTTTCAATATTTTTAATCGTTATGCCTAGTTATTCTTCAAATCTTGAAGACATTAAGATGATATCT

AATCTCTGTTTTACCTGCTACTATTTTCAATATTTTTAATCGTTATGCCTAGTTATTCTTCAAATCTTGAAGACATTAAGATGATATCT

TGTTGGGATGAATGATCTTTAAGTTTTCTGTGCAGCTTTTCGGAC-***ataa***gc-tgtgcta--ggcactagggcaaacctagataacagccat
---a***ataa***gc-catgcta--ggcactagggcaaacctagataacagccat
---a***ataa***gc-catgctg--ggcactaaggcaaacgtagataatggtcat
---G***ataa***gcccgtgcaaaaggctctagagcaaacctagataacagacat
TGTTGGCATGAATGATCTTTAAGTTTTCTGTGCAGCTTTTCGGAC-***ATAA***

Ctgggctacatagcaacggtcatgtgtaatcctgagttatgcacctgtcacatttgattaactgtctttgttctgcctttgtatcc-tc
Ctgggctacatagcaacggtcatgtgtaatcctgagttatgcacctgtcacaatttgattaactgtctttgttctgcctttgtatcc-tc
Ctgggctacatagcaatggtcatgtgtaatcctgagttatgcacttgtcacaatttgattaactgtctttcttctgcctttgtatcc-tc
Ctgggttgcatagcaatggtcatgtgtaatcctgagttatgaacctgtcacaatttgattaactgtctttgttctgcctctgtatccct-

Actttcgtgccactgtaaacttgtttcaggctagcccacccccttttt-tgaagtgtgtataaaagtcaagtgctgtctttgttctgggcc
actttcatgccactgtaaacttgtttcaggctagcccaccccgtttt-tgaagtgtgtataaaaatcaagtgctgtctttgttctgggcc
actttcgtgccactgtaaactttttcaggctagcccactcccttttt-tgaagtgtgtataaaagtcaagtgctgtctttgtcctgggcc
gctttcatgccactgtaagcttgcttcaagctagcccaccccccttttgtgaagtgtgtataaaagtcaagtgctgtctttgttctgggcc

cagtctttggatgttaagtgtgctgggtctgagtgcactcaataaagctcctcctatatacaccccaaggtctctctggccctcctgat
cagtctttggatgttaagtgtgctgggtctgagtgcactcaataaagttcctactatatacaccccaaggtctctctggtcctcctgat
cagtctttgtatgttaagtgtgctggatctgagttcactcaataaagatcctcctgtatacaccccaaggtctctctggtcctcctgat
cagtctttggatgttaagtctgctgggtctgagtgcactcaataaaagatcctcctgtttcaccccgaggtctctctcgtcctcctgat

tcccacaacaATGACGGTAGATGAAAACAAAACTACTTCCTAATTTTGTcaaaacataaacaaaactgaagtcaccacataaagtgcac
tcccgcaacaatgacggtagataaaaacaaaactacttcctaatttgtcaaaacataaacaaaactgaagtcaccatataaagtgcac
tcccacaacaatgatagtagatgaaaacaaaactacttcccaatcttgccaaaacataaacaaaactgaagtcaccatatgaagtgcac
tccygcaaca

aaacccc-ttattctgactaacatgagtctc--acctttgccaatcacaaccattagtcttactctattcttttgccttttagacaagaat
aaacccc-ttattctgactaacatgagtctc--acctttgccaatcacaaccattagtcttactctattcttttgccttttagacaagaat

aaaccccccttattctgactaacatgagtgtctcacctttgccaatcacaaccattagtgttactctattctttttgccttctagacaagaat

```
Chr2:235427735-235428072
Alignment
Human, Chimp, Rhesus, MER46C consensus, donor
ATTCCTTACTTTACAACAAAGTATTCCTTACTTTAGATGAGTATATTATTAAAGATTTATTATGggtccacagctccttatctgcaatg
Attccttactttacaacaaagtattccttactttagatgagtatattattaaagatttattatgggtccacagctccttatctgcaatg
                     tattccttcctttagatgagt-tattattaaagatgtattatgggcccacaactccttatgtgcaatg
                                                                ggtccacaatcccttatccgcaatt

Gcaaaatccaaatgctctggaaaacaaaaagtgttcttttctacatttgacgtaaatttgtttgtatgcaaatctgaccttaactaaca
gcaaaatccaaatgctctggaaaacaaaaagtgttcttttctacatttgacgtaaatttgtttgtatgcaaatctgaccttaactgaca
gcaaaattcaaatactctggaaaaaaa---gtgttcttttctacatttgacataaatttgtttgtatgtaaatctgaccttaactgaca
ccgaaatccaaaaagctctgaaaaccgaaagtttttcataagtttggcacgaactcatttggcggca-aaacctgacctgaactgata


tgaggttattcaaagtctatatttatcctgcatggtgtgaatattcataggtttcactgtagagatattaatgtatttaattaATATCC
tgaggttattcaaagtctatatttatcctgcatggtgtgaatattcatacgtttcactgtagagatattaatgtatttaatta------
tgaggttattcatagtctgtatttatcctgcatggtgtgaatattcataagtttcactgtagaaatattaatgtatttaatta------
tgaggctatttatagtctttatttatcccacttagtgtgaatattcatatatttcgctgcagaaatattaatgtgtttgatta------
                                                                                   ATATCC

CATGGTGAAGCCACCATCACCCCAATCAGGGCTGTCTTCATGATAACTTCTGAGTGACTGGGACCATTTCTCTACCCTGAAGGCCCCTT
-----------------------------------------------------------------------------------------
-----------------------------------------------------------------------------------------
-----------------------------------------------------------------------------------------
CATGGTGAAGCCACCATCACCCCAATCAGGGCTGTCTTCATGATAACTTCTGAGTGACTGTGACCATTTCTCTACCCTGAAGGCCCCTT


CAGCTCCTCAAACTCAATGCATCTAATCCAAGAGTCTTTTACCCTGAAACCAGCTCCCCTTAGGGGTCTGACAACAGGACCTTCCTTCT
-----------------------------------------------------------------------------------------
-----------------------------------------------------------------------------------------
-----------------------------------------------------------------------------------------
CAGCTCCTCAAACTCAATGCATCTAATCCAAGAGTCTTTTACCCTGAAACCACCTCCCCTTAGGGGTCTGACAACAGGACCTTCCCTCT

CACTGGCATGAGACCTCAGGGCCCATCTGCCCTTCCTCCCTCTCTCACCAAGCCCAGAGTCCCTGTGATGCCTCACTGCCTTGGCTCAC
-----------------------------------------------------------------------------------------
-----------------------------------------------------------------------------------------
-----------------------------------------------------------------------------------------
CACTGGCATGAGACCTCAGGGCCCATCTGCCCTTCCTTCCTCTCTCACCAAGCCCAGAGTCCCTGTGATGCCTCACTGCCTTGGCTCAC

CCTTCTTTCCCATGCCCACCACCACAACCACATGCCTCTCCTCACACCCTGATTCCTCAC-GGTCAG---------gcctcaga
-------------------------------------------------------------------caggctgtggcctcaga
-------------------------------------------------------------------caggctgtggcctcaga
-------------------------------------------------------------------Cggggtgctgccccaga
CCTTCTTTCCCATGCCCACGACCACAACCACATGCCTCTCCTCACACCCTGATTCCTCACAGGTCT----------GCCT

cctcactagggagttTtacataacatatgttttgtgcagtgtattgctttttaaaatctcaaaaattctgaaatct
cctcactagggagttttacataacatatgttgtgtgcagtgtattgctttttaaaatctcaaaaattctgaaatct
cctcactagggagttttatataacatatgttgtgtgcagtgtattgctttttaaaatctcaaaaattctggaatct
ccccgctgggggtgttatataatatacggtatatgcactatattacctttct-aaaatccgaaaaattctgaattct

gaaata--tctg-ccttaagatttttaactaagggctaatggac---TGGTACAAGTTTATTGCAATAAACTTTCAATAGTATTTTAAATATATC
gaaata--tctg-ccttaagatttttaactaagggctaatggac---tggtacaagtttattgcaataaactttcaatagtattttaaatatatc
gaaaca--tctg-ccttaagatttttaactaaggggtattggat---tggtataagtttattacaataaactttcaatagtattttaaataagtc
gaaacacatctggccccaagggtttcggataagggattgtggacctg

TTTTAAAATCTATAGGAACACCCTATGTAAAGTAATG
ttttaaaatctataggaacaccctatgtaaagtaatg
ttttaaaatctataggaacacactatgaaaagtaatg
```

Chr5:5754098-5754240
Alignment
Human, Chimp, Rhesus, L1ME2 consensus, donor

Tgggcacagtggctcactcctgtaatccctgcactttgggaggttgaggtgagaggatcacttgaggctgggagctcaagaaaatgttc
tgggcacagtggctcgctcctgtaatccctgcactttgggaggttgaggtgagaggatcacttgaggctgggagctcaagaaaatgttc
tgggcacagtggctcactcctgtaatcccagcactttgggaggttgaggtgagaggatcactcgaggctgggagttcaagaaaatgtgc

tggcagctttctgatgaaactaaacaatcttagctgttctgtaactaggcatttacccaaaagaaatgaagatgtatgt***tca***<u>**TTTCTT**</u>C
tggcagctttctgatgaaactaaacaatcttagctgttctgtaactaggcatttacccaaaagaaatgaagatgtatgt***tca***-------
tggcagctttctgatgaaactaaaccatcttagctgttctgtagctaggcatttacccaaagaaatgaagatgtatgt***tca***-------
ctaggtatttacccaagagaaatgaaaacatatgtc***ca***-------
TCA------C

TGACCTACCTCCAAGGATCT---TCTCCAACCAAATCCTGTTCTTCTTCCTCTCTGAACACTCCTGGGGCTTTAGCACTTCATCCGTCC

TGACCTACCTCCAAGGATCTTCTTCTCCAGCCAAATCCTGTTCTTCTTCCTCTTTGAACACTCCTGGGGCTTTAGCACTTCATCCGTCC

TCATCTGCATGGATGTCCTCCTGGCTGGTCTGTCCTGTGAAAGCCTCTTGCact----------tgcacaagaatgctcataacagttatattcaaaat
--<u>caaaaacact</u>tgcacaagaatgctcataacagttatattcaaaat
--<u>cagaaacact</u>tgcacaagaatgctcataagagttatattcaaaac
--<u>caaaaagact</u>tgtacawgaatgttcatagcagctttattcataat
TCATCCGCATGGATGTCCTCCTGGCTGGTCTGTCCTGTGAAAGCCTCTTCCACT

agaCaaaaactggaaatggtttaagtgatcatcaataaaagaagaagtgaacaaactgatacattcatacaatagaattttcctcagca
agacaaaaactggaaatggtttaagtgatcatcaataaaagaagaagtgaacaaattgatacattcatacaatagaattttcctcagca
agacaaaaactggaaatggcttaagtgatcatcaataaaagaa---gtgaacaaattgatacattcatacaatggaatttgactcagca
agccaaaaactggaaa

aaaAagaaacaaattattgatatatgcaagacatgtatgattggcaaataacattatgcagtgaaacataagtcttacatgaaagagta
aaaaagaaacaaattattgatatatgcaagacatgtatgattggcaaataacattatgcagtgaaacataagtcttacatgaaagagta
aaaaagaaa----ttattgatatatgcaaagcatgtacgattggcaaacaatgttatgcagtgaaaaataagtcttacactaaagagta

--catTttatatgaagtttctatagcaggcaaaactaatctatgatagaaaaa-tcagaatagtggttacatccttgggatgtaggtgaggg
--cattttatatgaagtttctatagcaggcaaaactaatctatgatagaaaaa-tcagaatagtggttacatccttgggatgtaggtgaggg
--cattatagatgaagtttctatagcaggcaacactaatctatgatagaaaaa-tcagaatagtggttacacccttgggatgtaggtgaagg

ctgGctgagaagcaatatcaaggcctttctggggagatagtgacattctgtaccttgagagaggttgggtaacacaagttcatatattt
ctggctgagaagcaatatcaaggcctttctggggagatagtgacattctgtaccttgagagaggttgggtaacacaagttcatatattt
ctggctgagaagcagcatcaaggtctttctggggagatagtgacattctgtaccttgacagaggttgggtaacagaagttcatatattt

atcaaaactcattgaatgtatacttaagacttgcatgtttcattatatttaaattTATAGCAAGAGAAAAAA--TGTGTATATGAATATTA
atcaaatctcattgaatgtatacttaagacttgcatgtttcattatatttaaatttatagcaagagaaaaaa--tgtgtatatgaatatta
gtcaaatctcattgaatgtacacttaagacttgtgtgtttcattatatttaaatgtacggcaagagaaaaaaaatgtgtatatgaatatta

AACTTCAATTAATCCTATAAATACTGAAGTTTTTAGG
aacttcaattaatcctataaatactgaagtttttagg
aatttcagttaatcctataaatactgaagtttttagg

chr2:47268768-47268859

Alignment

Human, Chimp, Rhesus, AluJb consensus, donor

```
ATAGCAACTGCGGTAACCCCCATCTTCCACCCTTATCACCTCTTCGTGTGTGTTtttattattttgttgttgttgttgtttcttgagac
             Taacccccatcttccacccttatcacctcttcgtgtgtgttttattattttgttgttgttgttgtttcttgagac
                                        Tcttcatgggtgtttttattattttgttgttgttgttgtttcttgagac

Agggtcttactccagttgcccaggctggagtacaatggcataatcttggatcttggttcactgtatccttgacctcctgggctcagatg
agggtcttactccagttgcccaggctggagtgcaatggcataatcttggatcttggttcactgtatccttgacctcctgggctcagatg
agggtctcacttcagttgcccaggctagagtgcaatggcataatcttagatcttggttcactgtatccttgacctcctgggctcagatg
                                                         tcactgcagcctcgacctcccgggctcaagcg

Attctcccacctcagcctcctgagtagctgggactacaggagtgcaataccacatccggctaatttttgtatttgtagtagagacggg
attctcccacctcagcctcctgagtagctgggactacaggagtgcaataccacgtccggctgatttttgtatttgtagaagagacggg
attctcccacctcagcctcctgagtagctgggactacaggagtgcaataccacatctggctaatttttgtatttttagtagagatgat
atcctcctgcctcagcctcccgagtagctgggactacaggcgcgcgccaccacgcccggctaattttgtatttttt-gtagagacggg

gtttcgccatgttgtacagactgatctcaaacttctggtctcTCTGCCAAAGGCAGTTATAATTGCATGCTGCTGGCTGAGGGTCCATG
gtttcgccatgttgtacagactgatctcaaacttc------------------------------------------------------
gttttgccatgttgtacaggctgatctcaaacttctgggttc-----------------------------------------------
gtttcaccatgttgcccaggctggtctcgaactcctgggctc-----------------------------------------------
                                   TGGTCTCTCTGCCAAAGGCAGTTATAATTGCATCCTGCTGGCTGAGGGTCCATG

CTTTTCTTTTGTCCGGGCCCTGACCATCTCAACCTTTCACCTGCCGctcaaacttctgggttcaagcgatctgcctctgcctcctaaaa
----------------------------------------------ctcaaacTtccgggttcaagcgatctgcctctgcctcctaaaa
---------------------------------------------------------------aagcaatctgcctctgcctcctaaaa
-----------------------------------------------------------Aagtgatcctcccgcctcggcctcccaaag
CTTTTCTTTTGTCCAGGCCCTGCCCATCTCAACCTTTCACCTGCCGCT

tgctgggattacaggcgtgagccaccatgcccagGCTGTGTGCATTTTTAATCACTTCTCATTATG
tgctgggattacaggcgtgagccaccgtgcccaggctgtgtgcatttttaatcacttctcattatg
tgctgggattacaggcataagccaccatgcccaggctatgtgcgtttttaatcacttctcattatg
tgctgggattacaggcgtgagccaccgcgcccggcc
```

chr5:103039643-103039927
Alignment
Human, Chimp, Rhesus, MER5A1 consensus, donor

```
TCAAAATTTTTTGTTATGTTTTGGCAAGGGAATGTAATGATATATAGCCT
TCAAAATTTTTTGTTATGTTTTGGCAAGGGAATGTAATGATATATAGCCT
TCAAAATTTTTTGTTATGTggTGGCAAGGGAATGTAATGATATAaAGCCT

GAGGTCTGAAGTGATATAACCTGTTATTGCAGATAATACAACATTTACAT
GAGGTCTGAAGTGATATAACCTGTTATTGCAGATAATACAACATTTACAT
GAGGTCTGAAGTG-TATAACaTGTTtTTGCAGATAATAtAACATTTACAT

TACACGGTATGTATAAAACATATGTATATTACTTAATTTAAATATTAGGC
TACACGGTATGTATAAAACATATGTATATTACTTAATTTAAATATTAGGC
TAtACcGTgTGTATAAAACATATGTATATTACTTAATTTAAATATTAGGC

CGTGATTTTAAAATATGACTGTTCTATATGTCAGGCTCCAGTTTAGGTTG
tGTGATTTTAAAATATGACTGTTCTATATGTCAGGCTCCAGTTcAGGTTG
aGTGATTTTAAAATATGACTGTTCTgTATGcCAGGCTCCAGTTTAGGTTG

TTTAGGGCCTCCTtactgaaatttggcctacaaaccagcatgcagcaag---------
TTTAGGGCCTCCTTACTGAAATTTTGGCCTACAAACCAGCATGCAGCAAG---------
TTTAGGaCCTCCTTACTGAAATTTTGGCCTgCAAACCAGCATGtAGCAtG---------
             tactcaaagtgtggtccacggaccagca-gcatca-gcatcacctg

ggtgcttgccagaagtgcagaatttcaggttcc--------TTTTTAGCTCTGTGTGC
GGTGCTTGCCAGAAGTGCAGAATTTCAGGTTCC--------tttt-agc---------
GGTGCTTGCCAGAAGTGCAGAATTTCAGGT-cc--------gttttagc---------
ggagcttgttagaaatgcagaatctcgggccccaccccaga-----------------
                                               GCACTGTGTGC
CATCTTTTTGTAATCTCAGGTTTTATAGTCTCCTTTATCAATTGTAAGAG
--------------------------------------------------
--------------------------------------------------
--------------------------------------------------
CATCTTTTTGTAATCTCAGGTTTTATAGTCTCCTTTATCAATTGTAAGAG

GTGTGGGAGGCTAGTTTATATCTTTCAGAAGGGACACCTGGGTCAACAGG
--------------------------------------------------
--------------------------------------------------
--------------------------------------------------
GTGTGGGAGGCTAGTTTATATCTTTCAGAAGGGACACCTGGGTCAACAGG

TTTAAAGAACTTTGAAAGTTATTTGTATCTTTGCCAGTAACTGCATTGTG
--------------------------------------------------
--------------------------------------------------
--------------------------------------------------
TTTAAAGAACTTTGAAAGTTATTTGTATCTTTGCCAGTAACTGCATTGTG

TGAGAATCGCTGTAGCTGGTGTGTAAACTACCTTTGAATAAGAGGCAGCA
--------------------------------------------------
--------------------------------------------------
--------------------------------------------------
TGAGAATCGCTGTAGCTGGTGTGTAAACTACCTTTGAATAAGAGGCAGCA

AATGGAATTTATCAGGGGACAATGCCCTTTGTTTAGAAGGAGCAACAAAA
--------------------------------------------------
--------------------------------------------------
--------------------------------------------------
AATGGAATTTATCAGGGGACAATGCCCTTTGTTTAGAAGGAGCAACAAAA

GATGAATAAAAGGACACAGccagtctt----ctgaatcagaatcagcaatgtaa
---------------------------cctaCTGAATCAGAATCAGCAATGTAA
---------------------------cctaCTGAATtAGAATCAGCAATGTAA
---------------------------cctactgaatcagaatctgcgtttta-
```

GATGAATAAAAGGACACAGCCAGTCTT----***CTG***

tc-atgtctccaggggattcacaagcacactaaagtttgagacgcaTGGGC
TC-ATGTCTCCAGGGGATTCACAAGCAtACTAAAGTTTGAGAaGCATGGGC
TC-AgGTCTCCAGGGGATTCACAAGCAtACTAAAGTTTGAGAaGCATGGGC
acaagatccccaggtgattcatatgcacgttaaagtttgagaagca

TTGGATGACTCCTTTATAGTAA-GGTGAGTCGCCTCTTAAAAAGTCCTGTG
TTGGATGACTCCTTTATAGTAA-GGTGAGTCGCCTCTTAAAAAGTCCTGTG
TTGGATGACTCCTTTATAGTAAaGGTGAGTtGCCTCTTAAAAAGTCCTGTG

CATTTTTTACGGCCCAAGTCAGTAGTTTCTTGGCTAAATATGACGGTAAT
CATTTTTTACGGCCCAAGTCAGTAGTTTCTTGGCTAAATATGACaGTAAT
CATTgTTTgCGGCCCtAGTCAGTAGTTTCTTGaCTAAATATGAgGGTAAT

AACTGATTCTACAGAGGGCCTAAGAGTTAATGTGTCTTCTATAGCACCTG
AACTGATTCTACAGAGGGCgTAAGAGTTAATGTGTCTTCTATAGCACCTG
AACTGATTCTACAGAGGGtgTgAGAGTTAATGTGTCTTCTgTAGCACCTG

CCTTTCTATCATGCACAACTAAGGAAAATACTGAGAATGAAGACAGACTC
CCTTTCTATCATGCACAACTAAGGAAAATACTGAGAATGAAGACAGACTC
CCTTTCTATCATGCcCAACTAAGGAAAAcACTGAGAATGAAGACAGACTC

chr15:31987740-31988005
Alignment
Human, Chimp, Rhesus, L1MB3 consensus, donor

```
aaaagacaaatgacaatgcaggagaaatacttgcaacacatcagataaaa
AAAAGACAAATGACAATGCAGGAGAAATACTTGCAACACATCAGATAAAA
AAAAGACAAATGACAATGCAGGAGAAATAtTTGCAACACATCAGATAAAA

ggctattttcaTTTACATTGTATATTTTACAAATCACTTATTAAAAAAT
GGCTATTTTTCATTTACATTGTATATTTTACAAATCACTTATTAAAAAAT
GGCTATTTTTCATTTACATTGTATAcTTTACAgcTCACTTATTtAAAAAT

TTGTattgtgataagatatacataaaatttacgattttagtcacttctaa
TTGTATTGTGATAAGATATACATAAAATTTACcATTTTAGTCACTTtTAA
TTGTATTGgGATAAaATATACATAAAAcTTACcATTTTAGTCcCTTtTAA
                    cataaaatttaccattttaaccatttttaa

gtatacagtccggtggcattaagtacattcacattattgtgtaactatca
GTATACAGTCCGGTGGCATTAAGTACATTCACATTATTGTGTAACTATCA
GTATACAGTCCaGTGGCATTAAGTACATTCACATTATTGTGTAAgcATCA
gtgtacagttcagtggcattaagtacattcacattgttgtgcaaccatca

ccactatctgtccctagaactcatttatcatcccaaactaaaactcctta
CCACTATCTGTCCCcAGAACTCATTTATCATCCCAAACTAAAACTCCTTA
CCACcATCTGTCCCcAGAACTCATTTATCATCCCAAACTAAAACTCCTTA
ccaccatccatctccagaacttttttcatcttcccaaactgaaactctgta

cccattacacataacCAGGATTCTTTTACTTCGAAAGCTGAATTGCTAAT
CCCATTACAaATAAC-----------------------------------
CgCATTACACATAAC-----------------------------------
cccattaaacataac-----------------------------------
           TAACCAGGATTCTTTTACTTCGAAAGCTGAATTGCTAAT

TCCAAAGAACATTGTGTTGACAGTGCCTGCAATTAAGAAGCCATCACCTT
--------------------------------------------------
--------------------------------------------------
--------------------------------------------------
TCCAAAGAACATTGTGTTGACAGTGCCTGCAATTAAGAAGCCATCACCTT

TGTGTTATGACAGTTTTCTTCCTTACTACAAGCATTATTGTTACCTGGAA
--------------------------------------------------
--------------------------------------------------
--------------------------------------------------
TGTGTTATGACAGTTTTCTTCCTTACTACAAGCATTATTGTTACCTGGAA

TAAAAGATTCCTGCTTTTTTGTTCACATCTTGAGCTGGTTATGAGTATTA
--------------------------------------------------
--------------------------------------------------
--------------------------------------------------
TAAAAGATTCCTGCTTTTTTGTTCACATCTTGAGCTGGTTATGAGTATTA

ACCCATGTCACTGTTGGTACATTTATAGAGTATAAAATCACTCTATAATT
--------------------------------------------------
--------------------------------------------------
--------------------------------------------------
ACCCATGTCACTGTTGGTACATTTATAGAGTATAAAATCACTCTATAATT

GCAAATGGGCAAATAGGTTATTCCCATGAGAA------------ctcaccccgaaccc-tggc
--------------------------------tccccattctctCTCACCCCGAACCC-TGGC
--------------------------------tccccattctctCTCACCCCGAACCC-TGGC
--------------------------------tccccattcccccctccccccagcccctggc
GCAAATGGGCAAATAGGTTATTCCCATGAGAA------------CTC

aactaccattctattttctgtctctgtgaatctgaccattctgggtgact
```

```
AACTACCATTCTATTTTCTGTCTCTGTGAATCTGACCATTCTGGGTGACT
AACTACCATTCTATTTTCTGTCTCTGTGAATCTGACCATTCTGGGTGACT
aaccaccattctactttctgtctctatgaatttgactactctaggtacct

catataagtagaatcatacagtatttttcttttttttt-ctgactggcttat
CATATAAGTAGAATCATACAGTATTTTTCTTTTTTTTtCTGACTGGCTTAT
tATATAAGTcGAATCATACAGTATTTTTCTTTTTTT--CTGACTGGaTTAT
catataagtggaatcatacagtatttgtcctttt----gtgactggcttat

tt-----gtctcagcaaggttcatccttgttgtcgc-atgtttccgaatttccttt
tttatTTGTCTCAGCAAGGTTCATCCTTGTTGTCGC-ATGTTTCCGAATTTCCTTT
TT-----GaCTCAGCAAGGTTtATCCTTGTTGTCGCcATGTgTCtGAATTTCCTTT

ctgaagtctgagtaataatccactgtatcacatttattcatttatccatg
CTGAAGTCTGAaTAATAATCCACTGcATCACATTTATTCATTTATCCATc
CTtAAGTCTGAaTAATAATCCACTGcATCACATgTATTCATT-ATCCATc

aataaacacttaggttgtttttacttctttgctattgtgtataaATCAAT
AATAAACACTTAGaTTGaTTTTACTTCTTTGCTATTGTGTATAAATCAAT
AATAAACAtTTAGGTTGTTTcTACcTCTTTGCTAcTGTGTATgAATCAAT

ATTTTAAAAAGACAAATAATTCAGTTAATGGAGAAACTACAAGAGGGCTG
ATTTTAAAAAGACAAATAATTCAGTTAATGGAGAAACTACAAGAGGGCTG
ATTTTAAAAAGACAAATAATTCAGTTcATGGAGAAACTACAgcAGGGCTG

GGGCTTGATTCATACAGAAGTCTGCAGGAGAAAAGACAAACCAGCAA
GGGCTTGATTCATgCAGAAGTCTGCAGGAGAAAAGACAAACCAGCAA
GGGCcTGATTCATgCAGAAGTCTGCAGGgGAAAAGACAcACCAGCgA
```

chr10:96228347-96228586

Alignment

Human, Chimp, Rhesus, AluSc consensus, donor

```
ATCAGGCCTATTTGGTGGAAGCCAGTAGGTGGTTAGAAATCTTAAAGGCG
ATCAGGCCTATTTGGTGGAAGCCAGTAGGTGGTTAGAAATCTTAAAGGCG
ATCAGGCCTATTTGGTGGAAGCtAGcAGGTGGTTAGAAATCTTAAAGGCG

GGATTTGTAGTTAAACTGGAAATGTAggccaggcgtggtggctcagtcct
GGATTTGTAGTTAAACTGGAAATGTAGGCCAGGCGTGG---CTCAGgCCT
GGATTTGTAGTTAAACTGGAAATGTAGGCtgGatGTGGTGGCTCAcgCCT
                          ggccgggcgcggtggctcacgcct

gtaatcccagcactttgggaggcagaggcaggggcagaccatgaggtcaa
GTAATCCCAGCACTTTGGGAGGCAGAGGCgGGGGCAGACCATGAGGTCAA
GTAATCCCAGCACTTTGGGAGGCcGAGGCAGGGGCAGAtCATGAGtTCAA
gtaatcccagcactttgggaggccgaggc-ggg-cggatcacgaggtcaa

aagattgagaccatcctggccagcatgatgaaaccccatccctactaaaa
AAGATTGAGACCATCCTGGCCAaCATGATGAAACCCCATCCCTACTAAAA
AAGATcGAGACtATCCTGGCCAaCgTGgTGAAACCCCATCtCTACTAAAA
gagatcgagaccatcctggccaacatggtgaaaccccgtctctactaaaa

-tatgaaaattagctggccatggtggtgtgcacctgtagtcccagctgctA
-TATGAAAATTAGCTGGgCATGGTGGTGTGCACCTGTAGTCCCAGCTGCT-
-TAcaAAAATTAGCTGGgCATGGTGGcGTGCACCTGTAGTCCCAGCTatT-
atacaaaaattagctgggcgtggtggcgcgcgcctgtagtcccagctact-
                                            GCTACTA

TTTGGGGAGTCTCTTATGTGTAGCTGAATGTAATTGCTTAATCATATACC
--------------------------------------------------
--------------------------------------------------
--------------------------------------------------
TTTGGGGAGTCTCTTATGTGTAGCTGAATGTAATTGCTTAATCATATACC

AATAAATCAATGATGGTGCTCTAAAGGTCAAGAGCCATCACAAACTCCAA
--------------------------------------------------
--------------------------------------------------
--------------------------------------------------
AATAAATCAATGATGGTGCTCTAAAGGTCAAGAGCCATCACAAACTCCAA

CCACATTTAAAATCCATGGAGTTTAGTCCTCACATCCAGATCCATTGAAT
--------------------------------------------------
--------------------------------------------------
--------------------------------------------------
CCACATTTAAAATCCATGGAGTTTAGTCCTCACATCCAGATCCATTGAAT

CTTACAATTCCTAGTATTGCCTACACATACTAACTACCTTAAATCTTGCA
--------------------------------------------------
--------------------------------------------------
--------------------------------------------------
CTTATAATTCCTAGTATTGCCTACACATACTAACTACCTTAAATCTTGCA

AATCGATTCTGTCCAAAGCTGCTTTCTACCTATGTCATAGcacctgctca
------------------------------------------------CA
------------------------------------------------CA
------------------------------------------------cg
AATCGATTCTGTCCAAAGCTGCTTTCTACCTATGTCATAGCACC

ggaggctgaggcaggagaatcgcttgaacacggaaggtggaggttgcagt
GGAGGCTGAGGCAGGAGAATCGCTTGAAtgCGGAAGGTGGAGGTTGCAGT
GGAGGCaGAGGtAGGAGAATCGCTTGAACcCGagAGGTGGAtGTTGCAGT
ggaggctgaggcaggagaatcgcttgaacccgggaggcggaggttgcagt
```

```
gagcggagattgtg-cactgcactccagtctggtgacaaagcgagactctg
GAGCGGAGATTGTG-CACTGCACTCCAGcCTGGTGACAAAGCGAGACTCTG
GAGCaGAGATTGTGcCACTGCACTCCAGcCTGGTGACAgAGCGAGACTCTG
gagccgagatcgcgccactgcactccagcctggcgacagagcgagactccg

tctcaaaaaaaaaaaa----------GTGGAAATGTGATTTCAATTAAGCTGTTTCTCCAA
TCTCAAAAAAAAAAAAa---------cTGGAAATGTGATTTCAATTAAGCTGTTTCTCCAA
TCTCAAAAAAAAAAAAaaaaacaaaacTGGAAATGTGATTTaAATTAAGCTGTTTCTtgAA
tctca

TGATAAAATGAAAATTTCTAAAGGTAAATGAAATACAGC
TGATAAAATGAAAATTTCTAAAGGTAAATGAAATACAGC
TGgTAAAATGAAAgTTTCTAAAGGTAAATGAggTACAGC
```

chr15:31987740-31988005
Alignment
Human, Chimp, Rhesus, L1MB3 consensus, donor

```
aaaagacaaatgacaatgcaggagaaatacttgcaacacatcagataaaa
AAAAGACAAATGACAATGCAGGAGAAATACTTGCAACACATCAGATAAAA
AAAAGACAAATGACAATGCAGGAGAAATAtTTGCAACACATCAGATAAAA

ggctattttcaTTTACATTGTATATTTTACAAATCACTTATTAAAAAAT
GGCTATTTTTCATTTACATTGTATATTTTACAAATCACTTATTAAAAAAT
GGCTATTTTTCATTTACATTGTATAcTTTACAgcTCACTTATTtAAAAAT

TTGTattgtgataagatatacataaaatttacgattttagtcacttctaa
TTGTATTGTGATAAGATATACATAAAATTTACcATTTTAGTCACTTtTAA
TTGTATTGgGATAAaATATACATAAAAcTTACcATTTTAGTCcCTTtTAA
                         cataaaatttaccattttaaccattttaa

gtatacagtccggtggcattaagtacattcacattattgtgtaactatca
GTATACAGTCCGGTGGCATTAAGTACATTCACATTATTGTGTAACTATCA
GTATACAGTCCaGTGGCATTAAGTACATTCACATTATTGTGTAAgcATCA
gtgtacagttcagtggcattaagtacattcacattgttgtgcaaccatca

ccactatctgtccctagaactcatttatcatcccaaactaaaactcctta
CCACTATCTGTCCCcAGAACTCATTTATCATCCCAAACTAAAACTCCTTA
CCACcATCTGTCCCcAGAACTCATTTATCATCCCAAACTAAAACTCCTTA
ccaccatccatctccagaacttttcatcttcccaaactgaaactctgta

cccattacacataacCAGGATTCTTTTACTTCGAAAGCTGAATTGCTAAT
CCCATTACAaATAAC-----------------------------------
CgCATTACACATAAC-----------------------------------
cccattaaacataac-----------------------------------
           TAACCAGGATTCTTTTACTTCGAAAGCTGAATTGCTAAT

TCCAAAGAACATTGTGTTGACAGTGCCTGCAATTAAGAAGCCATCACCTT
--------------------------------------------------
--------------------------------------------------
--------------------------------------------------
TCCAAAGAACATTGTGTTGACAGTGCCTGCAATTAAGAAGCCATCACCTT

TGTGTTATGACAGTTTTCTTCCTTACTACAAGCATTATTGTTACCTGGAA
--------------------------------------------------
--------------------------------------------------
--------------------------------------------------
TGTGTTATGACAGTTTTCTTCCTTACTACAAGCATTATTGTTACCTGGAA

TAAAAGATTCCTGCTTTTTTGTTCACATCTTGAGCTGGTTATGAGTATTA
--------------------------------------------------
--------------------------------------------------
--------------------------------------------------
TAAAAGATTCCTGCTTTTTTGTTCACATCTTGAGCTGGTTATGAGTATTA

ACCCATGTCACTGTTGGTACATTTATAGAGTATAAAATCACTCTATAATT
--------------------------------------------------
--------------------------------------------------
--------------------------------------------------
ACCCATGTCACTGTTGGTACATTTATAGAGTATAAAATCACTCTATAATT

GCAAATGGGCAAATAGGTTATTCCCATGAGAA------------ctcaccccgaaccc-tggc
--------------------------------tccccattctctCTCACCCCGAACCC-TGGC
--------------------------------tccccattctctCTCACCCCGAACCC-TGGC
--------------------------------tccccattccccctcccccccagcccctggc
GCAAATGGGCAAATAGGTTATTCCCATGAGAA------------CTC

aactaccattctattttctgtctctgtgaatctgaccattctgggtgact
```

```
AACTACCATTCTATTTTCTGTCTCTGTGAATCTGACCATTCTGGGTGACT
AACTACCATTCTATTTTCTGTCTCTGTGAATCTGACCATTCTGGGTGACT
aaccaccattctactttctgtctctatgaatttgactactctaggtacct

catataagtagaatcatacagtatttttctttttttt-ctgactggcttat
CATATAAGTAGAATCATACAGTATTTTTCTTTTTTTTtCTGACTGGCTTAT
tATATAAGTcGAATCATACAGTATTTTTCTTTTTTT--CTGACTGGaTTAT
catataagtggaatcatacagtatttgtcctttt----gtgactggcttat

tt-----gtctcagcaaggttcatccttgttgtcgc-atgtttccgaatttccttt
tttatTTGTCTCAGCAAGGTTCATCCTTGTTGTCGC-ATGTTTCCGAATTTCCTTT
TT-----GaCTCAGCAAGGTTtATCCTTGTTGTCGCcATGTgTCtGAATTTCCTTT

ctgaagtctgagtaataatccactgtatcacatttattcatttatccatg
CTGAAGTCTGAaTAATAATCCACTGcATCACATTTATTCATTTATCCATc
CTtAAGTCTGAaTAATAATCCACTGcATCACATgTATTCATT-ATCCATc

aataaacacttaggttgtttttacttctttgctattgtgtataaATCAAT
AATAAACACTTAGaTTGaTTTTACTTCTTTGCTATTGTGTATAAATCAAT
AATAAACAtTTAGGTTGTTTcTACcTCTTTGCTAcTGTGTATgAATCAAT

ATTTTAAAAAGACAAATAATTCAGTTAATGGAGAAACTACAAGAGGGCTG
ATTTTAAAAAGACAAATAATTCAGTTAATGGAGAAACTACAAGAGGGCTG
ATTTTAAAAAGACAAATAATTCAGTTcATGGAGAAACTACAgcAGGGCTG

GGGCTTGATTCATACAGAAGTCTGCAGGAGAAAAGACAAACCAGCAA
GGGCTTGATTCATgCAGAAGTCTGCAGGAGAAAAGACAAACCAGCAA
GGGCcTGATTCATgCAGAAGTCTGCAGGgGAAAAGACAcACCAGCgA
```

chr1:224786689-224786777
Alignment
Human, Chimp, Rhesus, AluJo consensus, donor
CACGCCCTCCCCAACCATTCCCTCCTTCCTCCATAGTAATGAGCTTTAAA
cacgccctccccaaccattccctccttcctccatagtaatgagctttaaa
catgccctccctatccattctccccttcctccatagtaatGAGCTTTAAA

TATTTGATTTAGAtttttttcttt-gagacagaatctcgctctgtcaccca
tatttgatttagatttttttcttt-gagacagaatctcgctctgtcaccca
TACCTGATTTAGAttttttttctttgagagagggtctcactccgtcaccca
-----------------------t-gagacagggtctcgctctgtcgccca

ggctggagtgtagttgtgcggtcttggctcactgcagccttaacctccag
ggctggagtgtagttgtgcggtctcggctcactgcagccttaacctccag
ggctggagtgtagttgtgcgatctcggctcactgcagccttaacctcccg
ggctggagtgcagtggcgcgatcatagctcactgcagcctcgaactcctg

gactcaagtgatccttctgcctcagcctcctgagtagctgagactacagc
gactcaagtgatccttctgcctcagcctcctgagtagctgagactacagc
ggctcaagtgatcctcctgcctcagcctcctgagtagctgagactacagc
ggctcaagcgatcctcctgcctcagcctcccgagtagctgggactacagg

tatgtgcc***acca***AGGACTCCTAGACTGTGCACTACTGAAACGTGGGCAGA
tatgtgcc***acca***--------------------------------------
tatgtgcc***acca***--------------------------------------
cgcgcgcc***acca***--------------------------------------
ACCAAGGACTCCTAGACTGTGCACTACTGAAACGTGGGCAGA

ATTCTGAACCACACCTGGAAGCCGAGGGGACATGCCTGGCTAATGTTTTA
--------------------------------tgcctggctaatgtttta
--------------------------------tgcctggctaatgttgtg
--------------------------------cgcccggctaattttgt
ATTCTGAACCACACCTGGAAGCCGAGGGGACA

ATtaatttatttatttattttg---tagaaacaggatctcattatgttgctca
atttatttatttatttattttg---tagaaacaggatctcattatgttgctca
actttttttttttttttttttttatagaaacaggatcttattatcttgctca
atttttt--------------g---tagagacggggtctcgctatgttgccca

ggcaggtctagaattcctgggctcaagcaatcctcctgcctcaggctccc
ggcaggtctagaattcctgggctcaagcaatcctcctgcctcaggctccc
ggcaggtctagaattcctaggctcaagcaatcctcctgcctcaggctccc
ggctggtctcgaactcctgggctcaagcaatcctcccgcctcggcctccc

aaagtgTGATTTAGATCATTTATTTAACCTTTAATTTAGAGTAAAGTTTC
aaagtgtgatttagatcgtttatttaacctttaatttagagtaaagtttc
aaagtgTCATTTAGATTGTTTATTTAACTTTTAATTTAGAATAAAGTTTC
aaagtg

CTAGCCTCTTTTGTAGTTAGATGA
ctagcctcttttgtagttagatga
CTAGCCTCTTTTGTAGTTAGATGA

chr10:130986332-130986439

Alignment

Human, Chimp, Rhesus, Tigger2a consensus, donor

```
CTTTCCTATCTGTAGCACTTTCATATATATTCTGTATTTGCAGATAGTTC
CTTTCCTATCTGTAGCACTTTCATATATATTCTGTATTTGCAGATAGTTC
CTTTCCTATCTGTGGCACTTTCACATATATTCTATATTTGCAGATAGTTC

TAAATGACCAAGAGGTTGCTGTTTCTTTGAATGCAGGATAGAGTAcagtt
TAAATGACCAAGAAGTTGCTGTTTCTTTGAATGCAGGATAGAGTAcagtt
TAAATGACCAAGAGGTTGTTGTTTCTTTGAATGCAGAATAGAGTAcagtt
                                             CAGTT

gacacttgaacgatgcggaggctcgaggcgccgacaccca-cacagtcaaa
gacacttgaacgatgcggaggctcgaggcgccgacaccca-cacagtcaaa
gacacttgaacaatgtggaggctcgaggctccgacaccca-cacagtcaaa
GACCCTTGAACAACGCGGGGGTTAGGGGCGCTGACCCCCCGCGCAGTNAAA

a-tcc-cctgtagctttcgactttccaaaaacct-----------gtccactattgaccagaa
a-tcc-cctgtaactttcgactttccaaaaacct-----------gaccactattgaccagaa
a-tcc-cctgtagctttcgactttccaaaaacct-----------aaccacagttgaccagaa
AATCCACGTATAACTTTTGACTCCCCMAAAACTTAACTACTAATAGCCTACTGTTGACTGGAA

gccctgccaataacataaacagttgatt----------------ttatcctgtattgtatactgta
gccctgccaataacataaacagttgatt----------------ttatcctgtattgtatactgta
gccctgccaataatgtaaacagttgatt----------------atatcctgtattgtacactgta
GCCTTACCGATAACATAAACAGTCGATTAACACATATTTTGTATGTTATATGTATTATATACCGTA

gtcttacaataaagtaagctatagaaaagaaaatgttattaagaaaagca
gtcttacaataaagtaagctagagaaaagaaaatgttattaagaaaagca
ttcttacaataaagtaagctagagaaaagaaaatgttactaagaaaatca
TTCTTACAATAAAGTAAGCTAGAGAAAAGAAAATGTTATTAAGAAAATCA

taaggaagggaaaatatatttactattctttaagtggCTTACTTATACTG
taaggaagggaaaatatatttactattctttaagtggc------------
taaggaagagaaaatatatttactattctttaactggc------------
TAAGGAAGAGAAAATATATTTACTATTCATTAAGTGGA------------
                                               CTG

GCCGGGAGATGTAGGAGGAAGTTTCCTGCAGGGATGGGGAGGAGGCTCTG
--------------------------------------------------
--------------------------------------------------
--------------------------------------------------
GCCGGGAGATGTAGGAGGAAGTTTCCTGCAGGGATGGGGAGGAGGCTCTG

AGAAAGATTTTGTTTTCCAATAAGAGCACCAGGTACATTGGTACAA---ggat
----------------------------------------------agtggat
----------------------------------------------agtggat
----------------------------------------------AGTGGAT
AGAAAGATTTTGTTTTCCAATAAGAGCACCAGGTACATTGCT

---------tactataaaggccttcatcatcattgtcttcacattgaataggctgagga
---------tactataaaggccttcatcatcattgtcttcacattgaataggctgagga
---------tattataaaggccttgatcatcattgtcttcacattgaataggctgagga
CATCATAAAGGTCTTCATCCTCNTCGTCTTCANGTTGAGTAGGCTGAGGA

gggggaggaggaggagggataggtcttg-tgtctcagggacagcagagata
gggggaggaggaggaggggtaggtcttg-tgtctcagggacagcagagata
gggggaggaggaggaggggtaggtcttg-tgtctcagggacagcagagaca
---------GGAGGAGGGGTTGGTCTTGCTGTCTCAGGG-TGGCAGAGATG

gaaaaaaatccacatataataggacccacacagttcaaatccatgttgtt
gaaaaaaatccacatataataggacccacacagttcaaatccatgttgtt
gaaaacaattcacatataataggacccacacagttcaaatccatgttgtt
```

GAAGAAAATCCGCGTATAAGTGGAYCCACGCAGTTCAAACCCATGTTGTT

tacgggtaaactgTATATGAAAAGTGAGTATGGTAAAACCATCCATGTTT
tacgggtaaactgTATATGAAAAGTGAGTATGGTAAAACCATCCATGTTT
tatgggtaaactgTGTATGAAAAGTGAGTATGGTAAAACCATCCATGTTT
CAAGGGTCAACTG

TTAATATGCAAATATAAATAAGATGAGACAACAGGCTAAGAA
TTAATATGCAAATATAAATAAGATGAGACAACAGGCTAAGAA
TTAATATGCAAATATAAATAAGATAAGATAACAGGCTAAGAA

chr11:122113399-122113556
Alignment
Human, Chimp, Rhesus, L2 consensus, donor
TCGGGAAgccaggcgtggtggttcacacccgcaatctcagctacttggga
TCGGGAAgccaggcgtggtggttcacacctgcaatctcagctacttggga
TCAGGAAgccaggcgtggtggttcacacctgtaatcccagctacttggga

ggctgaggagagaggatcgcttgagcccaggagtttcagtccagcttggg
ggctgaggagagaggatcacttgagcccaggagtttcagtccagcttggg
ggctgaggaaagaggatcgcttgagcccaggagtttcagtccagcccagg

caacatagtgagaccctctctcAaaatgaaatgaaatggaatgaaatgaa
caacatagtgagaccctctctcaaaatgaaatgtaatggaatgaaatgaa
caacatagtgagaccctgcctcgaaatgaaatggaatggaatggaatgaa

gaaatgaaaggaaataaa----GGAtcaaggaaagcaacatagaagatttaagc
gaaatgaaaggaaataaa----GGAtcaaggaaagcaacatagaagatttaagc
atgaaatgaagaaatgaaatAAGTAtcaaggaaagcaacatagaagatttaagc

taiga-ttgagaggtgggcagagatattactgtagggaagggaaagaatgt
taiga-ttgagaggtgggcagagatattactgtagggaagggaaagaatgt
taagacatgagaggtgggcagagatattagtgtagggaaggagaagaatgt

tcctgaagaaagaatgcatgcccactgctcagctgtgagaaaatgtggca
tcctgaagaaagaatgcatgcccactgctcagctgtgagaaaatgtggca
tcctgaagaaagaatgcatgcccattgctcagctgtgagaaaatgtggca

cgttcaaggaactgaaagaaatctaatgttgccggaagagggtagcctaa
cgttcaaggaactgaaagaaatctaatgttgctggaagagggtagcctaa
cgttcgaggcactgaaagaaatctagtgttgctagaagaggttagcctaa

agtaaaccagggaagggccagaaaatagagcatacag----gcaaattagccat
agtaaaccagggaagggccagaaaatggagcatgcag----acaacttagccat
agtaaaccaggtaagggccagaaaatagagcatgcaggtaggtggcttagccat

gttaaggttctggaactttaccctagaggaaatagggagctactgggttg
gttaaggttctggaactttacgctagaggaaatagggagctactgggatg
gttaaggttctggaactttaccctagaggaaatagggagctactgagatg

(TE insertions in Rhesus)
tttttgtcacgcacatccgtgtgaagagaccaccaaacaggctttgtgtg
agcaacaaggcagtttatttcacctgggtgcaagcgggctgaatccaaaa
agagagtcagcaaagggtggtgggatcatcattggttcttataggttttg
ggataggcagtggagttaggagcaatgttgcgggcagggggtggatctca
caaagtatattctcaagggtggggagaattacaaagaaccttcttaaggg
tggggaagattacaaagtacattGATCAGcgcccgccttgtttttttgtat
tttt tagtagagacggggtttcaccgtgttagccaggatggtttcgatc
tcctgacctcatgatccgcccgtctcggcctcccaaagtgctgggattac
aggcttgagccaccacgcccggcctattttcacttcttttgtggatcttc
agttgcttcaggccatctggatgtgcacatgcaggtcacaggggcttagc
ttgggctcagaggcctgacag

ttttaagaagaagagtgtatgattatcattgtatatttggagaataaatt
ttttaagaagaagagtgtatgattattattgtatatttggagaataaatt
ttttaagcagaagagtgtatgattattgttgtatatttggagaataaatt

ggaaagggcaaaaccggaagcaggagatttgttaaagctgccggagttgt
ggaaagggcaaaactggaagcaggagattt-taaaagctgctggagtagt
ggaaagggcaaaactggaagcaggagatttgttaaagctgctggagtact

caaagcgaggggtgat-ggggcttaacacagagagaaaacaagggataaaa
caaagcgaggggtgataggggcttaacacagagagaaaacaagggataaaa
caaagcaaggggtgataagggcttagcatagagagaaaacaagggatcaaa

```
aagaaccagtgtccaagaaatttaggaggcacacatggacaggacctaat
gagaaccagtgtccaagaaatttaggaggcacacatggacaggacttaat
gagaaccaatgcctaagaaatttaggaggcacacatggacaggacttaat
                                      ACAGGATTTGCT

gatgaattagataggagaggtgagagaaagagagaagtcaaggatggatc
gatgaattagataggagaggtgagagaaagagagaagtcaaggacagatc
gatgaattagataggagagttgagagaaagagagatgtcaaggacggatc
GATGGATTGGATGTGGGGTGTGAGAGAAAGAGAGGAGTCAAGGATGACTC

ttaggtggatcttagcctgTTGATCTCAGTGCTAAGTGTAGTACAGGCCG
ttaggt--------------------------------------------
ttaggt--------------------------------------------
CAAGGT---TTTTGGCCTG-------------------------------
    GTGCGTGTTAGCCTGTTGATCTCAGTGCTAAGTGTAGTACAGGCCA

TGAGGTAGATGCTCAGTAAGAATGAGGAGCGTCGCTAAAGCCCCACCTTC
--------------------------------------------------
--------------------------------------------------
--------------------------------------------------
TGAGGTAGATGCTCAGTAAGAATGAAGAGCGTCGCTAAAGCCCCACCTTC

TACATAAACTCCTGAATTCTCCCAGCATTCTCTCTGTGTTCCCCTAGCCC
--------------------------------------------------
--------------------------------------------------
--------------------------------------------------
TACATAAACTCCTGAATTCTCCCAGCATTCTCTCTGTGTTCCCCTAGCCC

TTTTTTTTTTCATTCTGCATGAATGAAA------cttgggaaattgcatgga--tgct
----------------------------ttctaacttgggaaattgcatgga--tgct
----------------------------ttctaacttgggaaattgcatgga--tgct
--------------------------------AGCAACTGGAA--GGATGGAGTTGCC
TTTTTTTT-CCATTCTGCATGAAT

ctgccctgagccaag--agactctggagagaagcaaaattggcggggaaaaa--
ctgccctgagccaag--agactctggagagaagcaaaattggcggggaaaaa--
gtgccctaagccaag--agactctggagagaagcgagattggaggggaaaaa--
ATTAACTGAGATGGGGAAGACTGTGG-GAGGAGC-AGGTTTGAGGGGGAAGATC

ggtgagttcactttttgaacacatgaggtttagtgcaagcaagatattca
ggtgagttcaccttttgaacacatgaggtttagtgcaagcaagatattca
ggcgagttcattttttgaacacacgaggtttagtgcaagtaagatattca
-AGGAGTTCAGTTTT

agtggagatttctagtaaggaataggtcaggacgccagagggttgagcag
agtggagatttctagtaaggaataggtcacgatgccagagggttgagcag
agtggaggtttctagtaaggaataggtcaggaagccagagggtggagcag

gagaaactggagggacctggagactaagggcatccttagcatgtagaagc
gagaaactggagggacctggagactaagggcatccttagcatgtagaagc
gagaaactgcagggacctggagactaagggcatccttagcatgtaaaagc

tggaggtatggcccagggaagagctgggattacccggagagaatgagtag
cggaggtatggcccagggaagagctgagattacccggagagaatgagtag
cggaggtagagcccagggaatggctgagattacccagagagaatgagtag

aatgtatggagaaagactggacagattcctgaggaatactagcaattaaa
aatgtatggagaaagactggacagattcctgagaaatactagcagttaaa
aatgtatggagaaagattggacagattcccgaggaatactagcaattaaa

gtgcctgtagagaagtcagagtttacaaaggaaactgagaataagccact
gtgcctgtagagaagtcagagtttacaaaggaaactgagaataagccact
```

```
gtgcctgtagagaagtcagagtttacaaaggaaaccgagaataagccact

agagaaaagggcaaaagacaaaccaaaggggcataatcaggaaagtaaaa
agagaaaagggcaaaagacaaaccaaaggggcataatcaggaaagtaaaa
agagaagagggtaaaagacaaaccaaaggggcagaatcaggaaagtaaaa

gtagagaacaaagggagggctcctaatgggaaatgcc---------gttgagaggtcaa
gtagagaacaaagggagggctcctaataggaaatgcc---------gttgagaggtcaa
gtagagaacaaagggagggttcgtagtgggagacgcccccactactgttgagaggccaa

gagacaaaaaTCCCATTGAATAGAGTGTGGTgactcagattctcactg----tt
gagacaaaaaTCCCATTGAATAGAGTGTGGTgactcagattctcactg----tt
gagacaaaaaTCCCGTTGAATAGAGTGTGGTGACTCAGATTCTCACTGCTGCTT

acttactaagtgaccttagaccagttattta-----cactgagcctcaTCCATAA
acttactaagtgaccttagaccagttattaattttacactgagcctcaTCCATAA
ACT----CAGTGACCTGAGACCTGTTATTAATTTTACACTGAGCCTTATCCATAA

GTTGGAACGATTGCATTGTATTTAGATTGGTGTCTTAATACATGTG
GTTGGAACGATTGCATTGTATTTAGATTGGCGTCTTAATACATGTG
TTTGGAACGATTGCATTGTATTTAGATTGGCATCTTAATACATGTT
```

chr12:54320736-54320817
Alignment
Human, Chimp, Rhesus, MER49 consensus, donor

```
ggctagtttcgaactcctgagctcaagtgatccatccaccttggcctccc
ggctagtttcgaactcctgagctcaagtgatccatccaccttggcctccc
ggctagttttgaactcctgagttccagtgatctacccacctcagcctccc

aaagtgctaggattacaggcgtgagccaccgcccccagcaggaggcttta
aaagtgctaggattacaggcgtgagccaccgcccccagcaggaggcttta
aa-gtgctaggattataggcctgagccaccacccctggaaagtgtcttta
                                          aggagacttta

tttcttaaaaa-ctgttacagcatgtaaggtggccattctaacaggctggg
tttcttaaaaa-ctgttacagcatgtaaggtggccattctaacaggctggg
tttcttaaaaaactgttgcagcatgtaaggtggccattctaacaggctggg
tttcttataaa-gggttacagcctgcaaggtggccatcccg-caggctggg

aagcataggctccagccaaagcccaaaaggcaggcacttcgagggagagg
aagcataggctccagccaaagcccaaaaggcaggcacttcgagggagagg
aagcataggctccagtcaaagcccaaaaggtaggcacttcgagggagagg
aagcgtagcctctggccaagacc--agagacaggcacttcgaaggaggag

caggtggaacagggatttatgtgctaagcaggttggccaggtgtacacat
caggtggaacagggatttatgtgctaagcaggttggccaagtgtacacat
caggtggaacagggatttatgtgctaagcaggttggccaagtgtacacat
gggttggggcaggagctttatgctgaa-cgggttggctaagcatacatat

tcagtaggttacgggaggagctaggaatattcctgaaggtggtcctaaca
tcagtaggttatgggaggagctaggaatattcctgaaggtggtcctaaca
tcagtaggttatgggaggacctaggaatattcctgaaggtggtcctaaca
tcaacaggttacaggaggagctatgaatattcatgaaggtggtcctgacg

tgtaggtgctaaataaacatatatgtagcatatgacccatgttcaccttg
tgtaggtgctaaataaacatatatgtagcatatgacccatgttcaccttg
tgtatgtgctaaataaacatgtatgtagcatatgacccatgttcaccttg
catgcgtattgaacaaacatgcatgtaacatacgacccatgttcactttg

gggtgcaaacttaacatttacgtgcattacaatttagacccttacatcaa
gggtgcaaacttaacatttaaatgcattacaatttagacccttacctcaa
ggatgcaaacttaacatttaaatgcattacgattTAG-------------
gggtggagacttaacatttaaatgtattacaattaggccctatacgtcaa

aaggtgaagcagagacacaaaggcattcaagtgtgcagcctctgtaaact
aaggtgacgcagagacacaaaggcactcaagtgtgcagcctctgtaaact
--------------------------------------------------
aaggtttttcag-gacacgaaggcgtgcaagtgcacagcctctgtaaact

ggccagaagcagcccctgggcagtggtcttcttatcaggagaaagttact
ggccagaagcagtccctgggcagtggtcttcttatcaggagaaagttact
--------------------------------------------------
ggccagaaccagtccatggccggtggtct-cttatcaggagaaagttact

gaaatcaatcatttggccaatcaaagctgtagttatggcttgtggaacag
gaaatcaatcatttggccaatcaaagctgtagttatggcttgtggaacag
-------------GGGccaagcaaagctgtagctatggcttgtggaacag
gaaatcagtctcttgtccaatcaaagctgtagttatggctggtggaacag

gggagtcagccagtaagtgtctggtggtggaggagctacaactgttttgc
gggagtcagccagtaagtgtctggtggtggaggagctacaactgttttgc
gggagtcagtcagtaagtgtctggtggtggaggagctacgactgttttac
ggg-gtcagttagtcagcgtctggtg-tggatgagctgcaattgttttaa

tattgcttatctcgaagccagagcttgtttagctgctagagaaaaaggaa
```

```
tattgcttatcttgaggccagagcttgtttagctgctagagaaaaaggaa
tattgcttatctcgaggccagagttgtttagctgctaaagaaaaaggaaa
tattgcttatctcgaggccagtgcttgtttagctgttagagaaaaagaaa

aaccttgtggcagttagaacatagtttattctttaagtgtaggaatgtgt
aaccttgcggcagttagaacatagtttattctttaagtgtaggaatgtgt
aaccttgtagcagttagaacatagtttattctttaagtataggaatatgt
aaccttgtggcagttagaacatagtttattctttaagtgtaggggtgcgt

gacttaacctttgcctggcatggccttaagtcctgttcataatttgatat
gacttaacctttgcctggcatggcctgaagtcctgttcataatttggtat
gacttaacctttgcctggcatggccttaagtcctgttcacaatttggtat
gacttaacccttgcctggcatggccttaggtcctgtttataatttggtat

attattgccacaaagagtctattctgtcaatctcatgacctctattttaa
cttattgccacaaagagtctattctgtcaatctcatgatctctattttaa
cttattgccacaaagagtctattatgtcaatctcatgatctctattttaa
cttattgccacaaagagtccgttctgtcagtcttatgatctctattttaa

cattaatgctggtcaattgttgtgtctaaaccatagaagagaggaggtac
cattaatgctggtcaattgttgtgtctaaaccatagaagacaggaggtac
cattaatgctggtcaattgttgtgtctaaaccatagaagagaggaggtat
cattaatgctggtcagttgttgtgtctaaaccacaaaagggagggggtat

aataaagtgtgtctgaccttctattccatcTTCTAGATTGTTCTGGATCC
aataaagtgtgtctgaccttctattccatc--------------------
aataaagcgtgtctgaccttccattccatc--------------------
aatgaggcgtgtctgacctcccatcccgtc--------------------
                          CATCTTCTAGATTGTTCTGGATCC

CTGAGCACAGTGATTGCCATGGCTGCACCGGTAGCCAGAGGTCCATGTCA
--------------------------------------------------
--------------------------------------------------
--------------------------------------------------
CTGAGCACAGTGATTGCCATGGCTGCACCGGTAGCCAGAGGTCCATGTCA

GTCATGAAAGCAGccatcatggctgaggactccgtttttaaggtttctca
------------------atggctgaggactctgtttttaaggtttctca
------------------acggccgaggactcagtttttgaggtttctca
------------------atggccgggaactcagtttttaaggtttctc-
GTCATGAAAGCAG

gtgggTGGGGGGCGTGGGATTTCATCTTTAGTTTACAatatgc
gtgggtgggggcgtaggatttcatctttagtttacaatatgc
gtgggtgggggacttaggatttcatctttagtttacaatatgc
tggggt
```

```
chr17:56774348-56774495
Alignment
Human, Chimp, Rhesus, AluSx consensus, donor
GGGATGATTTGGATAGCAATTTTTGGATGGAAATCATAGTTTTAAGAAAG
GGGATGATTTGGATAACAATTTTTGAATGGAAATCACAGTTTTAAGAAAG
GGGATGATTTACATAACAATTTTTGAGTGGGGATCATAGTTTTAAGAAAG

TTCCGAGGCCACAACTTTCTAGATATGATAGACAGAAAAGTGATTATGAG
TTCCGAGGCCACAACTTTCTAGATATGATAGACAGAAAAGTGATTATGAG
TTCCAAGACCACAGCTTTTTAGAAACTATTGACAGAAAAGTGACTATGAG

ACGTGGACAGGCAGTGGGGGCAATCATGAACTTCGGACAGTGGTTTAGGG
ACGTGGACAGGAAGTGGGGGCAATCATGAACTTAGGACAGTGGTTTAGGG
ATGTGGACAGACAATGGAGTAAATAATGAACTTAGGACAATGGTTTAGGG

CTTGGttcttttgagacagagtctcagtctttcgcc----caggctggagtaca
CTTGGttcttttgagacagagtctcagtctttcgcc----caggctggagtaca
C----ttcttttgaga----tggaatctctttagct----cgggctgga-----
           tgaga----cggagtctcgctctgtcgcccaggctggagtgca

gtggtgtg---atctc--ggctcactgcaacctccacccctcagggttcaagcaa
gtg--gtg---tgatctcggctcactgcaacctccacccccagggttcaagcaa
gtgcagtggcaagatctcggctcactgcaacccctgccccctgggttcaagaga-
gtggcgcg---atctc--ggctcactgcaacctccgcctcccgggttcaagcga

ttctcctgcctcagcctcctgagtagctgggactacaggcatgcaccacc
ttctcctgcctcagcctcctgagtagctgggactacaggcatgcaccacc
ttcttctgcctcagcctcctgagtatctgggactacaggcatgcaccacc
ttctcctgcctcagcctcccgagtagctgggattacaggcgcgcgccacc

acacctggctaattttgtatttttagtacagacgaggtttcacta-------AATC
acacctggctaattttgtatttttagtacagacgaggtttcact
acacctgactaattttgtagtttagtagagatggggtttcactacgttggc----
acgcccggctaattttgtatttttagtagagacggggtttcaccatgttggc----
                                           TA-------ATTC

--TCCATTCAGAAGCTACTCTGGCACCATACACAACGGTGCACAATTGAAGG
----------------------------------------------------
----------------------------------------------------
TCTCCATTCAGAAGCTACTCTGGCACCATACACAACGGTGCACAATTGAAGG

GCTTGTTCTGCTCCTCCACATGGCACCTTTAGAAACGTCACTTCCCCACC
--------------------------------------------------
--------------------------------------------------
GCTTGTTCTGCTCCTCCACATGGCACCTTTAGAAACGTCACTTCCCCACC

TTGTGTGGCAACAGCCTTGGCCAACGTGCTTATGAAGAGCCATGGcaggc
---------------------------------------------caggc
---------------------------------------------caggc
TTGTGTGGCAACAGCCTTGGCCAACGTGCTTATGAAGAGCCATGGCAG

tggtcttgaactcctgacctcaggtgatccacccacatcagcctcccaaa
tggtgtcaaactcctgacctcaggtgatccctccacatcagcctcccaaa
tggtctcgaactcctgacctcaggtgatccgcccgcctcggcctcccaaa

gtgctgggattacaggcatgagccaccgtgcccagcCTAGGGCCTGGTTC
gtgctgggattacaggcgtgagctgccgtgcccagccTAGGGCCTGGTTC
gtgctgggattacaggcgtgagccaccgcgcccggcc

TTAAGAACGCCATTCCCTTCTTGATCCTCAGGCCCAGTGAG
TTAAGCATGTCATTTCCTTCTTGATCCTCAGGCCCAGTGAG
```

Human-specific RDs - donor and acceptor greater than 5 kb from each other or on other chromosome

```
chr21:31838499-31838601
Alignment
Human, chimp, rhesus, AluSx consensus, donor
gagggagatggtctcaaaaaataaataa-----------taaataaataaa--taaaAATAGG
GAGGGAGAcGGTCTCAAAAAATAAATAA-----------TAAATAAATAAA--TAAAAATAGG
    GAGAcGGTCTCAAAAAATAAATAAatatatataaaTAAATAAATAAAttTAAAAATAGG

CCACTTgtggtggctcacgcctgtaatcccagcactttgggaggcccagg
CCACgTGTGGTGGCTCACGCCTGTAATCCCAGCACTTTGGGAGGCCCAGG
CCAggTGcGGTGGCTCACaCCTGTAATCCCAGCACTTTGGGAGGCCCAGG
      gcggtggctcacgcctgtaatcccagcactttgggaggccgagg

taggcagattacatgaggtcaggaTGAAAAGCCCATTTCTTTCTCAAAAG
TAGGCAGATTACATGAGGTC------------------------------
cAGGCAGATcACATGAGGTC------------------------------
cgggcggatcacctgaggtc------------------------------
                        ATGAAAAGCCCATTTCTTTCTCAAAAG

GGCCACGATCCCTCTTGCTGTCCCAGACCACCAGAGCTAACATTTGGTTC
--------------------------------------------------
--------------------------------------------------
--------------------------------------------------
GGCCACGATCCCTCTTGCTGTCCCAGACCACCAGAGCTAACATTTGGTTC

CGGTGAAATGGAAACCTCGAAATGACGAaggagttcaagagcagcctggc
----------------------------AGGAGTTCAAGAGCAGCCTGGC
----------------------------AGGAGTTCAAGAGCAGCCTGGC
----------------------------aggagttcgagaccagcctggc
CGGTGAAATGGAAACCTCGAAATGACGA

caacatggtgaaa------ccccacccctac-aaaaatacaaaaattagccaggcac
CAACATGGTGAAA------CCCCACCCCTAC-AAAAATACAAAAATTAGCCgGGCAC
CAACATGGTGAAActgaaaCCCCAtCCCTAC-AcAAATACAAAAATTAGCCgGGCAC
caacatggtgaaa------ccccgtctctactaaaaatacaaaaattagccgggc--

gatggtgggcgcc---tgtaatcccagctactcaggaggctgaggcaggagaa
GATGGTGGGCGCC---TGTAATCCCAGCTACTCAGGAGGCTGAGGCAGGAGAA
aATGGTGGGCaCC---TGTAATCCCAGCTACTCAGGAGGCTGAGGCAGGAGAA
g-tggtggcgcgcgcctgtaatcccagctactcgggaggctgaggcaggagaa

tcacttgaaccccggtggcaaaggttgcagtcagctgagatcacgccatt
TCACTTGAACCCCGGTGGCAAAGGTTGCAGTCAGCTGAGATCgCGCCATT
TCACTTGAACtCaGGTGGCAAAGGTTGtAGTgAGCTGAGATtgCaCCAcT
tcgcttgaacccgggaggcggaggttgcagtgagccgagatcgcgccact

gcactccagcctgggtaacagagcaagactccgtctcaaaataaataaat
GCACTCCAGCCTGGGTAACAGAGCAAGACTCCGTCTCAAAATAAATAAAT
GCACTCCAGCCTGGGTAACAGAGCAAGACTCCGTCTCAAAATAAATAAAT
gcactccagcctgggcgacagagcgagactccgtctca

aaacaagcaaacaaaCAGGCAGTCTAACAGAGCAGCTGGGCATGgttatg
AAACAAGCAAACAAACAGGCAGTCTAACAGAGCAGCTGGGCATGGTTATG
AAAt----AAACAAACAGGCAGTCTAACAGAGCAGCTGGGCATGGTTATG
```

chr15:84200277-84200858
Alignment
Human, chimp, rhesus, MSTA consensus, donor

```
GATTTTATTATTTTAAAGTGATGAGATAATTG
GATTTTATTATTTTAAAGTGATGAGATAATTG
 ATTTTATTATTTTAAAGTGATGAGATAATTG

AAAATAAAAA-CATTTCTCCAGAAACAAGATTATTGAAATACAAAATTTTC
AAAATAAAAAaCATTTCTCCAGAAACAAGATTATTGAAATACAAAATTTTC
AAAATAAAAA-CATTTCTCCAGAAACAAGATTATTGAAATAtAAAATTTTC

CCAACAATTGTTGAAACCTGGCATCTCTAAAATTATATATAATGCTtgat
CCAACAATTGTTGAAACCTGGCATCTCTAAAAgTATATATAATGCTTGAT
CCcAaAATTGTTGAAACtTGGCATCTCTAAAAaTAcATAcAATGtTTGAT
                                              tgat

atggtttagatctgtgtccctgctgaaatctcatgtcggatagtaatcct
ATGGTTTAGATCTGTGTCCCTGCTcAAATCTCATGTCGGATAGTAATCCT
ATGGTTTAGATCTGTGTCCCTGCTcAAATCTCATGTCaGATAGTAATCCc
atggtttggatctgtgtccccacccaaatctcatgttgaattgtaatccc

cagtgttggaggtggggcctggtgggaggtgattggatcatgggggcaga
CAGTGTTGGAGGTGGGGCCTGGTGGGAGGTGATTGGATCATGGGGGCAGA
CAGTGTTGGAGGTGGGGCCTGGTGGGAGGTGATTGGATCATGGGG-CAGA
cagtgttggaggtggggcctggtgggaggtgattggatcatgggggtgga

tttctcatgaatggtttagcatcattcttcagatgctgttgtcatgataa
TTTCTCATGAATGGTTTAGCATCATTCTTCAGATGtTGTTGTCgTGATAA
TTTCTCATGAATGGTTTAGCAcCATcCTTttGATGCcGTTtTCATGATAg
tttctcatgaatggtttagcaccatccccttggtgctgtcctcgtgatag

tgagtgaattctcacgagatctggtcatttaaaagtatgtagcacctccc
TGAGTGAATTCTCACGAGATCTGGTCATTTAAAAGTgTGTAGCACCTCCC
TGAGTGAATTCTCACcAGATCTGGTCATTTAAAAGTgTGTAGCACCTCCC
tgagtgagttctcgtgagatctggttgtttaaaagtgtgtggcacctccc

tctttcctctc--ttgcttctgctcctaccatgtgagacaactcacgctctc
TCTTTCCTCTC--TTGCTTCTGCTCCTACCATGTGAGACAACTCACGCTCTC
TCTTTCCTCTC--TTGCTTCTGCTCCTACCATGTGAGACAACTCgCcCTCTC
ccctcgctctctcttgctcctgctctggccatgtgacgtgcctgctcccc--

tcctcctttgccttccgccatgattgaa-gtAAAAAAAAAAAAAAAAAGAA
TCCTCCTTTGCCTTCCGCCATGATTGAAAGT--------------------
TCCTCCTTTGCCTTCtGCCgTGATTGAAAGT--------------------
-----cttcgccttccgccatgattgtaagt--------------------
                                                GAA

AAGATGATGTGGTTCCTAGGAGATAGTTAAGTCTTAGCAGGGAACCAAAG
--------------------------------------------------
--------------------------------------------------
--------------------------------------------------
AAGATGATGTGGTTCCTAGGAGATAGTTAAGTCTTAGCAGGGAACCAAAG

ATACTTTCAGTTCTAAATCCAATGTGTGCTGCTGACTCTTACCCTTCCTG
--------------------------------------------------
--------------------------------------------------
--------------------------------------------------
ATACTTTCAGTTCTAAATCCAATGTGTGCTGCTGACTCTTACCCTTCCTG

TTATTCAGAAGAGAGAAGGAGGGAGAGTGCAAAGAAGTTCCGCTGTTGAT
--------------------------------------------------
--------------------------------------------------
--------------------------------------------------
```

TTATTCAGAAGAGAGAAGGAGGGAGAGTGCAAAGAAGTTCCGCTGTTGAT

ACCCGAGCATGACATAGCAGCTAGTGTTTTGCTGAATTGTGATGAAGGAA

ACCCGAGCATGACATAGCAGCTAGTGTTTTGCTGAATTGTGATGAAGGAA

TGTTGACAACCTTTTCAAAACTTACAAGGAAAGGGACTCTGTTGGAGGAA

TGTTGACAACCTTTTCAAAACTTACAAGGAAAGGGACTCTGTTGGAGGAA

CTCAAAGAAAAAAGTATCCATTGAAAGGATAAGTTAGTATTTATATTTTA

CTCAAAGAAAAAAGTATCCATTGAAAGGATAAGTTAGTATTTATATTTTA

GTGCAATTGTGTGGAATTGGTTTGGAAATTTACACACTGTCACTGAGGTG

GTGCAATTGTGTGGAATTGGTTTGGAAATTTACACACTGTCACTGAGGTG

ACAAAAAGAAATGTCTTAATTTTCTGTAATTGTTTTATTTCTCACAGTCT

ACAAAAAGAAATGTCTTAATTTTCTGTAATTGTTTTATTTCTCACAGTCT

TCTACTTGCTCAGTAGACCGAAGTTAGGTTGGAATGGAGAGGACATTGTT

TCTACTTGCTCAGTAGACCGAAGTTAGGTTGGAATGGAGAGGACGTTGTT

TAACCATTAACAATGTCTTTGTCTCCTTCAGTCTATATAGTGTATTTTCC

TAACCATTAACAATGTCTTTGTCTCCTTCAGTCTATATAGTGTATTTTCC

ATATTTAGATATCCTTTAAATAAATGCTAATTTTCACATTTA**AAAAAAAA**

ATATTTAGATATCCTTTAAATAAATGCTAATTTTCACATTTA

AAAAAAAGAAAGAaagttttctgaggcttccccagaagcaga--agctgcta------
-----------------TTTCTGAGGCTTCCCCAGAAGCAGA--AGCTGCTA------
-----------------TTTCTGAGGCTTCCCCAGAAGCAGA--AGCTGCTA------
-----------------ttcctgaggcctccccagaagccgagcagatgccagcacca

tgcttcctgtacagcctgcagcactgtgagccaatttaacctcttttctt
TGCTTCCTGTACAGCCTGCAGaACTGTGAGCCAATTTAACCTCTTTTCTT
TGCTTCCTGTACAGCgTGCAGaACTGTGAGaCAATTaAACCTCTTTTCTT
tgcttcctgtacagcctgcagaaccgtgagccaattaaacctcttttctt

tataaattacccagtctcaggtatttctttataccactgtgagaacggac
TATAAATTACCCAGTCTCAGGTATTTCTTTATACCACTGTGAGAACGGAC

```
TATAAATTACCCAGTCTCAGGTATTTCTTTATACCACTGTGAGAACGGAC
tataaattacccagtctcaggtatttctttatagcaatgcgagaacggac

taatacaATGTTGATGGTGTAATTTTTTTT---CAAATTCTTGATTCTGAAAA
TAATACAATGTTGATGGTGTAATgTTTTTT---CAAATTCTTGATTCTGAAAA
TAAcACAATGTTGATGaggtaatttTTTTTTTTCAAATTCcTGATTCTGAAAA
taataca

ATTAAGAAAAATAGAAACAAATAATT--TTGGCAAATTGCATGAATCTATCC
ATTAAGAAAAATAGAAACAAATAATT--TTGGCAAATTGCATGAATCTATCC
ATTAAGAAAAATAGAAACAAATAATTgtTTGGaAAATTGCATGAgTCTATCC

TGGGTCTTGCCTTTTTT-ACCCTTATACCTTACATTTCTTCCAGTTAGGAA
TGGGTCTTGCCTTTTTTtACCCTTATACCTTACATTTCTTCCAGTTAGGAA
TGGGTtTTGCCtTTTTTTACCCTTgTACCTTACATTTCTTtCAGTTAGaAA

GCTGGGAACCAAGAAAGACACCATCAACCCCACATGTAAGTGGCTATAAA
GtTGGGAACCAAGAAAGACACCATCAACCCCACATGTAAGTGGCTATAAA
GtTGGGAACCAAGAAAGACACCATCAACCCCACATaTAAGTGGCTATAAA

AGAAGTCATGAATGGCAGATGGACATGGGCACTTGTAATAATACATGATG
AGAAGTCATGAATGGCAGATGGACATGGGCACTTGTAATAATACATGATG
AGAAGTCATGAATGGCAGATGGACATGGGCACTTGTAATAAcACATGATG

TAAAAAATTCAAATTATGTTACTGCAGAACAGTTCACACATCAGTG
TAAAAAATTCAAATTATGTTACTGCAGAACAtTTCACACATCAGTG
TAAAAAATTCAAATTATGTTACTGCAGAACAtTTCACACATCAGTG
```

chr8:129173886-129174220
Alignment
Human, chimp, rhesus, MADE1 consensus, donor

```
GGGAAGCTGCAAAGAACCCTTAGGCTCAGAATTTCCTGCTGTCAGCCAGC
GGGAAGCTGCAAAGAACCCTTAGGCTCAGAATTTCCTGCTGTCAGCCAGC
GGGAAGCTGCAAAGAACCCTTAaGCTCAGAATTTCCaGCTGTCAGCaAGt

CCCCCTGCTGTTTCCCATTTATTTTTCTCTATCTCCTTTTCCATCTCCCC
CCCCCTGCTGTTTCCCATTTATcTTTCTCTATCTCCTTTTCCATCTCCCC
CCCCCTGCcGTgTCtCATTTATTTTTCTCTATgTCCTTTTCCATCTCCCC

TTCCAGCACCTTCTGAAACTCCCTAGTCCTGTACTACCAACACACCAACC
TTCCAGCACCTTCTGAAACTCCCTAGTCCTGTACTACCAACACACCAACC
TTCCAGCACCTTCTGAAACTCtCTAGTCtTGTACTACCAACACACCAACC

TTTGCTTAATTGTCACGTCAGACAGATGGCTCAGGTGTCCCCAGCTCCCT
TTTGCTTAATTGTCACGTCAGACAGATGGCTCAGGTGTCCCCAGCTCCCT
TTTGCTTAATTGTCACaTCAGACAGATGGCcCAGGTGTCtCCAGCTCCCT

GAAGCTCTTCCCTTCCCCTCCCCTGGGCAGGGGCCTCAGGCCTTCCTCTG
GAAGCTCTTCCCTTCCCCTCCCCTGGGCAGGGGCCTCAGGCCTTCCTCTG
GAAGCTCTTCCCTTCCCCTtCCCTGGGCAGGGGCCTCAGGCCTTCCTCTG

ACTGATCCAGCCCACCTCCCAGTGTCCATACTATtaggttg----gtgcaaaag
ACTGATCCAGCCCACCTCCCAGTGTCCATACTATTAGGTTG----GTGCAAAAG
ACTGATCCAGCCCACCTCCCAGTGTCCATACTATTAGGTTGcagatcGCA----
                                 ttaggttg----gtgcaaaag

--taa--ttgcag-----------tttttgccattacctttaatggca
--TAA--TTGCAG-----------TTTTTGCCATTAaangtaatggta
ATTAcTTTTGCAgCAACCTAAtaACTTTTGC-----------------

--------------------------------------------------
aaaantgtaattattttgaattaaattaataatatggataatgggaggt
--------------------------------------------------

--------------------------------------------------
gggatggattagttagaggaaggtttgagagatatgattaggggaggga
--------------------------------------------------

-------------------------GCCCCGGCACCCTGGCC
agggaagagattaagggagttgggg-----------------
------------------------------------------
                         GCCCCGGCACCCTGGCC

ATTGGCCCCTGGACAGCTGTTCACTCCCTTCTCAGGCTTGCTGGGGACAC
--------------------------------------------------
--------------------------------------------------
GTTGGCCCCTGGACAGCTGTTCACTCCCTTCTCAGGCTTGTTGGGGACAC

ATACGTCATGCCCTGAAGCGTTCCGGAACCGGTAGGGGCTGCCTGTCCTG
--------------------------------------------------
--------------------------------------------------
ACACGTCATGCCCTGAAGCGTTCCGGAACCGGTAGGGGCTGCCTGTCCTG

CCACTCACCCACTCCAGCTAGGAACTCCTCAGTGGGCAAGATAATTGAGC
--------------------------------------------------
--------------------------------------------------
CCACTCACCCACTCCAGCTAGGAACTCCTCAGTGGGCAAGATAATTGGGC

CCAGGTTGAATTATGACGTTCACAGGTAAGAAGCAGGGTTCAGATGAGGG
--------------------------------------------------
--------------------------------------------------
```

```
CCAGGTTGAGTTATGACGTTCACAGGTAAGAAGCAGGGTTCAGATGAGGG

CTGGGTCTACAGCCAAGTGTGGGAGAGCTGGTGAAAGCCTTGAGAGAGGC
--------------------------------------------------
--------------------------------------------------
CTGGGTCTACAGCCAAGTGTGGGAGAGCTGGTGAAAGCCTTGCGAGAGGC

AGAGACTGGCCAGCCTCACCCTGCATTCCCAATCCCCTCCTCTTTGTCAA
--------------------------------------------------
--------------------------------------------------
AGAGACTGGCCAGCCTCACCCTGCATTCCCAATCCCCTCCTCTTTGTCAA

AATCTTACTCTCAGCATGGtgccattacctttaatggcaaaaactgcaat
--------------------------------AATGGCAAAAACTGCAAT
--------------------------------------------------
---------------------------------------aaaaccgcaat
AATCTTACTCTCAGCATGGTGC

tatttttgcaccaacctaaATACTTTTGCACCGCCAGGAGATCTCCTGCC
TAcTTTTGCACCAACCTAAtaACTTTTGCACCGCCAGGAGATCTCCTGCC
-----------------------------ACaGCCAaGAGATCTtCTGCC
tacttttgcaccaacctaa

ATCTATTCAGTCTTCCATCCCGCTCTCTGGGGCTTTATTCTTTCTGTTCT
ATCTATTCAGTCTTCCATCCCGCTCTCTGGGGCTTTATTCTTTCTGTTCT
ATCTgcTCAGTCTTCCcTCCtGCTCTCTGGGGCTTTATTtTTTCTGTTCT

TTCTGCTTCTTGCTGCGCACGGTGCCCCCACCTCCTGTGGCTGAAATGCT
TTCTGCTTCTTGCTGCcCACGGTGCCCCCACCTCCTGTGGCTGAAATGCT
TTCTGtTTCTTGCTGtcCgtGGTGCCCCCACCTCCTGTGGCTGAAATtCT

GCCCTCTCCCATCCTGCCCAGATGCCTCTTCCACCAAGCTTTTCTGACTG
GCCCTCTCCCATCCTGCCCAGATGCCTCTTCCACCAAGCTTTTCTGACTG
GCCCTCTCCtATCCTGCCCAGATGCCTCTTCCACCAAGCTTTctcaACTG

CCGCATCTGGAATAATATATATATGCAACTTTAAGATCTGTCTTTCTTTT
tCGCATCTGGAATAATATATATATGCAACTTTAAGATCTGTCTTTCTTTT
CCaCATCTGGAATAATActTgTATGCAACTTTAAGATCTcTCTTTCTTTT

TTGATCTTTTC---ATATTTTTTTATTTCTATTTTGGTTATTTGCTGCCATGT
TTGATCTTTTC---ATATTTTTTTATTTCTATTTTGGTTATTTGCTGCCATGT
TTGgTCTTTTCttttcttttcTTTTTTTCTATTTTGGTTATTTGCTGCCATGT
```

```
chr13:81567317-81567414
Alignment
Human, chimp, rhesus, Tigger3b consensus, donor
aatactatgcagtcattaaaaagaatgaaagcatgtcctttgcagcaaca
AcTACTATGCAGTCATTAAAAAGAATGAAAGCATGTCCTTTGCAGCAACA
 ATACTATGtAGTCATTAAAAAGAATGAgAGCATGTCCTTTcCAGCAACA

tggatggagctata--gaccattatcctaagtgatctaagtcaaaaatgaaa
TGGATGGAGCTATA--GACCATTATCCTAAGTGATCTAAGgCAAAAATGAAA
TGGATGGAGCTATAtaGACtATTATCCTAAGcaATCTAAGgCAAAAATGAAA

aaccaaatttcatattttcttacttataaatggaagctaaacaatggaaa
AACCAAATTTCATATTTTCTTACTTATAAATGGAAGCTAAACAATGGAAA
AACCAAATTTCATATTTTCTTACTTATAAATGGAAGCTAAACAATGGAAA

cacatggatacaaagagaa---caacagacactggggcctacttcggggtaga
CACATGGATACAAAGAGAAgaaCAACAGACACTGGGGCCTACTTgGGGGTgGA
CACATGaATACcAAGagaAGAACAACAGACACTGGGGCCTACTTgaGGGTgGA

gaatgggaggagaaagaagg--------------------------
GAATGGGAGGAGAAAGAAGG--------------------------
GAATGGGAGGAGAAAGAAGgttttatttatttatttatttatttat

--------------------------------------------------
--------------------------------------------------
ttttttttttttttgagacagagtctggctctgccgcccaggctggagtg

--------------------------------------------------
--------------------------------------------------
cagtggccggatctcagctcactgcaagctccgcctcccgggttcacgcc

--------------------------------------------------
--------------------------------------------------
attctcctgcctcagcctcccgagtagctgggactacaggcgcccgccac

--------------------------------------------------
--------------------------------------------------
cgcgcccggctagtttttttgtatttttttagcagagacggggtttcaccgt

--------------------------------------------------
--------------------------------------------------
gttagccaggatggtctcgatctcctgacctcgtgatccgcccgtctcgg

-------------------------------------------------------
-------------------------------------------------------
cctcccaaagtgctgggattacaggcttgagccaccgcgcccggccgaaagaagG

ttttaaaaaatacctattgagtactatgct
TTTTAAAAAATACCTATTGAGTACTATGCT
TTTTAAAA-ATACCTATTGgGTgCTATGCT

tattatcagggtgactaaaatatctatacaccaaatccccatgacacata
TATTATCAGGGTGACTAAAATATCTATACACCAAATCCCCATGACACATA
TgTTATCAGGGTGACTgAAATATCTATACACCAAATCCCCATGACACATA

ctttatctacataacaatcttgcacatgtgccctaaacctaaaataaaag
CTTTATtTACATAACAATCTTGCACATGTGCCCTAAACgTAAAAgAAAAG
CTTTATCTAtATAACAATCTTGCACATGTGCCCTAAACCTAAAATAAAAa

ttaaaaaacaaaaacatggtgcccttaaactacactagatttttaaaat
TTAAAAAACAAAAACATGGTGCCCTTAAACTACACTAGATTTTTaAAAAT
TTAAAAAAtAAAAACATtGTGCCCTTAAACTA-----gATTTTTAAAAaT
```

```
atttttccttttcagtagtacattaatcttac---aactttttactctct
ATTTTTTCCTTTTTCAGTAGTACATTAATCTTAC---AACTTTTTTACTCTCT
ATTTTTTCtcTTTTCAGTAGTACATTAATCTTACtacAACTTTTTTACTCTCT

cgctaaatttttttt--actattttattcctttgttataacacttagcttaa
CGCTAAATTTTTTTT--ACTATTTTATTCCTTTGTTATAACACTTAGCTTAA
CcctatattTTTTTTTTACTATTTTATTCCTTTGTaATAACACTTAGCTTAA
                         actcttttgtaataacacttagcttaa

aacacacaattgtggtgctgaacaaatatattttctttctttatattctt
AACACACAATTGTGGTGCTGAACAAATATATTTTCTTTCTTTATATTCTT
AgCACACA-TTGTGGTGCTGtattACAAATgTATTTTCTTTCTTTATATT
aacacaaacacattgtacagctgtacaaaaatatttctttctttatatc

---actgtattgacttttttatattctttaattaattaatt----aacttattttac
-----TGTATTGACTTTTTTATATTCTTtaaTTAATTAATTAATTAACTTATTTTAC
CTcACTGTATTGACTTTTTTcTATTCTTTAATTAATTAATTAA----CTTATTTTAC
ctta-----------ttctataagctttttctattttaaattattttttttttac

ttttta-aaatttt----------------------------------gttaaaagtaagacagaaacacacatattagctta
TTTTTA-AAATTTTT---------------------------------GTTAAAAGTAAGACAGAAACACACATATTAGCTTA
TTTTTA-cATTTTTtaaaaatttaatatttacttttaaaaatttgGTTAAAAGTAAGACAGAAACACACAcATTAcgTTA
tttttaaacttttt----------------------------------gttaaaactaagacacaaacacacacattagccta

ggcctacactgtgttaggatcatcaatatccctgtcttcTGACGGATTTC
GGCCTACACaGTGTTAGGATCATCAATATCCCTGTCTTCc----------
GGCCTACACaGTGTTAGcgTCATCA-TATCCCTGTCTTCT----------
ggcctacacagggtcaggatcatcaatatcactgtcttcc----------
                                        GACGGATTTC

CACTCTATTGTGATGAAAAGATTTGAAAGATCTTTTTTTGAACTTCCAAG
--------------------------------------------------
--------------------------------------------------
--------------------------------------------------
CACTCTATTGTGATGAAAAGATTTGAAAGATCTTTTTTTGAACTTCCAAG

AAATTTCATTTACTATTTAAAAAAAACAAAAAAAAAAAtccctgtcttctacctctgcatctccagaggc---------------
--------------------------------------------------AgCTCTGCATCT-CAGAGGC---------------
--------------------------------------------------atctcaaCATCTCCAGAGGC---------------
--------------------------------------------------acctccacatct--------tgtcccactggaagg
AAATTTCATTTACTATTT

-----------aataccacgcatggagtagtcatctctatg----
-----------AATACCACGCATGGAGTAGTCATCTCTAcG----
-----------AATACCgCaCATGGAGTAGTCATCTCTATG----
tcttcaggggcaataacacgcatggagctgtcatctcctatgata

acaacaatgacttctggaatacctcttgaaggacatgcctggggaggttt------
ACAACAATGACTTCTGGAATACCTCTTGAAGGACATGCCTGGGGAGGTTT------
ACAACAAaGACTTCTGGAATACCTCTTGAAGGACATGCCTGaGGAtGTTT------
acaatgccttcttctggaatacctcctgaaggacctgcctgaggctgttttacagt

tatagttaacatc-----------t-ataagtagaaagagtacactc-aaaataacaataaaa
TATAGTTAACATC-----------T-ATAAGTAGAAAGAGTACACTC-AAAATAACAATAtAA
TATtGTTAACATC-----------T-ATAAGTAGAAgGAGTACACTC-AAAATAACAATAAAA
ta-----------acttttttttttaataagtagaaggagtacactctaaaataacgataaaa

tgtacagtataataaatacataaaccagtaacatagttgtt-at---cattatt
TGTACAGTATAATAAATACATAAACCAGTAACATAGTTGTT-AT---CATTATT
TGTACAGTATAATAAATACATAAACCAGTAACATAGTTGTT-AT---CgTTATT
agtatagtatagtaaatacataaaccagtaacatagtcgtttattatcattatc

aaatattacatactgcacataattgtatgtgctatacctttataagactg
```

```
AAATATTACATACTGCACATAATTGTATGTGCTATACCTTTATAAGACTG
AAATATTACATACTGCACATAATTGTATGTGCTATACCTTTATAAGACTG
aagtattatgtactgtacataattgtatgtgctatactttatacgactg

gtggcacagtaggttcatttacatcattatcaccacaaacacaggagtaa
GTGGCACAGTAGGTTCATTTACATCATTATCACCACAAACACAGGAGTAA
GTGGCACAGTAGGTTCATTTACATCATTATCACCACAAACACAGGAGTAA
gcagcgcagtaggtttgtttacaccagcatcaccacaaacacgtgagtaa

tgtgttgcactatgac-ctaatgcagctacaatgtcaccaggcaatagaaa
TGTGTTGCACTATGAC-CTAATGCAGCTACAATGTCACCAGGCAATAGAAA
TGTGTTGCACTATaAC-aTAtgaCAGCcACAATGTCACCAGGCAATAGAAA
tgcgttgcgctacgacgttacgatggctacgacgtcactaggcgataggaa

tttttcagttccattataatcttatgggagcagagacttatatgtggtgc
TTTTTCAGTTCCATTATAATCTTATGGGAGCAGAGACTTATATGTGGTGC
TTTTTCAGTTCCATTATAATCTTATGGGAtCAGAGAtTTAcATGTGGTGC
tttttcagctccattataatcttatgggaccaccgtcgtatatgcggtcc

atcattgacaaaaacgtgggtatgtggtgcatgactgTACATATTAAAAT
ATCATTGACAAAAACGTGGGTATGTGGTGCATGACTGTACATATTAAAAT
ATCATcGAtAAAAACGTGGGTATGTGGTGCATGACTGTACATATTAAAAT
gtcgttgaccgaaacgtcgttatgcggcgcatgactg

TATTAGTTTTTCGGAAACTACAATTTAATAGCAAACTAATTCAAAATAGT
TATTAGTTTTTCaGAAACTACAATTTAATAGCAAACTAATTCAAAATAGT
TATTAGTcTTTTGGAAAtTACAATTTAATAGtAtAtTAATTCAAAATAGT

AACTAAACAAATCAATGATCTAAAACATATTTGTTTTTATGGTAATATTA
AACTAAACAAATCAATGATCTAAAACATATTTGTTTTTATGGTAATATTA
AACaAAACAAATCAATcATCTAAAACATATTTcTTTTTATGGTAATATTA

GTTAAACAAAGGAAG---AAAATGTTGGACCTATGTGACAATATGTTTGATTT
GTTAAACAAAGGAAG---AAAATGTTGGACCTATGTGACAATATGTTTGATTT
GTTAAACAAAGGAAGtacAAAATGTTGGgCCTATGTGAtAATATGTTTGATTT

GTCAAAACTGATGTTTCTTCTGGACATTATATTCGATGGTTGGAC
GTCAAAACTGATGTTTCTTCTGGACATTATATTCGATGGTTGGAC
GTCAAAACTGATGTTTCTTCTGGACATTATATTtGATGGcTGGAC
```

Hominoid-specific RDs - donor and acceptor within 5 kb of each other

chr14:87413740-87413941
Alignment
Human, chimp, rhesus, donor

TCTGTTTTTTAAAAAGCTTCAAATTagccaggcacggtggctcacacctg
TCTGTTTTTTAAAAAGCTTCAAATTAGCCAGGCACGGTGGCTCACACCTG
TCTGTTTcTTAAAAAGCTTCAAATTAGCCAGGCACGGTGGCTCACACCTG

taatcccagcactttgggaggccaaggcaagtggatcacttcagctcagg
TAATCCCAGCACTTTGGGAGGCCAAGGCAAGaGGATCACTTCAGCTCAGG
TAATCCCAGCACTTTGGGAGGCCAAGGCgAGTGGAcCACTTCAGCcCAGG

agtgaacaacatggtgggcaacatggtgaaaccctgtctctacaaaaaat
AGTGAACAACATGGTGGGCAACATGGTGAAACCCTGTCTCTACAAAAAAT
--------ACATGGTGGGCAACATGGTGAAACCCTGTgTCTACAAAAAAT

GCTTCCATCTTAAATGGAAGATGAGTGGCCATCAGATAGAAGCAGTAAGC
GCTTCCATCTTAAATGGAAGATGAGTaGCCATCAGATAGAAGCAGTAAGC

      ATCTTAAAGGGAAGATGAGTGGCCATCAGATAGAAGCAGTAAGC

ATGGTGACACGT-AAAGAGAAGCAGCAGTAAGACTGGACAGGTCCCCTATG
ATGGTGACACGT-AAAGAGAAGCAGCAGTAAGACTGGACAGGTCCCCTATG

ATGGTGACATGTCAAAGAGAAGCAGCAGTAAGACTGGACAGGTCCCCTATG

TAAAATGCACTAGAAGATTCCAAAAAAAATTTGTATTTGACTATCTCTCTG
TAAAATGCACTAGAAGATTCCAAAAAAAATTTtTATTTGACTATCTCTCTG

TAAAATGCACTAGAGGATTTCAAAAAAATGTTTATTTGACTATCTCTCTG

ATGATAAAAAAGGGAGACCTCAAAACTACACATGAACAAAGTCATGTTTG
ATGATAAAAAAGGGAGACCTCAAAACTACACATGAACAAAGTCATGTTTG

ATGATAAAAAAGGGAGACCTCAAAACTACACATGAACAAAGTCATGTTTG

TGG--***aagaatt***agctgggcatggtggcacatgcctatagtctcagctactc
TGG--***AAGAATT***AGCTGGGCATGGTGGCACATGCCTATAGTCTCAGCTACTC
---gc***AAaAATT***AGCTGGGCATGGTGGCACATGCCTgTAGTCTCAGCTAtTC
TGG--***AGGAATT***

aggaggctgaggtgggaggatcacttgagccctggaagtcaagactgcag
AGGAGGCTGAGGTGGGAGGATCACTTGAGCCCTGGAAGTCAAGACTGCAG
AGGAaGCTGAGGTGGGAGGATCACTTtAGCCCTGGAAGTCAAGgtTGCAG

tgagtcaacatcacaccactgcactccagcctgggtgatagagtgcgact
TGAGTCAAgATCACACCACTGCACTCCAGCCTGGGTGATAGAGTGaGACT
TGAGTCAAggTCACACCACTGCACTCCAGtCTGGGTGAcAGAaTGaGACT

ctgtctcaaaaataaagaaataaaTAAGT----CTCCAAATAAATAGCATGTAA
CTGTCTCAAAAATAAAGAAATAAATAAGT----CTCCAAATAAATAGCATGTAA
CTGTCTCgAAAATAAAtAAATAAATAAaTaaacCTCCAAATAAATAGaATGTAA

CATAATCTTTGTAGGTGATTAAGCTCATCGCAATTTCAGCGTACCCATTC
CATAATCTTTGTAGGTGATTAAGCTCATCGCAATTTCAGCGTACCCATTC
CATAATCTcTGTAGGTGATTAAGtgCATCtCAATTTCAGCGTACCCATTC

chr16:57787578-57787730
Alignment
Human, chimp, rhesus, L1ME3 consensus, donor

```
aTTTGTACATCAAGTGGGATCAATAAAAATATTTGGCattagttttgcct
ATTTGTACATCAAGTGGGATCAATAAAAATATTTGGCATTAGTTTTGCCT
ATTTGTACATCAAGTGGGATCAgTgAAAATATTTGGCgTTAGTTTTGCCT
                                     attagttttgcct

gttcttggagttcaatataaatggaatc-t-caatatgtctattttATATGG
GTTCTTGGAGTTCAATATAAATGGAATC-T-CAATATGTCTATTTTATATGG
GTTCTTGGAGTTCAATATAAATGGAATC-T-CA-------------------
gttcttgaacttc-atataaatggaatcatacagtatg--tactctt-----
                                     GTCTATTTTATATGG

GACATATTAAACCCCCTTC-ATTCCCCCCCGCCACAATGTCTATTTCCTTT
GACATATTAAACCCCCTTC-ATTCCCCCCCGCCACAATGTCTATTTCCTTT
---------------------------------------------------
---------------------------------------------------
GACATATTAAACCCCTCTCCATTCCCCCCCCCCACAATGTCTATTTCCTTT

TTAGATTTGTCCTTATAAAATTTGAATAAATGAACTCCCTATTAAACAGG
TTAGATTTGTCCTTATAAAATTTGAATAAATGAACTCCCTATTAAACAGG
--------------------------------------------------
--------------------------------------------------
TTAGCTTTGTCCATATAAAATTTGAATAAATGAACTCCCTATTAAACAGG

TGGACATTATTACTTTTCACTCAGAAGATATATTTTAAAAGAGAAACA---ta
TGGACATTATTACTTTTCACTCAGAAGATATATTTTAAAAGAGAAACA---TA
------------------------------------------------TAGTA
-----------------------------------------------------
TGGACATTATTACTTTTCACTCAGAAGATATATTTTAAAAGAGAAACA---T

tgtagttctttgtttctgacttctctgaaaacaaagtttttaagatttat
TGTAGTTCTTTGTTTCTGACTTCTCTGAAAACAAAGTTTTTgAGATTTAT
TGTAGTTCTTTGTTTCTGACTcaTCTGAAAAtAAAGTTTTTgAGATTTAT
ttgcg-tc-tggcttct---ttcgctcaacataatgtctntgagatt

ttgtgttgctgtgtgtatcaatagtttgctagtttttttttttttttt
TTGTGTTGCTGTGTGTATCAATAGTTTGCTAGTTTTTTTTTTTTTTTT
aTGTGTTGCTGTGTGTATCAATAGTTTGCTAGaTTTT

tttttttccgagacagagtct
TTT-----CCGAGACgGAGTCT
```

```
chr16:27304337-27304412
Alignment
Human, chimp, rhesus, AluSq consensus, donor
taataatcatactagtctagtaaaCAGCAACAATTATAAGTACCtttatt
TAATAATCATACTAGTCTAGTAAACAGCAACAAcTATAAGTACCTTTATT
TAATAATCATACTAGTCTgGTAgCAGCAACAAcTgTAAGTAtCtcttttt

tttt-------agatggactttgctcttatcacccaggctggagtgtagtggcacg
TTTT-------AGATGGACTTTTGCTCTTATCACCCAGGCTGGAGTGTAGTGGCACG
tTTTtTTTTTTtGAcGGAgTTTTGCTCTTgTCgCCCAGGtTGGAGTGcAGTGGtgCG
            gacggagtttcgctcttgttgcccaggctggagtgcagtggcgcg

atttcagatcactgcaacctccacctcccgggttcaagcaattgtcctgc
ATTTCAGATCACTGCAACCTCCACCTCCCGGGTTCAAGCAATTcTCCTGC
ATTTCAGATCACTGCAACCTCCACCTCtgGGGTTCAAGCAATTcTCCTGC
atctcggctcactgcaacctccgcctcccgggttcaagcgattctcctgc

ctcagcctccccaggagctgggaatacaggcatgtgccaccacgcccggc
CTCAGCCTCCCCAGGAGCTGGGAATAtAGGCATGTGCCACCACGCCCGGC
CTCAGCCTCCtgAGtAaCTGtGAtTACAGGCgcccGCCACCACGCCCGGC
ctcagcctcccgagtagctgggattacaggcgcccgccaccacgcccggc

taatttttgtatttttagtacagatggggtttcaccatgttggccaggat
TAATTTTTGTATTTTTAGTACAGATGGGGTTTCACCATGTTGGCCAGGAT
TAATTTTTGTAgTTTTAGTAgAGATGGGGTTTCACCATGTTGGCCAGGAT
taatttttgtatttttagtagagacggggtttcaccatgttggccaggct

ggtctcaaattcctgatcttaggtCTCGGCCTAAGCAATCATTTGTCTCT
GGTCTCAAATTCCTtATCTTAGGTCTCGGCCTAAGCAATCATTTGTCTCT
GGTCTtgAAcTCCTGAcCTTAG----------------------------
ggtctcgaactcctgacctcag----------------------------
                               TCAGCAATCATTTGTCTCT

AGACTTCTCATTCCATAAGGGAATTAGCTCTTAAGTGTTTAAGAGCTTAAG
AGACTTCTCATTCCATAAGGGAATTAGCTCTTAAGTGTTTAAGAGCTTAAG
---------------------------------------------------
---------------------------------------------------
AGACTTCTCGTTCCATAAAGGAATTAGCTCTTAAGTGTTTAAG

-----------gcctcggccttccaaagtgctgggattacaggcgtgagccaccatgccc
-----------GCCTtGGCCTTCCAAgGTGCTGGGATTACAGGCGTGAGCCACCATGCCC
ATgATcTGcCTGCCTCGaCCTcCCAAAGTGCTGGaATTACAGGCaTaAGCCACCATGCCt
gtgatccacccgcctcggcctcccaaagtgctgggattacaggcgtgagccaccgcgccc

ggcAGTACCTCTTATACTTAGTACA
GGCAGTACCTCTTATACTTAGTACA
GGCAGTACCTCTTATACTTAGTACA
ggc
```

```
chr18:46082474-46082986
Alignment
Human, chimp, rhesus, MER54A consensus, donor
CTACGCATCAGTGTATGAGCTGTGCTTACATTGAAAGTCAAAAATCCCTC
CTACGCATCAGTGTATGAGCTGTGCTTACATTGAAAGTCAAAAATCCCTC
CTACGCATCAGTGTATGAGCTGTtCTTACATTGAAAGTgAAAAcTCCCTC

TTGTGTGTCTAACACTGCTGCCTTGTATCGAGAAAGATGAAACCACTATC
TTGTGTGTCTAACACgGCTGCCTTGTATCGAGAAAGATGAAACagacAGa
TTGTGTGTCTAACACTGCTGCCTTGTATCGAGAAAGATGAAA-TACTATC

TCAttgtattcctctctaatttggtcttgaggtctgtctttggagaatgg
------------------------cagaGAGGTCTGTCTTTGGAGAATGG
TCATTGTATTCCTCTCTAATTTaGTCTTGAGGTCTcTCTTTGGAGAgTGG

ccataaaccataatactgccttagtgggggtccagggaattttttttttt
CCtTAAACCATAATACTGCCTTAGTGGGGGTCCAGGGAAttTTTTTTTTT
CCATAAACCcTAAcACTGCCTTAGTaGGGGTCCAGGGAATtgTTTTTTTT

--tagaggcagggtctccctatgttgcccaggctggtcttgagctcctgggc
TTTAGAGGCAGGGTCTCCCTATGTTGCCCAGGCTGGTCTTGAGCTCCTGGGC
TTTAGAGGCAaGGTCTCaCTATGTTGCCCAGGCTGGTCTTGAGCcCCTGGGt

tcaagcagtcctcccacctcggcctcccaaagtgctgagattacagacct
TCAAGCAGTCCTCCCACCTCGGCCTCCCAAAGTGCTGAGATTACAGACCT
TCAAGCAaTCCTCCCACCTCaGCCTCCCAAAcTtCTGAtATTACAGgCCT

gaggcactgcacctgtcgtctctggggaatttggtcatgtacgtttacag
GAGGCACTGCACCTGTCGTCTCTGGGGAATTTGGTCATGTACGTTTACAG
GAGcCACTGCACCTGTCcTgTCTGGGGAATTTGGTCATGgACGTTTgCAG
                              tggtcgtggatgtttacag

tgtgcctttcacatgctactttttttatccaggtggatgatccaatgccta
TGTGCCTTTCACATGCTACTTTTTTATCCAGGTGGATGATCCAATGCCTA
TagGCCTTTCACATGCTACTTTTTTATCCAGGTGGATGgcCCAATGCCTA
tgtgcctttcacgggatacttctttatcctggcggacggcctaatgccta

agtgtctagccACTTCCTCTTGGCACTGAGAAGCTGAATCTGCGCGATCC
AGTGTCTAGCCACTTCCTCTTGGCACTGAGAAGCTGAATCTGtGCGATCC
AGTGaCTgaCC---------------------------------------
agtgtccgacc---------------------------------------
     CTAGCCACTTCCTCTTGGCACTGAGAAGCTGAATCTGCGCGATCC

TCAGATCAACCTCGGTCTCTCTGGCTGGATATTTATTGGGTTCTGCCTGA
TCAGATCAACCTCGGTCTCTCTGGCTGGATATTTATcGGGTTCTGCCTGA
--------------------------------------------------
--------------------------------------------------
TCACATCAACCTCGGTCTCTCTGGCTGGATATTTATCGGGTTCTGCCTGA

AAGGCCGTCTAACGCCTTTCCTCTTGGAAAAGTCGCAGGTCTTAA---TACAG
AAGGCCGTCTAACGCCTTTCCTCTTGGAAAAGTCGCAGGTCTTAA---TACAG
-----------------------------------------------------
-----------------------------------------------------
AAGGCCGTCTAACGCCTTTCCTCTTGGAAAAGTCGCAGGTCTTAATAATACAG

CTTCAGTTTATACGTTTCTTTGTGCCTTATAATAGTCATAAAGGTCTGGT
CTTCAGTTTATACGTTTCTTTGTGCCTTATAATAGTCATAAAGGTCTGGT
--------------------------------------------------
--------------------------------------------------
CTTCAGTTTATACGTTTCTTTGTGCCTTATAATAGTCATAAAGGTCTGGT

TAGATCTCCACTCCCTCCAGGAGCGCTCCAGGAGCTCACGGTTGGGCCTT
TAGATCTCCACTCCCTCCAGGAGCGCTCCAGGAGCTCACGGTTGGGCCTT
```

```
--------------------------------------------------
--------------------------------------------------
TAGCTCTCCACTCCGTCGAGGAGCGCTCCAGGAGCTCACAGTTGGGCCTT

TCATAGCACGTAGGGAAAGATCCTTGCTATTTTGCTATTTATTGTACTGG
TCATAGCACGTAGGGAAAGATCCTTGCTATTTTGCTATTTATTGTACTGG
--------------------------------------------------
--------------------------------------------------
TCGTAGCACTTAGGGAAAGATCCTTGCTATTTTGCTATTTATTGTACTGG

CTTAAAATGGGGGTGGGCAGGCAGGAGCGCCCTTTTCGGAGCGGGCGGGA
CTTAAAATGGGGGTGGGCAGGCAGGAtCGCCCTTTTCGGAGCGGGCGGGA
--------------------------------------------------
--------------------------------------------------
CTTAAAATGGGGGTGGGCAGGCAGGAGCGCCCTTTTCGGAGCGGGCGGGA

AAGGGCGGCGACCACGGTTGGCCCCCCGCAGACCCCGCAGCTCCTGCTGT
AAGGGCGGCGACCACGGTTGGCCCCCCaCAGACCCCGCAGCTCCTGCTGT
--------------------------------------------------
--------------------------------------------------
AAGGGCGGCGACCACGGTTGGCCCCCCGCAGACCCCGCAACTCCTGCTGT

GCCCCTCAGCTGCCATTAGAGTCCCCATGGGGTCCCTCCTGCCTGTGTCT
GCCCCTCAGCTGCCATTAGAGTCCCCATGGGGTCCCTCCTGCCTGTGTCT
--------------------------------------------------
--------------------------------------------------
GCCCCTCAGCTGCCATTAGAGTCCCCATGGGGTCCCTCCTGCCTGTGTCT

CCACAAGAGGGACCCCATGGGGACTCTAATGAGAGCCCTGCATCCTACCA
CCACAAGAGGGACCCCATGGGGACTCTAATGAGtGCCCTGCATCCTACCA
--------------------------------------------------
--------------------------------------------------
CCACAAGAGGGACCCCATGGGAACTCTACTGAGAGCCCTGCATCCTACCA

AGGAGCCCCCAGAAGTCGGGCCCTGcgtgtccaagtgtccatctcacagg
AGGAGCCCCCAGAAGTCGGGCCCTGCGTGTCCAAGTGTCCATCTCACAGG
-------------------------tgagaCCAAGTaTCCATCTCAtAGG
-------------------------cgtgaccaggtgtccctctcacagg
AGGAGCCCCCAGAAGTCGGGCCCTGCGTG

aaacttat----gttggcagatgctcttgtggctcttgtctgacctgtgtccag
AAACttgtTTATGTTGGCAGATGCTCTTGTGGCTCTTGTCTGACCTGTGTCCAG
AAACTTATttatGTTGGCAGgTGCTCTTGTGGtTCTTGTCTGACCTGTGTCCAG
aaacttgtttatactggcagacgcccttgtggctcttgtctgacctgtgtccag

tttatttctaccaagatagccaatc--taggagagctctgaccaagagaaaa
TTTATTTCTACCAAGATAGCCAATC--TAGGAGAGCTCTGACCAAGAGAAAA
TTTATTTCTACCAtGATAGCCAgTC--TAGGAGAGCTCTGACCAAGAGAAA-
tttattcctaccaagatagccactctctaggagagccctgaccgggaggaga

gtcaggttcaagtgtgccagtagaatggacagaggcaacaaaacccatga
GTCAGGTTCAAGTGTGCCAGTAGAATGGACAGAGGCAACAAAACCCATGA
CTtAGGTTCAAGTGTGCCAGTgGAATGGACAGAGGCAACAAAACCCATGA

aatagcagaagcattttcttacagcaccaaagatgagaagagtaaaaatg
AATAGCAGAAGCATTTTCTTACAaCACCAAAGATGAGAAGAGTAAAAATG
AATAGCAGAAGCATTTTCTTACAGCACCAAAGATGAGAgGAGTAAAAATG

agggccagtaggaaggttgcaggtacaatatgctcaacc--------gggggccagac
AGGGCCAGTAGGAAGGTTGCAGGTACAATATGCTCAACC--------GGGGGCCAGAC
AGGGCCAGTAGGAAGGTcGCAGGgACAATAcaCTCAACCaacaagttGGGGGCCAGAg

agaaagatgaacccatgggccaaggcctttgtttggggtccagggtgttc
```

```
AGAAAGATGAACCCATGGGCCAAGGCCTTTGTTTGGGGTCCAGGGTGTTC
AGAAAGATGAACCCATGGGCCAAGGCCTTTGTT-GGGGTCCAGGGTGTTg

ttacccaagcaggtctccttcagagagttctaagtggtgggtttagagca
TTACCCAAGCAGGTCTCCTTCAGAGAGTTCTAAGTGGTGGGTTTAGAGCA
TTACCCAAGCAGGTCTtCTTCAGAGAGTTCTAAGTGGTGGGTTTAGAGCA

agcaggcatgcatcccatggggtcatgctgtgactgagaag---tcactgtgg
AGCAGGCATGCATCCCATGGGGTCATGCTGTGACTGAGAAG---TCACTGTGG
AGCAGGCATGCATCCCAaGGGGTtATGCTGTGACTGAGAtgtGGTCACTGTGG

catatctgtgtagtctgtgtggcatgtggggtcagtggggccagttgggc
CATATCTGTGTAGTCTGTGTGGCATGTGGGGTCAGTGGGGCCAGTTGGGC
CATATCTGTGTAGTCTGTGTGGCATGTGGGGTCAGTGGGGCCAGcTGGGC

cagtttagtgggttgtatctagctgccttgtggagaggtggtaaccaaga
CAGTcTAGTGGGTTGTATCTAGCTGCCTTGTGGAGAGGTGGTAACCAAGA
CAGTcTAGTGGGTTGTATCTAGCTGCCccaTGGAGAcaTGGTcACCAgGA

gccagttgtataaggtagatatgtggattaagaacattgagaaactagga
GCCAGTTGTATAAGGTAGATATGTGGATTAAGAACATTGAGAAACTAGGA
GCCAGTTGTATAAaGcAGATATcTGGATTAAGAACATTcAGAAACTAGGA

ataggcagaaagctggaaactgtgtccagggtgactaagccagcttctgg
ATAGGCAGAAAGCTGGAAACTGTGTCCAGGGTGACTAAGCCAGCTTCTGG
gTAGGCAGAAAGCTaGAAACTGTaTCCAGGGTGACTAAGCCAGCTTCTGa

aatgagaaagttaaacctactttcaaaacagatgcagaagtgacatgata
AATGAGAAAGTTAAACCTACTTTCAAAACAGATGCAGAAGTGACATGATA
AATGAGAAAGTTAAACCTACTTTCAAAACgGATGCAGAAGTGACATGATA

ggaattcattacaTCCATGGTCAGAACTAAATGTATTTTATAGTTCATTT
GGAATTCATTACATCCATGGTCAGAACTAAATGTATTTTATAGTTCATTT
GGAATTCATTACATCCATtGTT-GAACTAAATGTATTTTATAGTTCATTT

ATTTTAGTTTACATGAAGTCTACTTATATTAGCATacaaatattcatagt
ATTTTAGTTTACATGAAaTCTACTTATATTAGCATACAAATATTCATAGT
ATTTTAGTTTACATGAAaTCTACTTATATTAGCATACgAATATTCATAGT

tatgtaacaaccaccccactcaagacacagaactct
TATGTAACAACCACCCCACTCAAGACACAGAAtTCT
TAcGTAACAACCACCaCACTCAAGACACAGAACTCT
```

```
chr1:234348661-234348870
Alignment
Human, chimp, rhesus, AluSq consensus, donor
AATCTTGTTTTCCAATCTCTCTTAGAAATTATCTCCTCCCCAGGTTTCCA
AATCTTGTTTTCCAATCTCTCTTAGAAATTATCTCCTCCCCAGGTTTCCA
aatcttgctttccaatctctctcagaaattatctcctccccaggtttcca

TAATACTCTCTGTCTTGAGTTTCCTACTACTAATTCTTGGGTGTTTTCTT
TAATACTCTCTGTCTTGAGTTTCCTACTACTAATTCTTGGGTGTTTTCTT
taacactctctctc--gagtttcctactactaattcttgggtgttttctt

GTCCTTTTCTTTTCATGTTTTTCTGTTTGAttg----ttttgtttctttttgtt
GTtCTTTTCTTTTCATGTTTTTCTGTTTGtttgATTGTTTTGTTTCTTTTTGTT
gttcttttcttttcatgttttTCTGTTTATTTGAttgttttgtttctttctgtt

gttttgagacggagtctcgctctgttt-cctaggctggacagcagttgctt
GTTTTGAGACGGAGTCTCGCTCTGTTT-CCTAGGCTGGAgAGCAGTTGCTT
tttttgagctggagtctcgctctgttt-cctaggctgga------------
    tgagacggagtttcgctcttgttgcccaggctgga------------
                                               GCTT

tatcctagAACTTATGATGTAGCTGCCTGTGTGCCCATCTGTCTCCACAA
TATCCTAGAACTTATGATGTAGtTGCCTGTGTGCCCATCTGTCTCCACAA
--------------------------------------------------
--------------------------------------------------
TATCCTAGAACATATGATGTAGTTGCCCGTGTGCCCATCTGTCTCCACAA

CAGAGCAAGGTCCTGGAAGCAGAGGTCGGGGGCGTGGGGGAGGTCTTGCT
CAGAGCAAGGTCCTGGAAGCAGgaGTCGGGG-CGTGGGGGAGGTCTTGCT
--------------------------------------------------
--------------------------------------------------
CAGAGCAAGGTCCTGGAAGCAGGGGTAGGGG-CGTCGGGGAGGTCTTGCT

TGTCCTCTTTATGTCTCAGTGCTTAGTGTACCTTATATTTGGACAATGAA
TGTCCTCTTTATGTCTCAGTGCTTAGTGTACCTTATATTTGGACAATGAA
--------------------------------------------------
--------------------------------------------------
TGTCCTCTTTATGTCTCAGTGCTTAGTGTACCTTATATTTGGACAATGAA

TAAATAAATGTGCGCTGCTCTAGAAAAGAGATCATTGTCTCAATGGAGTG
TAAATAAATGTGCGCTGCcCTAGAAAAGAGATCATTGTCTCgATGGAGTG
--------------------------------------------------
--------------------------------------------------
TAAATAAATGTGCGCTGCTCTAGAAAAGAGATCATTGTCTCGATGGAGTG

AAAATCAGTAAGGGGTAGG--------------tctcggctcactgcaacctccacctccctgg
AAAATCAGTAAGGGGTAGG--------------TCTCGGCTCACTGCAACCTCCAtCTCCCTGG
-------------------gtgcgatggtgggatctcggctcactgcaacctccacctccctgg
-------------------gtgcagtggcgcgatctcggctcactgcaacctccgcctcccggg
AAAATCAGTAAGGGG

ttcaagtaattctcctgcctcagtctcccgagtagctgggattacaagcg-
TTCAAGTgATTCTCCTGCCTCAGTCTCCCGAGTAGCTGGGATTACAAGCG-
ttcaagtgattctcccgcttcagtctcccgagtagctgggattacaagtgc
ttcaagcgattctcctgcctcagcctcccgagtagctgggattacaggcgc

ctcaccaacccgccaggctaaatttttgtatttttagtaagagatggggt
CcCACCAACCCGCCAGGCTAAATTTTTGTATTTTTAGTAAGAGATGGGGT
ccaccaacccgccagggtaacttttttgtatttttagtaagagatgggtt
ccgccaccacgcccggctaa--tttttgtatttttagta-gagacggggt

ttcaccacattggccaggctggccttgaactcctgacctcagttgatctg
TTCACCACgTTGGCCAGGCTGGCCTcGAACTCCTGACCTCAGTTGATCTG
```

ttcgtcatgttggccaggctggcctcgaactcctgacctcaggtgatctg
ttcaccatgttggccaggctggtctcgaactcctgacctcaggtgatcca

cgggcctcggcctcccaaagtgctgggattacaggcgtaagccactgtgc
CGGGCCTCGGCCTCCCAAAGTGCTGGGATTACAGGCGTAAGCCACTGTGC
cgggcctcggcctcccaaagtgttgggattacaggtgtaagccactgtcc
cccgcctcggcctcccaaagtgctgggattacaggcgtgagccaccgcgc

ctggccCCttttcatgtttttatgttttgagacggagtctcgctctgtca
CTGGCCtCTTTTCATGTTTTTATGTTTTGAGACGGAGTCTCGCTCTGTCA
ctggccTC
ccggcc

```
chr1:5680589-5680737
Alignment
Human, chimp, rhesus, consensus, donor
attacctaAGATACACTAttttttttttttttttttttttgagacggagtt
ATTACCTAAGATAtACTATTTTTTTTTTTT---------GAGACGGAGTT
ATTACCTAAGATAtatatataTTTTTTT-----------GAGAtGGAGTT

ttgctctttttgcccaggctggagtgcaatcacacgatcttggctcaccg
TTGCTCTTTTTGCCCAGGCTGGAGTGCAATCACACGATCTTGGCTCACCG
TTGCTtTTTTTGCCCAGGtTGGAGTGCAATgACAtcATCTTGGCTCACCa

caacctccacctcccgggttcaactgattctcctgcctcagcctcccgag
CAACCTCCACCTCCCGGGTTCAACTGATTCTCCTGCCTCAGCCTCCCGAG
CAACCTCCgCCTCCCGGGTTCAACTGATTCTCCTGCCTCgGCCTCCCGAG

tagctgagattacaggtatgcgccaccacacctggccaattCCATTCCAT
TAGCTGAGATTACAGGTATGCGCCACCACACCTGGCCAATTCCATTCCAT
TAGCTGAGATTACAGccATGCaCCACCACACCcaGCCAATT---------

TCCTAAAGATCAGCGAAGCACTTTAATCTCCCAGGCCTCTGGCTCAGCTG
TCCTAAAGATCAGCGAAGCACTTaAATCTCCCAGGCCTCTGGCTCAGCTG
--------------------------------------------------
          CAGCAAAGCACTTTAATCTCCCAGGCCTCTGGCTCAGCTG

TTTCTGAGCAGAGAACTCCACACATCCTGAGGAAGGCCAAGTCTGGGGAA
TTTCTGAGCAGAGAACTCCACAtATCCTGAGGAAGGCCAAGTCTGGGGAA
--------------------------------------------------
TTTCTGAGCAGAGAACTCCACACATCCTGAGGAAGGCCAAGTCCAGGGCA

GGAAGGAAGATCAGCAGATGGGGGGAAGCCCTGCAGTCTCC---------tagtagaga
GGAAGGAAGATCAGCAGATGGGGGGAAGCCCTGtAGTCTCC---------TAGTAGAGA
-----------------------------------------ttgtatttcTAGTAGAGA
GGAAGGAAGATCAGCAGATGGGGGGAAGCCCTGCAG

cggggtttctccatgttgatcaggctgttctcgaactcccgacctcaggt
CGGGGTTTCTCCATGTTGATCAGGCcGTTCTCGAACTCCCGACCTCAGGT
tGGGGTTTCTCCATGTTGATCAGGCTGgTCTCGAACTCC-GACCTCAGGT

gatctgcctgcctcagcctcctaaagtgctgagatcacaggcatgagcca
GATCTGCCTGCCTCAGCCTCCTAAAGTGCTGAGATCACAGGCATGAGCCA
GATCcaCCgGCCTtgGCCTCtTAAAGTGCTGgGATCACAGGtgTGAGCCA

ccgcgcccggccACCTAAGATGTTTTATTATAGCAGCACAGGCAAACGAA
CCGCGCCCaGCCACCTAAGgTGTTTTATTATAGCAGCACAGGCAAACGAA
CCGtGCCtGGCCACCTAAGgTaTTTTATTATAGCAGCACAGGCAgACaAA

GGCAGTCTACAGGGCAGAATTCTGACAACTTCC
GGCAGTCTACAGGGCAGAATTCTGACAACTTCC
GGCAGTC
```

```
chr10:74929662-74929767
Alignment
Human, Chimp, Rhesus, MER20 consensus, donor
TGATCTCTGAGATCTAATGTGGAATGCCTTTTCTTATACATTTATtcaac
TGATCTCTGAGATCTAATGTGGAATGCCTTTTCTTATACATTTATTCAAC
                                             tcaac

tttggcactcttggtatcttgggct-gataattctttgttggggttgggga
TTTGGCACTgTTaaTATCTTGGGCT-GATAATTCTTTGTTGGGGTTGGGaA
cttggcactatcgacattttgggccggataattctttgttgtgg--ggg--

gagactgccctgtgcattgtgtgatTTATTAATGAAAAATACTTTGCAAA
GAGACTGCCCTGTGCATTGTGTGAc-------------------------
----ctgtcctgtgcattgtaggat-------------------------
                      GATTTATTAATGAAAAATACTTTACAAA

AACAGTATGTTTGGCCTAAAATAGTTCAGCTGACTCTGAGGGTTTACAAC
--------------------------------------------------
--------------------------------------------------
AACAGTATGTTTGGCCTAAAATAGTTCAGCTGACTCTGAGGGTTTACATT


AACAACTGAGCAAGTGACAATAATGGCTGTGCtgttgagga---------catccctgg
-----------------------------------------atttagcagCATCCCTGG
-----------------------------------------gtttagcagcatccctgg
GACGACTGAGCAAGTGGCAATAATGGCTGTGTTATTGAGGA---------CA

cctctacccactagataccggtggtacatcccctttatccattgagaacc
CCTCTACCCACTAGATACCGGTaGTACATCCCCTTTATCCgcTGAGAACC
cctctacccactagatgccagtagcatccccccccccagttgtgacaacc

aCTGCCTCAAGCTTGTACATCCAGTGCATGAGGGGAAAAAAGAGAACATC
ACTGCCTCAAGCTTGgACATCCAGcGCATGAGGGGAAAAAAGAGAACATC
a
```

```
chr11:59044173-59044241
Alignment
Human, chimp, rhesus, L1PA16 consensus, donor
CAAGAAGATAGGCCAAGAAGACAATCCTCATAGAAAGCAACTCTCACTTA
CAAGAAGgTtGGCCAAGAAGACAATCCTCATAGAAAGCAACTCTCACTTA
AAAGAAGATTGGCCAAGAAGACAATCGTCATAGAAGGCAACTCTCACTTA

TTCCCAAACTTTTCATGGGCTTGTtgtaaacaagttcgattaatgcagaa
TTCCCAAACTTTTCATGGGCTTGTTGTAAACAAGTTCGATTAATGCAGAA
TTCCCAAACTTTTCATGGGCTTGTTGTAAACAAGTTTTATTAATGCAGAA

gctcctttagcaatgaatgttctccaaaactaccatttgacgcagcaatc
GCTCCTTTAGCAATGAATGTTCTCCAAAACTACCATTTGACGtAGCAATC
GCTCCTTTAGCAATGAATGTTCTCcaaaactaccatttgacacagcaatc
                        cagaactaccatttgacccagcaatc

ccattgctgggtatatacccaaaagaaaataaattattctaccaaaaaga
CCATTGCTGGGTATATACCCAAAAGAAAATAAATTATTCTcCCAAAAAGA
ccattgctgggtatatacccaaaagaaaataaattattctaacaaaaaga
ccattactgggtatatacccaaaggaaaataaatcattctaccaaaaaga

cacatgcacctgtatgttca---cag------------tagcaaagacaaggaatcagcctaggt
CACATGCAC--------------------------------------------------------
cacatgcacctgtatgttcg---cag------------tagcaaagaccaggaatgagcctaggt
cacatgcactcgtatgttcattgcagcactattcacaatagcaaagacatggaatcaacctaggt

gcccatcaatggtggactagatgaagaaaatgtgatacattatacaccat
--------------------------------------------------
ggccatcaacggtggactggatgaagaaaatgtgatacattatataccat
gcccatcaatggtggactggataaagaaaatgtggtaca-tatacaccat

ggggtactatgcagacattttaaaaatgaattcatgtgctttacagtaa
--------------------------------------------CAGcAA
ggagtactatgcagccattttaaaaatgaatttttgtcctttacagcaa
ggaatactacgcagccataaaaaagaatgaaatcatgtcctttgcagcaa

catggatgcagctggaggccgtgatcctaagcaaattaatgcaggaacaa
CATGGATGCAGCTGGAGGCCaTGATCCTAAGCAAATTAATGCAGGAACAA
catggatgcagctggaggccatgatcctaagcaaattaatgcaggaacaa
catggatgcagctggaggccattatcctaagcgaattaacgcaggaacag

aaaatcgaa--taccacatgttctcacttatgggtgagagctaaccatcgat
AAAATCatAatTACCACATGTTCTCACTTATaGGTGAGAGCTAACCATtGAT
aaaatcaaa--taccacatgttctcacttatacgtgaaagctaaccattgat
aaaaccaaa--taccgcatgttctcacttataagtgggagctaaacattggg

ttacacatggacacaaagatgggaacaaaagacactgaggcctacttgag
TTACACATGGACACAAAGATGGGAACAAtAGACACTGAGGCCTACTTGAG
ttacacatggacacaaagatgggaacaatagacactggggcctacttgag
-tacacatggacataaagatgggaacaatagacactggggactactagag

gggaggagggtgggaagagtgtgagg-tcagaaaactacctattaggtact
GGGAGGAGGGTGGGAAGAGTGTGAGG-TCAaAAAACTACgTATTAGGTACT
gggaggagggtgggaagagtatgagggtcaaaaaactacctattaggtact
gggggaggga-gggaggagggcaagggttgaaaaactacctattgggtact

atgctcactaccaagatTGTCTTCTTGGCCAATCTTCTTTGGGCACTCTA
ATGCTCACTACCAcGATTGTCTTCTTGGCCAATCTTCTTTGGGCACTCTA
atgctcacta----------------------------------------
atgctcacta----------------------------------------
              GATTGTCTTCTTGGCCTATCTTCTTGGGGCACTCTA

CCCTAATGTTCTATCTCATGGAACCCTGCCCTTCTAGtcactacctgggt
```

```
CCCTAATGTTCTgTCTCATGGAACCCTGCCCTTCTAGTCACTACCTGGGT
-------------------------------------------tctgggt
-------------------------------------------cctgggt
CCCTAATGTTCTATCTCATGGAACCCTGCCCTTCTAGTC

gacaaaatcatttgtacaccaaaccccagcaacacacaatttactcatgt
GACAAAATCATTTGTACACCAAACCCCAGCAACACACAATTTACTCATGT
gacaaaatcatttgtacaccaaaccccagcaacacacaatttactcatgt
gatgggatcattcgtaccccaaacctcagcatcacgcaatatacccatgt

aacaagcctacacatgcacccgttgaacctaaaataaaagttaaacaaaa
AACAAGCCTACACATGCACCCGcTGAACCTAAAATAAAAGTTAAACAAAA
aacaagcctacacatgtacccactgaacctaaaataaaagttaaacaaaa
aacaaacctgcacatgtaccccctgaatctaaaataaaagttgaaaaaaa

aTGAAATCAATGTTATCTTCTTTGCTGATTGACATTTCCTGTGTTCTATT
ATGAAATCAATGTTATCTTCTTTGCTGATTGACATTTCCTGTGTTCTATT
aTGAAACCAATGTTCTCTTCTTTGCTGATTGACATTCCCTGTGTTCTATT
a
```

chr12:59216664-59216740
Alignment
Human, chimp, rhesus, AluJb consensus, donor
ACATTCTTCATATTAGTGTAAATTAAAATCTTCCTCTTggcgtg-----gtggct
ACATTCTTCATATTAGTGTAAATTAAAATCTTCCTCTTggcgta-----gtggct
ACATTCTTCgTATTAGTGTAAATTAAAATCTTCCTCTTGGCttggtGTGGcGGCT
ggccgggcgcggtggct

cacacctataatcccagcactttaggaggctgaggtgggcagaccactta
cacacctataatcccagcactttaggaggctgaggtgggcagaccactta
CACACCTATAATCCCAGCACTTTAGGAGGCTGAGGTGGGCAGAtCACTTA
cacgcctgtaatcccagcactttgggaggccgaggcgggaggatcacttg

agctctggagttccagatcagcctggacaacatggagaagccccacctct
agctctggagttccagatcagcctggacaacatggagaagccccacctct
AGCcCTGGAGTTCCAGATCAGCCTGGACAACATGGAGAAGCCCCAtCTCT
agcccaggagttcgagaccagcctgggcaacatggtgaaaccccgtctct

acaaaacatatacacacacacagaaaa-----gttagccggacatggcagcatgc
acaaaacatgtacacacacacagaaaa-----gttagccggacatggcagcatgc
ACAcAAaATACaaaaaAaAaAaAaAaAAAAaTTAGCCaGACATGGCAGCATGC
acaaaaaatacaaaaa-----------------ttagccgggcgtggtggcgcgc

acctgtagttccagctactagagagactgaggT**CTCTACCAAGAGACTG**A
acctgtagttccagctactagagagactgaggTCTCTACCAAGAGACTGA
ACCTGTAGTTCCAGCTACTAGAGAGACTGAGGT-----------------
gcctgtagtcccagctactcgggaggctgaggc-----------------
A

GTTCTACCAAGTAAAATAGAATTGCAGCGTCTCTATTTGGGTGGGATATG
GTTCTACCAAGTAAAATAGAATTGCAGCGTCTCTATTTGGGTGGGATATG

GTTCTACCAAGTAAAATAGAATTGCAGCTTCTCTATTTGGGTGGGATATG

ACTTACATTT----***gga***tcacttgagtctgggaggtcaaggatgaagtgagctg
ACTTACATTT----***gga***tcgcttgagtctgggaggtcaaggatgaagtgagctg
----------GAGA***GGA***TCACTTGAGTCTGGGAGGTCAAGGcTGAAGTaAGCTG
----------agga***gga***tcgcttgagcccgggaggtcgaggctgcagtgagccg
ACTTACACTT----***GGA***

tgatcatgccactgcacacccacctgggtgacagagtgagaccctgtctc
tgatcatgccactgcacacccacctgggtgacagagtgagaacctgtctc
TGATCAcaCCACTGCACAtCCACCTGGGTGACAGAGTGAGACCCTGTCTC
tgatcgcgccactgcactccagcctgggcgacagagcgagaccctgtctc

aggaaaaaaaaaaaTCTTCCTCTTAAAATTATATGTGGCAGG
aggaaaaaaaaaa-TCTTCCTCTTAAAATTATATGTGGCAGG
AGGAAAgAAAAAA-TCTTCtTCTTAAAATTATATGTaGCAGG
a

```
chr13:100373056-100373198
Alignment
Human, chimp, rhesus, AluJo consensus, donor
CTGACATTCATCAACATTAACAAATATCACTGAAAACACCATTGCACACT
CTGACATTCATCAACATTAACAAATATCACTGAAAACACCATTGCACACT
AgGACAcgCATCAgCATTAACAAATgTCACTGAAAACACCAcTGCACACT

TGGCTTTCCACAGtttttttttttt--caaagaccagctctctc--tgttgccc
TGGCTTTCCACAGTTTTTTTTTTTTt-CAAAGACCAGCTCTCTC--TGTTGCCC
TGaCTTTCCACAaTTTTTTTTTTTTtcCAAAGACCcaGtCTtTCTCTGTTGCCC
                            tgagacagggtctcgctctgtcgccc

aagctggagtgcagttgtgtaatcatatctcactgcagcttTATACAAAT
AAGCTGGAGTGCAGTTGTGTAATCATATCTCACTGCAGCTTTATACAAAT
AgGCTGGAGTGCAGTgGTGTAATCgTATCTCAgTGCAGCcT--------
aggctggagtgcagtggcgcgatcatagctcactgcagcct---------
                                     GCTT--------T

GAATGACGTAACAACGAAATCACAAATGATAAATGGTTTTCAAAA-TCAAA
GAATGACGTAACAACtAAATCACAAATGATAAATGGTTTTCAAAA-TCAAA
---------------------------------------------------
---------------------------------------------------
GAATGACGTAACAACTAAATCACAAGTGATAAATGGTTTTTAACAATCAAA

AAA-TTTAACATCATACTATACATCTGGCATTCATGCTGGTGATTTGAAGG
AAA-TTTAACATCATACTATACATCTGGCATTCATGCTGGTGATTTGAAGG
---------------------------------------------------
---------------------------------------------------
AAAATTTAACATCATATTATACATCTGGCATTCATGCTAGTGATTTGAAGG

TGCATGACCCTTCTGAATTGTTTGTATAAAGCAG---Gctcctgggctcccac
TGCATGACCCTTCTGAATTGTTTGTATAAAGCAG---GCTCCTGGGCTCCCAC
----------------------------------TGACCTCCTGGGCTCCCAC
----------------------------------cgaactcctgggctcaagc
TGCATGACCCTTCTGAATTGTTTGTATAAAGTAG

aatcctccaatcgtcctgcctcagcttctt-agtagttgaaacta----catgtg
AATCCTCCAATCGTCCTGCCTCAGCTTCTT-AGTAGcTGAAACTA----CATGTG
AATCCTCCAgTCcTCCTGaCTCAGCcTCTcCAGcAGcTGAAACTAcaggCATGTG
gatcctcc

acaccatgcctggctaatgttttttagaattttttttttttttttttttt
ACACCATGCCTGGCTAATGTgTTTTAGAATTTTTTTTTTTTTTTTTTTT-
ACACCATGCCTGcCTAgTaTTTTTTAGA-TTTTTTgTTgTTgTTgTT---

tttgtagagacctagtttcactatgttgcccagactggtctcaaactcct
---GTAGAGACCTAGTTTCACTATGTTGCCCAGACTGGTCTtAAACTCCT
--TGTAGAGACCTAGTTTCACTATGTTGCCCAGACTGaTCTCAAACTCCT

ggactcaagcaaccctcctgccttggcctcccaaagtATCAATATTTTAA
GGgCTCAAGCAACCCCC-TGCCTTGGCCTCCCAAAcTATCAATATTTTAA
GGgaTCcAtCAACCCTCCTGCCTTGGCCTCCCAcAGTATCAATATTTTAA

ATAAAGACCAAACCAAAGGCTTTTGT
ATAAAGACCAAACCAAAGGCTTTTGT
ATAAAGACCAAACCAAAGGCTTTTGT
```

chr16:13932912-13933203
Alignment
Human, chimp, rhesus, Charlie1a consensus, donor

```
AGTGCCAAAGACTGAAGAGCAGAAGAGATTT-TAGGGAAATATACATATAC
AGTGCCAAAGACTGAAGAGCAGAAGAGATTTgTAGGGAAATATACATATAC
AGTGCCAAcGACTGAAGAGCAGAAGAGATTTgTAGGGgAATATACATATAC

AGCTGGCCACCATCTCGTTTGGCTACCACTGTTTTGGCCTCT-GTCACTGT
AGCTGGCCACCATCTtGTTTGGCTACCACTGTTTTGGCCTCTtGTCACTGT
AGCTGGaCACCATCTtGTTTtGCTAtCACTGcTTTGGCCTCT-GcCACTGT

TTGACCAGACcagcggttttcaaagtgtggtccgtgaacccctggggtca
TTGACCAGACCAGCGGTTTTCAAAGTGTGGTCCaTGAAgCCCTGGGGTCA
TTGACCAGACCAGCGGTTTcCAAAGTGTGGTCtGTGAA-CCCTGGGGTCA
                                            gggtcg

agatttaatacagttaataattctgatggttctttagggacattcttaag
AGATTTAATACAGTTAATAATTCTGATGcTTCTTTAGGGACATTCTTAAG
AGATTTAATACAGTTAATAATTtTGATGcTTCTTTAGGGACATTCTTAAG
agatttaataaaattaatatttttactgcttcatcaaggacattcttaag

tga---tg----------aactgc-agaagcatgacagtaaagaatgcactgaatgccagtaca
TGA---TG----------AACTGC-AGAAGCATGACAGTAAAGAATGCAtTGAATGCCAGTACA
TGA---TG----------AACTGC-AGAAGCATGACAGTAAAGAATGCAtTGAATGCCAGTACA
tgaaactggcatttttttnactgcgag-cgcgtggcggtgaagaatacaatgactactagtaca

ctttcatggcactgccttggttcatgctacggcaccagcaTGTACAATTA
CTTTCATGGCACTGCCTTGGTTCATGCTACaGCACCAGCATGTACAATTA
CTTTCATGGCACTGCCTTGGTTCgTGCTACGGCACCAGCA----------
gtttggtgccactgccttgatccgtgctaaggcgccagca----------
                                      CATGTACAATTA

TGGAGCATTCTTATGTAAAACTCTTCTACCTGTAGATATCCAAATGTTGT
TGGAGCATTCTTATGTAAAACTCTTCTACCTGTAGATATCCAAATGTTGT
--------------------------------------------------
--------------------------------------------------
TGGAGCATTCTTATGTAAAACTCTTCTACCTGTAGACATCCAAGTGTTGT

CATTTCAAAGTAAAAAATGTTATTTATTTTCCTCTTGATTATAGTTAAAA
CATTTCAAAGTAAAAAATGTTATTTATTTTCCTCTTGATTATAGTTAAAA
--------------------------------------------------
--------------------------------------------------
CATTTCAAAGTAAAAAAAGTTATTTATTTTCTTCTTGATTATAGTTAAAA

TAAGAGATAACCACTTACTATATAACTGCAAAAGTCAGAGTGATGCTTAT
TAAGAGATAACCACTTACTATATAACTGCAAAAGTtAGTGATGCTTATAT
--------------------------------------------------
--------------------------------------------------
TAAGAGATAATCACTTAC--TATAACTGCAAAAGTCAGAGTGATGCTTAT

ATGCCAATCCACATATTTTCCTATAAATTATTACTTTCCAGTAAACCAAA
--GCCAATCCACATATTTTaCTATAAATTATTACTTTCCAGTAAACCAAA
--------------------------------------------------
--------------------------------------------------
ATGCCAATCCACATACTTTACTATAAATTATTACTTTCCAGTAAACCAAA

GAAGCTGTGTTTTAAA-TGACCTCCAATTTTATTCTTAAATAACAGCAAAT
GAAGCTaTGTTTTAAA-TGACCTCCAATTTTATTCTTAAATAACAGCAAAT
---------------------------------------------------
---------------------------------------------------
GAAGCTATGTTTTTAAGTGACCTCCAATTTTATTCTTAAATAACAGCAAAT

TCCTATTAACAACATTGCAAATATTAAAATAAC-------------ttgcttttacgccatca
```

TCCTATTAACAACATTGCAAATATTAAAATAAC-------------***TTGC***TTTTACaCCATCA
---------------------------------GTATTACCCAGCA***TTGC***TTTTACaCCATCA
---------------------------------gttttacccacca***ttgc***ttttgcaccatca
TCCTATTAACAACATTGCAAATATTAAAATAAC-------------***TTGC***

ctggaaatgtcaacgcagtgaaaaaagtcaaataatgtcatggtattgtt
CTGGAAATGTCAACaCAGTGAAAAAAGTCAAATAATGTCATaGTATTGTT
CTGGAAATGTCAACaCAGTGAAAAAAGTCAAATAATGTCATaGTATTGTT
gtgcaaatgtcaacacagtgaaaaa-ggcaaataacgtcttagtattatt

acgagaaaagttctgacatcacatagccc-tgaaaatatcttgggggcccc
ACGAGAAAAGTTtTGACATCACATAGCCC-TGAAAAcATCTTGGGGaCCCC
AtGAGAAAAGTTtTGACATCACAcAGCCC-TGAgAAcATCTTGGGGaCCCC
atgaaaatagttttgacctcgcggaccccctgaaagggtctcggggaccct

tgggagttcacggaccacacccagaaaaccactgGTCCAGCTGAACAGCG
TGGGAGTTtAtGGACCACACCCAGAAAACCACTGGTCtAGCTGAACAGCG
cGGGAGTTCAtGGACCACACCCAGAAAACCACTGGTCtAGCcGAACAGtG
caggggtccgcggaccacactttgagaaccgctg

ACAGATCACAGTAGTGGGAGGCGGTGTGGTTGGTAGGAAGACAGGATGAC
ACAGATCACAGTAGTGGGAGGaGGTGTGGTTGGTAGGAAGACAGGATaAC
AgAGATCACAaTAGTGGGAGGaGGTaTGaTTGGTAGGAAaACAGGATGgt

AGCCAGTTACGTATGTAGGTCATGTGACCATCAGAGACTGTTTTAAATTA
AGCCAGTTACaTATGTAGGTCATGTGACCATCAGAGACTGTTTTAAATTA
AGCtgGTTACaTATGTAGGTCATGTGACCATCAGAGACTaTaTTAAATTA

ACCATAAATTGATGGCACTTTTT
ACCATAAATTGATGGCACTTTTT
ACCgTAAATTGATGGCACTTTTT

chr2:226988215-226988397
Alignment
Human, chimp, rhesus, MLT1A1 consensus, donor

TATTTTTCTTATt-actgtggtttaaatgt----gtctcctccaaaattcaggag
TATTTTTCTTATT-gCTGTGGTTTAAATGT----GTCTCCTCCAAA-TTCAGGAG
TAtTTTTTCTTATTgCTGTGGTTTAAATGT----GTCTCCTCCAAAATTtAGGAG
                tatggtttgaatgttttgtcccctccaaaattcatgtg

ttg-----------ccaatgtgatattttaagaagtgggacattt-aagaagtgattagat
TTG-----------CCAATGTGATATTTTAAGAAGTGGGACATTT-AAGAAGTGATTAGAT
TTG-----------tCAATGTGAcAgTTTTAAGAAGTGGGACATTT-AAGAgGTGATTAGgc
ttgaaacttaatkgccaatgtracagtattaagaggtggggcctttargaggtgattaggt

tttgagggatcctcacttatgaatgtgattaaagcccttataaaagagac
TTTGAGGGATCCTCACTTATGAATGgGATTAAAGCCCTTATAAAAGAGgC
TTTGAGGGATCCTCACTTATGAATGgGATTAAAGCCCTTATAAAAGAGgC
catgagggctcctccctcatgaatgggattaatgcccttataaaaagggc

tttatgcagt---ttttggttaccttgcccttctgc-cttctgccatatgagga
TTTATGCAGT---aTTTGGTTACCTTGCCCTTCTGC-CTTCTGCCATATGAGGA
TTTATGCAcc---aTTTGGTTAgCTTGCCtTTCTGC-CTTCTGCCgTATGAGGA
ttgatggagtgggttctctctctctctcttctgctcttctgccatgtgagga

ttcagcaataaggTG**GAACAGCAC**AAAGAAATACTAATCCCTGCACCCTT
TTCAGCAATAAGGTGGAACAGtACAAAGAAATACTAATCCCTGCAtCCTT
TgCAGCAATAAGGTG-----------------------------------
cacag---
------------------------AAAGAAATA-TTATCCCTGCATCCTT

GCACTGCATTCATCATTGCATGGATTCTGTTCCTAATAATGAATACTTTC
GCACTGCATTCATCATTGCATGGATTCTGTTCCTAATAATGAATACTTTC

GCACTGCATTCATCATTGCATGGATTCTGTTCCTAATAACGAATAATTTC

TTTTCTAATTTGGTTGACACAGACACCATGAGCTTGCTATTGATTTTCCG
TTTTCTAATTTGGTTGACACAGACACCATGAGCTTGCTATTGATTTTCCG

TTTTCTAATTTGGTTGACACAGACACCATGAGCTTACTATTGATTTTCCG

GAAGTGGACATTTTCTCCTTCCACCCACTGCTG***TTCATCTTG***TAAGTtca
GAAGTGGACATTTTCTCCTTCCACCCAgTGCTG***TTCATCTTG***TAAGTTCA
---------------------------------***cTgATCTTG***TAAGTTCA
---tca
GAAGTGGATGTTTTCTCCTTCCACCCAGTGCTG***TTCAAATT***

ccagacaactgaactcgccagaacccagatcttggacttctcagcctcca
CCAGACAACTGAAtgtGCCAGAACCCgGATCTTGGACTTCTCAGCCTCCA
CCAGACAACTGAACTtGCCAGcACCCgGATCTTGGACTTCTCAGCCTCCA
ccagacaccaaacct-gctggnaccttgatcttggacttcccagcctcca

gaactgtgagaaaataaatttctggccaggtgcagtggctcacacctgta
GAACTGTGAGAAAATAAATTTCTGGCCAGtTGCAGTGGCTCACACCTGTA
GAACTGTaAGAAAATAAATTTCTGGCCAGGTGCAGTGGCTCACACCTGTA
gaactgtgagaaa-taaatttct

attccagcac
ATTCCAGCAC
ATTCCAGCAG

```
chr3:190518240-190518621
Alignment
Human, chimp, rhesus, MER20 consensus, donor
tagttccaacttaagaccc-ctctgcctgatcctaTACTTCCTTTAGTTca
TAGTTCCAACTTAAGACCAgCTCTGCCTtATCCTATACTTCCTTTAGTTCA
TcaTTCCAACTTAAGACCAgCTCgGCCTGATCCTATACTTCCTTTAGcTCA
                                                 ca

gtggttctcaagagagggtgattttccc-atgggggacatatgacaatgt
GTGGTTCTCAAGAGAGGGTGATTTTgCCC-gTGGGGGACATATGACAATGT
GTGGTTCTCAAGAcAGGGTGATTTTgCCC-ATGGGGGACATtTaACAATGT
gtggttctcaaccgggggtgattttgccccccaggggacatttggcaatgt

cccagtgtcccagtgagatattatcggttgtcataTCAAATGTATACTTA
CCCAGTGTCCCAaTGAGATATTATtGGTTGTCATATCAAATGTATACTTA
CCCAGTGTCCCAGTGAGATATTACtGGTTGTCAcATC-------------
            ctggagacattttggttgtcacaac-------------
                                                TTA

CAAAGG--------------TATATAAAATGTTCTCTGATAAAGACTAATTCAGTTTAGCTAAG
CAAAGGcatacttacaaaggTATATAAAATGTTCTCcGATAAAGACTAATTCAGTTTAGCTAAG
----------------------------------------------------------------
----------------------------------------------------------------
CAAAGG--------------TATACAAAATGTTCTCCGATAAAGACTAATTCAGTTTAGCTAAG

TTTAAAGTGCACTTGTATGTGTGTGCATATATCTGCATGTGTTCAGAGAG
TTTAAAGTGCACTTGTGTGTGTG--CATATATCTGCgTGTGTTCAGAGAG
--------------------------------------------------
--------------------------------------------------
TTTAAAGTGCACTTGTGTGTGTGTGCACATATGTGCATATGTTCAGAGAG

GAGGGTTGAGGCATGGGATAGGATGAGGATGGAAATTACGATGGGTAGAA
GAGGGTTGgGGgATGGGATAGGATGAGGATGGAAATTgCtATGGGTAGAA
--------------------------------------------------
--------------------------------------------------
GAGGGTTGGGGGATGGGATAGGATGAGGATGGAAATTGCTATGGGTAGAA

ACCTCTCTTGTGCAAGGAAAAAGGAGCTACTGCCTCTTCTTATTTGTCTC
ACCTCTCTTGTGCAAGaAAAAAGGAGCTACTGCCTCTTCTTATTTGTCTC
--------------------------------------------------
--------------------------------------------------
ACCTCTCTTGTGCAAGAAAAAAGAAGCTACTGCCTCTTCTTATTTGTCTC

TACACACTTGAATTTATAGTCACAGGGTCTCTATGAGGATAATTCCAGGA
TACACACTTGAATTTATAGTCACcGGGTCTCTgTGAGGATAATTCCAGGA
--------------------------------------------------
--------------------------------------------------
TATGCACTTGAATTTATAGTCACAGGGGCTCTATGAGGATAATTCCAGGA

AAAAATATGCAAGGATGATATATACAGTCTGTCCAGCAGCGTGACTTCTC
AAAAcTATGCAAGGATGATATATACAGTCTGTCCAGCAGtGTGACTTCTC
--------------------------------------------------
--------------------------------------------------
AAAA-TGTGCAAAGATGATATATACAGTCTGTCCAGCAGTGTGACTTCTC

AAGTGATTTCAAGTACTGGGTATTGGACGGGCGTGCCCTCTATTACAGGA
AAGTGATTTCAAGTACTGGGTATTGGAtGGGCGTGCCCTCTgTgACAGGA
--------------------------------------------------
--------------------------------------------------
AAGTGATTTCAAGTACTGGGTATTGGATGGGCGTGCCCTCTATGACAGGA

GACTTCCTTCTATACACAgggaggtat------------ctg-t-ctggtatctggtgggagga
GACTTCCTTCcATACACAGGGAGGTAT------------CTG-T-CTGGTATCTGGTGGGtGGA
```

```
---------------------------tggggtggaagcCTG-T-CTGGTATCTGGTGGGtGaA
---------------------------tgggggggggratgctactggcatctagtgggtaga
GACTT

ggatagggaggctgcttaacatcctaacatgcagaagacattctctgcca
GGATAGGGAGGCTGCTTgACATCCTAACATGCAGAAGACATTCTCTGCCA
GGATAGGGAGGCTGaTTAACATCCTAACATGCAGgAGACcTTCTCTcCCA
ggccagggatgctgctaaacatcctacaatgcacaggacagcccccacaa

tagcaaaagattgtctggcctaaaatagcaatagtcccaaggtgaagaaa
TAGCAAAAGATTGTCTGGCCTAAAATAGCAATAGTCCCAAGGTGAAGAAA
TAGCAAAAaATTGTCTGGCCTAAAATAGCAATAGTCCCAAGGTGAAGAAA
---caaagaattatccggcccaaaatgtcgatagtgccaaggttgagaaa

ctttgGTTTAGCTGTACAGACAGCCACATTTGTGACTCTACTTCATAGAA
CTTTGGTTTAGCTGTACAGACAGCCACATTTGTGACTCTACTTCATAGAA
CgTTGtTTTAGCTGTACAGACAGCCACATTTGTGACTCTACTgCATAGAA
ccctg

AACCAGATGAAA
AACCAGATGAAA
AACCAGATGAgA
```

```
chr3:49720849-49721014
Alignment
Human, Chimp, Rhesus, L1MB2 consensus, donor
cccgggttcaagcagttctcctggattatagatgtgctaccgcgcccagc
CCtGGGTTCAAGCAcTTCTCCTGGATTATAGgcGTGCcACtGtGCCtAGC

tacttttgtatttttggtagagacagggtttcactttgttggcgtggc
TAaTTTTTGTATTTTTTaGTAGAGACAGGGTTTtgCTaTGTTGGCaTGGC

tggtctcaaactcctggcctcatgtgatcctgccacctcggcctcccaaa
TGGTCTCgAACTCCTGGCCTCgTGTGATCCTcCCACCTCGGCCTCCCAAA

gtgctggggttacaggcgtgcgccactgtgcccggtcacccatcatagcc
GTGCTaGGGTTACAGGCGTGCGtCACcGTGCCCGGcCACCCATgATAGCC

atttttaagtgttaagtctattcacattgtcgtgcagccaatctccacac
ATTTTTAAGTGTTAAGTCTgTTCACATTGTCGTaCAGCCAATCTCCAgAC
       agtgttaagtatattcacattgttgtgcaaccgatctccagaa

tcatttcatcttgcaaaactgatactccgtgcctgttgaacaaaacctcc---
TCATTTCATCTTGCAAAACTGATACTCtGTGCCcGTTaAACAgAACCTCC---
ctttttcatcttgcaaaactgaaactctatacccattaaacaacaactcccca

tgtcctcccttccccagcttccctgacagctcccaCCCATCCCCACCTGT
TGTtCTCCCcTCCCCAG---------------------------------
tttccccctcccccag---------------------------------
               AGCTTCCCTGACAGCCCCCACCCACCCCGACCTGT

CAGCAGATCCCTGGGTCTGGCAGCTGGGGGAGCAGTGGTTCTGGCATCAG
--------------------------------------------------
--------------------------------------------------
CAGCAGATCCCTGGGTCTGGCAGCTGGGGGAGCAGCGGTTCTGGCATCAG

GTCCCTGAGTGCTCTGGCCAGCGGCTCACAGACCCCCAGCTGGGCCTTAC
--------------------------------------------------
--------------------------------------------------
GTCCCTGGGTGCTCTGGCCAGCGGCTCACAGACCCCCAGCTGGGCCTCAC

TTCCATGTCATGAGGGGACCACCATGGCCCTGTTCTGAGGCTAGCTCAGG
--------------------------------------------------
--------------------------------------------------
TTCCATGTCATGAGGGGACCACCATGGCCTTGTTCTGAGGCTGGCTCAGG

TTgccccctggcaaccaccatcctactttctgtctct--gaatttgactatac
---CCCCTGGCAACCACCATCCTACTTTCTGTCTCT--GAATTTGACTATcC
---cccctggcaaccaccattctactttctgtctctatgaatttgactactc
GT

taagcacctcatctaggaagaatcatacactatttgttcttttgtgtatg
TAAGCACCTCATCTAGGgAGAATCATACACTATTcGTTtTTTTGTGTcTG
taggtacctcatataagtggaatcatacagtatttgtctttttgtgactg

gctttagcataatgtcctcaaggttcattcgtgttatagcatgtgtcaga
GCTTTAGCATAATGTCCTCAAGcTTCATTtGTGTTATAGCATGTGTCAGA
gctt

atttcttcattttccaggctgaataatattctgttgtacggacaccacat
ATTTCTTCgTTTTCCAGGCTGAATAATATTCcacTGTAtGGACACCACAT

ttcatatatccagtcctttgctgatggacactcgggttgcttctactttg
TTCgTgTATCCAGTCCTTTGtTGATGGACgCTtGaGTTGCTTCTACTTTG

ggctactgtgaataatgctgtaatgaacatgggtttgcaaagatctcttt
```

GGCTACTGTGAATAATGCTGTAATGAACATGGGTgTGCAAgGATCTCTTT

gagaccctaatttcaattcttttggttatatacccagaagtggaattact
aAGACCCTAATTTCAATTCTTTTGGTTATATACCCAGAAGTGGAATTgCT

gtatcatatggtaattctgtttttttattttatttttaattttaattttt
aTATCATATGGTAATTCTGTTTTTTATTTTATTTT--aTTTTA--TTTTT

atttttttttgagatggagtctcactctgtcacccagg
---------GAGATGGAGTCTCACTCTGTtgCCCAGG

chr4:161923972-161924184
Alignment
Human, chimp, rhesus, LTR10F consensus, donor

```
aaggcactaaagggaacccattaccacagctttgttgggctcagtccagg
AAGGCcCTAAAGGGAACCCATTACCACAGCTTTGTTGGGCTCAGTCCAGG
AAGGtcCTAAAGGGAACCaAcTcCCACAGCTTTGTTGGGCTCAGTCCAGG

tagcagcccccacatgttggagttgttataaataaaatttcggtgccaca
TAGCAGCCCCCACATGTTGGAGTTGTTATAAATAAAATTTtGGTGCCACA
TAGCAGCCCtCACATGTTGGAGTTGTTATAAATAAcAgTTtGGTGCtgCc
                       tgttatatataaagtttcggtgccgca

aaataaatagcacttgaatataaaattttctttttaattctcag-----caagtt
AAATAAATAGCACTcGAATATAAAATTTTCTTTTTAATTCTCAG-----CAAGTT
AAAgAAATAGCACTTGAATATAAAATTTTCTTTTTAATTCTCAt-----CAAGTT
aaagaaatagcactcgaatataaaattttctttttaattctcagcaaggcaaggt

acttctatagaagggtgcacccttacagatggagcaatggtaagtgcaca
ACTTCTATAGAAGGGTGCACCCTTACAGATGGAGCAATGGTAAGTGCACA
ACTTCTATAaAAaGGTGCgCCCTTACAGATGGAGCcATGGTgAGcaCACA
acttctatagaagggtgcaccctcacagatggagcaatggtgagcgcaca

cttggacaaggcaggggaaggggttcttatccctgacgcacgtggcccct
CTTGGACAAGGCAGGGGAAGGGGTTCTTATCCCTGACaCACGTGGCCCCT
CTTGGACAAGtgAGGGGAAGGGGTTCTTATCCCTGAtGCACGTGGCCCCT
cctggacaagggagggaaaggggttcttattcctgatgcacgtggtccct

gctgctgtgtctttcccctatgggctagggttagacctcacaggctaaac
GCTGCTGTGTCTTTCCCCTATcGGCTAGGGTTAGACCTCACAGGCTAAAC
GCTGCTGTGTCTTTCCCCTATtGGtTAGGGTTAGAtggCACAGGCTAAAC
actgctgtgtcattcccctattggctagggttagaccgcacaggctaaac

taattctgattggct-----aaagagagtgacagggtgaatagtttggccagaaa
TAATTCTGATTGcCT-----AAAGAGAGTGACAGGGTGAATAGTTTGGCCAGAAA
TAATTCTaATTGGCT-----AAAGAGAGcGAtgGGGTGAgTgGTTTGGtggcAAA
taattccgattgactaatttaaagagagtgacggggtgagtggtttggcgggaaa

aa----TTGTTACGACAGAGCATAACCATACCATAAATGGTATTTTATATTTTA
AA----TgGTTACGACAGAGCATAACCATACCATAAATGGTATTTTATATTTTA
AA----TgGTTAtGACAGAGCA--------------------------------
aaaaaatggttatg----------------------------------------
                                   TGGTATTTTGTATTTTA

CCAGGCCCAGTTAGGTTTATCCAAGCCAGAAGCTAGGCCTGTATCTATTT
CCAGGCCCAGTTAGGTTTATCCtAGCCAGAAGCTAGGCCTGTATCTATTT
--------------------------------------------------
--------------------------------------------------
CCAGGTCCAGTTAGGTTTATCCTAGCCAGAAGCTAGGTCTGTATCTATTT

GCTCTCAGCAGGTCAGTTACAATTTTGTATTCCTTTTTACATTATTCATG
GCTCTCAGCAGGTCAGTTACAATTTTGTATTCCTTTTTACATTATTCATG
--------------------------------------------------
--------------------------------------------------
GCTCTTAGCAGACCAGTTAAAATTTTGTATTCCTTTTTACATTATTCATG

ATCATCTTTATAATATCTCTTTTGCATGTAAATTTCACAATTTTTCTTTA
ATCATCTTTATAATATCTCTTTTGCATGTAAATTTCACAATTTTTCTTTA
--------------------------------------------------
--------------------------------------------------
ATCTTCTGTATAATATCTCTTTTGCATGTAAATTTCACAATTTTTCTTTA

CCATATGAA--------AATGAATcagggtggagcaggtaatctgaatgagtcagggt
CCATATGAA--------AATGAATCAGGGTGGAGCAGGTAATCTGAATGAGTCAGGGT
```

```
---------ggtaatcgaatgagtcagggtggagcaggtaATcAgAATGAgTCAGGGT
-------------------------------cagggtggagaatgaatgagtcagggt
CCATATGAA--------AAT

ggagcaggt-------aatctgaatgagtcagggtggagcaggtaatcagaacgagt
GGAaCAGGT-------AATCTGAATGAGTCAGGGTGGAGCAGGTAATCAGAAtGAGT
GGAGCAGGT-------AATCaGAATGAGTCAGGGTGGAGCAGGTAATCaGAATGAGT
ggagcaggtggtaggtaatcggaatgagtcagggtggagcaggtaatcggaatgagt

cagggt----------------------------gaagtaggtaatcataaaaggcttctttatgaggaagttaagtt
CAGGGT----------------------------GAAGTAGGTAATCATAAAAGGCTgCTTTATGAGGAAGTTAAGTT
CAGGGTGGAGCAGtTAATCAGcAtGAGTCAGGGTGgAGTAGGTAATCAaAAAAGGtTgCTTTATGAGGAAGTTAAGTT
cagggt----------------------------ggagcaggtaatcggaaaaggttgctttacgaggaagttaagtt

taaaagtagaaggcaaagaattgaacatactgacatattgattctttgaa
TAAAAGTAGAAGGCAAAGAATTGAACATACTGACATATTGATTCTTTGAA
TAAAAGTAGAAGGCAAAGAATTGAACAcACTGACATATTGATTCTTTGAA
taaaagtagaaggcaaagaattgaacatactgacatattaattctttgaa

aagaaaattagaactcatgtctaacaGAGTTTCATCTGTGTAGTTCTCCC
AAGAAAATTAGAACTCATGTCTAACAGAGTTTCATCTGTGTAGTTCTCCC
AAGAAAtTTAGAACTCATaTCTAACAGAGTcTCATCTGTGTAGTTaTCCt
gagaaatttagaattcatatttaaca

AGTTAGTGCTACAGTTCT
AGTTAGTGCTACAGTTCT
AGTTAGTGCTACAGTTCT
```

chr4:119252346-119252480

Alignment

Human, chimp, rhesus, AluY consensus, donor

```
ACACTTCAACTAATTTTTAATCATGAttttttttttttttttttt--gagat
ACACTTCAACTAATTTTTAATCATGATTTTTTTTTTTTTTTTTTTttGAGAT
ACACTTCAACTgcTTTTTAATCAaaaaaaaaATTTTTTTTTTTTcc-gagac
                                              tgagac

ggagtctcgctctgtcacccaggctggagtgtagcgacgccatcttagct
GGAGTCTCGCTCTGTCgCCCAGGCTGGAGTGTAGCGACGCCATCTTAGCT
aGAGTCTCGCTCTGTCgtCtAGGCTGGAGTGcAGtGgtGCCATaTcAGCT
ggagtctcgctctgtcgcccaggctggagtgcagtggcgcgatctcggct

cactgcaagctctgcctcccgggttaacgccattctcctgcctcagcctc
CACTGCAAGCTCTGCCTCCCGGGTTAACGCCATTCTCCTGCCTCAGCCTC
CACTGCAAGCTCcGCCTCCCaGGTTAAtGCCATTCTCCTGCCTCAGCCTC
cactgcaagctccgcctcccgggttcacgccattctcctgcctcagcctc

ctgagtagctgggactacaggtgcccgccaccatgcctggcaaaattttt
CTGAGTAGCTGGGACTACAGGTGCCCaCCACCAcGCCcGGCAAAATTTTT
CccAGTgGCTGGGACTACAGGTGCCCacccccacgactggctaattTTTT
ccgagtagctgggactacaggcgcccgccaccacgcccggctaatttttt

ttt-gtatttttagtagagatgggatttcaccatCCCATCTGAACTGAGAT
TTT-GTATTTTTAGTAGAGATGGGATTTCACCATCCCATCTGAACTGAGAT
TTTTGTATTTTTAGTAGAGATGGGATTTCACCATg----------------
    gtatttttagtagagacggggtttcaccgtg----------------
                                        TGAACTGAGAT

AAATATGTCAGAAGGCAGATATCATTTGTCTAACTCTTGCATGATTCAAA
AAATATGTCAGAAGGCAGATATCATTTGTCTAACTCTTGCATGATTCAAA
--------------------------------------------------
--------------------------------------------------
AAATATGTCAGAAGGCAGATATCATTTGTCTAACTCTTGCATGATTCAAA

TATATGAATATTTTAGTCACAGATTTCACTATGATATTCAGGAAAGGACA
TATATGAATATTTTAGTCACAGATTTCACcATGATATTCAGGAAAGGACA
--------------------------------------------------
--------------------------------------------------
TATATGAATATTTTAGTCACAGATTTCACCATGATATTCAGGAAAGGACA

ACAAATGCCTGAGCTATCTttttagccaggatggtcttgatctcctgacc
ACAAATGCCTGAGCTATCTTTTTAGCCAGGATGGTCTTGATCTCCTGACC
---------------------TTAGCCAGGATGGTCTcGATCTTaaGACC
---------------------ttagccaggatggtctcgatctcctgacc
ACAAATGCCTGAGCTATCTTTT

tcttgatctgcccgcctcagcctcccaaagtgctgggattacaggcatga
TCTTGATCTGCCCGCCTCAGCCTCCCAAAGTGCTGGGATTACAGGCATGA
TtgTGATCcaCCCGCCTCAGCCTCCCAAAGTGCTaGGATTACAGGCgTGA
tcgtgatccgcccgcctcggcctcccaaagtgctgggattacaggcgtga

gccaccatgcccggccCATAATTTTTAAAGTAATAAGCAACAGACATTTG
GCCACCATGCCCGGCCCATAATTTTTAAAGTAATAAGCAACAGACATTTG
GCCACCAaGCCCaGCCCATAATTaTgAAAGTAATAAGCAAgAGACATTTG
gccaccgcgcccggcc

TTTTCAATGTAATTCTAATGTAAAACATTCTCAAAGTAGGATTAAAAGAA
TTTTCAATGcAATTCTAATGTAAAACATTCTCAAAGTAGGATTAAAAGAA
TTTTCAATGTAATTCTAATGTAAAACATTCTCAAAGTAGGATTAAAAGAA
```

chr5:126417529-126417831
Alignment
Human, chimp, rhesus, L1PA6 consensus, donor

```
tctgttgagaatactatggatcgtcggcagatttatttattttATTTTA
TCTGTTGAGAATACTATGGAT-----------TTTATTTATTTTATTTTA
TCTGaTGAGAATACTATGGAT---CGGCAGATTTTATTT-TTT-------

TTTTTTTCCCACTTTCTCCtatattttgttattatactttttattatatat----
TTTTTTTCCCACTTTCTCCTATATTTTtTTATTATAC-----TTA-ATAT----
-------tCtACTTTCTCCTATATTTTTATTATACgTTTA--TTATATATaTTA

gttatatatatta-ta--CCtttttattatactttaagttctagggtacatgt
GTTATATATATTA-TA--CtTTTTTATTATACTTTAAGTTCTAGGGTACATGT
--TATATATgagacTATAttTTTTTATTATACTTTAAGTTCTAGGGcACATGT
                    tttttattatactttaagttctagggtacatgt

gcacaacgtgccgttttgttacacatgtatacatgtgccatgttggtttg
GCACAACGTGCCGTTTTGTTACAtATGTATACATGTGCCATGTTGGTTTG
GCcCAACGTGCaGTTTTGTTACAtATGTATACATGTGCCATGTTGGTTTG
gcacaacgtgcaggtttgttacataggtatacatgtgccatgttggtttg

ctgcacccatcaactagtcatttacattaggtatttctcctaatgctatc
CTGCACCCATCAACTAGTCATTTACATTAGGTATTTCTCCTAATGCTATC
CTGCACCCATtAACTtGTCATTTACATTAGGTATTTCTCCTAATGCTATC
ctgcacccatcaactcgtcatttacattaggtatttctcctaatgctatc

cctccctgctccccgcaccccacgacaggccccggtgtgtgatgttccac
CCTCCCTGCTCCCCGCACCCCACGACAGGCCCCGGTGTGTGATGTTCCAC
CCTCCCgcCTCCttcCACCCgACGACAGGCCCCaGTGTGTGATGTTCCcC
cctcccccagccccccaccccacgacaggccccggtgtgtgatgttcccc

accctgtgtccaagtgttctcattgttcaattcccacctatgagtgagaa
ACCCTGTGTCCAAGTGTTCTCATTGTTCAATTCCCACCTATGAGTGAGAA
ACCCTGTGTtCAAGTGTTCTCATgGTTCAATTCCCACCTATGAGTGAGAA
gccctgtgtccangtgttctcattgttcaattcccacctatgagtgagaa

cacgctgtgtttggttttctgtccatgcgacagtttgctccaaatgatgg
CAtGCTGTGTTTGGTTTTCTGTCCATGCGACAGTTTGCTCCAAATctTGG
CAtGtgGTGTTTGGTTTTCTGTCCATGtGAtAGTTTGCTCagAATGATGG
catgcggtgtttggttttctgtccttgtgatagtttgctgagaatgatgg

tttccagcttcatccatgtacctacaaaggacatgaactcatcctttttt
TTTCCAGCTTCATCCATGTctCTACAAAGGACATGAACTCATCCTTTTTT
TTTCCAGCTTCATCCAcGTcCCTACAAAGGACATGAACTCATCCTTTTT-
tttccagcttcatccatgtccctgcaaaggacatgaactcatcctttttt

atggctgcatagtattccatggtgtatatgtgccatattttcttaatcca
ATGGCTGCATAGTATTCCATaGTGTATATGTGCCATATTTTCTTAATCCA
ATGGCTGtATAGTATTCCATGGTGTATATaTGCCATATTTTCTTAATCCA
atggctgcatagtattccatggtgtatatgtgccacattttcttaatcca

gtctatcactgatggacattggggttggttccaagtctttgctattgtga
GTCTATCAtTGATGGACATTGGGGTTGGTTCCAAGTCTTTGCTATTGTGA
GTCTATCACTGATGGACATTtGGGTTGGTTCCAAGTCTTTGCTATTGTGA
gtctatcattgatggacatttgggttggttccaagtctttgctattgtga

acagtgtcgcaataaacatacctgtg--catgtgtctttagagtagcatgat
ACAGTGTCGCAATAAACATACCTGTG--CATGTGTCTTTAGAGTAGCATGAT
AtAGTGCcaCAATAAACATACaTGTGtgCATGTGTCTTTAGAGTAGCATGAT
atagtgccgcaataaacatacgtgtg--catgtgtctttatagcagcatgat

ttataatcctttgATTTATAATTGGCTTATCATGTTATTATTAGCATTAG
TTATAATCCTTTGATTTATAATTGGCTTATCATGTTATTATTAGCATTAG
```

```
TTATAATCCTTTG-------------------------------------
ttataatcctttg-------------------------------------
                   AATTGGCTTATCATGTTATTATTAGCATTAG

CAATACATAGATTATGGGTAGTTTGTAATGTTTTAAGATAATCATCCTAA
CAATACAcAGATTATGGGTAGTTTGTAATGTTTTAAGATAATCATCCTAA
--------------------------------------------------
--------------------------------------------------
CAATACATAGATTATGGATAGTTTGTAATGTTTTAAGATAAACATCCTAA

AATTTTCAGAGAAGGTTTCAAAAAGTCAACACAGGATCTTAAAAGCAAAA
AATTTTCAGAGAAGGTTTCAAAAAGTCAACACAGGATCTTAAAAGCAAAA
--------------------------------------------------
--------------------------------------------------
AATTTTCAGAGAAAGTTTCAAAAACTCAACACAGGATCTTAAAAGCAAAA

AAACCCAAGAACATGCTAAATTACAGAAGTACCAAATGTGCTAAAATTTT
AAACCCAAGAACATGCTAAATTACAGAAGTACCAAATGTGCTAAAATTTT
--------------------------------------------------
--------------------------------------------------
AAAACCAAGAGCATGCTAAATTACAGAAGTACCAATTGTGCTAAAATTTT

GCTTTTATATTAAGAATTTTAGAAGAATCTAGATTGGGTTTTTTGTTTTG
GCTTTTATATTAAGAATTTTAGAAGAATCTAGATTGGGTTTTTTGTTTTG
--------------------------------------------------
--------------------------------------------------
GCTTTTATATTAAGAACTTTAGAAGAATCTAGATTGGGTTTTCTGTTTTG

TTTT-----TTAGACCTGGGGTCCACAGAAGTCTATGCAAATTATACCTGAAAAT
TTTTgttttTTAGAtCTGGGGTCCACAGAAGTCTATGCAAATTATACCTGAAAAT
-------------------------------------------------------
-------------------------------------------------------
TTTTGTTTTTTAGACCTGGGGTCCACAGAAGTCTATGCAAATTATACCTGAAAAT

ATGTGTATATATTTTTA--tatatacacagtaataggatcactgggtcaaat
ATGTGTATATATTTTTA--TATATACACAGTAATAGGATCACTGGGTCAAAT
-----------------GGTATATACcCAGTAATAGGATtgCTGGGTCAAAT
-----------------ggtatatacccagtaat-gggatgctgggtcaaat
ATGTGTATATATTTTTA

ggtatttctagttctagatccttgaggaatcgccacactgttttccacaa
GGTATTTCTAGTTCTAGATCCTTGAGGAATCGCCACACTGTTTTCCACAA
GGTATTTCTAGTTCTAGATCCTTGAGGAATtGCCACACTcTcTTCCACAA
ggtatttctagttctagatccttgaggaatcgccacactgtcttccacaa

tggttgaactagtttgcagtcccaccaacagcataaaagcgttcctgttt
TGGTTGAACTAGTTTGCAGTCCCACCAACAGCATAAAAGCGTTCCTGTTT
TGGTTGAcCTAGTTTaCAcTCCCACCAACAGtgTAAAAGCaTTCCTaTTT
tggttgaactagtttacactcccaccaacagtgtaaaagcgttcctattt

ctccacatcctctccagcacctgttgtttcctgccttttaatgatcgcc
CTtCACATCCTCTCCAGCACCTGTTGTTTCCTGCCTTTTTAATGATCGCC
CTCCACATCCTCTCCAGCACCTGTTGTTTCCTGaCTTTTTAATGATtGCC
ctccacatcctctccagcatctgttgtttcctgacttttttaatgatcgcc

attctaactggtgtgagatggtatctcattgtggttttgatttgcatttc
ATTCTAACTGGTGTGAGATGGTATCTCATTGTGGTTTTGATTTGCATTTC
ATTCTAACTGGTGTGAGATGGTATCTCATTGTGGTTTTGATTTGCATTTC
attctaactggtgtgagatggtatctcattgtggttttgatttgcatttc

tctgatggccagtgatgatgagcatttttttcatgtgtctgttggctgcat
TCTGATGGCCAGTGATGATGAGCATTTTTTTCATGTGTCTGTTGGCTGCAT
TCTGATGaCCAGTGATGATGAGCATTTTTTTCATGTGTCTGTTGGCTGCAT
```

```
tctgatgaccagtgatgatgagcatttttttcatgtgtctgttggctgcat

aaatgtcttcttttgaaaagtgtctgtccatatcctttgcccactttttg
AAATGTCTT---TTGAAAAGTGTCTGTtCATATCCTTTGCCCACTTTTTG
AgATGTCTTCTTTTGAgAAGaGTaTGTtCATATCCTTTGCCCACTTTTTG
aaatgtcttcttttgagaagtgtctgttcatatcctttgcccactttttg

atgggattgtttgatttttttcttgtaaatttgtttaagttctttgtagat
ATGGGATTGTTTGATTTTTTCTTGTAAATTTGTTTAAGTTCTTTGTAGAT
ATGGagTTGTT--ATTTT--CTTGTAAATTTGTTTAAGTTaTTTGTAGAT
atggggttgtttgtttttttcttgtaaatttgtttaagttctttgtagat

tctggatattagccctttgtcagctgggtagattgcaaaaatttctcca
TCTGGATATTAGCCCTTTGTCAGaTGGGTAGATTGtAAAAATTTTCTCCA
TCTGGgTATTAGCCCTTTGTCAGaTGaGTAGATTGCAAAAAcTTTCTCCc
tctggatattag

attcagtaggttgtctgttcactctgatggtagtttcttttgctgtgcag
ATTCAGTAGGTTGTCTGTTCACTCTGATGGTAGTTTCTTTTGCTGTGCAG
ATTCAGTAGGTTGgCTGTTCACTCTGATGGTAGTTTCTTTTGCTGTGCAG

aagctctttagtttaattagatccagttagcctattttggcttttgttgc
AAGCTCTTTAGTTTAATTAGATCCAGTTtGCCTATTTTGGCTTTTGTTGC
AAcCTCTTTAGTTTAATTAGATCCcaTTtGtCTATTTTGGCTTTTGTTGC

cattgcttttggtgttttagtcatgaagtccttgcccatgcctatgtcct
CATTGCTTTTGGTGTTTTAGTCATGAAGTCCTTGCCCATGCCTATGTCCT
CATTGCTTTTGGTGTTTTAGTCATGAAGTCCTTGCCCATGCCTATGTCCT

gaatggtattgcttaggttttcttctagggtttttatggttttaagtcta
GAATGGTATTGCTTAGGTTTTCTTCTAGGGTTTTTATGGTTTcAAGTCTA
GAATGGTATTGCcTAGGTTTTCTTCTAGGGTTTTTATGGTTTTAgGTCTA

acatttaagtctttaatccatcttgaattaatttttgtataaggtgtaag
ACATTTAAGTCTTTAATgCATCTTGAATTAATTTTTGTATAAGGTGTAAG
ACATTTAAGTCTTTAATCCATCTTGAATTAATTTTTaTATAAGGTaTAAG

gaagggatccagtttcagctttctacatatggctagccagttttcccagc
GAAGGGATCCAGTTTCAGCTTTCTACATATGGCTAGCCAGTTTTCCCAGC
GAAGGGATCCAaTTTCAGCTTTCTACATATGGCTAGCCAGTTTTCCCAGC

accatttattaaataggaaatcctttccccattgcttgtttttgtcaggt
ACCATTTATTAAATAGGAAATCCTTTCCCCATTGCTTGTTTTTGTCAGGT
ACCATTTATTAAATAGGgAATCCTTTCCCCATTtCTTGTTTTTGTCAGGT

ttgtcaaagatcagatggttgtagatgtgtagtattatttctgagggctc
TTGTCAAAGATCAGATGGTTGTAGATGTGTAGTATTATTTCTGAGGGCTC
TTGTCAAAGATGG-----TTGTAGATGTGTgGTgTTATTTCTGAGGGCTC

tgtt------------------ctgttccatttgtctatatctctgttttggtaccagtaccatgctg
TGTT------------------CTGTTCCATTTGTCTATATCTCTGTTTTGGTACCAGTACCATGCTG
TGTTatttctgagggctctgttCTGTTtCATTgGTCTATATCTCTGTTTTGGTACCAGTACCATGCTG

ttttggttactgtaggcttgGaagaagaatcaatatcatgaaaatggcca
TTTTGGTTACTGTAGGCTTGGAgGAAGAATCAATATCATGAAAATGGCCA
TTTTGGTTACTGTAGcCTTGGAgGAAGAATCAATgTCgTGAAAATGGCCA

tactgcccaaggtaatttatagattcagtgccatccccatcaagctacca
TACTGCCCAAGGTAATTTATAGATTCAGTGCCATCCCCATCAAGCTACCA
TACTGCCCAAGGTAATTTATAGATTCAaTGCCATttCCATCAAGCTACCA

atgactttcttcacagaattggaaaaaaact
ATGACTTTCTTCACAGAATTGGAAAAAAACT
```

ATGACTTTCTTCACAGAATTGGAAAAAACT

chr7:19724202-19724323
Alignment
Human, chimp, rhesus, AluSc consensus, donor

```
TTTTTAAATGTGAAATCTCTTAAAAAATTTCT-ACTTTATTATGGGTTGTT
TTTTTAAATGTGAAATCTCTaAAAAAATTTCT-ACTTTATTATGGGTTGTT
TTTTTAAATGTGAAATATCTAAAACAATTTTACACTTGATTATGGGTTGTG

CTACCACATTTGCTAATTATTACAAGTTTTGCTCAGTTTATGGAtttttt
CTACCACATTTGCTAATTtTTACAAGTTTTGCTCAGTTTATGGATTTTTT
----CACATTTGCTAATTTTTACAAGTTGTGCTCAGTTTATGGAtttctt

tttttttttttttttt------gagaaagagacttgctctgtcaccaggctggag
TTTTTTTTTTTTTTT--------GAGAAAGAGACTcGCTCTGTCACCAGGCTGGAG
tcttttcttttttttttttttttaagacagaaactcactctgtcaccaggctggag
                      tgagacggagtctcgctctgtcgccaggctggag

tgcagtggtgctgtcttggctcactgcaacctccacttcccaggttcaag
TGCAGTGGTGCTGTCTTGGCTCACTGCAACCTCCACTTCCCAGGTTCAAG
tgcagtggtgcgatcttggctcactgcaacctccgcctcccgggttcaag
tgcagtggcgcgatctcggctcactgcaacctccgcctcccgggttcaag

tgattctcctgcctcagcctcccgagtagggggactacaggcgcatgcc
TGATTCTCCTGCCTCAGCCTCCCaAGTAGGGGGACTACAGatGCATGCC
tgattctcctgcctcagcctcccaagtaggggggctgcaggcgcgtgcc
cgattctcctgcctcagcctcccgagtagctgggactacaggcgcgcgcc

accacgcccagctaattttgtatttttagtagagatggggtttcaccat
ACCACGCCCAGCTAATTTTTGTATTTTTAGTAGAGATGGGGTTTCACCAT
accgtgcccagctaattttgtatttttagtagagacggggtttcaccat
accacgcccagctaattttgtatttttagtagagacggggtttcaccat
                                                 T

TCTTTTTTACTCCCTTTGCCAGGAAAACAATTTATTGAATACAAGAGTCC
TCTTTTTTACTCCCTTTGCCAGGAAAACAATTTATTGAATACAAGAGTCC
--------------------------------------------------
--------------------------------------------------
TCTTTTTTACTCCCTTTGCCAGGAAAACAATTTATTGAATACAAGAGTCC

TTTATTAGGAATGCAAATGCATTTCACAAGTAATATATTGCTTAAAAACC
TTTAcTAGGAATGCAAcTGCATTTCACAAGTAATATATTGtTTAAAAACC
--------------------------------------------------
--------------------------------------------------
TTTACTAGGAACATAAATGCATTTCACAAGTAATATATTGCTTAAAAACC

AAGCTATAGCTTTAATAAAATTAtttcaccatgttggccaggatggtctc
AAGCTATAGCTTTAATAAAATTATTTCACCATGTTGGCCAGGATGGTCTC
--------------------------------gttggccaggatggtctc
--------------------------------gttggccaggatggtctc
AAGCTATAGCTTTAATAAAATTATTTCA

tatttcttgaccttgtgattcactcacctcagcctcccaaagtgctgaga
TATTTCTTGACCTTGTGATTCACTCACCTCAGCCTCCCAAAGTaCTGAGA
aatttcttgacctcgtgatccgctcgcctcggcctcccaaagtgctgaga
gatctcttgacctcgtgatccgcccgcctcggcctcccaaagtgctggga

ttacaggcgtaagctaccacgccctgccCAGTTCATGGATTTATAATTGG
TTAtAGGCGTAAGCTACtACGCCCTGCCCAGTTCATGGATTTATAATTGG
ttacaggcgtgaatcaccgcacccggccCAATTCATGGATTTATAATTGG
ttacaggcgtgagccaccgcgcccggcc

TGCATCACCTTTCAAATTCCATGGAGTATTTAC
TGCATCACCTTTCAAATTCCATGGAGTATTTAC
TGCATCACCTTTCAAATTCCATGGAGTATTTAC
```

```
chr11:94292975-94293144
Alignment
Human, chimp, rhesus, MER20 consensus, donor
ttagttattagtacccactgtggcccaagtacttggctaggcattgggaa
TTAGTTATTAGTACCCACTGTGGCCCAAGTACTTGGCTAGGCATTGGGAA
TTAGTTATTAGTACCCtCTaTGGCCCAAGTACTTtGCTAGGCATTGGGAA

taccaaagtaaatgaggtagtcatagctctgttgcagagAGgtggttctc
TACCAAAGTAAATGAGGTAGTCATAGCTCTGTTGCAGAGAGGTGGTTCTC
TACCAAAGTAAcTGAGGTAGTCATAGCTCTGTTGCAGAGAGGTGGTTCTC
                                         gtggttctc

aacatggagcggttttggctcccaagggccatttgccaatgtctggatac
AACATGGAGCaGTTTTGGCTCCCAAGGGCCATTTGCCAATGTCTGGATAC
AACATGGAGCaGTTTgGGCTCCCAAGGGCCATTTGgCAATGT-TGGAgAC
aaccgggggtgattttgccccccaggggacatttggcaatgtctggagac

atttttgatggtcttgacttgggatgagg-ttgcta----------------------cagggatgttgctga
ATTTTTGATGGTCTTGACTTGGGATGAGG-TTGCTA----------------------CAGGGATGTTGCTGA
ATTTTTGATtGTCaTGACTTGGGATGAGG-TTGCTA----------------------aAGGGATGTTGCTGA
atttttggttgtcacaactggggggggggratgctactggcatctagtgggtagaggccagggatgctgctaa

acatcctataacacatagaacagcccccacaataaagaactaCACAACAA
ACATCCTATAACACAcAGAACAGCCCCCACAATAAAGAACTACACAACAA
ACATCCTATAAtgCATAGgAgAGCCCCCAtgATAAAGGACTa--------
acatcctacaatgcacaggacagcccccacaacaaagaatta--------

AGAAGCCACAGAATCCGCTTGTCAGACTGTCTCTTCTGCACTCCCCCTTT
AGAAGCCACAGAATCCaCTTGTCAGACTGTCTCTTCTGCACTCCtCCTTT
--------------------------------------------------
--------------------------------------------------
AGGAACCACCGAATCCGCTTGTCAGACTGTCTCTTCTGCACTTCTCCTTT

CAAGAAGACACACCAAGTTCCCATGATCACATACTCCCTTCCCCCGACCA
CAAGAAGACACAtCAAGTTCCCATGATCACATACTCCCTTCCCCtGACCA
--------------------------------------------------
--------------------------------------------------
CAAGAAGACACACCAAGTTCCCATGATCACATACTCCCTTCCCCTGACCA

CACATGGCCTGGGCCCACTGAAGGCCCAGGAGGCTGGACATGAGGCAGTG
CACATGGCCTGGGCCCACTGAAGGCCCAGGAGGCTGGACATGAGGCAGTG
--------------------------------------------------
--------------------------------------------------
CACATGGCCCAGGCCCACTGAAGGCCCAGGAGGCTGGACGCAAGGCAGTG

AGGTGGAATACAA---ggccccaaatgtcaacagtgttgaggttgaga--TC---TTA
AGGTGGAATACAA---GGCCCCAAATGTCAACAGTGTTGAGGTTGAGaaAcC---TTA
-------------TCTGGCCCCAAATGTCAACAGTGTTGAGGTTGAGaaAcCcTATTA
-------------tccggcccaaaatgtcgatagtgccaaggttgagaaaccctg
AGGTGGAATACAa---g

TTATTGAGCATAAGGTCTATGACAATTAAACACATAGTTAAAAT
TTATTGAGCATAAGGTCTATGACAATTAAACACATAGTTAAAAT
---TTGAGCATAAGGTCTATGACAATTAAACACATAGTTAAAAT
```

```
chr14:35205139-35205342
Alignment
Human, chimp, rhesus, AluSx consensus, donor
ATGATATGAATGAAATCTAAAATAAGAATACAACGAGCAAGAATTGGACT
ATGATATGAATGAAATCTAAAATAAGAATACAAaGAGCAAGAATTGGACT
ATGATATGAATGAAATCTAAAATAAGAATACAAaGAGCAAGAATTGGACT

AGGTGTTTCCTCCtttttttttttttctt--gagaaggagtttcactctgtc
AGGTGTTTCCTCCTTTTTTTTTTTTTCTT--GAGAAGGAGTTTCACTCTGTC
AGGTGTTTCCTCCttTTTTTTTTTTTTTtTTaAGAAGGAGTTTCACTCTGTC
                              tgagacggagtctcgctctgtc

gtgcaggctggagtacagtggcacaatctcagctcactgcaacctccacc
GTGCAGGCTGGAGTACAGTGGCACgATCTCAGCTCACTGCAACCTCCACC
aTGCAGGCTGGAGTAtAGTGGCACgATCTCgGCTCACTGCAACtTCCgCC
gcccaggctggagtgcagtggcgcgatctcggctcactgcaacctccgcc

tcccagggtcaaatgatcctcccatctctcccatctcagactcctgagta
TCCtAGGtTCAAAcGATCCTCCCATCTCTCCCATCTCAGACTCCTGAGTA
TCCCAGGtTCAAAcGATCCTCCCATCTCTCCCATCTCAGACTCCTGAGTA
tcccgggttcaagcgattctcctgc---------ctcagcctcccgagta

gctgggattacaggtgcgtgccaccacTACCAGAAAAAATTTTAAAGACT
GCTGGGATTACAGGTGCGTGCCACCACTACCAGAAAAAATTTTAAAGACT
GCTGGGATTACAGGcGtGcaCCACCAC-----------------------
gctgggattacaggcgcgcgccaccac-----------------------
                          CTACCAGAAAACATTTTGAAGACT

TATAAACAACAAGGGTCATCTAGCATGTCTGCTCCTAACTGGACACAATT
TATAAACAACAAGGGTCATCTgGCATGTCTGCTCCTAACTGGACACAATT
--------------------------------------------------
--------------------------------------------------
TATAAACAACAAGGGTGATCTGGTATGTCTTCTCCTAACTGGACACAATT

ATCTTTTTTTTTTAAGGATTAATATCTGTGGTAGGTTCCACTGGCAACTG
ATCcTTTTTTTTTAAGGATTAATATCTGTGGTAGGTTCCACTGGCAACTG
--------------------------------------------------
--------------------------------------------------
GTCTTTTTTTT--AAGGATTAATATCTGTGGTAAGTTCCACTGGCAACTG

TATAGTCACATTTATTCTGCACCATCTACTGTTCAAGCCCCTTAATGTCT
TATAGTCACATTTATTCTGCACCATCTACTGTTCAAGCCCCTTAATGcCT
--------------------------------------------------
--------------------------------------------------
TATAGTCACATTTATTTTGCACCATCTACTGTTCAAGCCCCTAAATGCCT

CAGGTACATGGGATCTCTATCCCAACTTCATA-----gttaatttttgtatttgt
CAGGTACATGGGATCTCTATCCCAACTTCATA-----GTTAATTTTTGTATTcGT
--------------------------------GCCCGGcTAATTTTTcTATTTGT
--------------------------------gcccggctaatttttgtattttt
CTGGTACATGGGATCTCCATCCCAACTCCATA-----GTT

agtagagatggagtttcaccatgttggccaggctggtctcaaactcctga
AGTAGAGATGGAGTTTCACCATGTTGGCCAGGCTGGTCTCAAACTCCTGA
AGTAGAtATGGAGTTTCACCAcGTTGGCCAGGCTaGTCTCAAACTCCTGA
agtagagacggggtttcaccatgttggccaggctggtctcgaactcctga

cctcaagtgatctgcctgtgtcagtctc-ccaaagtgttgggattacaggt
CCTCAAGTGATCTGCCTGTGTCAGTCTC-CCAAAGTGTTGGGATTACAGGT
CCTCAAGTGATCcGCCTGTGTCAGcCTCtCCAAAGTGcTGGGATTACAGGT
cctcaggtgatccgcccgcctcggcctccc-aaagtgctgggattacaggc

gtgagccaccatgcccagccTGGACTGGGTATTTCCAACACCATCAGAGG
```

GTGAGCCACCAcGCCCAGCCTGGACTGGGTATTTCCAACACCATCgGAGG
GTaAGCCACCAcGCCCAGCCTGGACTGGGTATTTCCAACACCAcCctAGG
gtgagccaccgcgcccggcc

AAATGACTTGTTTTAACCTTATTGTGGAGATATGTTAGGCTTGATTCTTG
AAATGACTTGTTTTAACCTTATTGTGGAGATATGTTAGGCTTGATTCTTG
AAATGACTTGTTTTAACCTTATTaTGGAGATATGTTAtGCTTGATTCTTG

chr2:82389199-82389351
Alignment
Human, chimp, rhesus, L1MA9 consensus, donor

```
TTAAGGACATAAAGTGTTATTGAACATGTCCTTAGAGGCTAAAGTAACTC
TTAAGGACATAAAGTGTTATTGAACATGTCCTTAGAaGCTAAAGTAACTC
          TAAAGTaTTATTGAAtATaTCCTTAGAGGCTAAAGTAACTC

TAGGGGCATTGGACTTCCAGCAAATTAAGGAAGGTTGACACATACTGCAT
TAGGGGCATTGGACTTCCAGCAAATTAAGGAAGGTTGACACATACTGCAT
TAGGGa-ATTGGACTTCCAGCAAATTAAGGAgGGTTGACgCATAtTGCAT

GTCGAGAAACTACAAGGTGATTCTGAGTCACATCTGACAGAACAAATAAT
GTCGAGAAACTACAAGGTGATTCTGAGTCACATCTGACAGAAgAAATgAT
GTCGAGAAACTACtAGGTGcTTCTGAGTCACATCTGACAGAACAAtTAAT

GACAGCCCTCTtacagaccaatggaacaaaacagaaagtctagaaataaa
GACAGCCCTCTTACAGACCAATGGAACAAAACAGAAAGcCTAGAAATAAA
GACAGCCCTCTTACAGACCAATGGAACAAAACAGAAAGcCTAGAAATAgA

gtcatgcatatgtggtcaactgattaca--------ca-tgtgaggaaatatttttcaa
GTCgTGCATATGTGGTCAACTGATTACA--------CA-TGTGAGGAAATATTTTTCAA
GctATGCATATGTGGTaAACgGATTACAgTTTTTGACACAGTGgGGAAAaAgTTTTCAA

tattttatttttgacacagtgaggaaatatttttcaataaatggtgttgg
cATTTTATTTTTGACACAGTGAGGAAATATTTTTCAATAAATGGTGTTGG
-----------------------------------------TAAAcGGTGTTGG

aaaaactgtgtatccacatgcaaagaaatgaaataagactcatctcacac
AAAAACTGTGTATCCACATGCAAAGAAATGAAATAAGACTCATCTCACAC
AAAAACTGTaTATCCACATGCAAAGgAATGAAATAAGACTCATCTCACAC

catttataaaagtcaactcaaaataaattaaaggcttaaatgtaagacct
CATTTATAAAAGTCAACTCAAAATAAATTAAAGGCTTAAATGTAAGACCT
CATTTATAAAAaTCAACTCAAAATAAATTAAAGGCTTAAATGTAAGAtCT

gaaatcgtaaagctactagaagaaatacagagaaaaaacttcttgatatt
GAAATtGTAAAGCTACTAGAAGAAATACAGAGAAAAAACTTCTTGATATT
GAAATtGTAAAGCTACTAGAAGAAACcGAGA---AAAACTTCTTGATATT

gatctggaaaatcgttttctggatataaccccaaaatcacaggtaacaga
GATCTGGAAAATCGTTTTCTGGATATAACCCCAAAATCACAGGTAACAGA
GATCTGGAcAATtGTTTTCTaGATATAACCCCAgAATCAtAGGTAACAGA

agcaaaaatagaaaacgtgatgatattaagctaaaaatggttttgcacag
AGCAAAAATAGAAAAtGTGATGATATTAAGCTAAAAATcGTTcTGCACAG
AGCAAAAATAGAAAAtGTGATGATATTAAGCTAAAAgTGGTTcTGtACAG

caaagaaaacacttgaaagagtgaagagacaacctatagaatATGTACCG
CAAA-----CACTTGAAAGAGTGAAGAGACAACCTATAGAATATGTACCa
CAAtGAAAgCACTTGAAAGAGTGAAGAGACAACCTATAGAAT--------
                                    agaaTATGTACCA

TATTACCATAGATACAGGGTGTGTAAAGCCTTTCTGCATAGAAATCAGCA
TATTACCATAGATACAGGGTGTGTAAAGCCTTTCTGCATAGAAATCAGCA
--------------------------------------------------
TATTACCATAGATACAGGGTGTGTAAAGCCTTTCTACATAGAAATCAGCA

AAGTTACAGGGCCACCAATTTTTGTGTGTGTCAATTTAAGGAGAAAAATA
AAGTTACAGGGCCACCAATTTTTGTGTGTGTCAATTTAAGGAGAAAAATA
--------------------------------------------------
AAGTTACAGAGCCACCAATTTTTGTGTGTGTCAATTTAAGGAGAAAAATA

AAGTTTCAATAGATAACATGAGCATTTTTCATAGAATGGGAGAAAC--gaga
```

```
AAGTTTCAATAGATAACATGAGCATTgTTCATAGAATGGGAGAAAC------
----------------------------------------------GggAGA
AAGTTTCAATAGATAAC--GAGCATTGTTCA

aactattttcaacccatacatctgagaaggggttaatatccaaaatatat
---TATTTTCAACCCATACATCTGAGAAGGGGTTAATATCCAAAATATAT
AACTgTTTTCAgCCCATACATCTcAGAAGGaGTTAATATCCAAAATATAT

aaataactcaaacaattcagtagcaagaaaacagagaatcaaattaaaaa
AAATAACTCAAACAATTCAGTAGCAAGAAAACAGAGAATCAAATTAAAAA
AAATAAtTCAAACAATTCAGTAGCAAtAAAACAaAtAATCAAATTAAAAA

tgggcaaaggacctcagtagatattgatccaaaaagatgtacaaatgtcc
TGGGCAAAGGACCTCAGTAGATAcTGATCCAAAAAGATGTACAAATGTCC
TGGGCAAAGGAttTCAGTAGAaATTGATCCAAAAAGATGTAtAAATGgCC

aagaagaacagaaaaataggttcaacatcactgatcatcacgaaaatgca
AAGAAGtAtAGAAAAATAGGTTtAACATCACTGATCATCACGAAAATGCA
AAGAAGtAtAGAAAAATAtGTTCAAtATCACTGATCATCAgGAAAATGCA

aatacaaaccacaatgatgtatcacttcacacctgttaggatggctatca
AATcCAAACCACAATGATGTATCACTTCACACCTGTTAGGATGGCTATCA
AATcCAAACCACAATGATaTATCACTTCACACtTGTTAGGATGGCTATCg

tcaagaaaacaaaaggtaagtgttgggagaggatatggtgaaaaggaact
TCAAGAAAACAAAAGGTAAGTGTTGGGAGAGGATATGGTGAAAAGGAACT
TtAAGAAAAtgAAAGaTAAGTGaTGGGAGAGGATATGGTGAAAAGGAACT

ccttgttcactatgggagggaatgtaaattggtgcagccattatggaaaa
CCTTGTTCACTgTGGGAGGGAATGTAAATTGGTGCAGCCATTATaGAAAA
CCTTGTTCACcgTGGGAGGGAATGTAAATTGcTGCAGCCATTgTGGAAAA

gaatatggaggttccttaaaaaattaaaaagtagaactcccatatgatac
GAATATGGAGGTTCCTTAAAAAATTAAAAAGTAGAACTtCCATATGATAC
GAATATGGAGaTTCCTTAAAAcATTAAAAAGTAGAACTtCCATATGATAt

agcaatttgactaatgagtatatatcaaaagaaaatgaaatcaactcctc
AGCAATTTGACT---GAGTATATATCAAAAGAAAATGAAATCAACTCCTC
AGCAATTTGACTAATGAGTATATATCcAAA----------TCAAgTCCTC

agagagatatcttcactcacatgttaattccagcattattcacaaaagcc
AGAGAGATATCTTCACTCACATGTTAATTtCAGCATTATTCACAAAAGCC
AGAGAGATAgCTTCACTCcCATGTTAATTgCAGCATTATTCACAAAAGCC

aagacacagactcaacctgagtattcattgatggaagcatggataaagaa
AAGACACAGACTCAACCTGAGTATTCAcTGATGGAAGCATGGATAAAGAA
AAGACACAGAaTCAACCTGAaTATTCAcTGATGGAAGgATGGATAAAGAA

aatgtagtgtgtgtgtgtttgtgtgtgtgtgtgtgtg-----------------aatattattcacc
AATGTAGTGTGTGTGTGTTtgtgtgtttgTGTGTGTGTGTGTGTG-------AATATTATTCACC
AATGTAGTGTGTGTGTGTgTGTGTGTGTGTGTGTGTGtgtgtgtgtgtgtgtttgaaTATTCACC

ctgaaaaaaaa-tcctgtcatttt-caacaacattgataagcctggaggaca
CTGAAAAAAAA-TCCTGTCATTTT-CAAtAACATgGATAAGCCTGGAGGACA
CTGAAAcAAAAATCCTGTCATTTTtCAACAACATgGATAAGCtTGGAGGACA

ttatgctaagtgaaataaactaggcacaggaagacaaataaggtatgatc
TTATGCTAAGTGAAATAAACTAGGCACAGGAAGACAAATActGTATGATC
TTATGCTAAGTGAAATAAACcAGGCACAGGAAGACAAATActGTgTcATC

tcacttatatcttcaatctgaaagaatcagacttatagaagtagaaagta
TCACTTATATCTTCAATCTGAAAGAATCAGACTTATAGAAGTAGAAAGTA
TCACTTATAaCTTCAATCTaAAAaAATCAGACTTATAGAAGTAGAAAGTA
```

```
gaatggtggttgtcatgggccgaggg---------gagcaagaaatggggaaatattgc
GAATGGTGGTTGTCATGGGCCGATtctcggcccAtGAGCAAGAAATGGGGAAATATTGC
GAATGGTGGTTGTCATGGGCtGAGGG---------GAaCAAGAAATGGGGAAgTgTTGC

tcagagaatacaaggtttcctttatgcaggatgaatatgttctagatacc
TCAGAGAATACAAGGTTTCCTTTATGCAGGATGAATATGTTCTAGATACC
TCAGAGAATACAAGGTTTCCTTTATGCAGGATGAATATaTTCTAGATACC

taacgtacagcctggtgacaatcattaacaatactgtatttaacgcttga
TAAtGTACAGgCTGGTGACAATCATTAAtAATACTGTATTTAACGCTTGA
TAAtGcACAGaCTGGTGACAATtgTTAAtAATACTGTATTTAACaCTTGA

aatttgctaggagagtaggttaaatgctctccccacccacaccaacacac
AATTTGCTAGGAGAGTAGGTTAAATGCTCTCCCCACCCACACgAACACAC
AATTTGCTAaGAGAGTAGGTTAAATGtTCTCaCCACctacacCCACACAC

aaacacacacacagtggtaactatgtgagctgatggatatagtaattggc
AAACACACACACAaTGGTAACTATGTGAGCTGATGGATATAGTAATTGGC
AAACACAaaa----TGGTAACTAgGTGAGCTGATGGATATAGTAATTGGC

ttgattgtaaatatatatgtacataaaa--tcatgttttataccttaaatat
TTGATTGTAA-TATATATGTAtATAAAA--TCATGTTTTATACCTTAAATAT
TTGATTGTAA-TATAcATGTAtacaaatAATCATGTTTTATACCTTAAATAT

atat---ttttatttgtcaattatacctcaataaaactggagaaaATAAAGTG
ATAT---TTTTATTTGTCAATTATACCTCAATAAAACTGGAGAAAATAAAGTG
ATATaatTTTTATTTGTCAATTATACtTCAATAAAACTGGAaAAAATAAAGTG

GAAAAAATGAACTGGA-TAGCTTGCATAGAATTTGAGAATTTGGATGATTT
GAAgAAATaAACTGGA-TAGCTTGCATAGAATTTGAGAATTTGGATGATTT
GAtagtttgttctggattagcttagacAGAATTTGAGAATTTGGATATTTT

TTTTTACATATTCTTGACTTAATATAAGGTTTCTTATATTATGTACTATC
TTTTTACtTATTCTTGACTTAATATAAGGTTTCTTATATTATGTACTATC
Tggg----TATTCTTGACT-AATATAAGGTTTtTcAcATTATGTACTATC

TATTACATTATGCTATTTCCTTTCCAAATTCTTGGGCAGTCAACACCACT
TATTACATTATGCTcTTTCCTTTCCAAATTCTTGGGCAaTCAACACCACT
TATTACATTATGCTATTTCCTTTCatac-TCTTGGGCAaTCAACACCACT

GTATTTTTACCATGTCACCTATCAGGGTCAATTCTCAGA
GTATTTTTACCATGTCACCTATCAGaGTCAATTCTCAGA
GTATTTTTACCATGTCcCCTATCAGaGgCAATTCTCAGg
```

chr5:27200130-27200356
Alignment
Human, chimp, rhesus, Tigger3b consensus, donor

```
ACACTGTGCTCACAAGCTCCTTTTTATAATCCATTTATTGATAGGCTAAT
ACACTGTGCTCACAAGCTCCTTTTTATAATCCATTTATTGATAGGCTAAT
GCACTGTGCTCACAAGCTtCTTTTTATAATCtATgTATTGATAGGCTAAT

TAACACTATTTACAAGGCAATTTCTTTAAGTAAATtcatgcactgcataa
TAACACTATTTACAAGGCAATTTCTTTAAGTAAATtcatgcactgcataa
TAACAaTATTTACAAGGCAATTTCTgTAAGTAAATTCATGCACTGaATAA

caatgtttcagtcaacaaccttggtcccataagattataatggagctaaa
caatgtttcagtcaacaaccttggtcccataagattataatggagctaaa
CAATGTTTCAGTCAACAACCTTGGTCCCATAAGATTAaAATGGAGCTAAA
              acgacggtggtcccataagattataatggagctgaa

gaattcctgttacctagtgatcctatctgtagcagtggcaatgt---agcaca
aaattcctgttacctagtcatcctatctgtagcagtggcaatgt---agcaca
aAATTCCTGTTACCTAaTGATgCTATCTGTAGCcaTGGtAATGT---AaCACA
aaattcctatcgccta----gtgacgtcgtagccatcgtaacgtcgtagcgca

aggcattaatcatgtgtttgcaatgatga---tataaacaaacctactgtgct
aggcattaatcatgtgtttgcaatgatga---tataaacaaacctactgtgct
AGGCATTAATCgTGTGTTTGCAAcGAcgaTGATATAAACAAACCTACTGTGCT
acgcattactcacgtgtttgtggtgatgctggtgtaaacaaacctactgcgct

atcagttgtataaaagtataagaaatacaattatgtacagtacaagatag
atcagttgtataaaagtataagcaatacaattatatacagtacatgatag
ATCAGcTGTATAAAAGTATAAGtAATACAATTATGTACAaTACAtGATAG
gccagtcgtataaaagtatagcacatacaattatgtacagtacataatac

ttgataatgataataaatgactatattactggtttatttatttactatac
ttgataatgataataaatgactatattactggtttatttatttactatac
TTGATAAgGATAATAAATGACTgTATTACTGcTTTATTTATTTACT-TAC
ttgataatgataataaacgactatgttactggtttatgtatttactatac

tatcctttctgtcattattttagagtgtactcctagcaataaagaaaa------gc
tatcctttctgtcattattttagagtgtactcctagcaataaagaaaa------gc
TATgCTTTCTaTCATTATTTTAGAGTGTACTCCTAcCAATAAAGAAAA------GC
tatacttttatcgttattttagagtgtactccttctacttattaaaaaaaaaagt

tgactgt----------caggcaggtccctcaggaagtattccagaagaaggtgttgtta
tgactgt----------caggcaggtccctcaggaagtattccagaagaaggtgttgtta
TGACTGT----------CAGGCAGGTCCCTCAGGAgGTATTCCAGAAGAAGGcaTTGTTA
taactgtaaaacagcctcaggcaggtccttcaggaggtattccagaagaaggcattgtta

tcactggagatgacagctccatgcatgttactgtccctgaagaccttcct
tcactggagatgacagctccatgcatgttactgtccctgaagaccttccc
TCACTGGAGATGACAGCTCCATGCATGcTAtTGcCCCTGAAGACCTTCtT
tcataggagatgacagctccatgcgtgttattgcccctgaagaccttcca

gtgggccaaaaatgtggaagttgaagacagtgatattgatgattcaaacc
gtgggccaaaaatgtggaagttgaagacagtgatattgatgattcaaacc
GTGGGCCAAAAATGTGGAAGTTGAAGACAGTGATATTGATGATTtAAACC
gtgggacaaga-tgtggaggtggaagacagtgatattgatgatcctgacc

ctgtgaaggcttacggtaatgtgtgtgcatgtctgtgttttaacttttca
gtgtgaaggcttacggtaatgtgtgtgcatgtctgtgttttaacttttca
-TGTGAAGGCTTAgGGTAATGTGTG--TGTGTCTGTGTTTTAACTTTTCA
ctgtgtaggcctaggctaatgtgtgtgtttgtgtc----ttagtttttaa

caaaaaagtttaaatgtccaaaatt-taaacatagaaaaaagtttaaagag--------
caaaaaagtttaaatgttcaaaatt-taaacatagaaaaaagtttaaagac--------
```

```
CAAAAAcGTTTAAATGTtCAAAAaaGcTAAACATAGAAAAAGTTTAAAGAa--------
caaaaaagtttaaaaagtaaaaaaaaaataawtttaaaaatagaaaaaagcttatagaa

taaggatactaaaac-g----tatatttgtacagatgtacagtgtg-ttgtatttt
taaggatactaaaaa-g----tatatttgtacagatgtacagtgtgtttgtatttt
TAAGGATACTAAAAaTa----TATATTTGTACAGATGTACAGTGcGTTtGTgTaTT
taaggatataaagaaagaaaatattttgtacagctgtacaatgtgtttgtgtttt

aagctatgtTTCTTGAACAGCTTGACTTCATTTAAGAGTGAAGCCATATT
aagctatgtTTCTTGAACAGCTTGACTTCATTTAAGAGTGAAGCCATATT
AAGCTATGCT----------------------------------------
aagctaagtg----------------------------------------
      TGTTTCTTGAACAGCTTGACTTCATTTAAGAGTGAAGCCATATC

TCTCTTTTGCTGTTTCCCACTTAAAACTTTTTAAGAACTGCCTAGTTATA
TCTCCTTTGCTGTTTCCCACTTAAAATTTTTTAAAAACTGCCTAGTTATA
--------------------------------------------------
--------------------------------------------------
TCTCCTTTGCTGTTTCCCACTTAAAATTTTTAAAAAACTGCCTAGTTATA

CATTAGTTTATTTTCTATTACACACTTTCTTTTCTAACTCTTACTGCATT
CATTAGTTTATTTTCTATTACACACTTTCTTTTCTAACTCTTACTGCGTT
--------------------------------------------------
--------------------------------------------------
CATTAGTTTATTTTCTATTACACACTTTCTTTTGCAACTCTTACTGCATT

CTAATGCTGTTTCTGACTTATAAAAATGAGATACATCTCAGGGCAAGTTT
CTAATGCTGTTTCTGACTTATAAAAATGAGATACATCTCAGGGCAAGTTT
--------------------------------------------------
--------------------------------------------------
TTAATGCTCTTTCTGACTTATAAGAATGAGATACATCTCAGGGCAAGTTT

GGGTTGGAGGTGTGGGGTGTCAGTTCACAAAAGTTAT---------aagagtcaaaatg
GGGTAGGAGGTGTGGGGTGTCAGTTCACAAAAGTTAT---------aagagtcaaaatg
---------------------------------------ATTACA--CGAGTCAAAATG
-------------------------------------ttattacaaaagagtcaaaaag
GGGTAGGAGGTGTGGGGTGTCAGTTCACAAAAG

ttaaaaaaaatgaaaaagtttatacagtaaaaaagttacagtaagctacg
ttaaaaaaaatgaaaaagtttatacagtaaaaaagttacagtaagctacg
TTAAAAAAA-TtAggAAGTTTATAaAGTAAAAAAGTTACAGTAAGCTACa
ttwaaaaaattwaaaa-gtttataaagtaaaaaagttacagtaagctaag

gataatgtatttttt-ggggaaagaaaaaaattaaaataaatttaatatag
gataatgtattttttttggggaaagaaaaaaattaaaataaatataatatag
GATAATGcATTTTTGaGGAAgGAAAAAAA--TTAAAATAAATTTAgTATAG
gttaatttattattgaagaaagaaaaatattttwaa-taaatttagtgtag

ccta-gaatacagtgtttacaaagtctacagtaaagtacagtattgtctta
ccta-gaatacagtgtttacaaagtctacagtaaagtacagtattgtctta
CCTA-GAtTACAGTGTTTACAAAGTCTAtAGTAAAGTtCAGTgTTGTCTTA
cctaagtgtacagtgtttataaagtctacagtagtgtacagtaatgtccta

ggccttcacattcattcaccactcactcactgacttatccagatcaactt
ggccttcacattcattcaccactcactcactgacttatccagatcaactt
GGCCTTCACATTCATTCACCACTCACTCACTGACTTAcCCAGATCAACTT
ggccttcacattcactcaccactcactcactgactcacccagagcaactt

ccagtcctgcaagctggaagctccatttatagtcaatgtccaatacaggt
ccagtcctgcaagctggaagctccatttatagtcaatgtccaatacaggt
CCAGTCCTGCAAGCTGGAAcCTCCATTTATAGTaAATGTCCAATACAGGT
ccagtcctgcaagct
```

```
atatcattcttACGTTTTTCTTTCATGAAGTAAAGAGCTAAGCCAGGCAT
atatcattcttACGTTTTTCTTTCATGAAGTAAAGAGCTAAGCCAGGCAT
ATATtATTCTTAtGTTTTTCTTTtATaAAGTAAAGAGCTAAGCCAGaCAT

TACTTAGATTTATT------ATCTTCTCTGTATCCATAAAATGAAGGAACTA
TACTTAGATTTATT------ATCTTCTCTGTATCCATAAAATGAAGGAACTA
TACTTAGATTTATTgctattATCTTCTCTGTATCCAcAAAATGAAGGAAtTA
```

chr9:12757578-12757740
Alignment
Human, chimp, rhesus, Tigger3(Golem) consensus, donor

```
acgaaactccgtctgaaaaaaaatatatatatataatggaaggtgaaaaa
GCGAAACTCCGTCTGAAAAAAtATATATATATATAATGGAAGGTGAAAAA
                                     ggaaggtgaaaaa

ttcttatc--ctagtaacattgtagctatcctaatgaagtggcacaatgcat
TTCcTATCacCTAGTgACATTGTAGCTATCCTAATGAAGTGGCACAATGCAT
ttcctataacctagtgacattgtagctatcccaatgaggtggcacaatgcat

tactttttctatatttagatatgtttagatacaaacataTCTTTATGTTG
TACTTTTTCTATATTTAGATATGTTTAGATACAAACATATCTTTATGTTG
tactttttctatatttagatatgtttagatacaaaaata-----------
taccttttctatgtttagatatgtttagatacacaaata-----------

TACGTATCTGTATGTTTTATGTTGTAATTGTATGCAGTGATGTAAAGTAG
TACGTATCTGTATGTTTTATGTTGTAATTGTATGCAGTGATGTAAAGTAG
--------------------------------------------------
--------------------------------------------------
                       GTAATCGTATGGAGTGATGTAAAGTAG

CCTGTCATTTTTAATACTTTTTTTGTGATTAAGTAAGTTTGTATTTATTC
CCTGTCATTTTTAcTACgTTTTTTGTGATTAAGTAAGTTTGTATTTATTC
--------------------------------------------------
--------------------------------------------------
CCTGTCGTTTTTAATACTTTTTTTGTGATTAAGTAAGTTTGCGTCTATTC

TTTGCAGTGACTTTAAGTGTTCTTGGCTGTTCTTGGCTGTAAATATACAA
TTTGCAGcGACTTTAAGTGTTCTTGGCTGTTCTTGGCTGTAAATATACAA
--------------------------------------------------
--------------------------------------------------
TTTGCAGTGACTTTAAGTGTTCTTGGCTG

ACAtatatttagatatgtttagata-----cattgttttacagttgctgacagta
ACATATATTTAGATATGTTTAGATA-----CATTGTTTTACAGTTGCcGACAGTA
-------------------------cttaccattgttttacaattgctgacagta
-------------------------cttaccattgtgttacaattgcctacagta

ttcagtataggaacttgatgtacagttttgtagcctaggagcaataggcc
TTCAGTATAGGAACaTGATGTACAGTTTTGTAGCCTAGGAGCAAcAGGCC
ctcagtataggaacatgctgtacaggtttgtagcctaggagcaataggcc
ttcagtacagtaacatgctgtacaggtttgtagcctaggagcaataggct

atctcacataccctaagtgtgcagtaggctatccaatctatgtatgcata
ATCTCACATACCCTAAGTGTGCAGTAGGCTATCCAATCTATGTATGCgTA
atctcatatgccctaagtgtgcagtaggctgtccaatctatatatgtgga
ataccatatagcctaggtgtgtagtaggctataccatctaggtttgtgta

agtacactctatgatgtttgcacagcaactaaatcacctgatgatgcatt
AGTACACTCTATGATGTTTGCACAGCAACTAAATCACCTGATGATGCATT
agtatactctatgatgtttgcatagcaactaaatcacctgatgatgcatt
agtacactctatgatgttcgcacaacgacgaaatcgcctaacgacgcatt

tgtcagaatatattcctgccgttaagcgaaatgtgactATCTATGTTCAG
TGTCAGAATATATTCCTGCCGTTAAGCGAAATGTGACTATCTATGTTCAG
tgtcagagtatattcctgtcattaagcgacatgtgactATCTATGTTCAG
tctcagaacgtatccccgtcgttaagcgacgcatgact

AAGTAGAACTCTTAATAGTTTTGAAAGAGGACTAATGCCGATTTGTGGGT
AAGTAGAACTCTTAATAGTTTTGAAAGAGGACTAATGCCGATTTGTGGGT
AAGTAGAACTCTTAATAGTTTTCAAAGAGGACTAATGCCCGTTTGTGGGT
```

AATAGAAAAAATGTGGGTTTACAAATAAAATATATTTAtttta
AATAGAAAAAAcGTGGGTTTgCAAATAAAATATATTTATTTTA
AATGGGAAAAACATGGGTTTGCCAATAAAATATAtttatttta

```
chr10:9325611-9325766
Alignment
Human, chimp, rhesus, AluSx consensus, donor
ACACTGGAGAAGTCCCTCAGCTGTTCAGACCACAGATTCTGTAAAAAAAG
ACACTAGAAAAGTCCTTCAGCTGTTCAGACCACGGATTCTGTAAAAAGAG

GATAGCAGATGTCCCCAGATCTCCCAGTCCCCTCCCAAGTTTCAAGAATT
                     TCCCAGTCCCCTCCCAAGTTTCAAGAATT
GATAGCAGATCTCCCCAGATCTCCCAGTCCCCTCTCAAGTTTCAAGAATT

CTAAGGCTATCCTGGGGATATCAGTAGTCAGCGTAggctgggcgcggtgg
CTAAGGCTATCCTGGGGATATCAGTAGTCAGTGTAggctgggcacggtgg
CTAAGGTTATCCTGGGGATATCAGTAGTCAGTGTAggccgggcacagtgg
                                   ggccgggcgcggtgg

ctcacgcctgtaatcccaacactttgggaggccgaggcaggcggatcact
ctcacgcctgtaatcccaacactttgggaggccgaggcaggcggatcact
ctcacacctgtaattccaacactttggaaggccatggcaggaggatcacc
ctcacgcctgtaatcccagcactttgggaggccgaggcgggcggatcacc

tgaggtcaggagttcaagaccagcctggccaacatggtgaaaccccatct
tgaggtcaggagttcaagaccagcctggccaacacggtgaaaccccatct
tgaggtcaggagttcaagaccagcctggccaacacagtgaaaccccgtct
tgaggtcaggagttcgagaccagcctggccaacatggtgaaaccccgtct
                                               TCT

ctacCCAGGGCTCAAATGTACCACTGCCAGGAGTGTTACAGGTTATTTGA
ctacCCAGGGCTCAAATGTACCATTGCCAGGAGTGTTACAGGTTATTTGA
ctac----------------------------------------------
ctac----------------------------------------------
ATACTCAGGGCTCAAATGTACCATTGCCAGGAGTGTTACAGGTTATTTGA

CATAAAACAGAGAAAAACCAGTAGCATCTTTTACAAAGCTCAGAAACTTA
CATAAAACAGAGAAAAACCAGTAGCATCTTTTACAAAGCTCAGAAACTTT
--------------------------------------------------
--------------------------------------------------
CATAAAACAGAGAAAAACCAGTAGCATCTTTTACAAAGCTCAGAAACTTT

TTCTCTCCTTTCCCCTTTTGATGTGGGGTCACTGGCCTCCTTAGCTCTGA
TTCTCTCCTTTCCTCTTTTGATGTGGGGTCACTGGCCTCCTTAGCTCTGA
--------------------------------------------------
--------------------------------------------------
TTCTCTCTTTTCCCCTTTTGATGTGGGGTCACTGGCCTCCTTAGCTCTGA

ACACCTTCAGA-------acaaaaattagccaggcgtggtgatgcacgcctgtaatc
ACACCTTCAGA-------acaaaaattagccaggcatggtgatgcacacctgtaatc
-----------taaaaattcaaaaattagccaggcgtggtgatgcacacctgtaatc
-----------taaaaatacaaaaattagccgggcgtggtggcgcgcgcctgtaatc
ACACCTTCAGA-------ACA


ccagctactcaggaggctgaggcacaagaattgcttgaacccaagaggca
ccagctactcaggaggctgaggcacaagaatcacttgaacccaagaggca
ccagctactcaggaggctgaggcacaagaatcgctcgaacccgagaggcg
ccagctactcgggaggctgaggcaggagaatcgcttgaacccgggaggcg


aagtttgcagtgagccaagatcccgccacagcactc-----------caggag----actgtgtc
aagtttgcagtgagccaagatcccaccacagcactc-----------caggag----actgtgtc
aaatttgcagtgagccaagatcccaccacagcactctagcctgggcgaaggag----actctgtc
gaggttgcagtgagccgagatcgcgccactgcactccagcctgggcgacagagcgagactccgtc
```

```
tcaaaaaaaaaaaa-TAGTAGTGAGTGTAAATGTCTATATCATAACAACGT
tcaaaaaaaaaaaaaTAGTAGTGAGTGTAAATGTCTATATCATAACAACAT
tcaaaaaaaaaaaa--GGTAGTGAGTGTAAATGTCTATATCATAACAATGT
tca

GTTCTAACAAAGATCTGTCAACCATGGATAATCCAGATTTTATTTGGCTA
GTTCTAACAAAGATCTGTCAACCATGGATAATCCAGATTTTATTTGGCTA
GTTCTAACAAAGATCTGTCAACCATGGATAATCCAGATTATATTTGGCTA

GTATAACGTTAGcat
GTATAACATTAGcat
GTATAACGTTAGCCt
```

```
chr12:213519-213618
Alignment
Human, chimp, rhesus, MER41B consensus, donor
AGGACACCCTTGAGTAAGGACTGTGAGTGACACTTATGGCCCTTGAGAGA
AGGACACCCTTGAGTAAGGACTGTGAGTGACACTTATGGCCCTTGAGAAA
AGGATACCC--GAGTAAGGACTGTGAGTGACACTTGCGGCCCTTGAGAAA


TATTACTAGAAATACTACCCTAGTGATATTTGCCTGGGTTCCCTGGCAGC
TATTACTAGAAATACTACCCTAGTGATATTTGCCTGGGTTCCCTGGCAGC
TATTACTAGAAATATTACCCTAGTAATATTTGCCTGGGTTCCCCGGCAGC


TGTGAACCCAAGAAGGGACAGTGTCAGACACCTTATGACCACAAACAGCT
TGTGAACCCAAGAAGGGACAGTGTCAGACACCTTATGACCACAAACAGCT
TGTGAACCCAAGAAGGGACAGTGTCCGACACCTTTTGACCACAAACAGCT


GCTAACATgtcagaggcatttgaaccacagcggctccagcttgaataggg
GCTAACATgtcagaggcatttgaaccacagcggctccagcttgaataggg
GCTAACACgtcagaggcatttgaaccagagcgactccagcttgaacaggg
       tgtcagaggcgtttgaaccagagcaactccatcttgaataggc

gctgggtaaaataaagctgagacccactggggctgcattcccaggaggta
gctgggtaaaataaagctgagacccactggggctgcattcccaggaggta
g-tgggtgaaataaggctgagacctactggg-ctgcgttcccaggaggtg
gctgggtaaaatraggctgaracctactggg-ctgcattcccagacggtt


a-ggcattctaagtcacaggatgagacaggaggtcgtcacctcacaagata
a-ggcattctaagtcacaggatgagacaggaggttgtcacctcacaagata
a-ggcattctcagtcacgggatgagacaggaggtcgtcacgtccccagaca
aaggcattctaagtcacaggatgagataggaggtcggcac-----aagata


ccagtcataaagaccttgctgataaaacaggtttcagtaaagaagctggc
ccggtcataaagaccttgctgataaaacaggtttcagtaaagaagccggc
caggtcataaagaccttgctgataaaacaggtttcagtaaaggagacagc
caggtcataaagaccttgctgataaaacaggttgcagtaaagaagccggc


caaaacccgtcaaaaccaagatggcaacagaagtgaactctggtcCTAGA
caaaacccgccaaaaccaagatggcaacagaagtgacttctggtcctaga
caaaacccgcgaaaaccaagatggcgacagaagtgacctctggtc-----
yaaaacccaccaaaaccaagatggccacgagagtgacctctggtc-----
                                             CAAGA


GGAGGAGCTCTAGGTTGCACGCTCACTCCCCCGGCAGGATGGTCTCTGCG
ggaggagctctaggttgcacactcactcccccggcaggatggtctctgtg
--------------------------------------------------
GGAGGAGCTCTAGGTTGCACACTCACTCCCCCGGCAGGGTGGTCTCTGCA


CCCAGGAGGCAGAGGCAGGGCATAACCGGGTAACCACACTTCAGGG--cctc
cccaggaggcagaggcagggcataaccgggtaacaacacttcaggg--cctc
----------------------------------------------ttcctc
----------------------------------------------gtcctc
CCCAGGAGGCAGAGGCAGGGCATAACCGGGTAACAACACTTCAGGG--CCTC

accaccaattatatgtaaattattatgcattagcatgc-tgaaagacactc
accaccgattatatgtaaattattatgcattagcatgc-tgaaagacactc
accattctttacatgcaaattagtatgcgttagcatgcctgaaagaccctc
```

```
actgctcattatatgytaattataatgcattagcatgc-taaaagacactc

ccaccagctccatgacagtttacaaatgccatggcaacgtcaggaagtta
ccaccagctccatgacagtttacaaatgccatggcaacgtcaggaagtta
ccaccagtgccatgacagtttacaaatgcgaagg----------gagtta
ccaccagcaccatgacagtttacaaatgccatggcaacgtcaggaagtta

acctctatggtctaaaatgggagg-gaaccctcagttatgggaattgccca
acctctatggtctaaaatgggggg-gaaccctcagttatgggaattgccca
acctctatggtctgaaatcgggggagaaccttcagttttgggaattgccca
ccctatatggtctaaaaaggggag-gaaccctcagttccgggaattgcccg

cacctttcc-aggaaactaatgaataatccgccccttatttagcatataat
cacctttcc-aggaaactaatgaataatccgccccttatttagcatataat
cacctttcc-ggaaaactgatgaataacccaccccttgtttagcatataat
cccctttcctkgaaaaytcatgaataatccaccccttgtttagcatataat

caagaaataaccataaaaatgggcaaccagtagccctggggctgccctgc
caagaaataaccataaaaatgggcaaccagtagccctggggctaccctgc
cgagaaataaccataaaaacgggcaaccagtagccctggggc-atcctac
caagaaataaccataaaaatrggcaaccagcagccctcggg

ctatggagtagccattcttcattcctttt--atttcattttattttattttat
ctatggagtagccattctttattcctttttatttcattttattttattttat
ctatggagcagccattctttattcctttt--attttattttattttattttat

ttcattttattttattttatttcattttattttattttagagacagagtc
ttcattttattttatttt--------------------agagacagagtc
tttattttattttatttt----------------------aGAGAgagtc

tcactctgttgcccaggctagagtgcagtggcgcgatctca
tcactctgttgcccaggctagagtgcagtggcgcgatctca
tcactctgttggccaggctggagtgcagtggcaccatctca
```

Hominoid-specific RDs - donor and acceptor greater than 5 kb from each other or on other chromosome

chr3:151723788-151724097
Alignment
Human, chimp, rhesus, consensus, donor

```
aaatctttaacacatgttcattttatatctgtatagagtcataattttac
AAATCTTTAACACATGTTCATTTTATATCTGTATAGAGTCATAATTTTAC
AAATCTTTAACACATTCATTTTATATCTGTATAGAG--TCATAATTTTAC

ccctctgtgaaaatcatccttagccaggcacagtgactcgtgcctataat
CCCTCTGTGAAAATCATCCTTgGCCAGGCACAGTGACTCGTGCCTATAAT
CtCTtTtTGAAAATCATCCTTgGCCAaGCACAGTGACTCaTGCCTATAAT

tccagcactttgggaggccaaggcgggaggatcacttgagcccaggagtt
TCCAGCACTTTGGGAGGCCAAGGCGGGAGGATCACTTGAGCCCAGGAGTT
TCCAGCACTTTGGaAGGCCAAGGCGGGAGGATCACTTaAGCCCAGGAGTT

tgagaccagcctggacaacatagtgaaTTCTCTGTGTTAATTTCACTTTT
TGAGACCAGCCTGGACAACATAGTGAATTCcCTGTGTTAATTTCACTTTA
TGAGACCACCTGGACAACATAGTGAAA-----------------------
                                 GTGTTAATTTCACTTTT

TAAAAAAAAAATTTTTCCCCTCGTACAAACTCTCAGTCTCCCTGTGACTG
AAAAAAAAAAttTTTTTCCCCTCGTACAAACTCTCtGTCTCCCTGTGACTG
--------------------------------------------------
TAAAAAAA-CATTTTCCCCCTCATACAAACTCTCAGTCTCCCTGTGACTG

AACATCACCAATGATGTTCTGAAAGACTCAGGTGAAAAGTGAAGGATTGA
AACATCACCAATGATGTTCTGAAtGACTCAGGTGAAAAGTGAAGGATTGA
--------------------------------------------------
AACATCGCCAATGATGTTCTGAATGACTCAGGTGAAAAGTGAAGGATTGA

GGTAGAATTTGTTGTCTGAGAACAGATCTAACAGCCAGAACAAATAAGTC
GGTAGAATTTGTTGTCTGAGAACAGATCTAACAGCCAGAACAAATAAGTC
--------------------------------------------------
GGTGGAATTTGTTGTTTGAGAACAGATCTAACAGCCGGAACAAATAACTC

CTGCATAGGAAGCCAAGTTGGGAGCCAGCGAGGAAGGATAAGGGGGCAGG
CTGCATAGGAAGCCAAGTTGGGAGCCAGCGAGGAAGGATAAGGGGGCAGG
--------------------------------------------------
TTGCATAGGAAGCCAAGTTGGGAGCCAGAGAGGAAGGATAAGGGGGCAGG

GACTCAAAGTAGGTGGTCCACTTTCCCTTTGGGGGTAATTTCATCCTTTG
GACTCAAAGTAaGTGGTCCACTTTCCCTTTGGGGGTAATTTCATCCTTTG
--------------------------------------------------
GACTCAAAGTAGGCGGTCCACTTTCCCTTTGGGGGTAATTTCA

GGGGTAATTTCA-----TCTCTACAAAAAATAAAAATAGAAATaaaaataaaaaa
GGGGTAATTTCA-----TCTCTACAAAAAATAAAAATAGAAATAAAAATAAAAAA
------------ccccatgtctacaaaaaataAAAATAAAAAA------------

ttagccagatgtgatggcacatgcctgtagtcccagctactcaagaggct
TTAGCCAGATGTGATGGCACATGCCTGTAGTCCCAGCTACTCAAGAGGCT
TTAGCCAGATGTGATGGtacac----GTAGTCCCAGCTACTtgAGAGGCT

gaagggggagaattgcttg-------gaggtcagggctgcagtgagccgtgattgtg
GAAGGGGGAGAATTGCTTG-------GAGGTCAGGGCTGCAGTGAGCCGTGATTGTG
GAAGGGGGAGAATTGCTTGaggccagGAGGTCAGGGCTGCAGTGAGCtGTGATTGTG

ccactgcattcctgcctgagtgacagagcAGTGCCACTGTGACAGAGTGT
CCACTGCATTCCTGCCTGAaTGACAGAGCAGTGCCACTGTGACAGAGTGT
```

CCACTGCATTCCTGCCTGAGTGACAGAG

chr3:153769536-153769606
Alignment
Human, chimp, rhesus, consensus, donor

```
ATATTAACCTGTACCTGAAGG---TCTTAGAAGTAGATGAAGgactgggcaca
ATATTAACCTGTACCTGAAGa---TCTTAGAAGTAGATGAAGGACTGGGCACA
ATATTAACTGTACCTGAAaGccatTCTTAGAAGTAGATGAAGGACgGGGCACA

gtcgctcacacctgtaatcccatcactttgggaggccaaagtgagaggat
GTtGCTCACACCTGTAATCCCATCACTTTGGGAGGCCgAAGTGAGAGGAT
GTCGCTCACgCCTGTAATCCCATCACTTTGGaAGGCCAAAGTGAGAaGAT

cccttgagcctgggagtttgagaccagcctggcaacatagggagacctca
CCCTTGAGCCTGGGAGTTTGAGACCAGCCTGGCAACATAGGGAGACCTCA
CCCTTGAGCCTGGGAGTTTGAGACCAGCCTGGCAgCATAGGGAGACCTCA

tctctatttaaaagaaaaaaattacctagatctgtggcacgtgcctgtgg
TCTCTATTTAAAAGAAAAAAATTACCTAGATCTGTGGCAtGTGCCTGTGG
TCTCTATTTcaaAAAAGAAAATTACCTgGAagTGTtGCACaTGCCTGTGG

tcccagctactcaggagactgaagcgagataattgcttgagcctgggagg
TCCCAGCTACTCAGGAGgCTGAAGCGAGATAATTGCTTGAGCCTGGGAGG
TCCCAGCTACTCAGGAGgCTGcAGtGAGATgATcGCTTGAGCCTGcGAGG

tcgaggctgcagtgagctataattgttccactTCGGAGAGGAGGACAAGA
TCGAGGCTGCAGTGAGCTATAATTGTTCCACTTCGGAGAGGAGGACAAGA
TCGAGGCTGCAGTGAGCTATAATTGTgCCACTgC----------------
                                  GGAGAGGAGGACAAGA

ACAGAGCCTGAAGAAGTGAAATAAATATGGGTGTGGCTACATGAAAAAAA
ACAGAGCCTGAAGAAGTGAAATAAATATGaGTGTGGCTACATGAAAAAAA
--------------------------------------------------
ACAGAGCTTGAAGAAGTGAAATAAATATGGGTGTGGCTACATGAA

AAAAa--ttgttccacttcactccagcctgggtgaaagaacaagaccctgtc
AAAAAaaTTGTTCCACTTCACTCCAGCCTGGGTGAAAGAACAAGACCCTGTC
-------------------ACTCCgGCCTGGGTGAcAGAACAAGACCCTGTC

tcaaaaaagaaaagaaaagaaaaTAGATGAAGGGACA
TCAAAAAAGAAAAGAAAA-----TAGATGAAGGGACA
TCAAAAAgGAAAAGAAAAGAAAATAGATGAAGGGACA
```

chr8:14556636-14556714
Alignment
Human, chimp, rhesus, consensus, donor

```
cactcaataaatatGAGCTTtactactactgctaccactgcttctgctac
CACTCAATAAATATGAGCTTTACTACTgCTGCTACCACTGCTTCTGCTAC
CACTCAATAAATATGAGCTTTACTACTACTaCTACCACTGCTTCTGCTAC

catcattacttcttttgttgttgttttgttttgagacagagtctcgctct
CATCATTACTTCTTTTGTTGTTGTTTTGTTTTGAGACAGAGTCTCGCTCT
CATCATTACTTtTTTTtttttTTTTtTTTTc-GAGACAGAGTCTCaCTCT

gtcgcccaggctggaatgcaatggcgtgatctcggctcactgaaactact
GTCGCCCAGGCTGGAATGCAATGGCGTGATCTCGGCTCACTGAAACTACT
cTtGCCCAGGCTGGAgTGCAATGatGcaATCTTtGGCTCACTGcAACcAtc

gcttcccgagttcaagcgattctcctgcctcagcctcccaagtagctggg
GCTTCCCGAGTTCAAGCGATTCTCCTGCCTCAGCCTCCCAAGTAGCTGGG
GCcTCCtGgGTTCAAGCGATTCTCCTGCCTCAGCCTCCCAAGTAGCTGGG

attataggcacgcaccgccatgcccagctaattttttttttttgtaTT
ATTATAGGCgCGCACCaCCATGCCCgGCTAATTTTTTTTTTTTTGTATT
ATTAcAGGCgCGCACCaCCAcGCCtgGCTAATT-----------------
                                             tgTAT-

TTGAAATAACTTTAGGTGTTTTATTTCCACACATGTAAAGGTCTCATAAT
TTGAAATAACTTTAcGTGTTTTATTTCCACAaATGTAAAGGTCTCATAAT
--------------------------------------------------
TTGAAATAACTTTACGTGTTTTATTTCCACAAATGTAAAGGTCTCATAAT

TTTGTCATAAATCATGCACAAAGAACAAtttctatttttagtagagatgt
TTTGTCATAAATCATGCACAAAGAACAATTTCTATTTTTAGTAGAGATGT
----------------------------TTTgTATTTTTAGTAGAGATGg
TTTGTCATAAATCACGCACAAAGAACAC

ggtttcactatattggtcaggctggtttcgaactcctgaccttgtgatct
GGTTTCACTAcATTGGTCAGGCTGGTTTCGAACTCCTGACtTTGTGATCT
GGTTTCACT--gTTGGcCAGGCTGGTTTCaAACTCCTGtgatccaccca-

atttttctcagcctcccaaagtgctgggactacaggcaggaagccactgc
ATTTTTCTCAGCCTCCCAAAGTGCTGGGACTACAGGCAGGAGC-CACTGC
-----cctcgGCCTCCCAAAGTGCTGGGACTACAGGCgtgatC-CACcGC

acTGGCCCCATCATTACTTTTAATGC
ACTGGCCCCATCATTACTTTTAATGC
ACTcGCCCCATCATTACTTTTAATGC
```

chr2:70183106-70183725
Alignment
Human, chimp, rhesus, consensus, donor

```
acctcaggtgatccacccacctcaacctcccaaagtgctaggattacagg
ACCTCAGGTGATCCACCCACCTCAACCTCCCAAAGTGCTgGGATTACAGG
ACCTCAGGTGATCCACCCACCTCAACCTCCCAAAGTGCTAGGATTAC-GG

cgtgagccaccacacccagccacatcttagtattattataaaaatagttC
tGTGAGCCACCACACCCAGCCACATCTTAGTATTATTATgAAAATAGTTC
CGTGAGCCACCACACCtAaCCACATCTTAGTgTTATTATGAAAATAGTT-

CGCCGCCATCTTTCTTCCTGGCAGGGGCCGACGCAGGGACCGGCGCGGGG
CGCCGCCATCTTTCTTCCTGGCAGGGGCCGACGCAGGGACCGGCGCGGGG
--------------------------------------------------

GTGAGAGCGCGCGGCCGGATTCACCACAACATGGCAACTCTTTTTATAAG
GTGAGAGCGCGCGGCCGGATTCACCACAACATGGCAAaTCTTTTTATAAG
--------------------------------------------------
                 GATTCACCACAACATGGCAAATCTTTTTATAAG

GAAAATGGTGAACCCTATGCTATATCTCAGTCGTCACACAGTGAAGCCTC
GAAAATGGTGAACCCTATGCTATATCTCAGTCGTCACACAGTGAAGCCTC
--------------------------------------------------
GAAAATGGTGAACCCTCTGCTCTATCTCAGTCGTCACACGGTGAAGCCTC

GAGCCCTCTCCACATTTCTATTTGGATCCCTTCGAGGTGCAGCCCCCGTG
GAGCCCTCTCCACATTTCTATTTGGATCCCTTCGAGGTGCAGCCCCCGTG
--------------------------------------------------
GAGCCCTCTCCACATTTCTATTTGGATCCATTCGAGGTGCAGCCCCCGTG

GCTGTGGAACCCGGGGCAGAAGTGCGCTCACTTCTCTCACCCGGCCTCCT
GCTGTGGAACCCGGGGCAGAAGTGCGCTCACTTCTCTCACCCGGCCTCCT
--------------------------------------------------
GCTGTGGAACCCGGGGCAGCAGTGCGCTCACTTCTCTCACCCGGCCTCCT

GCCCCACCTGCTGCCCGCGCTGGGGTTCAAAAACAAGACTGTCCTTAAGA
GCCCCACCTGCTGCCCGCGCTGGGGTTCAAAAACAAGACTGTCCTTAAGA
--------------------------------------------------
GCCCCATCTGCTGCCTGCGCTGGGGTTCAAAAACAAGACTGTCCTTAAGA

AGCGCTGCAAGGACTGTTACCTGGTGAAGAGGCGGGGTCAGTGGTACGTC
AGaGCTGCAAGGACTGTTACCTGGTGAAGAGGCGGGGTCAGTGGTACGTC
--------------------------------------------------
AGCGCTGCAAGGACTGTTACCTGGTGAAGAGGCGGGGTCGGTGGTACGTC

TACTGTAAAACCCATCCGAGGCACAAGCAGAGACAGATGTAGACCCTTTC
TAtTGTAAAAtCCATCCGAGGCACAAGCAGAGACAGATGTAGACCCTTTC
--------------------------------------------------
TACTGTAAAACCCATCCGAGGCACAAGCAGAGACAGATGTAGACCCTTTC

CCTCCAGAGTCACGCACATACTCGTCATCGCGTCTCTTGGGAGAATGGTT
CCTCCAGAGTCACGCACATACTCGTCATCGCGTCTCTTGGGAGAATGGTT
--------------------------------------------------
CCTCCAGAGTCACGCACATACTCGTCATCGCATCACTTGGGAGAATGGTT

GTATCTTATGGAAGGAATTATCACATCAAGGAGTCAGGGGAAAGTGACTG
GTATCTTATGGAAGGAATTATCACATCAAGGAGTCAGGGGAAAGTGACTG
--------------------------------------------------
GTATCTTATGGAAGGAATTATCACATCAAGGAGTCAGGGGAAAGTGACTG

GAAGCAAACGCCCTAAAAGTTACCCATCACGTTTCAGTGTAAATGAGTAA
GAAGCAAACGCCCTAAAAGTTACCCATCACGTTTCAGTGTAAATGAGTAA
--------------------------------------------------
```

GAAGCAAACGCCCTAAAAGTTACCCATCACGTTTCAGTGTAAATGAGTAA

CTATAGAAGACATTGCATTATCTTATTTCCAAAATGTTCCAATTAAAAAA
CTATAGAAGACATTGCATTATCTTATTTCCAAAATGTTCCAATTAAAAAA

CTATAGAAGACATTGCGTTATCTTATTTCCAAAACGTTCCAACTAAAAAA

CATTTTCCTATTAAAA**AAAA**gaaaatagttttgacctctcagacccctga
CATTTTCCTATTtAAAAAAAGAAAATAGTTTTGACCTCTCAGACCCCTGA
------------------------------TTaACCTCTCAGACCCCTGA
CATTTTCCTATTAAAA

aaggatctgagggcgccctagggccccccaccactctttgagaaatgctA
AAGGATCTGAGGGCGCCCTAGGGCCCCCCACCACTCTTTGAGAAATGCTA
AAaGATCTcAGGaaGCCCTAGGGgCCCCCACCACTCTTTGAGAAAcGCTA

CTCTAcagaaagattttac
CTCTACAGAAAGATTTTAC
CTgTgCAGAAAGATTTTAC

chr13:58663684-58663759
Alignment
Human, chimp, rhesus, L1MA8 consensus, donor

```
gactttgtctcaaaaaaaaaataa-------ttaattaatcaaaaaaatacaataca
GACTTTGTCTCAAAAAAAA---------TAATTAATTAATCAAAAAAATACAgTACA
GACTcTGTCTCAAAAAAATAataatgATTAATTAATaAAAAAataaAATACAATACA
                                            aaaaatacaata--

ttgttaactgtagtcaccatattgcagagtagatctcttTTTTTTTAAGC
TTGTTAACTGTAGTCACCATATTGCAGAGTAGATCTCTTTTTTTTTAAGC
TTGTTAACTGTAGTCACCATATTGCAcAGTtGATCTCT------------
ttattaactatagtcaccatgctgtacaaatagatctc------------
                                             TAAGC

TACCTTTCTTTATTGAAATCAATTAGAATTGATAGACATTTGAATGCATA
TACCTTTCTTTATTGAAATCtATTAGAATTGATAGACATTTGAATGCATA
--------------------------------------------------
TACCTTTCTTTATTGAAATAAATTACAATTGATGGACATTTGTGTGCATA

TTTTACATACCTTT----------GTtcccatctaactgaaattttgtatcctttttcca
TTTTACATACCTTT----------GTTCCCATCTAACTGAAATTTTGTATCCTTTTTCCA
--------------tgaatttatggTTCCCATCTAACTGAAATTTTGTATCCTTTTTCCA
--------------tgaacttattcctcctatccaactgtaattatgtattctttgacag
TTTTACAGACTTTT----------GT

gtatctctccaa---ctcccaaaccatggcccctggtagccagcactctactc
GTATCTCTCCAA---CTCCCAAACCATGGCCCCTGGTAGCCAGCACTCTACTC
aTATCTCTCCAA---CTCCCAAACCATGGCCCCTGGTAGCCAGCACTCTACTC
acatctctccaaatcctcccactccccagcccctggtaaccaccattctactc

tctgcttctataagccaaacatttttagatttcacctataagtgagatca
TCTGCTTCTATAAGCCAAACATTTTTAGATTTCACCTATAAGTGAGATCA
TCTGCTTCTATgAGCCAAACATTTTTAGAcTTCACCTATAAGTGAGATCA
tctgcttctatgagttcaactttttagattccacatataagtgagatca

tgcagtatttgtctct--gtgcccagcttctttgacttaacatgatatcctc
TGCAGTATTTGTgtCTCTGTGCCCAGCTTCTTTGACTTAACATGATATCCTC
TGCAGTATTTGTgtCTCTGTGCCCAGCTTCTTTGACTTAACgTGATATCCTC
tgcggtatttgtctttctgtgcctggcttatttcacttaacataatgtcctc

caggctcatccatactgttgcaactgaaaacaaatttcactttttttaag
CAGGCTCATCCATACTGTTGCAACTGAAAACAAATTTTCACTTTTTTTAAG
CAGGtTCATCCATACTGTTGCAACTGAAAACAAATTTTCACTTTTTTTAAG
caggttcatccatgttgtcgcaaatgacaggatttccttctttttaagg

ttgaatagtattccattatgaacatataccacattttctttcttcattca
TTGAATgGTATTCCATTATGAACATATACCACATTTTCTTTaTTCATTCA
TTGAATAGTATTCtgTTATGAACATATACCACATTTTCTTTaTTCATTCA
ctgaatagtattccattgtgtatatgtaccacattttctttatccattca

tccgttgatggacacttagg---ttgattttgtatcttggctattggcaaata
TCCGTTGATGGACACTTAGG---TTGATTTTGTATCTTGGCTATTGGCAAATA
TCCGTTGATGGACACTTAGGaggTTGATTTcaTATCTTGGCTATTGGCgAATA
tccgttgatggacacttagg---ttgattccatatcttggctattgt-gaata

gtgctgcaataaacatgagagtacagatatctcttcaaaata---attttatt
GTGCTGCAATAAACATGAcAGTACAGATATCTCTTCAAAATA---ATTTTATT
tTGCTGCAATAAACATGAGAGTgCAGATATCTCTTCAAAATA---ATTTTATT
atgctgcaatgaacatgggagtgcagatatctctttgacatactgatttcatt

tcctttggatatataaccagtagtgggattgctggatcatatggtaattc
TCCTTTGGATATATAACCAGTAGTGGGATTGCTGGATCATATGGTAATTC
```

TCCTTTGGATATATAACCAGTAGTGGGATTGCTGGAgCATATGtTAATTC
tcctttggatatatacccag-agtgggattgctggatcatatggtagttc

tatttttagctttctaaggaacttccatactgtattccatgatggctatg
TATTTTTAGCTTTCTAAGGAACTTCCATACTGTATTCCATGATGGCTATG
TATTTTTAGCTTTCTAAGGAACTTCCATACTGTATTCCATGATGGCTATG
tattttaatttttgaggaacctccatactgttttccataatggctgta

ctaacttgcattcccaagcaaaggtt-taaagtttccctttctccacatc
CTAACTTGCATTCCCAAGCAAAGGTT-TAAAGTTTCCCTTTTCTCCACATC
CTAACTTGtATTCtCAAGCAgAGGTT-cAAgGTTTCCCTTTTCTCCACtTC
ctaatttacattcccaccaacagtgtgcaagggttccctttctccacatc

ctcgccaaaacttgttctctcatctctttttgataaaagccattgtaaca
CTCGCCAAAACTTGTTCTCTCATCTCTTTTTGATAAAAGCCATTGTAACA
CTtGCCAAAAtTTGTTCTCcCATCTCTTTTTGATAAAAtCCATTGTAACA
ctcccaacacttgttatctttcg-tctttttgataatagccattctaaca

agtatgaggtgatatctcattacggttttgacttgcatttctctgatgat
AGTATGAGGTGATATCTCATTAtGGTTTTGACTTGCATTTCTCTGATGAT
AGTATGAGGTGAcATCTCATTAtGGTTTTGAtTTtCATTTCTCTGATGAT
ggtgtgaggtgatatctcattgtggttttgatttgcatttccctgatgat

tagtgatgttgagtatttatacctgttggcatttatatatcttcctttga
TAGTGATGTTGAGTATTTATACCTGTTGGCATTTATATATCTTCCTTTGA
TAGTGATGTTGAGTATTTATACCTGTTGGCATTTATATATCTTCCTTTGA
tagtgatgttgagcattt

gaatgtctgttcaggcactttgcccatttttttaattgggttatttgtttt
GAATGTCTGTTCAGGCACTTTGCCCATTTTTTAATTGGGTTATTTGTTTT
GAATGTCTGTTCAGGaACTTTGCCCATTTTTTAATTGGGTTATTTGTTTT

ctagccactgaattgttggagttcctataatattttggatattaactcct
CTAGCCACTGAATTGTTGGAGTTCCTATAATATTTTGGATATTAACTCCT
CTAGCCACTGgATTGTTGGAGTTCCTATAATATTTTGGATATTAACTCCT

tgtatcagatgtttggttttgcaaatatttctcccatttcaccagttgtc
TGTATCAGATGTTTGGTTTTGCAAATATTTCTCCCATTTCACCAGTTGTC
TGTATCAGATGTTTGGTTTTGCAAATATTTCTCCCATTTCACCAGTTGTC

tcttcactccattaactgtttgctttgctatgcagaaactgattaatttT
TCTTCACTCCATTAACTGTTTGCTTTGCTATGCAGAAACTGATTAATTTT
TCTTCAtTCCATctACTGTTTcCTTTGtTATGCAGAAACTGATTAATTTT

GAGtcaagcttcagccagtcatagatagccaactagcccactggttaaat
GAaTCAAGCTTCAGCCAGTCATAGATAGCCAACTAGCCCACTGGTTAAAT
GAaTCAAGCTTCAGCCAGTCATAGATAGCCAACTAGCCCAaTGGTTAAAT

aatttggggcctttcatcagattatacccatatagggcaagggctcagct
AATTTGGGGCCTTTCATCAGATTATACCCATATAGGGCAAGGGCTCAGCT
AATTTGGGG

chr10:38169932-38170382
Alignment
Human, chimp, rhesus, L1MC3 consensus, donor

```
ggcacacacctgtaatcccagctactcgggaggctgaggaaggagaatca
GGCACACACCTGTAATCCCAGCTACTCGGGAGGCTGAGGAAGGAGAATCA
GGCACACACCaGTAATCCCAGCTACTCGGGAGGCTGAGGAAGaAGAATtg

cccaaaccggaaggcagaggttgcagtgagccgagattatgccactgcac
CCCAAACCGGAAG-CAGAGGTTGCAGTGAGCCGAGATTATGCCACTGCAC
CCCAAACCGGAAGGCAGAGGTTGCAGTGAGCtGAGATcATGCCACTGCAC

tccagcctggacaa--gagtgagactatgtctc---------aaaaaaagaaaaaagaaaa
TCCAGCCTGGACAA--GAGTGAGACTATGTCTCaaaaaaaaaAAAAAAAGAAAAAAGAAAA
TCCAGCCTGGACAAcaGAGTGAGACTccGTCTc--------CAAAAAAAGAAAAAAGAAAg

aaagaaaaccagtgctcctttgaaaaccagctgcttctaggtctggc-------agg
AAAGAAAACCAGTGCTCCTTTGAAAACCAGCTGCTTCTAGGgCTGGC-------AcG
AAAGAAAACCAGTGCTCCTTTGAAAACCAGCTGCTTCTAGGgCTGGggcagacCAcG

tatataatgagcctgaaatcacttctggtaccagaaaatgagaaagtact
TATATAATGAGCCTGAAATCACTTCTGGTACCAGAAAATGAGAAAGTACT
TATATAATGAGCCTGAAATCACTTCTGGTACCAGAAAATGAGAAAGTACT

caaatgaaaacacaatagtgggctatgtcaaagggacacaggaaccaatt
CAAATGAAAACACAATAGTGGGCTATGTCAAAGGGACACAGGAACCAAcT
CAAAcGAAAACACgATAaTGGGCTATGTCAAAGGGACACAGGAACCAAcT
                   ggggtatgtcaaagggacacagragccaact

gaaaacacagcctcatggccaa-gctgaagcagttggaacaacaaaat-aaC-------
GAAAACACAGCCTCATGGCCAA-GCTGAAGCAGTTGGAACAACAAAAT-AAC-------
GAAAACACAGCCTCATGGCCAA-GCTGAAGCAGTTGGAgCAACAAAATgAAC-------
gaaagagctccca-atggccaaagctggaacaatttgagcaacaaaataaattatrgta

AAGGTATATGGTTAAACCAAAGGAAGATTCAGATGAAAATC---CTTCTTTAA
AAGGTATATGGTTAAACCAAAGGAAGATTCAGATGAAAATCcttCTTCTTTAA
AAGGTA-----------------------------------------------
-----------------------------------------------------
    TATATGGCTAAACCAAAGGAAGATTCAGATGAAAATCCTTCTTCTTTAA

GACTGTCTTCTCAGCTTCTCTGTTTTCTAGCATCTTGTGTCATAGATGAA
GACTGTCTTCTCAGCTTCTCTGTTTTCTAGCATCTTGTGTCATAGATGAA
--------------------------------------------------
--------------------------------------------------
GACTGTCTTCTCCGCTTCTCTGTTTTCTAGCATCTTGTGTCATAGATGAG

AAGGCATATGTTCACATGATTTTT-TATAGCTATAAATAATCCCTCTCTAG
AAGGCATATGTTCACATGATTTTT-TATAGCTATAAATAATCCCTCTCTAG
---------------------------------------------------
---------------------------------------------------
AAGGCATATGTTCACATGATTTGTTTATAGCTATATATAATCCCTCTCTAG

AAGCTAGTAAAATTTTATCTGTGGATTTTTAAAATTTTTACCTGAATGTG
AAGCTAGTAAAATTTTATCTGTGGATTTTTAAAATTTTTACCTGAATGTG
--------------------------------------------------
--------------------------------------------------
AAGCTAGTAAAATTTTATCTGTGGATTTTTAAAATTTTTACCTGAATGTG

TCTAAATGTGCACATTTCTAAAGATGTGTATTTAATTTCCAAGAATGTTT
TCTAAATGTGCACATTTCTAAAGATGTGTATTTAATTTCCAAGAATGTTT
--------------------------------------------------
--------------------------------------------------
TCTAAATGTGCACATTTCTAAAGATGTGTATTTAATTTCCAAGAATGTTT
```

```
TGATGCTTATTTGTTTTTCAGTAATAGCCTGCTCTTGTTTTATGTGATGA
TGATGCTTATTTGTTTTTCAaTAATAGCCTGCTCTTGTTTTATGTGATGA
--------------------------------------------------
--------------------------------------------------
TGATGCTTATTTGTTTTTCAATAATAGCCTGCTCTTGTTTTATGTGATGA

TGATCTACTGAAACTTTCTGAGGCAATGACTAAAAGTATTTATTTTAAAG
---TCTACTGAAACTTTCTGAGGCAATGACTAAAAGTATTTATTTTAAAG
--------------------------------------------------
--------------------------------------------------
---TCTACTGAAATTTTCTGAGGCAATGACTAAAAGTATTTATTTTAAAG

TTATGTTCTATTTCCTAACTTAACTCTACTTCCTTTGGAGTAAATTTCTC
TTATGTTCTATTTCCTAACTTAACTCTACTTCCTTTGGAGTAAATTTCTC
--------------------------------------------------
--------------------------------------------------
TTCTGTTCTATTTCCTAACTTAACTCTACTTCCTTTGGAGTAAATTTCTC

TGACCACTTTGTTGTCCTTCTTTCATGCATTAAAAACAAAATTGTCTACC
TGACCACTTTGTTGTCCTTCTTTCATGCATTAAAAACAAAATgGTCTACC
--------------------------------------------------
--------------------------------------------------
TGACCACTTTGTTGTCCTTCTTTCATGCATTAAAAACAAAATTGTCTACC

---Aaaaaaaaaaaaaaaggtagtgttggattataac-caaagtataaaataac
aaaAAAAAAAAAAAAAAAGGTAGTGTTGGATTATAAC-CAAAGTATAAAATAAC
----------------------GTGTTGGATTATAAC-CAAAGTATAAgATAAC
----------------------gtattggattataacycaaagtataaaataaa

tagcaatgagttcatactaatataaataaatgcttaaataagtaaacaca
TAGCAATGAGTTCATACTAATATAAATAAATGCTTAAATAAGTAAACACA
TAGCAATGAGTTCATACTAATATAAATAAATGaTTAAAT----AAACACA
tatccatgagtccatactgatataaatgaatgattaaataaataaataaa

aaagaacagacaaatctcctgcacagaattcctaataatttatatggata
AAAGAACAGACAAATCTCCTGCACAGAATTCCTAATAATTTATATGGATA
AgAGAACAGACAAATCTCCTGCACAGAATTCCTAATAATTTATATGGATc

ctctgccatcaaggaggtgtagcgt-aactctccacttcattccaaagagt
CTCTGCCATCAAGGAGGTGTAGCGT-AACTCTCCACTTCATTCCAAAGAGT
CTCTcCCATCAAGGAGGTGTAGCGTtAAtTCTCCAgTTCATTCCAAAGAGT

acagttgaaaacggaggacaaaggagtaactttacagtggagaaacatga
ACAGTTGAAAACGGAGGACAAAGGAGTAACTTTACAGTGGAGAAACATGA
ACAGcTGAAAACaGAGGACAAAGGAGTAAaTTTACAGTGGAGAAACATGA

ccacactacctccaccaggta--------------atcaacagtgataaatcatgctcatagca
CCACACTACCTCCACCAGGTA--------------ATCAACAGTGATAAATCATGCTCATAGCA
CCACACTACCTCCACCAGGTAatcaacagtgatacATCAACAGTGATAAATCATGCTCATAGCA

ttttcccttgataggatgtgactgaatgacactacctctgtagtcttcct
TTTTCCCTTGATAGGATGTGACTGAATGACACTACtTCTGTAGTCTTCCT
TTTTCCtTTGATAtGAcaTGACTGAATGACACTACCTCTGTgGTCTTCCT

ctaagaaaccctaacctcagtctaataatgaggaagacagtagacaaata
CTAAGAAACCCTAAtCTCAGTCTAATAATGAGGAAGACAGTAGACAAATA
CTAAGAAACCCTAACCTCAGTCTAATcATGAGGAAGACAGTAGACAAATA

tcaactgagggacattctacaaaacacctgacttgaacccctcaaaactg
TCAACTGAGGGACATTCTACAAAACACCTGACTTGAACCCCTCAAAAtTG
TCAACTGAGGGACATTCTACAAAACACCTGACTTGAACCCCTCAAAAtTG

tcagggttatcaaaaacaaggaaagtctgagaaactgtcacagctaagaa
```

```
TCAGGGTTATCAAAAACAAGGAAAGTCTGAGAAACTGTCACAGCTAAGAA
TCAaGGTTATCAAAAACAAGGAAAGTCTGAGAAACTGTCACAGCcAAGAA

gagcctaaggagacatgatgactaagtagaatgtgggcatcctggaacag
GAGCCTAAGGAGACATGATGACTAAGTAGAATGTGGGCATCCTGGAACAG
GAGtCTAAGGAGACATGATGACTAAGTAGAATGTaGGCATCCTGGAACAG

aaaaattacattagggaaaaattaagaaaatcccaataaagtggtcatca
AAAAATTACATTAGGGAAAAATTAAGAAAATCCCAATAAAGTGGTaATCA
AAAAATTACATTAGGGAAAAATTAAGAAAATCCCAATAAAGTGGTCATtA

cttaataatatatcagtattgtttcattaattgtgacaaatgtactacat
CcTAATAATATATCAGTATTGTTTCATTAATTGTGACAAATGTACTACAT
CTTAATAATATATCAaTATTGTTTCATTAATTGTGACAAATGTACTACAT

taatatgttaat-------gaaactgtgtctatgggaggtatatggaaactctttgt
TAATATGTTAAT-------GAAACTGTGTCTAcGGGAGGTATATGGAAACTCTTTGT
TAATATGTTAATgatagggGAAACTGTGTtTATGGGAtGTATATGGAAACcgtttgT

actactgtcttcacaacttttccataaatctaaaactctcctaaagcaga
ACTACTGTCTTCACAACTTTTCCATAAATCTAAAACTCTCCTAAAGCAGA
ACT---GTCTTCACAACTTTTCCATAAATCTAAAACTCTtCTAAAGCAaA

aagtttatATAATAAGAAAGAAACATACAAGAGGACAAGATTAATGAAGT
AAGTTTATATAATAAGAAAGAAACATACAAGAGGACAAGATTAATGAAGT
AAGTTTAcATAATAAGAAAaAAACATgtAAGAGtACAAaATTAATGAAGT

GGaatgatgtcagcaaaatggagaggtaggtggcaccaagctcaggtccc
GGAATGATGTCAGCAAAATGGAGAGGTAGGTGGCACCAAGCTCAGGTCCC
GGAATtATGTCAGCAtAATGGAGAaGTAGGcGGCACCAAGCTCAGGTCCC
```

chr4:47986403-47986658
Alignment
Human, chimp, rhesus, consensus, donor

```
ccctTgtccttgctcctgctttctccacataaaatgcctgctcccacttt
CCCTTGTCCTTGCTCCTGCTTTCTCCACATAAAATGCCTGCTCCCACTTT
CCCTTGcCCTTGCTCCTGCTTTCaCCACATAAAATGCCTGCTCCCACTTT

gccttgagccatgaGactaatacattgctggtgggaatataaaatggtat
GCCTTGAGCCATGAGACTAATACATTGCTGGTGGGAATATAAAATGGTAT
GCCTTGAGCCATGAGACTAATACATTGCTGGTGGGAATATAAAATGGTAc

tgctactcaggaaaacagttggacaatttctttaaacattaaacatacaa
TGCTACTCAGGAAAACgGTTGGACAATTTCTTTAAACATTAAACATACAA
TGCTACTCAGGAAAACAGTTGGACAATTTCTTTAAACATTAAACATgCAA

ctaccttacaacctagcaattgaacccataggcatttctcccagagaaat
CTACCTTACAACCTAGCAATTGAACCCATAGGCATTTCTCCCAGAGAAAT
CTACCaTACAACCTAGCAATTGAACCCATAGGCATTTgTCtCAGAGAAAT

gaaaacttatgctcacacacataaaagctgtacacaaatgtttatagaag
GAAAACTTATGCTCACACACATAAAAGCTGTACACAAATGTTTATAGAAG
GAAAACTTATGCTCACACACAaAAAAaCTGTACACAAATGTTTAcAGAAG

gcttcttcctaatagccaaagtctgggaaaacctatgtgcccttctaaag
GCTTCTTCCTAATAGCCAAAGTtTGGGAAAACCTATGTGCCCTTCTAAAG
aCTTCTTCCTAATAGCCAAAGTtTGGGccAACCTATGTGCCCTTCTAAAG

gtgaatggttaaacaaaatgtgtgctgtgtatcatactaaggaatatcaa
GTGAATGGTTAAACAAAATGTGTGCTGTGTATCATACTAAGGAATATCA-
GTGAATGGTTAAACAAAATGTGTGCTGTGTATCATACTAAGGAgTATCA-

cccagcaataaaaagtaacaaactattgatccacacaacaacttagataa
CCCAGCAATAAAAAGTAACAAACTATTGATCCACACAACAACTTAGATAA
CtCAGCAAgAAAAAGTAACAAACTATTGATCCACACAACAgCTTAtATAA

atcttcagaggattatgctTTTATGCATGTAACTTCACAGTATAAAGGAA
ATCTTCAGAGGATTATGCTTTTATGCATGTAACTTCACAGTATAAAGGAA
ATCTTtcaAGGATTATGCT-------------------------------
                  CTTTTATGCATGTAACTTCACAGTATAAAGGAA

ATACAAACAATGTCTCCCTTCTCTAGTTTATTCCACTTACCCCAGTCCTC
ATcCAAACAATGTtTCCCTTCTCTAGTTTATTCCgCTTACCCCAGTCCTC
--------------------------------------------------
ATCCAAACAATGTCTCCCTTCTCTAGTTTATTCCGCTTACCCCAGTCCTC

CTAGGCTTCTGTGAATTTCAGAAAGCAAAAACAAAAAAAAAAAAA-CTTTT
CTAGGCTTCTGTGAATTTCAGAAAGCAAAAACAAAcAAAAAAAAA-CTTTT
---------------------------------------------------
CTAGGCTTCTGTGAATTTCAGAAAGCAAAAACAAACAAAAAAAAAACTTTT

TGTTCAAGGCGATTTAAAAAAACAACTTCACAAGATAGGGAGAATTGTGG
TGTTCAAGGCGATTTAAAAAAACAACTTCACAAGATAGGGAGAATTGTGG
--------------------------------------------------
TGTTCAAGGCGATTTAAAAAAACAACTTCACAAGATAGGGAGAATTGTGG

TGTGCTTGTCACATGTTACCAGTGGTAACAATTTTAATGACAAAAAAATC
TGTGCTTGTCACATGTTACCAGTGGTAACAATTTTAATGACAAAAAAATC
--------------------------------------------------
TGTGCTTGTCACATGTTACCAGTGGTAACAATTTTAATGACAAAAAAATC

CACTAATTCCAAATGTATAAACA---TTTaaaaaagccaataaagaaa-ggtta
CACTAATTCCAAATGTATAAACA---TTTtAAAAAGCCAATAAAAAA--GGTTA
-----------------------tagttt-AAAAAGCCAAccaaaaAAAGGTTA
```

CACTAATTCCAAATGCATAAACA

catactatatgattccatctacataacattcttgaaatgacaaaactgta
CATACTATATGATTCCATCTACATAACATTCTTGAAATGACAAAACTGTA
CATAtTATATGATTaCAcCTACATAAtATgCTTGtAATGACAAAACTGTA

gaagtggagaacagattcgtggttgctaggttttcctgaaggggtggggc
GAAGTGGAGAACAGATTCGTGGTTGCTAGGTTTTCCTGAAGGGGTaGGGC
GAtGTGGAcAACAGATTCGTGGTTGCTAGGTTTTCCTGAAGGGGTGGGGC

aagatggaagtggctatggctataaaagggtaacatgagtgacctttatg
AAGATGGAAGTGGCTATGGCTATAAAAGGGTAACATGAGTGACCTTTATG
AAGATGGAAGTGGCTATGGCTATAAAAGGcTAACATGAGTGACCTTTATG

gagatggaaatgtcctttttt-gactgtatcaatattgacctcctggctgt
GAGATGGAAATGTCCTTTTTT-GACTGTATCAATATTGACCTCCTGGCTGT
GAGATGGAAATGTtCTTTTTTtGACTaTATCAATATTGACaTCCTGGCTGT

gatgttgtactatagttttgcaaaggaaaactgggaaatgtgaactctct
GATGTaGTACTATAGTTTTGCAAAGGAAAACTGGGAAATGTGAACTCTCT
GATaTTGTACTAcAGTTTTGCAgAGGAAAACTGGtAAATGTGAActCTCT

--gattatttcttacaacccagatgtgaatctataattatctcaaaattaaa
--GATTATTTCTTACAACCCAGATGTGAATCTATAATTATCTCAAAATTAAA
CTGATTATTTCTTACAACCCAcATGTGAATCTATAATTATaTCAAAATTAAA

agtttaattAGATTTTTT-AATTAATAGCTAATAATGacaatagccaacat
AGTTTAATTAGATTTTTT-AATTAATAGCTAATAATGACAATAGCCAACAT
caTTTAATTAGATTTTTTaAATTAATAGCTAATAATGACAATAGCCAACAT

ttattgagtgtttaatgctaagtgtgatcctatgcagttgatcca
TTATTGAGTGTTTAATGCTAAGTGTGATCCTATGCAcTTGATCCA
TTATTGAGTGcTTAATGCTAAGTaTGATCCTATGCAcTTGATCCA

```
chr2:177997430-177997556
Alignment
Human, chimp, rhesus, consensus, donor
CCAATTCTAGAGATTTTTTTTTT-CCTTCAGCttttaagttctggggtaca
CCAATTCTAGAGATTTTTTTTTTtCCTTCAGCTTTTAAGTTCTGGGGTACA
CCAATTCTAtAGATTTTTTTTT--CCTTCAaCTTTTAAGTTCTGGGGTACA

tgtgcaggatgtgcaggtttgttacatagctaaacgtgtgacatggtggt
TGTGCAGGATGTGCgGGTTTGTTACATAGCTAAACGTGTGACATGGTGGT
TGTGCAGGATGTGCAGGTTTGTTACtTAGCTAAACGTGTGcCATGGTGGT

ttgctgcacagatcaacccatccccttggttattaggttattaagcccac
TTGCTGCACAGATCAACCCATCCCCTTGGTTATTAGGTTATTAAGCCCAC
TTGCTaCACAGATCAACCCATCaCCTTGGTTATTAGGTTATTAAGCCCAC

tatccattagctgtttttcctgatgctctccctccttctactgctcccct
TATCCATTAGCTGTTTTTCCTGATGCTCTCCtTCCTTCTACTGCTCCCCT
TATCCATTAGCTaTTTTTCCTGATGCTCTCCCTCtTTCcACcGCTCCCCc

tgatgggccccagtgtatgttgttcccctccttgtgttcatgtgttctca
TGATGGGCCCCAGTGTATGTTGTTCCCCTCCcTGTGTTCATGTGTTCTCA
TGATGtGCCCCgGTGTATGTTGTTCCCCTCCcTGTGTTCATGTGTTCTCA

ttgttcagctcccacttataagtgagaacatgcggtgtttggttttctgt
TTGTTCAGCTCCCACTTATAAGTGAGAACATGCGGTGTTTGGTTTTCTGT
TTGTTCAGCTCCCACTTATAAGTGAGAACATGCGGTaTTTGGTTTTCTGT

tcctgtgttagtttgctgaggataatggcttccagctctatccatgtccc
TCCTGTGTTAGTTTGCTGAGGATAATGGCTTCCAGCTCTATCCATGTCCC
TCCTGTGTgcaTTTGCTGAGGATAATGGCTTCtAGCTCTATCCcTGTCCC

tgcaaaggacatgattgtgttcctttctatggctgcatagtattccatgg
TGCAAAGGACATGATTGTGTTCCTTTCTgTGGCTGCATAGTATTCCATGG
TGCAAAGGACATGATTGTGTTCCTTTCTATGGCTGCATAGTATTCCATGG

tgtatatgtaccatattttctttatccagtcaatcattgatggacatttg
TGTATATGTACCATATTTTCTTTATCCAGTCAATCATTGATGGACATTTG
TGTATATGTACCATATTTTCTTT-TCCAGTCAATCATTGATGGgCATTTG

ggttgattccatgtgtttgctattgtgaattgtgctgcaatgaacatatg
GGTTGATTCCATGTGTTTGCTATTGTGAATTGTGCTGCAATGAACATATG
GGTTGATTCCATGTcTgTGCTATTGTGAATgGTGCTGCAATGAACATATG

catgcatatatctttataatagaatgatttTTTTTTTTGCCTGTACAACT
CATGCATgTATCTTTATAATAGAATGATTTTTTTTTT-GCCcGTACAACT
CATGCATgTATCTTTATAATAGAATGATT---------------------
                                    TTGCCCGTACAACT

ATTTAATGTCTGTATGCCTCTGAATAGTAATTAGGAGAAAACATATGGAA
ATTTAATGTCTGTATGCCTCTGAATAGTAATTAGGAGAAAACATATGGAA
--------------------------------------------------
ATTTAATGTCTGTATGCCTCTGAATAGTAATTAGGAGAAAACATATGGAA

ATGAAAATTATTTCAAATTTTAAAATCTTGAAACAGAAAGCAATATTTAT
ATGAAAAgTATTTCAAATTTTAAAATCTTGAAACAGAAAGCAATATTTAT
--------------------------------------------------
GCGAAAATTATTTCAAATTTTAAAATCTTGAAACAGAAAGCAATATTTAT

AATGTATCtattcctttgtgtatttacccagtaaggggactgctgggtca
AATGTATCTATTCCTTTGTGTATTTACCCAGTAAGGGGAtTGCTGGGTCA
------TaTATTCCTTTGTGTATaTACCCAGTAAGGGaAtTGCTGGGTCA
GATGTATCTA
```

```
aatggtatttctgcctctagatctttgaggaatcaccacactgtcttcca
AATGGTATTTCTGCCTCTAGATCTTTGAGGAATCACCACACTGTCTTCCA
AATGGTATTTCTGCCTCTAGATCTTTGAGGAATCACCACACTGTCTTCtA

caatggttgaactaatttacagtccac---cagtgtaaaagcattcctttatc
CAATGGTTGAACTAATTTACAGTCCAC---CAGTGTAAAAGCATTCCTTTATt
CAATGGTTGAACTAATTTAtactcccaccaCAGTGTAAAAGCATTCCTTTATC

tctgcaacctcactggcatctgttgtttctggacttttaataatcgcca
TCTGCAACCTCgCcGGCATCTGTTGTTTCTGGACTTTTAATAATtGCCA
TCTGCAAtCTtgCcaGCATCTGTTGTTTCTGtACTTTTaAATAATCGCCA

ttctgacgtgaggtggtatctcattgtggttttgatgtgcgtttctctaa
TTCTGACaTGAGGTGGTATCTCATTGTGGTTTTGATGTGCGTTTCTCTAA
TTCTaactggc------------------TTTTGATtTGCaTTTCTCTAA

tgatgagtgatgctgagctttttttcatgtttgttggctgcatgaatgtc
TGATGAGTGATGCTGAGCTTTTTTTCATGTTTGTTGGCTGCATGAATGTC
TGATGAGTGATGCTGAGCTTTTTTTCATGTTTGTTGGCTGCATGAATGTC

ttcttttgagaagtgtctgttcatgtcctttgcccacttttttaatggggt
TTCTTTTGAGAAGTGTCTGTTCATGTCCTTTGCCCACTTTTTAATGGGGT
TTCTTTTGAGAAGTGTCTGTTCATGTCCTTTGCCCACTTTTTAATGGGGT

tggtttttcttgtaaatttgtttaagttccttgtagactctggatatta
TGGTTTTTCTTGTAAATTTGTTTAAGTTCCTTGTAGACTCTGGATATTA
TGGTTTTTCTTGTAAATTTGTTTAAGTTCCTTGTAGACTCTGGATATTA

gacctttgtcagatgctccgattgcaaaaattttctcccgttctgtaggt
GACCTTTGTCAGATGCTCCGATTGCAAAAATTTTCTCCCaTTCTGTAGGT
GACCTTTGTCAGAgGCTCaGATTGCAAAAATTTTCTCCCacTCTaTAGGT

tgtctgttcactctgatgatagtttcttttgctgtgcagaagctctttag
TGTCTGTTCACTCTGATGATAGTTTCTTTTGCTGTGCAGAAGCTCTTTAG
TGTCTaTTaACTCTGATGATAGTTTCTTTTGCTGTGCAGAAGtTCTTTAG

tttaattagatctcatttgtcaattttggcttttgctgcaattgcttttg
TTTAATTAGATCTCgTTTGTCAATTTTGGCTTTTGtTGCgATTGCTTTTG
TTTAATTAGATCcCATTTGTCAATTTTGGCTTTTGtTGCAATTGCTTTTG

gcgttttgtcgtgaaatctttgcccatgcctatgttctgaatggtattg
GCaTTTTTGTCGTGAAATCTTTGCCCATGCCTATGTTCTGAATGGTATTG
GCGTTTTcGTCaTGAAATCTTTGCCCATGCCTgTGTTCTGAATGtTATTG

cctagattttttctggggtttttatagttttgggttttacatttaagtc
CCTAGATTTTTTTCTGGGGTTTTTATAGTTTTGGGTTTTACATTTAAGTC
CCTAGATTTTTTTCTGGGGTTTTTATAGTTTTGGaTTTTACATTTAAGTC

tttaatccatcttgagttaatttttgtgtaagatgtaaggaagggatata
TTTAATCCATCTTGAGTTAATTTTTGTGTAAGATGTAAGGAAGGGATATA
TTTAATCCATCTTGAGTTAATTTTTGTGTAAGATGTAAaGAAGGGgTATA

gtttcaattttctgcatatgcctacccagttctcccagcaccatttatta
GTTTCAATTTTCTGCgTATGCCTACCCAGTTCTCCCAGCACCATTTATTA
GTTTCAATTTTCTGCATATGgCTAgCCAGTTCTCCCAGCACCATTTATTA

aatagggaatcctggccgggcgcagtggctcacacccgtaatcccagcac
AATAGGGAATCCTGGCCGGGCGCAGTGGCTCACACCCGTAATCCCAGCAC
AATAGGGAATCCTGtCCGGGCGCAGTGcCTCACgcctaTAATCCCAGCAC

tttgggaggctgaggcgggtggatcacctgaggtcagga
TTTGGGAGGCTGAGGCGGGTGGATCACCTGAGGTCAGGA
TTTGGGtggccgaggtGGGTGGATCACCTGAGGTtggga
```

chr5:162752527-1627528300
Alignment
Human, chimp, rhesus, consensus, donor
ggtttctttttcagctaattcattaccagtgtgcagacatgctaccgatc
GGTTTCTTTTTCAGCTAATTCATTgtCAGTGTGCAGACATGCTACCGATC
GGTTTCTTTTTCAGCTAATTCATTAtCgaTGcatAGACATGgTACtGATC

atttctatataccaataacattgaaggtgaaagccaaatcaagaacacaa
ATTTCTATATACCAATAACATTGAAGGTGAAAGCCAAATCAAGAACACAA
ATTTCTATATACCAATAACATTGcAGGTGAAAGCCAAATCAAGAACACtA

tcccatttacaatagccacagacacacatgaaaaaagtacctagaaaata
TCCCATTTACAATAGCCACAGACACACATGAAAAAAGTACCTAGAAAATA
TCCCATTTACAATAGCCACAGACACACATGAAAAAAGTtCCTAGgAAAgA

catctaatcaaagaaatgaaagatctctacaaggagaactacaaagcact
CATCTAATCAAAGAAATGAAAGATCTCTACAAGGAGAACTACAAAGCACT
CATCTAAcCAAAGAgATGAAAGATCTCTACAAGGAGAgtTACAAAaCACT

gctaaaagaaatcagacatgacccaatcaaatcgaaaaatattccaggct
GCTAAAAtAAATCAGAtATGACCCAATCAAATCGAAAAATATTCCAGGCT
GCTAAAAGAAATCAGACATGACaCAATCAAATgGAAAcATATTCCAtGCT

catggattagaagaattaatattgtaaaaatggccatcctgcccaaagaa
CATGGATTAGAAGAATTgATATTGTAAAAATGGCCATCCTGCCCAAAGcA
CATGGATTAGAAGAATTAATATTGTAAAAATGaCCATCCTGCCCAAAccA

atctatagattcagtgctattcctatcaaactgccaacatcatttttaac
ATCTATAGATTCAGTGCTATTCCTATCAAACTGCCAACATCATTTTTAAC
ATCTATAGATTCAGTGCTATTCCTATCAAACTGCCAACATCATcTTTTAC

agaattagaaaaaagtcttctagaaAAAAATTAAATATTTCACAATTGTG
AGAATTAGAAAAAAGTCTTCTAGAAAAAAATTAAATATTTCACAATTGTG
AGAATTAGAAAAAAGTCTTCT-----------------------------
AGAAAAAAATTAACTATTTCACAATTGTG

AAATTATAATTTAATGTAAGGTAAATTCAGGTAGAACCCATCATCTCCCA
AAATTATAATTTAATGTAAGGTAAATTCAGaTgGAACCCAcCATCTCCCA

AAATTATAATTTAATATTAGGTAAATTCAGGTGGAACCCATCATCTCCCA

ACCCACTTCCCTACAACACTCTATGCCAAGAAGAGATCTTCCTAAGATAT
ACCCACTTCCCTACAACACgCcATGCCAAGAAGAGATCTTCCTAAGATAT

ACCCCGTTCCCTACAACACTCCATGCCAAGAAGAGACCTTCCCAAGATAT

TTTCCTTTTCCCAAGGAGAGGTTGGGGTGGAACCCCCGTGTGAAGGATTT
TTTCCTTTTCCCAAGGAGAGGTTGGGGTGGAcCCCCtGTGTGAAGGATTT

TTTCCTTTTCCCAAGGAGAGTTTGGGGTAGACCACCCGTGTGAAGGATTT

TAAAGAGGAAGTTTCAGGAAAGAGATGACATAGGGGAGATCAAGGCAGAG
TAAAGAGGAAGTTTCAGGAAAGAGATGACAcAGGGGAGATCAAGGCAGAG

TAAAGAGGAAGTTTCAGGAAAGAGACGACATAGGGGAGATCAAGGCAGAG

AATGAGAGAGCTGCCATGAGGGACTAGAAATAGGGACACCTTTGTTTGGT
AATGAGAGAGCTGCCATGAGGGACTAGAAATAGGGACACCTTTGTTTGGT

AATGAGAGAGCTGCCATGAGGGACTAGAAATAGGGGCACCTTTGTTTGGT

GTGGGGGAACAGGTTAGGAAAA**AAAAAAAA**agaaaaaagtcttgtaaaat
GTGGGGGAACAGGTTAGGAAAAAAAAAAAA-GAAAAA-GTCTTcTAAAAT

---AAAAT
GTAGGGGAACAGGTTAGGAAAA

tcatatggaaccaaaaatgggcctgaatagccaaagcaatcctaaacaaa
TCATATGGAtCCAAAAATGGGCCTGAATAGCCAAAGCAATCCTAAACAAA
TCATATGGAACCAAAAATGGGCCTGAgTAGCCAAAGCAATCaTAAACAAA

acacacacacacacaaacacacaca
ACACACACACACACAcACACACACA
ACACACACACACACAcACACACAaA

chr18:31908135-31908312
Alignment
Human, chimp, rhesus, consensus, donor
TGCAGGTTGACTTGAGCTATGTCAAAAGGGAGTACTGAGAAAGTAAGAAG
TGCAGGTTGACTTGAGCTAcGTCAAAAGGGAGTACTGAGAAAGTAAGAAG
TGCAGGTTGACTTcAaCTAcaTCAAAAtGGgGTACTGAGAAAGTAgGAAG

ATAACTctggttactgacagctaaactgcaatcttgccaatgaaacataa
ATAACTCTGGTTACTGACAGCTAgACTGCAATCTTGCCAATGAAACATAA
ATAACTgTGGTTACTGACAaCTAgACTGCAgTCTTGCCAgTGAAACATAA

actgttctcacaggatataaacaataattaggcaagtccattctatgact
ACTGTTCTCACAGGATATAAACAATAATTAGGCAAGTCCATTCTATGACT
ACTGTTCTCACAGGATATAAACAATAATTAGGCAAGTCCATTCTATGACT

gttgtggagcaagacagagccaaggccaccctgcagccatgaaaattgct
GTTGTGGAGCAAGACAGAGCCAAGGCCACCCTGCAGCCATGAAAATTGCT
GTaGTaGAGCAAaACAGAGCCAAGGCtACCtTGCAGCCATGAAAATTGCT

agtatctccttctgataactaacatgaataattacagcttctttgccagt
AGTATCTCCTTCTGATAACTAACATGAATAATTACAGCTTCTTTGCCAGT
gGTATtgCCTTCTGATAACTAACATGAATAATTACAcCTTCTTaGCCAGT

gacatctctatccccatcttaaccctctgacctcctacataaaaa-tgatc
GACATCTCTATCCCCATCTTAACCCTCTGACCTCCTACATAAAAA-TGATC
GACATCTCTAgCCCCATCTcAACCCTCTGACCTCCTACATAAAAAaTGATC

aaagatctacccaatcaccaaattgctcctgctcctcacagcacccaatc
AAAGATCTACCCAATCACCAAATTGCTCCTGCTCCTCACAGCACCCAATC
AAAaATCTACCCAAcCACCtAgTTGCTCCTGCTCCTCACAGCACCtAATC

cagaactgactctgcacctctaaagtctctttagaatcacgcaacacaag
CAGAACTGACTCTGCACCTCTAAAGTCTCTTTAGAATCACGCAACACAAG
CAGAACTGACTtTGCACCTCTAAAGTaTCcTTAGAATCACG-----CAAG
TAAATACAATAAGATTTTAA

cttaaatctt**TTT**TTTTGATCAGAATAAATTTATTGGACATTTTTTAAAA-
CTTAAATCTTTTTTTTTTttATCAGAATAAATTTATTGGACATTTTTaAAA-
CTcAAATCTT---
TTTTGATCAGAATAAATTTATCAGACATTTAAAAAAAG

-AATTTCAAAACCTAAAATAAAATAATATCCAAGTATAAATGCAATTTTCC
AAATTTCAAAACCTAAAATAAAATAATATCCAAGTATAAATGCAATTTTCC

AAATTTCAAAACCTAAAATATAATAATATCCAAGTATAAATGCAATTTTCC

TTTTAAAATATATACGATGCAACAATTACATTTATGAACTAATTCTTAAT
TTTTAAAgTgTATACGATGCAACAATTACATTTATGAACTAATTCTTAAT

TTTTAAAGTATATACGATACAACAATTACATTTATGAACTAATTCTTAAT

ACATAGGTATAAAAATGTGAATTTATTTGAAGTG**ACCTC**caagcttaaat
ACATAGGTATAAAAATGTGAATTTATTTGAAGTGACCTCCAAGCTTAAAT

ATGTAGATATAAAAATATGAATTTATTTGAAGTG

cttaaaataagcccttcccaattccttcttaataacacaccccatagttg
CTTAAAATAAGCCCTTCCCAATTCCTTCTTAATAACACACCCCATAGTTt
---AAAATAAGCC-TTCCCAATTCCTTC-TAATAACAtACCCCATAGTTt

ctgtggtgtgttctcattcttgtttgctgcagcaaactaaataaactttg
CTGTGGTGTGTTCTCATTCTTGTTTGCTGCAGCAAACTAAATAAACTTTG
CTGTGGTGTGTTCTCATTCTTGTTTGCTGCAGCAAACTAAATAAACTTTG

tttaacattatatggttcttatggtctttggttggtgggTTTGGTTTTTG
TTTAACgTTATATGGTTCTTATGGTCTTTGGTTGGTGGGTTTGGTTTTTG
TTTAAagTTATATGGTTCTTATGGTCTTTGGcTGGTGGGTTTGGTTTTTG

TTTGTTTGGTTGGTAtttttaagatagggtcttgctatgtcaccctggc
TTTGTTTGGTTGGTATTTTTAAGATAGGGTCTTGCTATGTCACCCTGGC
TTTGTTTGtTTGGTATTTTTAAGATAGGGTCTTGCT

chr8:8994997-8995187
Alignment
Human, chimp, rhesus, consensus, donor

```
CGATGAAGACCAAGATGGTAAAGATGAAAAATTGTagctaacatttactg
CGATGAAGACCAAGATGGTAAAGAaGAAAAATTGTAGCTAgCATTTACTG
CaATGAAGACCAAGATGGTAAAGAaGAAAAATTGTAGCTAACATTTACTG

cacatttactacaagccaagcattgcactatgaagtttaagtgcattatt
CACATTTACTACAAGCCAAGCATTGCAtTATGAAGTTTAAGTGCATTATT
CACATTTACTACAAGtCAAGCATTGCgCTATGAAcTTTAAGTGCgTTtTT

cattaaccccttcaataaAATTTGTAATTTTCATTTCAGAAGCAAATATT
CATTAACCCCTTCAATAAAATTTGTAATTTTCATTTCAGAAGCAAATATT
CATTAACCCCTTCAATAA--------------------------------
                  AATTTGTAATTTTCGTTTCAGAAGCAAATATT

CGTGTTGTACAAATTTCTGATTGCCCTAAATGTAGAGAGACTGATGGGGA
CGTGTTGTACAAATTTCTGATTGCCCTAAATGTAGAGAGACTGATGGGGA
--------------------------------------------------
CGTGTTGTACAAATTTCTGATTGCCCTAAATGTAGAGAGACTGATGGGGA

AAGTATGATGGGTTTGATTTTTATATCAAATCATCAGGCATGGAGAAATA
AAGTATGATGGGTTTGATTTTTATATCAAATCATCAGGCATGGAGAAATA
--------------------------------------------------
AAGTATGATGGGTTTGATTTTTATATCAAATCATCAGGCATGGAGAAATA

TCTTTTAGAAGTGTTAAAATAAATGTTCCTACTGTATATTTAAAATACAA
TCTTTTAGAAGTGTTAAAATAAATGTTCCTACTGTATATcTAAAATACAA
--------------------------------------------------
TCTTTTAGAAGTGTTAAAATAAATGTTCCTACTGTATATTTAAAATACCA

AAAATGATAAtaataataataacaattcctctgaaatagttaccgtgaat
AAAATGATAATAATAATAATAAttATTCCTCTGAAATAGgTACCGTGAAT
-------------------------TTCCTCTGAAATAGgTACCGTGAAT

accctcatttaaaatgaggaagctaagccttttcaaagttaagtaacttg
AaCCTCATTTAAAATGAGGAAGCTAAGCCTTTTCAAAGTTAAGTAACTTG
AtCCTCATTTAAAATGAGGAAGCTAAGgCTTTTCAAAGTTAAGTAACTTG

cctaaggtctcttaacaactagcgaaaaggatagaactcaaacctaggtc
CCTAAGGTCTCTTAACAACTAGCGAAAAGGATAGAACTCAAACCTAGGTC
CCTAAGGTCTCTTAACAACTAGCGAAAAGGATAGAACTCAAACCT--GTC

tttctgaccctgcagtttgtgtttctatccacGGTGTAAGTGAATGAGTC
TTTCTGACCCTGCAGTTTGTGTTTCTATCCAtGGTGTAAGTGAATGAGTC
TTTCTGACCtTaCAGcTTGTGTTTCTATCCAtGGTaTAAaTGAATGAGTC

AATGTATGCTAAAGTCAAGACTCTTATC
AATGTATGCTAAAGTCAAGACTCTTATC
AATGTATGCTAAAGTCAAGACgCTTATC
```

chr8:138184846-138184991
Alignment
Human, chimp, rhesus, consensus, donor

```
agctaactttaacctatcgcaAGGAGCAAACCCACAGTGAAAAGCtgcat
AGCTAACTTTAACCTATCaCAAGGAGCAAACCCACAGTGAAAAGCTGCAT
AGCTAtCTTTAACCTATtGCAAGGAGCAAACCCACAGTGAAAAGCaGCAT

tactctacttggtctgccataagaaaataccatagactgggtggctgaaa
TACTCTACTTGGgCTGCCATAAGAAAATACCATAGACTGGGTGGCTGAAA
TACcCTACTTGGgCTGCCATAAGAAAATACCATAGgCTGGGTGGCTaAAA

caacaggaatttattttcttacagttctggaggctggaagtcgagagatc
CAACAGGAATTTATTTTCTTACAGTTCTGGAGGCTGGAAGTCGAGAGATC
CAACAGGAATTTATTTTCTTACAGTTCTGGAGGCTGGAAGTCGAGAGATC

agggtgtcagcagggtgctgttctgacgaagacgctcttactggcttaca---
AGGGTGTCAGCAGGGTGCTGTTCTGAtGAAGACGCTCTTACTGGCTTACA---
AGGGTGTCAGCAGGGTcCTGTTCTGgtGAAGgCaCTCTTtCTGGCcTACA---

ggcttctgactgtgtcctcatgtggccttcctctgtgaaagctcactctt
GGCTTCTGACTGTGTCCTCATGTGGtctt--------tAAGCTCACTCTT
GGCTTCTGACTGTGTCCTCAcGTGGCCTTCCTtTGTGAAAGCTCAtTCTT

tcttcttgtaaggataccaatcCTGTCGATATCCTTAGGAGTCCTCAAAT
TCTTCTTGTAAGGATACCAATCCTGTCGATATCCTTAGGAGTCCTCAAAT
TCTTa---TAAGGcTACCAATCctatca----------------------
                                 CTTAGGAGTCCTCAAAT

GCATCATGCTCTTCCACACATATTTCTCCCTTCTCTGCTTTTTGTGTGTG
GCATCATGCTCTTCCACACATATTTCTCCCTTCTCTGCTTTTTcTGTGTG
--------------------------------------------------
GCATCATGCTCTTCCACACATATTTCTCCCTTCTCTGCTTTTTCTGTGTG

CCTTTCTTTTAGCCTGGTTAACTTAATCTAAATTAAGTCTCCTTAATTCT
CCTTTCTTTTAGCCTGGTTgACTTAATCTAAATTAAGTCTCCTTAATTCT
--------------------------------------------------
CCTTTCTTTTAGCCTGGTTAACTTAATCTAAATTAAGTCTCCTTAATTCT

CTAAACTTCTCAGGTCCTAAgtcctgtcggattaggaccacatccttatg
CTAAACTTCTCAGGTCCTAAGTCCTGTCGGATTAGGACCAtATCCTTATG
-----------------------------GATTAGGACCACATCCTTATG
CTAAACTTC

atctcatttaaccatcttaaaagccctttctccaaagatggtcacattgg
ATCTCATTTAACCATCTTAAAAGCCCTTTCTCCAAAGATGGTCACATTGG
ATCTaATTTAACCATCTTAAAAGCCCTTTCTCCAAAaATGGTCACATTGG

gggtttgggcttcaacatctgaattttcagtgggcacagtgcagtcaata
GGGTTTGGGCTTCAACATCTGAATcTTCAGTGGGCACAGTGCAGTCAATA
GGGTTTGGGCTTCAACATCTGAATTTTCAGTGGGCACAaTGCAaTCAAcg

gcaACATCAGAGAAATGAACAAGATACACataacatccctt
GCAACATCAGAGAAATGAACAAGATACACATAACATCCCTT
GCAACATCAGAGAAATGAACAAGATACACgTAACATttctt
```

chr5:23057186-23057475
Alignment
Human, chimp, rhesus, consensus, donor
ttggggatggtgcaaatatattttgcctgtggaatggacataaactt-------------GGT
TTGGGGATGGTGCAAATATATTTTGCaTGTGGAATGGACATAAACTT-------------GGT
           GCAAATATgTTTTGCaTGTGGAATGGAtATAAACTTgggggaactagatGGT

AGACTCTTATGGATTGAATAATGTTCCCCCAAATTCATCTATCCAAAA-TG
AGACTCTTAgGGATTGAATAATGTTCCCCCAAATTCATCTATCCAAAA-TG
AGACTCgTATGGgTTGAATAATGTTCCCtCAAATTCATCTATCCAAAAaTt

TGaaaatgcgacttcatttagaaatatggtatttcccgatgtaattagta
TGAAAATGCGACTTCATTTAGAAA---TGTATTTCCCGATGTAATTAGTA
TGAAAATGtGACTTCATTTAGAAA-cATGaTATTTCCtGATGTAATAGTA

ttagggtagaggacacagagagggacacagagaggaatggcatattaaga
TTAGGGTAGAGGACACAGAGAGGGACACAGAGAaGAATGGCATATgAAGA
TTAGGGTAGAGaACACAGAGAaGGACACAGAGAGGAATGGCATATgAAGA

aagaagtggaattatgttgccacaagccaagaaatatcaggagctaccag
AAGAAGTGGAATTATGTTGCCACAAGCCAAGAAATATCAGGAGCTACCAG
AAGAgGTGGAgTTATGTaGCCACAAGCCAAGAAATATCAGGAaCTACCAG

aagctagaagaggcaaggaaggattttcccttctagtcttgggaagggag
AAGCTAGAAGAGGCAAGGAAGGATTTTCCCTTCTAGTCTTGGGAAGGGAG
AAGCTAGAAGAGGCcAaGAAGGATTTTCCCTTCaAGTCTTGGGAAGGGAG

tatggccctgctgccactttgattttggacttctggcttttagaattgtg
TATGGCCCTGCcGtCACTTTGATTTTGGACTTCTGGCTTTTAGAATTGTG
gATGGCCCTGCTGCCACTTaGATTTTaGACaTCTGGCTTTcAGAATTGTG

aaagaatacatttctttctttT**TTTTTTTTTTTT**--GCAATCTTGTTTTTG
AAAGAATAaATTTcCTTTCTTTTTTTTTTTTTTTTttGCAATtTTGTTTTTG
AAAGAATAaATTTcCTTTCTTtt-----------------------------
                                     GCAATTTTGTTTTTG

AAATTAATTTTTATTTTCTTCACATTGTGTTGGATGATCAGAGGATTATA
AAATTAATTTTTATTaTCTTCACATTGTGTTGGATGATCAGAGGATTATA

AAATTAATTTTTATTATCTTCACATTGTGTTGGATGATCAGAGGATTATA

ATTATTCCCTGCCCCAGGGGAGAGTATTGCAAAGGTACATAGGGCATGAA
ATTATTCCCTGCCCCAGGGGAGAaTATTGCAAAGGTACATAGGGCATGAA

ATTATTCCCTGCCCCAGGGGAGAGTATTGCAAAGGTACATAGGGCATGAA

CGTGGGGAGGGCAAGAAGCGTGCCCAAAGCGTGCTATAGCACAGTGTGTG
CGTGGGGAGGGCAAGAAGCGTGCCCAAAGCGTGCTATAGCACAGTGTGTG

CGTGGGGAGGGCAAGAAGTGTGCCCAAAGCGTGCTATAGCACAGTGTGTG

TTCCACTCCTCATACAGCAATGAACTGGTGAAGGAGTTAAAATCTTAATG
TTCCACTCCTCATACAGCAATGAACTGGTGAAGGAGTTAAAATCTTAATG

TTCCACTCCTCATACAGCAATGAACTGGTGAAGGAGTTAAAATCTTAATG

ATTTCTAAATGATCAACAGTTTTAAAAAACATAAATATGCAGCATGAGAG
ATTTCTgAATGATCAACAGTTTTtAAAAACATAAATATGCAGCATGAGAG

ATTTCCAAATGATCAACAGTTTTTAAAAACATAAATATGCAGCATGAGAG

CCTACATTTGTA----------Ttttctggtactttgctacagcagctctaggaaactga
CCTACATTTGTA----------TTTTCTGGTACTTTGCTACAGCAGCTCTAGGAAACTGA

------------<u>aagaaaccaa</u>TTTTCTGGTACTTTGCTACAGCAtCTCTAGGAAACTGA
CCTACATTTGTA

tGTAACATATGATAACCAACACAACATGTAAATT
TGTAACATATGATAAtCAACACAACATGTAAATT
TGTAAtATATGATAACtAAtACAACATGTAAATT

chr6:148274962-148275037
Alignment
Human, chimp, rhesus, consensus, donor
AGATCTGCACAGAGTTACCTTCAAGACAGTGCCAAGCCTTTGCtgtacca
AGATCTGCACAGAGTTACCTTCAAGACAGTGCCAtGCCTTTGCTGTACCA
AGATCTGgACAGAGTTACCTTCAcGACAaTGCCAtGCCTTTGCTGTACCA

gttatctattgctgttttagaaactacccaaaaacttagtgtcctaatat
GTTATCTATTGCTGTTTTAGAAACTACCCAAAAACTTAGTGTCCTAATAT
GTTATCTATTGCTGTTTaAGAAACTACCCAAAAACTTAGTGTCtTAATAT

tatgaccatgtgttattatttatgaatctgtgggtcagctgagtagctgg
TATGACCATGTGTTATTATTTATGAATCTGTGGGTCAGCTGAGTAGCTGG
TAcGACCATGTGTTATTATTTtTGAATCTGTGGGTCAaCTGAGTAGCTGG

tctgggccagatgcagctgatcccagacagactagccatgcatcagctct
TCTGGGCCAGATGCAGCTGATCCCAGACAGACTAGCCATGCATCAGCTCT
TCTGGGCCAGATGCAGCTGATCgCAGACAGACTAGCCATGCAcCAaCTCT

cagctgttgggttggttggggcctgccagatctagaagggcctcactcac
CAGCTGTTGGGTTGGTTGGGGCCTGCCAGATCTAGAAGGGCCTCACTCAC
CAGCTGcTaGGTcaacTGGGGCCTGgCAGATCTAGAAGGGCCTCACTCAC

ctgtctggtgattgcctggctaagagtgatggctgtaactagaccatatg
CTGTCTGGTGATTGCCTGGCTAAGAGTGATGGCTGTAACTAGACCATATG
CTGTCcGGTGATTGCCTGGCTAAGAGTGATGGCTGTAACTAGACCATATG

tctctagccattcatcatgctacgacagatttttctttcacaaaacaagg
TCTCTAGCCATTCATtATGCTACGACAGATTTTTCTTTCACAAAACAAGG
TCTCTAGCCATTCATCATGCTAgGACAGcTTTTTCTTTCACAAAACAAGG

acagactctcaaaagagtgtgtgcaagcatgcaaggcccatgaggcctag
ACAGACTCTCAAAAGAGTGTGTGCAAGCATGCAAGGCCCATGAGGCCTAG
ACAGAaTCTCAAAAGAGTGTGTGgAAGCATGCAAGGCCCATGAGGCCcAG

actcagacctgatgcaccgttacttctcatagattctgttggtcaaagcc
ACTCAGACCTGATGCACCGTTACTTCTCATAGATTCTGTTGGTCAAAGCC
ACTCgaACCTGATGCACCaTTACTTCTCcTAGATTCTGTTGGTCAAAGCC

agtccaagacaaagggtgggggaaaagattctatttctt**TTTTTTTTTA**T
AGTCCAAtACAAAGGGTGGGGGAAAAGATTCTAcTTCTTTTTTTTTT-AT
AGTtCAAtACAAAGGGTGGGGtaaatattcc-ACTTCTT-----------
T

GCTGAATGTTTATAATTAAGTAAGCTTACTCTAAGAAGCACAAATTTAGA
GCTGAATGTTTATAATTAAGTAAGCTTACTCTAAGAAGCACAAATTTAGA

GCTGAATGTTTATAATTAAGTAAGCTTACTCTAAGAAGCATGAATTTAGA

ACACAAGAAACTAAAAacttcttagtgggaaaaactgcaaagtgtgatta
ACACAAGAAACTAAAAACTTCTTAGTGGGAAAAACTGCAAAGTGTGAgTA
-----------------------AGTGaGAAAAACTGtAAAGTGTGAgTA
ACACAAGAAACTAAAA

gaggaaggagtggagaattggagctatttcttgcaatcagtttaTGTAGA
GAGGAAGGAGTGGAGAATTGGAGCTATTTCTTGCAATCAGTTTATGTAGA
GAGGAAGGAGTGGAGAATTGGAGCTATTTCTTGCAgTCAGTTTATGTAGA

TGTGA
TGTGA
Tacga

chr4:140781323-140781711
Alignment
Human, chimp, rhesus, consensus, donor

```
cctctgaaagtagtcacatggttatacctggtatttatttatttttatt
CCTCTGAAAGTAGTCACATGGTTATACCTGGTATTTATTTATTT---ATT
 CTCTGAAAGcgGTCACATGGTTATACCTGGTATTTATTTATT----ATT

attattattattatttgagacagggtctcaccctgtcacacaggctggag
ATTATTAaTATTATTTGAGACAGGGTCTCACCCTGTCgCACAGGCTGcca
ATTgTTATTATTATT-GAGACAGGGTCTCACCtTGTtgCACAGGCTGGAG

tgcagtggtgcaattatggctcactgctacctctgccacctgggctcaag
TGCAGTGGTGCAATTATGGCTCACTGCTACCTCTGCCACCTGGGCTCAAG
TGCAGTGGTGCAATcATGGCTCACTGCaACgTCTGCCACCTGGGCTCAAG

ccatcctctcaccttagccttctgaacagctgggatacatgtgcacTAAT
CCATCCTCT--CCTcAGCCTcCTGAACAGCTGGGATACATGTGCACTAAT
CCATCCTCTCACCTcAGCCTcCTGAAtAGCTGGGATACgTGTGCAttatT

TTTTTTTTTTTTGAAACAAAAATATTTGTGTAGAAGGCACAAAAGCTACA
TTTTTTTTTTTTGAAACAAAAATATTTGTGTAGAAGtCgCAAAAGCTACA
TTTT----------------------------------------------
           TGAAACAAAAATATTTGTGTAGAAGGCATAAAAGTTATC

ATCACAGACTCCACTGTGCAAAGGCGCAACCTGCCTCATTGATCTCTAGT
ATCACAGACcCCACTGTGCAAAGGtGCAtttTGCCcCATTtATCTCTAGT
--------------------------------------------------
ATCACAGACTCCACTGTGTAAAGGCATAACTTGCTTTATTTATCTCTAGT

GTACACCAACCCAGCTTCCCTTTCCATTCAGCCTGTGAAAGGAGATAGTG
GTACACCAACCCAGCcTCCCTTTCCATTCAaCCTGTGAAAGGAGATAGTG
--------------------------------------------------
GTATATGAACTTAGCCTCCCTTTCCATTCAGCCTGTGAAAGGAGATAGTG

CTTGGGCCATTTGGTAAAAGAAGGGGATGGGAGATGATCAAAACCCCAAG
CTTGGGCCATTTGGTA---GAAGGGGATGGGAGATGATCAAAACCCCAAG
--------------------------------------------------
CTTGGGCCATTTGGTAGAAGAAGGGGATGGGAGATGATCAAAACCCCAAG

TAAGGTTCATCCA--ATATGGTGTCTAAGCAGCAAATGACTAATTGCTGAAG
TAAGGTTCATatcc-ATATGGTGTCTAAGCAGCAAATGACTAATgGCTGAAG
----------------------------------------------------
TAAGGTTCATATCCAATATAGTGTCTAAGCAGCAAATGACTAATGGCCGAAG

AAGGAGACTAGACAGAGGATTAGAGGCAGCCATGGGGCCAGTGCAGCTGT
AAGGAGACTAGACAGAGGATTAGAGGCAGCCATGGGGCCgGTGCAGCTGT
--------------------------------------------------
AAGGAGACTAGACAGAGGATTAGAGGCAGCCATGGGGCTGGTGCAGCTGT

GGAGAGCTCTGAGCAAAGAAACAAGGTTGGCAGGTGAGGAGGCCTAGGAT
GGAGAGCTCTGAGCAAAGAAACAAGGTTGGCAGGTGAGGAGGCCTAGGAT
--------------------------------------------------
GGAGAGCTCTGAGCAAAGAAACAAGGTTGGCAGGTGAGGAGGCCTAGGAT

AGAGGCCAGAAGGCCAAGCCTGGGGCTGCGTGTGCActaattttgtatt
AGAGGCCAGAAGGCCAAGCCTGGaGCTGCaTGTaCACTAATTTTTGTATT
---------------------------------------------GTATT
AGAGGCCAGAAGGCCAAACCTGGGGCTG

tttttgctttttgatagagatgagatttcatcatgttgcacaggtgggt
TTTTTGtTTTTTTGgTAGAGATGAGATTTCATCcTGTTGCACAGGTGGGT
TTTTTGtTTTTTTGgTAGAcATGAGATTTCATCATGTTGCACAGactGGT
```

cttgaagtcctgggctcaagtggtctgcctgtctcggcctctcaaagtgc
CTcGAAcTCCcGGGCTCAAGTGaTCTGCCTGTtTCaGCCTCTCAAAGTGC
CTcGAAcTCCTGGGCTCAAGTGaTCTGCCTGTCcCGGCCTCTCAAAGTGC

tgggattataggtgtgagccactgcacccggctcatagctggtatttata
TGGGATTATAGaTGTaAGCCACTGCACCCaGCTCATAGCTGGTATTTATA
TGGGATTATAGGcaTaAGCCACTGCACCCGGCTCATAGCTGGTATTTATA

actacattctcccattatttattccatagttc
ACTACATTCTCCCATTATTTATTCCATAGTTC
ACTACATTCTCCCATTATTTATTCCATAGTTC

```
chr8:62616228-62616312
Alignment
Human, chimp, rhesus, consensus, donor
AATTGAAAAAAAAATTAAGAAAGGGAAATGAGAggccgggcacggtggct
AATTGAAAAAAAAATTAAGAAAGGGAAATGAGAGGCCaGGCgCGGTGGCT
     AAAAAAAAATTAAGAAAGGGAAATGtGAGGCtGGGCgCaGTGcCT

cacacctgtaatcccagcactttggaaggccgaggtgggcggatcacgag
CACACCTGTAATCCCAGCAaTTTGGAAGGCCGAGGTGGGCGGATCACGAG
CACACCTGTAATCCCAGCACTTTGGgAGGCCGAGGcGGGCaGATtAtGAG

gtcaggagatcgagaccatcctggttaacaaggggaaaccccgtctctac
GTCAGGAGATCGAGACCATCCTGGTTAACAAGGtGAAACCCCGTCTCTAC
GTCAGGAGATCGAGACCATCCTGGTTAACAAGGtGAAACtCtGTCTCTAC

taaaaaatacaaaaaa--ttagAGTTAAGGCAATATAATGGTTTTGTATTTT
TAAAAAATACAAAAAA--TTAGAGTTAAGGCAATATAATGGTTTTGTATTTT
TgAAAA-TAcAAAAAAAATTAG------------------------------
                        TTAAGGCATTATAATGATTTTGTATTTT

--AAAAGATAGAATTTATTTATTCAACATGACTTTTATAAGAGTAAAGATAT
--AAAAGATgGAATTTATTTATTCAACATGACTTTTATAAGAGTAAAGATAT
----------------------------------------------------
TTAAAAGATGGAATTTATTTATTCAACATGACTTTTACAAGAGTAAAGATAG

AATTCAtattaaaaaaaaaattagccgggcatggtggtgggcacctgtag
AATTCATcTTAAAAAAAAA-TTAGCCGGGCATGGTGGTGGGCACCTGTAG
------------------------CCaGGggTGGTGGTGGGCgCCTGTAG
AATTCATCTAAA

tcccagctacttgggaggctaaggcaggagaatagcgtgaacccgcgagg
TCCtAGCTACTTGGGAGGCTAAGGCAGGAGAATAGCGTGAACCCGCGAGa
TCCCAGCTACTcGGGAGGCTgAGGCAGaAGAATgGCGTGAACCtGCGAGG

cggagcttgcagcgagctgagattgcgccactgcactccagcctgggcaa
CGGAGCTTGCAGtGAGCTGAGATTGCaCCACTGCACTCtAGCCTGGGCAA
CaGAGCTTGCAGtGAGCcGAGATcataCCAgTGCACTCCAGCCTGGGtAA

cagagtgagactccgtctaaa-----------------aaaaaaGGGAAATGCCCTATTCATGGTAG
CAGAGTGAGACTCtGTCTAAA-----------------AAAAAAGGGAAATGCCCTATTCATGGTAG
CAGAGcaAGACTCtGTCTAAAaaaaaaaaaaagaaaagAAAAAAGGGAAATGCCCTATTCATGaTAG

TTAAAAGTCACTCAAGGGCCA
TTAAcAGTCACTCAAGGGCCA
TTAAGTCACTCAAGGGCCAgA
```

```
chr12:97618896-97619143
Alignment
Human, chimp, rhesus, L1MB4 consensus, donor
AGTTTGTACCCCTTTAGCAGGAAACTAAGAGGAAAAATACCTCCCTTTAA
AGTTTGTACCCCTTTAGCAGGAAACTAAGAGGAAAAATACCTCCCTTTAA
AGTTTGTACCCCTTTAGCAGGAAACTAAGAGGAAAAATAtCTCCCTTTAA

GTTTGTTAAAATCTATAAGAACAGAAAAGAAACCACTGCATAAGCAAGAC
GTTTGTTAAAATCTATAAGAACAGAAAAGAAACCACTGCATAAGCAAGAC
GTT----AAAATCTATAAGAACAaAAAAGgAACCAtTGCATttGCAgaAC

TTGAAGATTAAAACttttt-tattgtgataaaatgcatataatgtaaaagt
TTGAAGATTAAAACTTTT--TATTGTGATAAAATGCATATAATGTAAAAGT
TTGAAGAgTAAAACtTTTTTTATTGTGATAAAATGCATATAATGgAAAAtT
               ttttttattgtggtaaaatatacataacataaaatt

tgccattttaaccatctaaaaatgtgcatttcaatggcatta----cattcaca
TGCCATTTTAACCATCTAAAAATGTGCATTTCAATGGCATTA----CATTCACA
TGCCATTTTAACCATCTgAAAATGTaCATTTCAgTGGCATTA----CATTCACA
taccattttaaccattttaagtgtacaattcagtggcattaagtacattcaca

atgctgtgtagccattactactatatatttccaaaactttttcatcaccc
ATGCTGTGTAaCCATTACTACTATATATTTCCAAAACTTTTTCATCACCC
ATGCTGTGTAaCCATTACcgCTtcATATTTCCAAAACTTTTTCATCACCC
tgttgtgcaaccatcaccactatcc-atttccagaactttttcatcatcc

taaacagaaactttgtaaccatgaagtaataactcctctttcccctctct
TAAACAGAAACTTTGTAACCATGAAGTAATAACTCCTCTTTCCCCTCTCT
TAAACAGAAACTTTGTAACCATGAAGTAATAACTCCTCTTTCCCCTtTCT
caaacagaaactctgtacccattaaacagtaactccccatttcccctcc

tgctagacctttgtaatctctaatgtaa-tctgcgaatttgcctaggtatt
TGCTAGACCTTTGTAATCTCTAATGTAA-TCTGCGAATTTGCCTAGGTATT
TGtTAGACCTTTGTAAcCTCTAATGTAA-TCTGtGAATTTGCCTAGGTATT
ccccagcccctggtaaccctattctactttctgtctctatgaatttgctat

----------tcatataaatggaatcatataatatcggtccttttgtgtctgactcattt
----------TCATgTAAATGGAATCATATAATATtGGTCCTTTTGTGcCTGACTCATTT
----------TCATATAAgTGGAATCATATAATATCaGTCCTTTTGTGTCTGACTCATTT
tctaggtacctcatataagtggaatcatacaatatttgtccttttgtg-ctggcttattt

cattgagcat---gttttcaaggttcattcatgttgtagcatgtatcagaact
CATTGAGCAT---GTTTTCAAGGTTCATTCATGTTGTAGCATGTATCAGAACT
CATTGAGCAT---GTTT-CAAGGTTCATTCATGTTGTAGCATGTATCAGAACa
cacttagcataatgttttcaaggttcatccatgttgtagcatgtatcagaatt

gtatttcttcttatggctgaataatattccattgtatgtatatatcacat
GTATTTCTTCTTATGGCTGAATAATATTCCATTGTATGTATATATCACAT
GTATTTCcTCTaATGGCTGAATAATAcTCCATTGTATGTATATATCACAT
tccttccttttaaggctgaataatattccattgtatgtatataccacaT

tttTTCTTTGTTTTTTTTTTTCTTACAAACCAGTGTTTATTTTCACATAC
TTTTTCTTTGTTTTTTTTTTTCTT-ACAAACCAGTGTTTATTTTCACATAC
TTT-----------------------------------------------
TTT-----------------------------------------------
                  TTTCTTACAAACCAGTGTTTATTTTCACATAC

AGCCTTGTTGTAACCAATGTACAGATCAAAACTCAAAATATTCCTCTTCA
AGCCTTGTTGTAACCAATGTACAGATCAAAACTCAAAATATTCCTCTTCA
--------------------------------------------------
--------------------------------------------------
AGCCTCGTTTTAACCAATGTACAGATCAAAACTCAAAATATTCCTCTGCA
```

```
GAAAACTTTGAACCTTATCCCCTAGCCGACCTATAATGGTTGAATTTCTG
GAAAACTTTGAACCTTATtCCCTAGCCGACCTATAATGGTTGAATTTCTG
--------------------------------------------------
--------------------------------------------------
GAAAACTTTGGACTTTATTCCCTAGCTGACCTATAATGGTTGAATTTCTG

TCAAGTATATATTTATGTATATAATGGGCTTAATTATTTAAGAGAATAAC
TCAAGTATATATTTATGTATATAATGGGCTTAATTATTTAAGAGAATAAC
--------------------------------------------------
--------------------------------------------------
TCAAGTATAAATTTATGTATATAATGGGCTTAATTATTTAAGAGAATAAC

TGAGTGATTTGTGTGATCTGTGAAAAGGAATTGGACTATCATACTTGGGT
TGAGTGATTTGTGTGATCTGTGAAAAGGAATTGGACTATCATACTTGGGT
--------------------------------------------------
--------------------------------------------------
TGAGGGATTTGTGTGATCTTTGAAAAGGAATTGGACTGTCATACTTGGGT

TGtatatcacattttttttcatccattcatctgttgatggacacttggttt
TGTATATCACgTTTTTTTCATCCATTCATCTGTTGATGGACACTTGGTTT
---------------ggTCATCCATTCATCTGTTGATGGACACTTGGTTT
---------------gtttatccattcatccgttgatggacacttgggtt
TG

gtttccaccttttggctactgtgaataatgctgc----agcattggtgtacaag
GTTTCCACCTTTTGGCTACTGTGAATAATGCTGC----AGCATTGGTGTACAAG
GTTTCCACCTTTTGGCTACTcTGAATAATGCTGC----AaCATTGGTGTACAAa
gtttccaccttttggctattgtgaataatgctgctatgaacattggtgtacaag

tatctatttgagccttgtgtgtttacctaggagtggaattactgggtcat
TATCTATTTGAaCCTTGTGTGTTTACCTAGGAGTGGAATTACTGGGTCAT
TATCTATTTGAGCCTTGTGTGTaTgCCTAGGAGTGGAATTACTGGGTCAT
tatctgttcgag

atggtaattctttatttatttagtttctgaggaaccactagatttttcc
ATGGTAATTCTTTATTTATTTAGTTTTCTGAGGAACCACTAGATTTTTCC
ATGGTAATTCTTTATTTA----GTTTTCTGAGaAACCACTAGATTTTTCC

acagcagctgcaccgttttacattccactagcaatgtacaagggttccaa
ACAGCAGCTGCACCGTTTTACATTCCACTAGCAATGTACAAGGGTTCCAA
ACAGCAGCTGCACCaTTTTACATTCCACTAGCAATGTtCgAGGGTTtCAA

tttctccacatccttgataaaacttgttattgttcattttaaaattata
TTTCTCCACATCCTTGATAAAACTTGTTATTGTTCATTTTTAAAATTATA
TTTCTCCACATCCTTGATAAAACTTGaTATTGTTCATTTTTAAAATTATA

gccatcctagtaggtgtgaagtggtttctccttgtggtttgatttgtgtt
GCCATCCTAGTAGGTGTGAAGTGGTTTCTCCTTGTGGTTTGATTTGTGTT
GCCATCCTAGTAGGTGTGAAGTGGTTTCTCCTTGTGGTTTGATTTGTGTT

ttcccaattaccaatgatgttgaacaAAAGATAATTTTTGAAAAGATTTT
TTCCCAATTACCAATGATGTTGAACAAAAGATAATTTTTGAAAAGtTTTT
TTCCCAATTACCAgTGcTGTTGAgCAAAAGATAAcTTTTtAAAAGATTTT

AAGAGACCTGTTCTAGTGAGAGGAAGATAATTTTATATTGCATGAAATGC
AAGAGACCTGTTCTAGTGAGAGGAAGATAATTTTATATTGCATGAAATGC
AAGAGACCTGTTCTAGTGAGAGGAAaATAATTTTATATTaCATGAAATGC

CTTGTTTTAAAAATCTGTACAGCAGTTCCCTGTTTTACCATGCTAAAAGA
CTTGTTTTAAAAATCTGTACAGCAGTTCCCTGTTTTACCATGCTAAAAGA
CTTGTTTTAAAAATCTGTACAGCAGTTCCCTGTTTTACCATGCTAAAAGA

AAGC
```

AAGC
AAGC

chr1:103811763-103811902
Alignment
Human, chimp, rhesus, L1PA15 consensus, donor
atcgcttgaacccaggaggcagatgttgcagtgagccgagatcgcaccat
ATCGCTTGAACCCgGGAGGtAGATGTTGCAGTGAGCCGAGATCGCACCAT

tgcactccagcctgggcaacaagagcaaaacttcgtcttaaaaaaaaaaa
TGCACTCCAaCCTGGGCAACAAGAGCAAAACTTCGTCTTAAAAAAAAAAA

aaaaattctaaaactgaaaaaaaccctggaaggtaatctaggaaatacca
AAA--TTCTAAAACTGAAAAAAACCCTGGAAGGTAATCTAGGAAATACCA
CA

ttctggacacaggccctagcaaggatttcatgataaagatgccaaaagca
TTCcGGACACAGGCCCTAGCAAGGATTTCATGATAAAGATGCCAAAAGCA
TTCTGGACACAGGCCCTAGCAAaGATTTCATGAcAAAGATGCCAAAAGCA

atttcaacaaaaacaaaaattgacaaatgggactgaattaatctagagag
ATTTCAACAAAAACAAAAATTGACAAATGGGACTtAATTAATCTAGAGAG
ATTTCAACAAAAA------TTGACAAATGGGAtTtAATTAAaCcAGAGAG

cttctgcacagtaaaataaactatcaacagagtaaacagtctactctgtg
CTTCTGCACAGTAAAAgAAACTATCAACAGAGTAAACAGTCTACTCTGTG
CTTCTGCACAGTAAAAgAAACTATCAACAGAGTAAACAGTCcACTCTGTG

agaatgagaggaaatatttgcaaactatgcatgtgacaaaggtttaatat
AGAATGAGAGGgAATATTTGCAAACTATGCATGTGACAAAGGTcTAATAT
AGAATGAGAGtAAATATTTGCAAACTAcGtATGTGACAAAGGTcTAATAT
taatat

ccagaatctttaagaaacttaaacaaattaacaggcaaaaagcaaacaac
CCAGAATCTTTAAGgAACTTAAACAAATTAACAGGCAAAAAGCAAACAAC
CCAGtATCTTTAAGgAACTTAAACAAATTAACAGGCAAAAAGCAAACAAC
ccagaatctataaggaacttaaacaaattaacaagcaaaaaacaaacaac

ctcattaaaaagtgagca------atgacatgaacagacacttttcaaaaaaagac
CTCATTAAAAAGTGAGCA------ATGACATGAACAGACACTTTTCAAAAAAAGAC
CTCATTAAAAAGTGgGCA------ATGA-----ACAGA-ACTTTTCAAAAgAAGAC
cccattaaaaagtgggcaaaggacatga-----acagacacttctcaaaagaagac

atatgcctggccaacaagcatatgaaaaaatgcttaacatcatgaatcat
ATATGCCTGGCCAACAAGCATATGAAAAAATGCTcAACATCATGAATCAT
ATATtCCTGGCCAAtgAGCATATGAAAAAATaCTcAACAcCATGAATCAT
atacacgtggccaacaagcatatgaaaaaatgctcaacatcactaatcat

tagaaaaatggaaatcaaaaccacaaggaagtatcattttacaccagtaa
TAGAAAAATGGAAATCAAAACCACAAtGAAGTATCATTTTACACCAGTAA
TAGAAAAATGGAAATCAAAACCACAAtGAgaTActATTTTACACCAGTAA
tagagaaatgcaaatcaaaaccacaatgagataccatctcacaccagtca

gaatgactattactaaaaagtcaaaaaataacagatgctgccaaggttgt
GAATGACTATTACTAAAAAGTCAAAAAATAACAGATGCTGCCAtGGTTGT
GAATGACTATTACTAAAAAGTCAAAAAATAgCAGATGCTGCCAAGGTTGT
gaatggctattattaaaaagtcaaaaaataacagatgctggcgaggttgc

ggagaaaaggga==actct==**<u>tTTTTTTTTTTTTT</u>**ATAGCTTAAATTTCTGAAA
GGAGAAAAGGGAACTCT**<u>TTTTTTTTTT---T</u>**ATAGtTTAAATTTCTGAAA
aGAGAAAAGG-AACTCT---------------------------------
ggagaaaagggaacgct---------------------------------
ATAGCTTAAATTTCTGAAA

ACTTTATTTTCAAAAATAAGCAAGCGTGAGAATGGAACAAAGAACACTAT
ACTTTATTTTCgAAAATAAGCAAGCaTGAGAATGtAACAAAGAACACTAT

```
--------------------------------------------------
ATTTAATTTTCGAAAACAAGCAAGCATGAGAATGGAACAAAGAACACTAT

GTTGTTCCATTAAATCTGCAGGACACAAATTCATCTACTGTTGACATTTT
GTTGTTCCATTAAATCTGCAGGACACAAATTCATCTACTGTTGAtATTTT
--------------------------------------------------
GTTGTTCCATTAAATCTGCAGGACACAAATTCATCTATTGTTGACATTTT

ATGGCATTTactcttatacactgctgttaggaatgtaaattaattcagcc
ATGGCATTTACTCTTATACACTGCTGTTAGGAATGTAAATTAATTCAGCC
--------------TATACcCTGCTGTTAGGAATGTAAATTAgTTCAGCC
--------------tatacactgctggtgggaatgtaaattagttcagcc
ATGGCATTTACT

attctggaaagc-ctgtggtgatttctcgaagaactcaaaacagaattacc
gTTCTGGAAAGC-CTGTGGTGATTTCTCGAAGAACTCAAAACAGAATTACC
ATTCTGGAAAGCagTGTGGcGATTTCTCaAAGAACTCAAAACAGAATTAtC
attgtggaaagcagtttggcgatttctcaaagaacttaaaacagaactacc

attcaacccagcaatcccattactgagtacatacccaaagggatataatc
ATTCAACCCAGCAATCCCATTACTGAGTACATACCCAAAGGGATATAATC
ATTCAACtgAGCAATtCCATTACTGAGTACATACCCAAAGGGATATAATC
attcgacccagcaatcccattattgggtatatacccaaaggaatataaat

aac-ttgtcataaagacacatgcatgcatatgtccatcacagcactattca
AAC-cTGTCATAAAGACACATGCATGCATATGTCCATCACAGCACTATTCA
AAC-cTaTCATAAAGACACATGCATGCATATGTCCATCAtAGCACTgTTtA
cattctaccataaagacacatgcacgcgtatgttcatcgcagcactattca

taatagcaaagacacggaattaacctaaatgtccatcagtgctagaccgg
TAATAGCAAAGACAtGGAATTAACCTAAATGTCCATCAGTGCTAGACtGG
TAATAGCAAgGACAtGGAATcAACCTAAATGcCCATtAGTGgTgGACtGG
caatagcaaagacatggaatcaacctaaatgcccatcaacggtagactgg

ataaagaaaatgtggtacatatactccatggaatattatgcagacataaa
ATaAAAGAAAATGTGGTA
ATAAAGAAAATGTGGTACATATACTCCATGGgAaATTATGCAGACATAAA
ataaagaaaatgtggtacatatacaccatggaatactacgcagccataaa

aaggaatgagatcgtttcctttgcagcaacatggatgaacttggaggcca
AAGGAATGAGAcCaTTTCCTTTGCAGCAACATGGAaGAACTTGGAGGCCA
aaagaacgagatcatgtcctttgcagcaacatggatggagctggaggcca

ttatcctaagttaactaatccaagaacagaaaatgagatatcatatgttc
TTATCCTAAGTTAACTAATCCAAGAAtAGAAAATGAaATATCATATGTTC
ttatcctaagcgaactaacgcaggaacagaaaaccaaataccgcatgttc

ccatttataggtgggagctaaacattgag----------cacaaagaagggaagaacaga
tCATTTATAGGTGaGAGCTAAACATTGAG----------CACAAAGAAGGGAAGAACAGA
tcacttataagtgggagctaaacattgagtacacatggacacaaagaagggaacaacaga

cattggggcctacttgagagtggagggtaggaggagagga----------tcaaaaaact
CAcTGGGGCCTACTTGAGAGTGGACGGTAGGAGGAGgGagagggagaaGATCAAAAAACT
caccggggcctacttgagggtggagggtgggaggagggtgagg------atcgaaaaact

acctttgaggtactattcttattacctgtgtgataaaacaatccatatac
AtCTTTGAGGTACTATTCTTATTACCTGTaTGATAAAACgATCCAcATAC
acctatcgggtactatgcttattacctgggtgacgaaataatctgtacac

caaacccctgtgacacacaatttacctatataacaaacctgcacatgtac
CAAACCCCTGTGACACACAATTACCg-ATATAACAAACCTGCACATGTAC
caaacccccgtgacacgcaatttacctatataacaaacctgcacatgtac
```

ccatgaacctaaaataaacgttaaaaa-TAACTATGATAAAAA-GCAAATGA
CtgTGAACCTAAAATAAACtTTAAAAAgtaataatgttaAAAAAGCAAATGA
ccctgaacctaaaataaaagttaaaaa

AAATATGAGTAACTTTTGACTTACAATTATACTTTTGGCAAATTATCCTC
AAcTATGAGTAACTTTTaACTTAaAgTTATACTTTTGGCAAATTATCCTC

AGGAAAACCACAGCATTTTTTAAAAT
AGGAAAACCAtAGCATTTTTTAAAAT

```
chr9:6027850-6028096
Alignment
Human, chimp, rhesus, consensus, donor
ACTTTTTTCAAAATATATGTAtttttatttcaataactcttccagtaca
ACTTTTTTCAAAATATATaTATTTTTTATTTCAATAACTCTTCCAGTACA
  TTTTTTCcAAAatataTATATaTTTTTTTCAATAgCTtTTCCAGTACA

agtggttttggttacatggatgaattgtacagtggtgaagtccaagatt
AGTGGTTTTGGTTACATGGATGAATTGTACAGTGGTGAAGTCCAAGATT
AGTGGTTTTGGTTACATGGATGAATTGTgtAGTGGTGAAGTCCAAGATT

ttagtgcacctgtcaccccaatagtttacattgtaccaaatatgtagttt
TTAGTGCACCTGTCACCCCAATAGTTTACATTGTACCAAATATGTAGTTT
TTAGTGCACtTGTCACCCCAATAGTTTACATTGTACtcAATATGTAGTTT

ttttattccccacctcctacat---------tcttctgagtctccaatgtTTCTTTGTT
TTTTATTCCCCACCTCCTACAT---------TCTTCTGAGTCTCCAATGTTTtttTTTG
TTTTAcTCCCCACCTCCcttccatttacccTCTTCTaAGTCTCCAATGTT--------

TT--------TTTTTTACTCAAATGGTTTTTATTTTCCTATCTGACATTTCTAACAAA
TTTgTTTgtttgtTTTACTCAAATGGTTTTTATTTTCCTATCTGACATTTCTAACAAA
----------------------------------------------------------
          TTTTTTACTCAAATGGTTTTTATTTTCCTATCTGACATTTCTAACAAA

ACGCCAGGTAGACGGAGTTAAAAAGAATCCACCGCACGAAAGGTAAACAA
ACGCCAGGTAGACGGAGTTAAAAAGAATCCACCGCACGAAAGGTAAACAA
--------------------------------------------------
ACGCCAGGTAGACGGAGTTAAAAAGAATCCACCGCACGAAAGGTAAACAA

AACAGACCCTCAGAAACTCCCTGGCAAGGATGTTCCCCTCCCCAGATTGG
AACAGACCCTCAGAAACTCCCTGGCAAGGATGTTCCCCTCCtCAGATTGG
--------------------------------------------------
AGCAGACCCTCAGAAACTCCCTGGCAAGGATGTTCCCCTCCCCAGATTGG

CCCAGTTTCACCAGCAACTGGTCTCAGCTCAGCCTTATGCCTTTCCACTG
CCCAGTTTCACCAGCAACTGGTCTCAGCTCAGCCTTATGCCTTTCCACTG
--------------------------------------------------
CCCAGTTTCACCAGCAACTGGTCTCAGCTCAGCCTTATGCCTTTCCACTG

ACAACCCCCACCCCTCCACATTCTCGTGATTCAGACCAGctaagtctcca
ACAcCCCCCACCCCTCCACATTCTCGTGATTCAGACCAGCTgAGTCTCCA
--------------------------------------------------
ACACCCCCCACCCCTCCACATTCTCGTGATTCAGACCAG

atgtttgttataccactctgtatgcctttatgtactcataacttagcttc
ATGTTTGTTATACCACTCTGTATGCCTTTATGTACTCATAACTTAGCTTC
---------ATACCACTCTGTATGCaTTTATGTACTCATAACTTAGCTTC

cacttacaagtgagaa--tacggtatttggtttttcattcctgagttacttc
CACTTAtAAGTGAGAA--TACaGTATTTGGTTTTTCATTCCTGAGTTACTTC
CACTTAtAAGTGAGAacATACaGTATTTGGTTTTTCATTCCTGAGTTACTTt

acttagaataatggcctccagctccaaccaagctgcttcataagacatta
ACTTAGAATAATGGCCTCCAGCTCCAACCAAGtTGCTTCAaAAGACATTA
ACTTAGAATAATGGC-TtCAGCTCCAACCAAGtTGCTTCAaAAGACATTc

ttttgtttttttaacggctgagtagtattccacaagtgtatatatacaac
TTTTaTTTTTTTAAtGGCTGAGTAGTATTCCACAgGTGTATATATACcAC
TTTTaTTTcTTTTAAtGGCTGAGTAGTATTCCACAGcATATATACAccAC

attttctttactcactcatcagtttatgggcacgtaggttggtcccatat
ATTTTCTTTACTCACTCATCAGTTgATGGGCACGTAGGTTGGTCCCATAT
ATTTTCTTTAtTCACTCATCAGTTgATGGGCACaTAGGTTGGTtCCATAT
```

```
ctttgcaattgtgaattgaactgtgataaacacatgcatacaaatgtctt
CTTTGCAgTTGTGAATTGAACTGTGATAAAaACATGCATACAAATGTCTT
CTTTGCAAcTGTGAATTGAACTGTGATAAACACATGCATACAAATGTCTT

ttgatataatgactgacttattttcctttgggtagatacccagtagtggc
TTGATATAATGACTGACTTATTTTCCTTTGGGTAGATACCCAGTAGTGGC
TTGATATAATGcCTGACTTATaTTCCTTTGGGTAGATgCCCAGTAGTGGt

attgctgggttaaatggtagatctacttttagttctttgagaaatctcca
ATTGCTGGGTTAAATGGTAGATCTACTTTTAGTTCTTTGAGAAATCTCCA
ATTGCTGGGTTAAATGGTAGATCTACTTTTAGTTCTTTGAGAAAcCTCCA

ttctgttttcaatagaggttgtaataatttacattttcaccagccactgc
TTCTGTTTTCAATAGAGGTTGTAATAATTTACATTTTCACCAGCCACTGC
TTCcGTTTTCcATAGAGGTTGTAATgttcc--------------------

tgatgagtgtttacttttcaccacatccacgccaacatctattttttttt-------------------
TGATGAGTGTTTACTTTTCACCACATCCACGCCAACATCTATTTTTTTTT-------------------
-------------CTTTcCACCACATCCACGCCAACATCTtTTTTTTTTTTTTttttttttttttccc

tttgacttttcaataatggccattctggctctggcaaggtggtatctcat
T--GACTTTTCAATAATGGCCATTCTGGCTCTGGCAAGGTGGTATCTCAT
----------CAATAATGGCCATTCTGGtTCaGGCAAGGTGGTAgCTCAc

tatggttttatttgcatttccctgatgattagtgatgttgagcatttt
TATGGTTTTATTTGCATTTCCCTGATGATTAGTGATGTTGAGCATTTTT
TgTGGTTTTTATTTGCATTTCCCTGATGATTAGTGATGTTGAGtATTTT-

tcatatctttgttggccatctgtatatcttcttttgagaagtatctattc
TCATATCTTTGTTGGCCATCTGTATATCTTCTTTTGAGAAGTATCTATTC
TCATATCTTTGTTGGCCATCTGTATATCTTCTTTTGAGAAGTATCcacga

atggcatttgccaactttttgatgggattatttgttttttttctt-gctga
ATGGCATTTGCCAACTTTTTTGATGGGATTATTTGTTTTTTTTCTT-GCTGA
----CATTTGCCtACTTTTTTGATGGGATTATTTGtTTTTTTTCTTGCTGA

tttgtttgagttccttgtagattctggatattagtcctctgtcagatgca
TTTGTTTGAGTTCCTTGTAGATTCTGGATATTAGTCCTCTGTCAGATGCA
TTTGTTTGAGTTCCTTGTAGATTtTGGATATTAGTCCTCTGTCAGATGCA

tagtttgcaaatattttatcccattc--tgggttgtctgcttacactgataa
TAGTTTGCAAATATTTTATCCCATTC--TGGGTTGTCTGCTTACACTGATcA
TAGTTTGCAAATATTTTATaCCATTCtgTGGGTTGTCTGCTTAtgCTGATgA

ttatttcctttgctgtgcagaagcttttaagtttaatttggttctattt
TTATTTCtTTTGCTGTGCAGAAGCTTTTTAAGTTTAATTTGGTTCTATTT
TTATTTCtTTTGCTGTGCAGAAGCTTTTaAAaTTTAATTTGGTTCTATTT

acgtgttttgtctttgttgcattcacattggggtcttggtcataaatt
ACaTGTTTTTGTCTTTGTTGCATTCACATTGGGGGTCTTGGTCATAAATT
ACaTGTTTTTGTaTTTGTTGCATTCACATTGGGGGTCTTGGTCATAAATT

cttttcttaggccaatgtccagaagaatttttcctagattttcttttaga
CTTTTCTTAGGCCAATGTCCAGAAGAATTTTTCCTAGATTTTCTTTTAGA
CTTTTCTTAGGCCAATGTCCAGAAGAgTTTTTCCTAGgTTTTCTTcTAGA

atttctttggtttcaggtcttaaagtctttaattcatctttagttgattt
ATTTCTTTGaTTTCAGGTCTTAAAGTCTTTAATTCATCTTTAGTTGATTT
ATgTCTTTGGTTTCAGGTCTTAAAGTCTTTAATTCATCTTTAGTTGATTT

ttgtgtatggtaagaaacaggggtccagtgtcattcttctacatgtggct
TTGTaTATGGTAAGAAACAGGGGTCCAGTGTCATTCTTCTACATGTGGCT
```

```
TTGTaTATGcTgAGAAACAGGGGTCCAGTtTCATTCTTCTACATGTGGCT

tgccagttatcccagcaccatttattgaatagggtgtgctttcctcaatt
TGCCAGTTATCCCAGCACCATTTATTGAATAGGGTGTGCTTTCCTCAATT
TGCCAGTTATCCCAGCACCAcTTATTGAATAGGGcaTGCTTTCCTCAATT

cttattttgtacatttgtcaaagatcagttggttgtaagtatttggct
CTTATTTTTGTACgTTTTGTCAAAGATCAGTTGGTTGTAAGTATTTGGCT
taTATTTTTGTAtgTTTTGTCAAAGATCcaTTGGTTGTAAGTATTTGGCT

ttatttctgggttctctattctgttcaattggtccatgtatccactttta
TTATTTCTGGGTTCTCTATTCTGTTCAATTGGTCCATGTATCCACTTTTA
TTATTTCTGGGTTCTCTATTCTGTTCAgTTGGTCCATGTATCCACTTTTA

taccagtaccatgctgttttggttaccatagcctcgtagtataattataa
TACCAGTACCATGCTGTTTTGGTTACCATAGCCTCGTAGTATAATTATAA
cACCAacACCATGCTGTTTTGGTTACtgTAGCCTCGTAGTATAATTATgA

tttgaaattaggtaatgtgatgcctccagatttgttctttttgcttaggg
TTTGAAATTAGGTAATaTGATGCCTCCAGATTTGTTCTTTTTGCTTAGGG
TTTGAAATTAGGTAATGTGATGCCTttgGAcTTGTTCTTTTTGCTTAGGa

ttgctttggttactcaagcttttttttttttatt---------------ccatatgaattttagg
TTGCTTTGGTTACTCAAGCTTTTTTTTTTT-ATT---------------CCATATGAATTTTAGG
ttgttttggtaactcaggctcttttttttttttTTTTTTTTTTTTATTCCATATGAATTTTAGG

attttttttctaatttt-at--gaaaaatgatgtgagtattttctttcttactt
ATTTTTTTtCTAATTTTAT--GAAAAATGATGTATTTTCTTTCTT----ACTT
ATTTTTTTtCTAATTTTATtaGAAAAATGATGTGAGTATTTTCTT----cCTT

ttttttttttttctatttttgagatggagtttcactcttgtcgcccag
TTTTTTTTTTTT-CTATTTTTTGAGATGGAGTTTCACTCTTGTCGCCCAG
TTTCTttcccctcTTTTTa--GAGAcGGAGTTcCACTgTTGTtGCCCAG

gctggagtgcaatggcgtgatctcagctcac
GCTGGAGTGCAATGGCGTGATCTCgGCTCAC
GCTGGAGTaCAATGGCGTGATCTCAGCTCAC
```

chr4:57321413-57322533
Human
Alignment
Human, chimp, rhesus, consensus, donor
TCTTCTTTTGTTTCTTTATGCCTGGTGGGAAGGAAGTCCTCCTCCCGGTA
TCTTCTTTTGTTTCTTTATGCCTGGTGGGAAGGAAGTtCTCCTCCCGGTA
TtTTCTTTTGTTTCTTTATGCCTGGTGGGAAGGAAGTCCTCCTgCCGGTA

Gaactgcctttgcaaaaattataacagtgagacaattatggcagtgaagg
GAACTGCCTTTGCAAAcATTATAACAGTGAGACAATTATGGCAGTGAAGG
GAACTGCCTTTGCAAAAATTtTAACAGTGAGACAATTATGGCAGTGAAGG

acagctgatctggccaacctccatcttacctttggacttcaggctgccct
ACAGCTGATCTGGCCAACCTCCATCTTgCCTTTGGgCTTCAGGCTGCCCT
AgAGCTGATCTGGCCAACCTCCgTCTTgCCTTTGGgCTTCAaGCTGCCCc

taattaatcctaggcttaggccaagctaactttgggagacatctagtttC
TAATTAtTCCTAGGCTTAGGCCAAGCTAACTTTGGGAGACATCTAGTTTC
TAATTAtTCCTAGGCTTAGGCCAAGCTAACTTTGGGAGACAcCTAGTTTC

TTTTTTTTTTTTTTTCTTT-GCAAGAGTCTGCTTTTAATTTTTATTTTATC
tTTTTTTTTTTTTTTTCTTTGCAAGAGTCTGCTTTTAATTTTTATTTTATC

---------------CTTT-GCAAGAGTCTGCTTTTAATTTTTATTTTATC

AAAGAGAATCACTTAAGTCAGTATTCATTTGAGTAATCTGGCTTAAATTA
AAAGAGAATCACTTAAGTCAGTATcCATTTGAGTAATCTGGCTTAAATTA

AAAGAGAATCACTTAAGTCAGAATTCATTTGAGCAATCTGGCTTAAATTG

GGGAACAGTCAACACTGGATTGCCATTTTAAGTTTCCAGATTAATACAAA
GGGAACAGTCAACACTGGATTGCCATTTTAAGTTTCCAGATTAATACAAA

GGGAATAGTCAACACTGGATTGCCATTTTAAGTTTCCAGATTAATACAAA

ATTAGTGTCAATAGTATAGGTTAAGCTGAAAACTTTTATAGTCAGAAGTA
ATTAGTGTCAATAGTATAGGTTAAGCTGAAAACTTTTATAGTCAGgAGTA

ATTAGTGTCAATAGTATACGTTAAGCTGAAAACTTTTATAGTCAGGAGTA

CGGTGAGACTCAGTTTTACCTATTAAATCAGTTTTATCTATAAATCTGAT
tGGTGAGACTCAGTTTTACCTATTAAATCAGTTTTATCTATgAATCTGAT

CGGTGAGACTCAGTTTTACCTATTAAATCAGTTTAATCTGTAAATCTGAT

TTTGCTGTAAAATCACGCTAATATTACACGCTTCAAAAAA-CATGCTTTTC
TTTGCTGTAAAATCACGCTAATATTACACGCTTCAAAAAA-CATGCTTTTC

TTTGCCGTAAAATCACGCTAATATTACACACTTCAAAAAAACATGCTTTTC

CTTTGAGAAACTTTCTACATCTCAGCCGAAATGCCTTAATTTCATAAAAT
CTTTGAGAAACTTTCTACATCTCAGCCGAAATGCCTTAATTTCATAAAAT

CTTTGAGAAACTTTCCACATCTCAGCCAAAATGCCTTAATTTCATAAAAT

AAATGTTACAATACAAAAATCTTTTGTAAAAATATCAACTATATATGAAT
AAATGTTACAATACAAAAATCTTTTGTAAAAATATCAACTATATATGAAT

AAATGTTACAATACAAAAATCTTTTGTAAAAATATCAACTATATATGAAT

TAAAATCAATCTCTTCTTTTGTTAAAAGTTACCTGTAATTAAGAATGTCA
TAAAATCAATCTCTTCTTTTGTTAAAAGTTACCTGTAATTAAGAATGTCA

```
TAAAATCAATCTCTTCTTTTGTTAAAAGTTACCTGTAATTAAGAATGTCA

GAACAACTGAGCCCCCATTCTAAAGTTTCTGTGGTGAAGTCATCTGTATT
GAACAACTGAGCCCCCATTCTAAAGTTTCTGTGGTGAAGTCATCTGTATT
--------------------------------------------------
GAAGAACCGAGCCTCCATTCTAAAGTTTCTGTGGTGAAGTCATCTGTATT

TCCTAGGTCAGTAAACCCAACAACATAATCTTGTGTTTTCCCATCTTTTA
TCCTAGGTCAGTAAACCCAACAACATAATCTTGTGcTTTCCCATCTTTTA
--------------------------------------------------
TCCTAGGTCAGTAAACCCAACAACATAATCTTGTGTTTTCCCATCTTTTA

CCAGTGCTAGTATGGGAATGACTTTGATATGCAGTCTCTCACAAAGGAAA
CCAGTGCTAGTgTGGGAATGACTTTGATATGCAGTCTCTCACAAAGGAAA
--------------------------------------------------
GCAGTGCTAGTGTGGGAATGACTTTGATATGCAGTCTCTCACAAAGGAAA

GGTGCTTTTTCCACATTCAGCTTCAAAAATTTGGTCTCGAGGTGTTTCTC
GGTGCTTTTTCCACATTCAGCTTCAAAAATTTGGTCTCGAGGTGTTTCTC
--------------------------------------------------
GGTGCTTTTTCCACATTCAGCTTCAAAAATTTGGTCTCGAGGTGTTTCTT

GGACAATATCGCCAGAAATCTGTCTAGTATTTTACACCTGAATGTGGAGT
GGACAATATCGCCAGAAATCTGTCTAGTATTTTACACCTGAATGTGGAGT
--------------------------------------------------
GGACAATATTGCCAGATGTCTGTCTAGTATTTTACACCTGTAAGTG
                                        aatgtgGAGT

CTCTGTAGAAATGGCAAACCACTTTTTTACTCTCCTTGACTTCTTGAAAA
CTCTGTAGAAATGGCAAACCACTTTTTTACTCTCCTTGACTTCTTGAAAA
--------------------------------------------------
CTCTGTAGAAATGGCAAACCACaTTTTcACTCTCCTTGACTTCTTGAAAA

AAGTCTCTTTCACTAGGGATTTCTCTGTATTCCCCATGTCCTTTGGAAAG
AAGTCTCTTTCACTAGGGATTTCTCTGTATTCCCCATGTCCTTTaGAAAG
--------------------------------------------------
AAGTCTCTTTCACTAGGGATTTCTCTGTATTCCCCATGTCCTTTaGAAAG

CCATTCTTGTTTCTGCTGTTGAGCTTTCCTTAGTGCCTCGAGTCTCTTTT
CCATTCTTGTTTCTGCTGTTGAGCTTTCCTTAGTGCCTCGAGTCTCTTTT
--------------------------------------------------
CCATTCTTGTTTCTGCTGTTGAGCTTTCCTTAGTGCCTgGAGTCTCTTTT

CTTTAATGTGTTCCAATTCATCCTCATCCATCTGATCCAGTTTTTGAATT
CTTTAATGTGTTCCAgTTCATCCTCATCCATCTGATCCAGTTTTTGAATT
--------------------------------------------------
CTTTAAgGcGTTCCAATTCATCCTCATCCATCTGATCCAGTTTTTGAATT

TCAGAATCCAAATGTTCTTCCACCAGTTTGGTAGTCTGAAGCAGCTGATT
TCAGAATCCAAATGTTCTTCCACCAGTTTGGTAGTCTGAAGCAGCTGATT
--------------------------------------------------
TCAGAATCCAAATGTTCTTCCACCAGTTTGGTAGTCTGAAGCAGCTGATg

CTCCAGGACTTTGGAAAACATGTCAACAGATGCATCAGCTTCCATTCTTC
CTCCAGGACTTTGGAAAACATGTCAACAGATGCATCAGCTTCCATTCTTC
--------------------------------------------------
CTCCAGGACTTTGGAAAACATGTCAACAGATGCATCAGCTTCCATTCTTC

TGAATGGTACAGAGTTCAGCCTGGGTGCTGAGCTCTCGGTGATGCCTGAG
TGAATGGTACAGAGTTCAGCCTGGGTGCTGAGCTCTCGGTGATGCCTGAG
--------------------------------------------------
caAATGGTACAGAGTTCAGCCTGGGTGCTGAGCTCTCGGTGAcGCCTGAG
```

AAGTGAAAGAGCAGCAGTTTCAAAAAACAGGTCCACTCCGGCTTTAACCT
AAGTGAAAGAGCAGCAGTTTCAAAAAACAGGTCCACTCtGGCTTTAACCT

AAGTGAAAGAGCAGCAGTTTCAAAAgACAcGTCCACTCCGGCTTTtgCCT

TGCAGTAGCTGCCAGCGGCTAgggagacatctagtttctagtttaattga
TGCAGTAGCTGCCAGtGGCTAGGGAGACATCTAGTTTCTAGTTTAATTGA
--------------------------------------TAGTTTAAcTGA
TGCAGTAGCTGCCgGCGGCTg

tagcaacccttcccaaaaattcaaccgcctttgtaaagctaatgagagac
TAGCAACCCTTCCCAAAAATTCAACCGCCTTTGTAAAGCTAATGAGAGAC
TAGtA--CCTTCCCAAAAATTCAACCGCCTTTGTAAAGCTAATGAcAGAC

cacaagactaggatgatagagaagcctgaattctgctaaggtgtagacat
CACAAGACTAGGATGATAGAGAAGCCTGAATTCTGCTAAGGTGTAGACAT
-ACAAGACTAGGATGATAGAGAAGCCTGAATTCTGCTAAaGTGTAGACAT

aaatgattgccagccattattctggggatcacaagatatccaacttcccc
AAATGATTGCCAGCCATTATTCTGGGGATCACAAGATATCCAACTTCCCC
AAATGATTGCCAGCCATTATTCTGGGGATCACAAGATATCCAACTTCCCC

aattactcctccagataccatcactattgtataacctatgattggccttt
AATTACTCCTCCAGATACCATCACTATTGTATAACCTATGATTGGCCTTT
AATTACTCCTCCgGATAaCATCACTATTGTATAACCTAcGATTGGCCTTT

ggagatatcttttcaggtttttgcatgtcacaaccgatggctccacctg
GGAGATgTCTTTTCAGGTTTTTGCATGTCACAACCGATGGCTCCACCTG
GGAGATATCTTTTCAGGTTTTTGCATGTCACAgCCGATGGCTCCACCTG

gacttggtcacggttcctgtggccccagccagaaggggctcagtgaggat
GACTTGGTCACaGTTCCTGTGGCCCCAGCCAGAAGGGGCTCAGTGAGGAT
GACTTGGcCACaGTTCCTGTGGCCgCAGCCAGAAGGGGCTCAGcGAGGAT

gaagaccatttgccacacccctatgattgcgcccccaaccaatcagcagc
GAAGACCATTTGCCACACCCCTATGATTGCGCCCCCAACCAATCAGCAGC
GAAGACCATTTGCCACACCCCTgTGATTGCGCCCCCAACCAATCAGCAGC

aagcacgattgcctagctaccccttcccctaaactatctttgagaaaccc
AAGCACGATTGCCTAGCTACCCCTTCCCCTAAACTATCTTTGAGAAACCC
AAGCACaATTGCCTAGCTACCgCTTCCCCTAAACTATCTTTGAGAAACCC

tagcctttgaatttccagagagatcgatttgagtaataactctgtctccc
TAaCCTTTGAATTTCCgGAGAGATCGATTTGAGTAATAACTCTGTCTCCC
TAGCCTTTGAATctcttt-------GATTTGAGTAATAACTCTGTCTgCC

aagtgacatgatcagcctcgtgtctattaaactctttctttattgcaatg
AAGTGACATGATCAGCCTCGTGTCTATTAAACTCTTTCTTTATTGCAATG
AcGTGACATGgcCAGCCTtGTGTCTATTAAACTCTTTCTTTATTGCAATG

ccgtcatctcagtgaattggtttttgtctgcgcagggggcaggaagaacc
CCGTCATCTCAGTGAATTGGTTTTTGTCTGtGCAGGGGGCAGGAAGAACC
CtGTCATCTC-GTGAATTGGTTTTTGTCTGtGCAGcaGGCgGGAAGAACC

cactgggtggttacaCCTGGGCAGGTGCTGCTTCCCTGAAGGACCAACTA
CACTGGGTGGTTACACCTGGGCAGGTGCTGCTTCCCTGAAGGACCAACTA
CACTGGGTtGTTACACCTGGGCAGGcGCTGCTTtCCTGAAGGACCAACTA

CGCCATGATGAGGAAAAGGGTAGtgtgtgggtacttttcgttctgtccca
tGCCATGATGAGGAAAAGGGTAGTGTGTGGGTACTTTTCGTTCTGTCCCA
gGCCgTGATGAGGAAA-GGGTAGTGTGTGaGTACTTTTCGTTCTGTCCCA

ccagatccactttgctccct

CCAGATCCACTTTGCTCCCT
CCAGATCCACTTTGCTCCCT

```
chr12:37804289-37804455
Alignment
Human, chimp, rhesus, MER41B consensus, donor
tcaaggtatctgcTTGCAGCAGATGGTTTGAA--------------------TCATGGAAAATGcagagg
TCAAGGTATCTGCTTGCAGCAGATGGTTTGAA--------------------TCATGGAAAATGCAGAGG
TCAAGGTATCTGCTTGCAGCAGATGGTTTGAAccattgattttcaatgcattt----------TGCAGAGG
                                                              cagagg

catttgaaccagagcaactccatcttgaatagagactgggtaaaataagg
CATTTGAACCAGAGCAACTCCATCTTGAATAGAGACTGGGTAAAATAAGG
tATTTGAACCAGAGCAACTCCATCTTGAATAGAGgCTGGGTAAAATAAGG
cgtttgaaccagagcaactccatcttgaataggcgctgggtaaaatragg

ctgagacctgctgggctgcattcccaggaagttag-gcattcttactcaca
CTGAGACCTGCTGGGCTGCATTCCCAGGAAGTTAG-GCATTCTTACTCACA
CTGAaACCTGCTGGGCTaCATTCCCAGGAAGTTAa-GCATTCTTACTCACA
ctgaracctactgggctgcattcccagacggttaaggcattctaagtcaca

ggatgagataggagatcagcaaaagatacaggtcaggaagac--tgctgata
GGATGAGATAGGAGATCAGCAcAAGATACAGGTCAcGAAGAC--TGCTGATA
GGATGAGATAGGAGATCAGCAcAAGATACAGGTCAcaAAGAC--TGCTGATA
ggatgagataggaggtcggcacaagatacaggtcataaagaccttgctgata

aaacaggatgaggtaaagaaggcatctgaaaccactgaaggc-aagatggc
AAACAGGATGcGGTAAAGAAGGCATCTGAAACCACTGAAGGC-AAGATGGt
AAACAGGATGcGGTgAAGAAGaCATCTGAAACtACTGAAGGC-AAGATGGt
aaacaggttgcagtaaagaagccggcyaaaacccaccaaaaccaagatggc

gataaaaagtgttttctagtcatcctcactgctcattatatgttaattat
GATAAAAAGTGTTTTCTAGTCATCCTCACTGCTCATTATATGcTAATTAT
GATAAAAAGTGTTTTCTgGTtgTCCTCACTGtTCATTATATGTTAATTAT
cacgagagtgacc-tctggtcgtcctcactgctcattatatgytaattat

aatgcattagcatgctaaaagtcactcccaccagcaccatgacagtttac
AATGCATTAGCATGCTAAAAGTCACTCCCACCAGCACCATGACAGTTTAC
AATGCATTAGCATatTAAAAGTCACTCCCACCAGCACCATGACAGTTTAC
aatgcattagcatgctaaaagacactcccaccagcaccatgacagtttac

aaatgccatggcaatgttagaaagttaccctatatggtctaaaaagagga
AAATGCCATGGCAATGTTAGAAAGTTACCCTATAcGGTCTAAAAAGAGGA
AAATGCCATGGCAATGTTAGAAAGTTACCCTATATGtTCTAAAAAGAGGA
aaatgccatggcaacgtcaggaagttaccctatatggtctaaaaaggggga

gaaactctcactttcaggaaatctccacccctttcctggaaaactcatgc
GgAACTCTCACTTTCAGGAAATCTCCACCCCTTTCCTGGAAAACTCATGC
GgAACcCTCACTTTCAGGAAATCcCCACtCCTTTCCTGGAAAACTCATGg
ggaaccctcagttccgggaattgcccgcccctttcctkgaaaaytcatga

ataatccatgccttgtttagcacataatcaagaaataactgtaagtattc
ATAATCCATGCCTTGTTTAGCAtATAATCAAGAAATAACTGTAAGTATTC
ATAATCCATGCCTTGTTTAGCACATAATCAAGAAATAACTaTAAGTATTC
ataatccaccccttgtttagcatataatcaagaaataaccataaaaatrg

ctagtcgtgcagcccatgctgctgctctgtctatggaataatcattcttt
tTAGTCGTGCAGCCCATGCTGCTGCTCTGTCTATGGAATAATCATTCTTT
tTAGTCGaGaAGCCCATaCTGCTGCTCTGTCTATGGAATAATCATTCTTT
gcaacca-gcagccctcggggctgctctgtctatggagtagccattCttt

ttttttttttttcAAATTTTGACTCATTTATTGAATTAAAATTATACT
TTTTTTTTTT----CAAATTTTGACTCATTTATTGAATTAAAATTATACT
T-------------------------------------------------
t-------------------------------------------------
              CAAATTTTGACTCATTTATTGAATTAAAATTATACT
```

```
ACGTATAAAACAGCAATCTATATGCAG--TGATTTAATGCATCATAATATAT
AtGTATAAAACAGCAATCTATATGCAG--TGATTTAATGCATCATAATATAT
----------------------------------------------------
----------------------------------------------------
ATGTATAAAACAGCAATCTATATGCAGAGTGATTTAATGCA---TAATATAT

ACATTTATCTTTACAAAGTCAGGATGCAAGTTCCAATGATTATGACTATA
ACATTTATCTTTACAAAGTCAGGATGCAAGTTCCAATGATTATGACTATA
--------------------------------------------------
--------------------------------------------------
ACATTTATATTTACAA-TACAGGATGCAAGTTCCAATGACTATGACTATA

AAATTTATATTAAACATTTCCATAAGTGACTTCggaataatcatcctttt
AAATTTATATTAAACATTTCCATAAGTGACTTCGGAATAATCATtCTTTT
--------------------------------------------------
--------------------------------------------------
AAATTTATATTAAACATTTCCATAAGTGA

attgctttactttctt--taaacttgctttcactttactctgtggacttgac
ATTGCTTTACTTTCTT--TAAACTTGCTTTCACTTTACTCTGTGGACTTGAC
ATTGCTTTACTTTCTT--TAAACTTGCTTTCACTTTACTCTaTGGAgcTGAC
attcctttactttcttaataaacttgctttcactttactctrtggactcgcc

ccaaattctttcttgtgcaaggtccaagaatgctttcgtgaggtgtggat
CCAAATTCTTTCTTGTGCAAGGTCCAAGAATGCTTTCGTGgGGTGTGGAT
CCAAATTCcTTCTTGTGCAAGGTCCAAGAAcGCTTTCtTGtGGTGTGGAT
ctgaattctttcttgcacragatccaagaaccctctcttggggtctggat

tgcgtcccgtttctggtaacaTcttgactagaaaaggctctgcctcta
TGCGTCCCcTTTCTGGTAACATCTTGACTAGAAAAGGCTCTGCCTCTA
TGtGTCCCcTTTCTGGTAACATCTTGACTAGAAAAGGtTCTGCC
cgggacccctttcttgtaaca
```

chr14:28665689-28665837
Alignment
Human, chimp, rhesus, MER45B consensus, donor

```
GTAGCACATCAAAAGTCTTGCTCAATATCCACACATGGGTTACCTGAAGG
GTAGCACATCAAAAGTCTTGCTCAATATCCACACATGGGTTACCTGAAGG
GTAGCACATCAAAAGTCTTGtTCAATATCCACACATGGGTTACCTGAAGG

AGAAAGCACAGAGTATGGTGAGTAGTAAAACTAAGGAGGGTGCTGTGAAA
AGAAAGCACAGAGTATGGTGAGTAGTAAAACTAAGGAGGGTGCTGTGAAt
AGAAAGCACAGAGTATGGTGAGTAGTAAAACTAAGGAGGGTGctgtgaaa

----------------------------------------------
----------------------------------------------
agcacagagcatggtgagtatcaaaaccaaggagggtcCTGTGAAA

AGCCATTTGGTTtcagaaaggccttgcacttggtttaacgctttgttgtt-
AGCCATTTGGTTTCAGAAAGGCCTTGCACTTGGTTTAACGCTTTGTTGTT-
AGCtATTTGGTTTCAGAAAGGCCTTGCAtTTGGccTAAtGCTTTGTTGTTg

ccatcttgaaga-ccataattattttatcttggaatttgtaattcgtaggt
CCATCTTGAAGAaCCATAATTATTTTATCTTGGAATTTGTAATTCGTAGGT
CCATCTTGAAGA-tCtTAATTATTTTgTCTTtGAgTTTGTAAcTtGTAGGT

gaagttcagtgagacaatataatacatgagtgagcagtagcggtatgtgc
GAAGTTCAGTGAGACAATATAATACATGAGTGAGCAGTAGCGGTATGTGC
GAAGTTCAGTGAGACA--------------TGAGCAGTAGtGGTATGTGC

catatgtgtgttcattgccattccttgctgcatggtcacacatagcattt
CATATGTGTGTTCATTGCCATTCCTTGCTGCATGGTCACACATAGCATTT
CAcATGTGTGTTCATTGCCATTaCTTGCTGCATGGTCACAtAcAGCATTT

ccagtgtctcatgaactatatacagaattccagtggtcccatatcacatg
CCAGTGTCTCATGAACTATATACAGAATTCCAGTGGTCCCATATCACATG
CCAGTGTCcCATGAACTATATACAGAATTCtAGTGGTCCCATATCACATG

agagtttagcaagactcaaagttagtacaaagtgttttgcatctatgact
AGAGTTTAGCAAGACTCAAAGTTAGTACAAAGTGTTTTGCATCTATGACT
AGAGTTTAGCAAGACTCAAAGTTAGTAaAAAGTGTTTTGCAcCTATGACT

gagtgagggcactggcagctctgagaagctgagctttccctcagaaacgg
GAGTGAGGGCACTGGCAGCTCTGAGAAGCTGAGCTTTCCCTCAGAAgCGG
GAGTGAGGGCACTGGCAGCTCTGAGAAGCTGAGCTTTCCaTtgGAAAtGG

aatttgggtcaaatgaaagaaggcagtg---ttttaagaaGTAGCTCTTGACT
AATTTGGGTCAAATGAAAGAAGGCAGTG---TTTTAAGAAGTAGCTCcTGACT
AATTTGGGTCAAATGAAAGAAGGCAGTGgagTTTTAAGAA-------------
     tcgaacgcagaaagaaggcaatggcattctaagaa-------------
                                        GTAGCTCCTGACT

CTTAGGGATGGATTTTGAGGAAAAAACAAGACTAAACAAAAACATGTAGC
CTTAGGGATGGATTTTGAGGAAAAAACAAGACTAAACAAAAACgTGTAGC
--------------------------------------------------
CTTAGGGATGGATTTTGAGGAAAAAACAAGACTAAACAAAAACATGTAGC

TCCCTATGTTTTCTCTCTAGGTTGTTGGACTGAAATATGCATTTTAGCTT
TCCCTATGTTTTCTCTCTAGGTTGTTGGACTGAAATATGCATTTTAGCTc
--------------------------------------------------
TCCCTATTTCTTCTCTCTAGGTTGTTGGACTGAAATATGCATTTTAGCTT

TGTGTGTTTCTAAAATAAACATTTCTAAAATTTACAGTaaaaaaaa--gcat----
TGTGTGTTTCTAAAATAAACATTTCTAAAATTTACAGTAAAAAAAAaaGCAT----
------------------------------------------------gcataaac
------------------------------------------------acacgaac
```

TGTGTGTTTCTAAAATAAACATTTCTAAAATTTACAGTAA

aatcaaagaatcctatcattttctttcttacttatgtaacttgtctgtat
AATCAAAGAATCCTATCATTTTCTTTCTTACTTATGTAACTTGTCTGTAT
AATCAAAGAAcCCTATCATTTTCTTTCTTACTTATGTAACTTGTCTGTAT
gaccaaggaaccctatcatatcctttcttactcgtgttacttccctgtat

taaccaaccacttttaatgaaaatgatggcatagaa----agggaaagacagga
TAACCAACCACTTTTAATGAAAATGATGGCATAGAA----AGGGAAAGACAGGA
TAACCAACCACTTTTAtTGAAAATGATGtCATAGAA----AGGGAAAGAtAGGA
tagccaaccacttacgctgaaaatgatgacatagaaggaaagggaaagataggg

taacccatatctccttttcctttcagcccaccttta----tgagtaagccaaag
TAACCCATATCTCCTTTTCCTTTCAGCCCACCTTTA----TGAGTAAGCCAAAG
TAtCCCATATaTCCTTTTCCTTTCAGCCCACCTTTA----TGAGTAAGCCAAAG
caacccatagttccttttcctttcagtccttccttactcatcagtaagccgaag

atagagaatgtcggtggaatgcaagcacagcaaaacgtaaaataaaaaca
ATAGAGAATGTCGGTGGAATGCAAGCACAaCAAAACGTAAAATAAAAACA
ATAGAGAATGTCaGTGGAATGCAAGCAtAGCAAAAtGTAAAATAAAAACA
gtagagagtgttggtagaatgtgcgcgtatcaagaagtgaaataaaaaca

tttgagttggttttatgcagg-ttttgactgtaatggtaagaatgaaacat
TTTGAcTTGGTTTTATGCAGG-TTTTGACTGTAATGGTAAGAATGAAACAT
aTTGAGTTGGTTTTATGCAGa-TTTcGACTGTAATGGTAAGAAcGAAACAc
gttgagttagttttgtgcagcgtttccactgttctggtaagaacgaaatac

acatacaagtactagctatgaactgtgcagtttcagtaatgttgcactag
ACATACAAGTACTAGCTATGAACTGTGCAGTTTCAGTAATGTTGCACTAG
ACAcgCAAGTACTAGCTATGAACTGTGCAGTTTCAGTAATGTTGCACTAG
atatgcatgtacgagctacgaa

ttaagtgtgcttgtatttgtatttaaagctggcattcattgcacaatatg
TTAAGTGTGCTTGTATTTGTATTTAAAGCTGGCATTaCACAATATgaatG
TTAAGTGTGCTTaTATTTGTATTTAAAaCTGGCATTaCACAATATG----

aacatgaatggtaaacacttgttaccaatttaatttttttaaatttttgtt
AACATGAATGGTAAACACTTGTTACCAATTTAATTTTTTAAATTTTTGTT
AAgATGAATGGTAAACACTaGTTACCA-------TTTTTTtatttgtaTT

tgcttagaatggcattaaatagtaaatctaaagcaaaaaggcttttcatt
TGCTTAGAATGaCATTAAATAGTAAATCTgAAGCAAAAAGGCTTTTCATT
TGCTTAGAAgGagATTAAATAGTAAATCTgAAaCAAAAAGGCTTTTCATT

ttcattttgcatttagccctacaaattacatatctagccttAGATGGCGG
TTCATTTTGCATTTAGCCCTACAAATTACATATCTAGCCTTAGATGGCGG
TTCATTTTGCATTTAGCCCTACAAATTAaATATCTAGtCTTAGATGGtGG

ACCATGTAATCTGATTTGGGACAGTAAGATTGATGATAATGTTTGCTACG
ACCATGTAATCTGATTTGGGACAGTAAGATTGATGATAATGTTTGCTACG
ACCATGTAATCcGATTTGGGACAGTAAGATTGATGATAATGTTTGCTACG

AGCCACAAGGAAAAATGGTCCTTTG
AGCCACAAGGAAAAATGtTCCTTTG
AGCCACAAGGAAAAATGtTCCTTTG

chr1:100451345-100452114
Alignment
Human, chimp, rhesus, MIRb consensus, donor

```
cctgaaggtaatgaaagggaaccactgaaaagaatcagagaatattacag
CCTGAAGGTAATGAAAGGGAACCACTGAAAAGAATCAGAGAATATTACAa
CCTaAAGGcAATGAAAGGGAACCACTGAAAAGAATCAGAGAATATTACAa

taatttgatttacggaagaccagtcttggtgcagtgtagagaaaggactg
TAATTTGATTTACaGAAGgCCAGTCTTGGTGCAGTGTAGAGAAAGGACTG
TAATTTGATTTACaGAAGACCAGTCTTGGTGCAGTGTAGAGAAAGGACTG

gaCATGTTGGCATTACAGACATCagtatgtccttctgagtaaaacagacc
aACATGcTGGCATTACAGACATCAGTATGTCCTTCTGAGTAAAACAGACC
aACATGcTcGCATTAgAGACATCAGgATGTCCTTCaGAGTAgAACAGACC

agaacctgaaagagcttctccacctgttagctgtaagtccttaacaagtt
AGAACCTGAAAGAGCTTCTCCACCTGTTAGCTGTAAGTCCTTAACAAGTT
AGAAtCTGAAAGAGCTTCTCCACCTGTTAGCTaTAAGTCCTTAACAAGTT
                                            caagtc

tctcaacctctctgagccccaatttccttaaatggaaagtggagacagta
TCTCAACCTCTCTGAGCCCCAATTTCCTTAAATGGAAAGTGGAGACAGTA
TCTCAACCTCTCTGAGCCCCAATTTCCTTAAATGGAAAGTGGgGACAGTA
acttaacctctctgagcctcagtttcctcatctgtaaaatggggataata

atatttacatgtcagggttactctgagaattaa--gagataatgGGCCCTGC
ATATTTACATGTCAGGGTTACTCTGAGAATTAA--GAGATAATGGGCCCTGC
ATATTTACATGTCAGGGTTACT-TGAGAATTAA--GAGATAATG--------
atacctacctcgcagggttgttgtgaggattaaatgagataatg--------
                                             GCCCcGC

CCCGCGTCTGCCTCAGAGGGGCCCGAGCCACCCGGGGCGTGCGCCGCGTC
CCCGCGTCTGCCTCAGAGGGGCCCGAGCCACCCGGtGCGTGCGCCGCGTC
--------------------------------------------------
CCCGCGTCTGCCTCAGAGGGGCCCGAGCCACCCGGTcCG----CCGCGTC...

CCCGCCGGGGCCGACAGAGCCGA---GCCGGGCCGCCATGGACCACAAGACTC
CCCGCCGGGGCCGACAGAGCCaA---GCCGGGCCGCCATGGACCACAAGACTC
-----------------------------------------------------
CCCGCCGGGGCCGACcGAGCCGA...GCCGGGCCGCCATGGACCACAAGcCgC

TGCTGCAGGAGCGGCCGCCCGCCTACAACCTGGAGGCCGGCCAGGGCGAC
TGCTGCAGGAGCGGCCGCCCGCCTACAACCTGGAGGCCGGCCAGGGCGAC
--------------------------------------------------
TGCTGCAGGAGCGGCCGCCCGCCTACAACCTGGAGGCCGGCCAGGGCGAC

TACGCGTGAGGCCCGCACGGCTACGGCGCCATCCCCGCCGCGCCCCCGCA
TACGCGTGcGGCCCGCACGGCTACGGCGCCATCCCCGCCGCGCCCCgGCA
--------------------------------------------------
TACGCGTGcGGCCCGCACGGCTACGGCGCCATCCCCGCCGCGCCCCCGCc

GCCGCCCTACTCCTACCTCGTCACACGGACACCCACCCACCATCCCAGGG
GCCGCCCTACTCCTACCTCGTCACACGGACACCCACCCACCATCCCAGGG
--------------------------------------------------
GCCGCCCTACcCCTACCTCGTCACA...--ACCCACCCACCATCCCAGGG

TCTACAACATCCACAGCTGGACCGTCATCCGCTATCCTACCAACTCTATC
TCTACAACATCCACAGCcaGACCGTCAcCCGCTATCCTACCAACTCTgTC
--------------------------------------------------
TCTACAACATCCACAGCcGGACCGTCAcCCGCTATCCTgCCAACTCTATC

GTGGTCGTGGGAGGCTGTCCCGTCTGCAGGGTCGGGGGGCTGCAGGACTG
GTGGTgGTGGGAGGCTGTCCCGTCTGCAGGGTCGGGGGGCTGCAGGACTG
```

```
--------------------------------------------------
GTGGTCGTaGGAGGCTGTCCtGTCTGCAGG
                       CTGCAGGGTTGGGGTGCTGGAGGACTG

CTTCACCTTCCTGGGCATCTTCCTGGCCATCATCCTGTTCCCCTTTGGGT
CTTCACCTTCCTGGGCATCTTCCTGGCCATCATCCTGTTCCCCTTTGGGT
--------------------------------------------------
CTTCACCTTCCTGGGCATCTTCCTGGCCATCATCCTCTTCCCCTTTGGGT

TCATTTGCTGTTTTGCCTTGAGGGAGCGAAGATGCCCCAACTGTGGAGCC
TCATcTGCTGTTTTGCCTTGAGGaAGCGAAGATGCCCCAACTGTGGAGCC
--------------------------------------------------
TCATTTGCTGTTTTGCCTTGAGGAAGCGACGATGCCCCAACTGTGGAGCC

ACCTTCGCTTAAAGGGAACACCAGGCTCGGCCTTTCCTACACCCAGCTCT
ACCTTCGCTTAAAGGGAACACCAGGaTCGGC-TTTCCTACACCCAGCTCT
--------------------------------------------------
ACCTTCGCTTAAAGGGAACACCAGGCCCGGC-TTTCCTACACCCAGCTCT

CTTTTTCTAATGTAAATGTTGTGTTCAATAGTTTTATTTGATTAAGCTTC
CTTTTTCTAATGT------TGTGTTCAATAGTTTTATTTGATTAAGCTTC
--------------------------------------------------
CTTTTTCTAATGTAAATGTTGTGTACAATAATTTTATTTGATTAAGCTTC

AGGACTGTTTTGTAAAGCGAGGTGGGACCGATGTGGCACACGCCAGCTGC
AGGACTGTTTTGTAAAGCGAGGTGGGACCGATGTGGCACACGCCAGCTGC
--------------------------------------------------
AGGACTGTTTTGTAAAGCGAGGTGGGACCGATGTGGCACACGCCAGCTGC

GGTTTCCCGGAGCGTGGAGAGGCAGCGCTGCTGCTCCTGCCCCAGGCTCA
GGTTTCCCGGAGCGTGGAGAGGCAGCGCTGCTGCTCCTGCCCCAGGCTCA
--------------------------------------------------
GGTTTCCCGGAGCGTGGAGAGGCAGTGCTGCTGCTCCCGCCCGAGGCTCA

TGACAACTCAATGAAGCACTGCTTTTATTTTTTGCAGTCTTCAATTTGAG
TGACAACTCAATGAAGCACTGCTTTTATTTTTTGCAGTCTTCAATTTGAG
--------------------------------------------------
TGACAACTCAATAAAGCACTGCTTTTATTTTTTGCAGTCTTCAATTTGAG

AAAGGTGAGAAATAATCTTTTCAATAAATGAGATTCATACCAAAAAAAAA
AAAGGTGAGAAATAATCTTTTCAATAAATGAGATTCATACCAAAAAAAAA
--------------------------------------------------
AAAGGTGAGAAATAATGTTTTCAATAAATGAGATTCATACCA

AAAAAAAAAGAGAgagataatgtatacagcat-------gcttactatggaggctgg
-----AAAAGAGAGAGATAATGTATACAGCAT-------GCTTACTATGGAGGCTGG
----------------------TATACAGCAT-------GCTTAgTATGGAGcCgGG
-----------------------------catgtaaagcgcttagcacagtgcctgg

cacatggtagctgctGCACAGTCACTAATGTGAGctagactgtatgctgc
CACATGGTAGCTGCTGCACAGTCACTAATGTGAGCTAGACTGTATGCTGC
CACATGGTAGCTGCTGCACAGTCACTAATGTGAGCTAGACTGTAaGCTGC
cacacagtaagcgct

tcaaggttagctgatggtcttactccattttatactggtcccaaggctta
TCAAGGTcAGCTGATGGTCTTACTCCATTTTATACTGGTCCCAAGGCTTA
TCAAGGTcAGCTGATGaTCTTACTCCATTTTATACTGGTCCCAAGGCTTA

gcactagaaatgattcccaggatcctcaatataagttggttgagtaaatg
GCACTAGAAATGATTCCCAGGATCCTCAATATAAGTTGGTTGAGTAAATG
GCACTAGAAATGATTCCCAGGAgCCTCAATATAAGTTtGTTGAaTAAATG

aaAA
```

AAAA
AAAA
