# Supplementary figures and images for: Repair-Mediated Duplication by Capture of Proximal Chromosomal DNA Has Shaped Vertebrate Genome Evolution
Source: PLoS Genet. 2009 May 8;5(5):e1000469. doi: 10.1371/journal.pgen.1000469 (PMC2671141; doi:10.1371/journal.pgen.1000469)

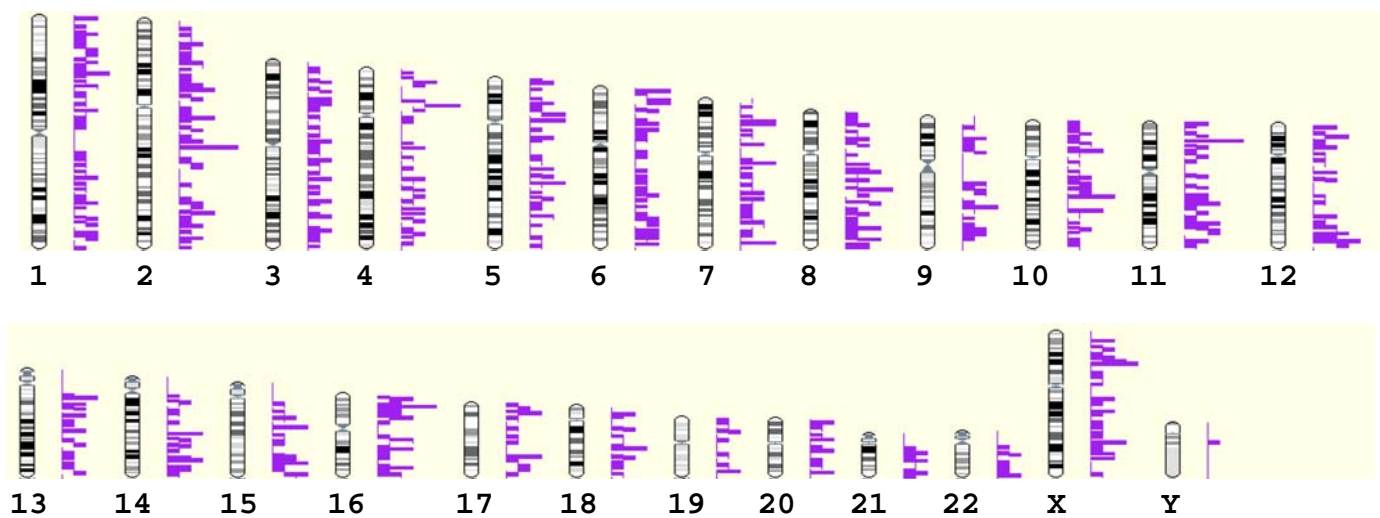

Supplement: Figure S2 — Chromosomal distribution of RDs in human. The histogram to the right of each chromosome indicates the number of RDs within the region. (0.06 MB PDF) [file pgen.1000469.s002.pdf]
