# Supplementary material for: Repair-Mediated Duplication by Capture of Proximal Chromosomal DNA Has Shaped Vertebrate Genome Evolution
Source: PLoS Genet. 2009 May 8;5(5):e1000469. doi: 10.1371/journal.pgen.1000469 (PMC2671141; doi:10.1371/journal.pgen.1000469)
Supplement: Table S2 — Loading order of gels. The numbers for each lane are designators for the 80 human subjects whose DNA forms the Coriell population panels used. (0.01 MB PDF) [file pgen.1000469.s004.pdf]

Table S2. Loading order of gels. The numbers for each lane are designators for the 80 human subjects whose DNA forms the Coriell population panels used.

Lane

1	2	3	4	5	6	7	8	9	10	11	12	13	14	15	16	17	18	19	20	21	22	23	24	25	26

African-American

100 bp LADDER	L999	**17106**	L1472	**17105**	L1454	**17104**	L1525	**17103**	L1595	**17102**	L1499	**17101**	L1604	L1035	L1473	L997	**17110**	**rhesus**	**17109**	**gorilla**	**17108**	**gorilla**	**17107**	500 bp LADDER	100 bp LADDER

Asian

100 bp LADDER	**17058**	**17086**	**17057**	**17085**	**17056**	**17084**	**17055**	**17083**	**17054**	**17082**	**17053**	**17081**	**17052**	**17060**	**17051**	**17059**	**17090**	**rhesus**	**17089**	**gorilla**	**17088**	**gorilla**	**17087**	500 bp LADDER	100 bp LADDER

European

100 bp LADDER	**10509**	**10408**	**10454**	**10434**	**10252**	**9941**	**10156**	**10345**	**10289**	**10643**	**10394**	**10406**	**10431**	**10450**	**10044**	**10635**	**10127**	**rhesus**	**10424**	**gorilla**	**10136**	**gorilla**	**10622**	500 bp LADDER	100 bp LADDER

South American

100 bp LADDER	**17308**	**17316**	**17307**	**17315**	**17306**	**17314**	**17305**	**17313**	**17304**	**17312**	**17303**	**17311**	**17302**	**17310**	**17301**	**17309**	**17320**	**rhesus**	**17318**	**gorilla**	**17319**	**gorilla**	**17317**	500 bp LADDER	100 bp LADDER
